# Global, regional, and national burden of kidney, bladder, and prostate cancers and their attributable risk factors, 1990–2019

**DOI:** 10.1186/s40779-021-00354-z

**Published:** 2021-11-24

**Authors:** Hao Zi, Shao-Hua He, Xie-Yuan Leng, Xiao-Feng Xu, Qiao Huang, Hong Weng, Cong Zhu, Lu-Yao Li, Jia-Min Gu, Xu-Hui Li, Dao-Jing Ming, Xiao-Dong Li, Shuai Yuan, Xing-Huan Wang, Da-Lin He, Xian-Tao Zeng

**Affiliations:** 1 Department of Urology, Zhongnan Hospital of Wuhan University, Wuhan, 430071 China; 2 Center for Evidence-Based and Translational Medicine, Zhongnan Hospital of Wuhan University, Wuhan, 430071 China; 3 Precision Medicine Centre, The Second People’s Hospital of Huaihua, Huaihua, 418000 China; 4 The First School of Clinical Medicine, Anhui Medical University, Hefei, 230000 China; 5 Department of Urology, Xianyang Central Hospital, Xianyang, 712000 China; 6 Department of Urology, The First Affiliated Hospital of Xi’an Jiaotong University, Xi’an, 710061 China; 7 Institutes of Evidence-Based Medicine and Knowledge Translation, Henan University, Kaifeng, 475000 China; 8 Department of Urology, Huaihe Hospital of Henan University, Kaifeng, 475000 China; 9 Institute of Urology, Wuhan University, Wuhan, 430071 China

**Keywords:** Genitourinary cancer, Kidney cancer, Bladder cancer, Prostate cancer, Incidence, Mortality, Disability-adjusted life-years, Global Burden of Disease

## Abstract

**Background:**

The burden of kidney, bladder, and prostate cancers has changed in recent decades. This study aims to investigate the global and regional burden of, and attributable risk factors for genitourinary cancers during the past 30 years.

**Methods:**

We extracted data of kidney, bladder, and prostate cancers from the Global Burden of Disease 2019 database, including incidence, mortality, disability-adjusted life-years (DALYs), and attributable risk factors from 1990 to 2019. Estimated annual percentage changes (EAPC) were calculated to assess the changes in age-standardized incidence rate, age-standardized mortality rate (ASMR), and age-standardized DALYs rate (ASDR). The associations between cancers burden and socio-demographic index (SDI) were also analyzed.

**Results:**

Compared with 1990, the global incident cases in 2019 were higher by 154.78%, 123.34%, and 169.11% for kidney, bladder, and prostate cancers, respectively. During the 30-year study period, there was a downward trend in ASMR and ASDR for bladder cancer (EAPC = − 0.68 and − 0.83, respectively) and prostate cancer (EAPC = − 0.75 and − 0.71, respectively), but an upward trend for kidney cancer (EAPC = 0.35 and 0.12, respectively). Regions and countries with higher SDI had higher incidence, mortality, and DALYs for all three types of cancers. The burden of bladder and prostate cancers was mainly distributed among older men, whereas the burden of kidney cancer increased among middle-aged men. Smoking related mortality and DALYs decreased, but high body mass index (BMI) and high fasting plasma glucose (FPG) related mortality and DALYs increased among kidney, bladder, and prostate cancers during the study period.

**Conclusions:**

Kidney, bladder, and prostate cancers remain major global public health challenges, but with distinct trend for different disease entity across different regions and socioeconomic status. More proactive intervention strategies, at both the administrative and academic levels, based on the dynamic changes, are needed.

**Supplementary Information:**

The online version contains supplementary material available at 10.1186/s40779-021-00354-z.

## Background

Kidney, bladder, and prostate cancers are the most common genitourinary cancers, with 0.39, 0.47, and 1.33 million new cases worldwide in 2017 [1]. Previous studies have reported notable heterogeneity in temporal trend of disease burden across geographic locations and socio-demographic index (SDI) levels [2–4]. Along with the aging population and socioeconomic development in recent decades, the incidence and mortality of genitourinary cancers have changed significantly [1, 5, 6].

The Global Burden of Disease (GBD) and GLOBOCAN databases are the most widely used data sources to evaluate the disease burden [7, 8]. The updated GBD 2019 evaluated 369 diseases and injuries worldwide and compared their prevalence in 204 countries and territories from 1990 to 2019, providing valuable information to adjust health policies [9–11]. Compared with GBD 2017, GBD 2019 has notable advantages in the estimation process and many other areas, including more countries, additional data sources, and new causes. To clarify the global burden of genitourinary cancers, we used the data from the GBD 2019 to analyze the incidence, mortality, disability-adjusted life-years (DALYs) of these three cancers, and also analyzed their attributable risk factors from 1990 to 2019.

## Methods

### Data source

GBD 2019 is updated and expanded on the basis of GBD 2017 and provides the most up-to-date global health data. GBD 2019 includes data on mortality and morbidity in 204 countries and territories, 369 diseases and injuries, and 87 risk factors from 1990 to 2019. Previous publications provide more details on these general GBD methods [9–11]. Briefly, the GBD 2019 data sources include household surveys, censuses, civil registration, vital statistics, disease registries, and other health-related data sources. Cause of Death Ensemble model (CODEm) is the framework used to model most cause-specific death rates. The incidence and mortality data from cancer registries were matched by cancer, age, sex, year, and location to generate mortality to incidence ratio, which in turn is used to estimate the cancer incidence. The use of mortality to incidence ratio allows a uniform method to estimate incidence. The GBD 2019 risk factors hierarchy is based on common features of individual risks. The attributable burden is assessed by comparative risk assessment framework in GBD since 2002 [10]. In this study, incidence, mortality, DALYs, and its corresponding age-standardized rates (ASR) by world standard population of kidney, bladder, and prostate cancers were gathered by the Global Health Data Exchange query tool (http://ghdx.healthdata.org/gbd-results-tool). Annual deaths and DALYs attributable to 87 risk factors are also available from the GBD results tool. The GBD world population standard was used for the calculation of ASR. The 95% uncertainty interval (UI) were reported for all estimates. The SDI is a composite index of development status strongly correlated with health outcomes, and the dataset is available online (http://ghdx.healthdata.org/gbd-2019). A region or country with an SDI of 0 would have a theoretical minimum level of development relevant to health, while a region or country with an SDI of 1 would have a theoretical maximum level. The SDI values for all estimated GBD 2019 locations from 1990 to 2019 are provided in the supplementary materials (Additional file 1: Table S1). For analysis, the SDI values were used to divide the countries into five SDI quintiles (low, low-middle, middle, high-middle, and high SDI) (Additional file 1: Table S2). In addition, age group and sex were also extracted from GBD results tool to analyze the burden of kidney, bladder, and prostate cancers.

### Cancer definition

All cancers were defined based on the International Classification of Diseases (ICD) diagnostic criteria. The associated ICD-9 and ICD-10 codes of kidney, bladder, and prostate cancers for incidence and mortality data were as follows: kidney cancer (C64-C64.2, C64.9-C65.9, Z80.51, Z85.52-Z85.54, 189–189.1, 189.5–189.6, 209.24, C64-C65.9, D30.0-D30.1, D41.0-D41.1, and 223.0–223.1), bladder cancer (C67-C67.9, Z12.6-Z12.79, Z80.52, Z85.51, 188–188.9, V10.51, V16.52, V76.3, D09.0, D30.3, D41.4-D41.8, D49.4, 223.3, 233.7, 236.7, and 239.4), and prostate cancer (C61-C61.9, Z12.5, Z80.42, Z85.46, 185–185.9, V10.46, V16.42, V76.44, D07.5, D29.1, D40.0, 222.2, and 236.5). The incidence, mortality, and DALYs estimation for genitourinary cancers were described in the GBD 2019 study [9].

### Attributable risk factors

Attributable risk factors in the analysis included smoking, high body mass index (BMI), and occupational exposure to trichloroethylene for kidney cancer; smoking and high fasting plasma glucose (FPG) for bladder cancer; and smoking for prostate cancer. The percentage of cancer related death and DALYs are available in GBD results tool. Details about the definitions of these risk factors and their relative risk for kidney, bladder, and prostate cancers were described elsewhere [10].

### Statistical analysis

The general methodology and estimation process of GBD 2019 have been described in previous publications [9, 12]. The ASR (per 100,000 population) was calculated by the sum of the products of age-specific rates (*a*_*i*_, where *i* denotes the *i*th age) and the number of population (or weight *w*_*i*_) in the same age group *i* of the selected reference standard population, divided by the sum of the standard population weights: ASR = $\frac{{\sum }_{i=1}^{A}{a}_{i}{w}_{i}}{{\sum }_{i=1}^{A}{w}_{i}}$× 100,000. We used estimated annual percentage changes (EAPC) to describe the trend of ASR within a specified time interval. The natural logarithm of ASR is linear with time. The EAPC were estimated by a linear regression model: *y* = *α* + *βx* + ε, where *y* is ln(ASR), *x* is the calendar year, and ε is the error term. The EAPC were calculated as 100 × (exp(*β*) − 1) and its 95% confidence interval (CI) can be obtained from the linear regression model. When the estimated EAPC value and its lower 95% CI were both > 0, ASR is considered as an upward trend. Conversely, if the estimated EAPC value and its upper 95% CI were both < 0, ASR is considered a downward trend. Data visualization and statistics were performed using R software (Version 4.0.5) and Microsoft Excel (Version 2016).

## Results

### Global incidence, mortality and DALYs

In 2019, the global incident cases were 371.75 × 10^3^ (95% UI 344.59–402.35) for kidney cancer, 524.30 × 10^3^ (95% UI 475.95–569.43) for bladder cancer and 1410.45 × 10^3^ (95% UI 1227.90–1825.77) for prostate cancer (Table 1). Compared with 1990, prostate cancer had the largest increase (169.11%), followed by kidney cancer (154.78%) and bladder cancer (123.34%). Between 1990 and 2019, prostate cancer had the highest percentage of incident cases among genitourinary cancers, followed by bladder and kidney cancers (Additional file 2: Fig. S1a). From 1990 to 2019, the age-standardized incidence rate (ASIR) of prostate cancer has been much higher than kidney and bladder cancers (Additional file 2: Fig. S2a). The EAPC of ASIR in genitourinary cancers showed an upward trend, except bladder cancer in women (EAPC = − 0.24, 95% CI − 0.29 to − 0.18, Table 1).

**Table 1:** Global incidence, mortality and DALYs of genitourinary cancers from 1990 to 2019

	Kidney cancer			Bladder cancer			Prostate cancer
	Both	Male	Female	Both	Male	Female	Male
1990*							
Incident cases (× 10^3^)	145.91 (140.80–150.33)	86.06 (82.70–89.44)	59.85 (57.39–62.13)	234.75 (225.46–243.08)	177.76 (170.77–184.13)	56.99 (53.45–60.31)	524.11 (409.13–613.01)
Deaths (× 10^3^)	72.10 (68.88–74.67)	43.46 (41.33–45.51)	28.64 (27.24–29.66)	121.50 (114.75–127.17)	88.12 (83.47–91.86)	33.38 (30.66–35.84)	233.00 (191.40–268.88)
DALYs (× 10^3^)	2039.40 (1943.70–2144.10)	1260.71 (1189.84–1349.99)	778.69 (738.21–829.72)	2567.06 (2429.47–2691.11)	1901.45 (1798.60–1987.27)	665.60 (613.69–722.30)	4360.51 (3528.03–4951.01)
ASIR (1/10^5^)	3.53 (3.40–3.63)	4.51 (4.34–4.67)	2.70 (2.59–2.80)	6.27 (5.98–6.50)	10.94 (10.43–11.34)	2.77 (2.59–2.93)	34.13 (26.83–39.65)
ASMR (1/10^5^)	1.86 (1.77–1.93)	2.50 (2.37–2.60)	1.36 (1.28–1.41)	3.49 (3.27–3.66)	6.13 (5.77–6.41)	1.70 (1.54–1.82)	18.13 (14.68–21.19)
ASDR (1/10^5^)	47.28 (45.22–49.37)	61.96 (58.81–65.46)	34.30 (32.65–36.08)	66.59 (63.00–69.73)	111.68 (105.56–116.61)	31.66 (29.19–34.29)	286.30 (232.78–326.21)
2019*							
Incident cases (× 10^3^)	371.75 (344.59–402.35)	240.54 (220.79–261.82)	131.21 (119.71–142.42)	524.30 (475.95–569.43)	407.87 (371.30–443.75)	116.44 (103.71–128.21)	1410.45 (1227.90–1825.77)
Deaths (× 10^3^)	166.44 (155.46–176.3)	108.77 (101.48–115.82)	57.67 (52.24–61.90)	228.73 (210.74–243.19)	169.21 (156.92–180.66)	59.53 (52.33–64.58)	486.84 (420.5–593.69)
DALYs (× 10^3^)	4052.82 (3801.04–4317.49)	2735.71 (2544.82–2931.33)	1317.11 (1228.35–1413.53)	4392.58 (4090.44–4702.73)	3326.50 (3093.91–3570.17)	1066.08 (962.71–1150.12)	8644.87 (7548.02–10,559.87)
ASIR (1/10^5^)	4.55 (4.22–4.93)	6.24 (5.74–6.79)	3.07 (2.80–3.33)	6.52 (5.93–7.09)	11.28 (10.23–12.27)	2.66 (2.37–2.93)	38.63 (33.63–49.83)
ASMR (1/10^5^)	2.08 (1.93–2.20)	2.99 (2.78–3.18)	1.33 (1.20–1.42)	2.94 (2.70–3.13)	5.09 (4.69–5.44)	1.36 (1.19–1.47)	15.28 (13.00–18.57)
ASDR (1/10^5^)	49.62 (46.46–52.94)	70.06 (65.14–75.07)	31.08 (28.95–33.38)	54.20 (50.35–57.97)	90.17 (83.63–96.61)	24.43 (22.10–26.35)	244.07 (211.78–297.72)
1990–2019							
ASIR							
EAPC (95% CI)	0.87 (0.76–0.99)	1.13 (1.01–1.26)	0.39 (0.30–0.49)	0.07 (0.04–0.10)	0.06 (0.03–0.08)	− 0.24 (− 0.29 to − 0.18)	0.26 (0.15–0.37)
ASMR							
EAPC (95% CI)	0.35 (0.26–0.45)	0.61 (0.51–0.70)	− 0.13 (− 0.20 to − 0.05)	− 0.68 (− 0.71 to − 0.65)	− 0.71 (− 0.73 to − 0.68)	− 0.89 (− 0.94 to − 0.84)	− 0.75 (− 0.84 to − 0.67)
ASDR							
EAPC (95% CI)	0.12 (0.04–0.20)	0.40 (0.30–0.49)	− 0.41 (− 0.46 to − 0.36)	− 0.83 (− 0.86 to − 0.79)	− 0.84 (− 0.87 to − 0.81)	− 1.04 (− 1.08 to − 0.99)	− 0.71 (− 0.78 to − 0.64)

*DALYs* disability-adjusted life-years, *ASIR* age-standardized incidence rate, *ASMR* age-standardized mortality rate, *ASDR* age-standardized DALYs rate; *EAPC* estimated annual percentage change, *CI* confidence interval

*All data reported as number or rate (95% uncertainty interval)

Worldwide, prostate cancer caused 486.84 × 10^3^ male deaths in 2019, which was 4.48 times of kidney cancer and 2.88 times of bladder cancer (Table 1). Compared with 1990, the percentage of deaths from prostate and kidney cancers increased, whereas the percentage of deaths from bladder cancer decreased in 2019 (Additional file 2: Fig. S1b). The age-standardized mortality rate (ASMR) for bladder cancer and prostate cancer showed a downward trend from 1990 to 2019 (Additional file 2: Fig. S2b). The ASMR for kidney cancer showed an upward trend before 2010, but gradually declined after 2010. Over the past 30 years, the EAPC of ASMR showed an upward trend only for kidney cancer (EAPC = 0.35, 95% CI 0.26–0.45, Table 1).

Prostate cancer caused 8644.87 × 10^3^ (95% UI 7548.02–10,559.87) DALYs in 2019, more than the sum of bladder and kidney cancers (Table 1). The percentage of DALYs due to prostate and kidney cancers continued to expand from 1990 to 2019 (Additional file 2: Fig. S1c). The trend of age-standardized DALYs rate (ASDR) was consistent with ASMR for all three cancers (Additional file 2: Fig. S2c). The downward trend of EAPC of ASDR was most prominent for bladder cancer in women (EAPC = − 1.04, 95% CI − 0.1.08 to − 0.99, Table 1).

### Regional incidence, mortality and DALYs

**Fig. 1:**
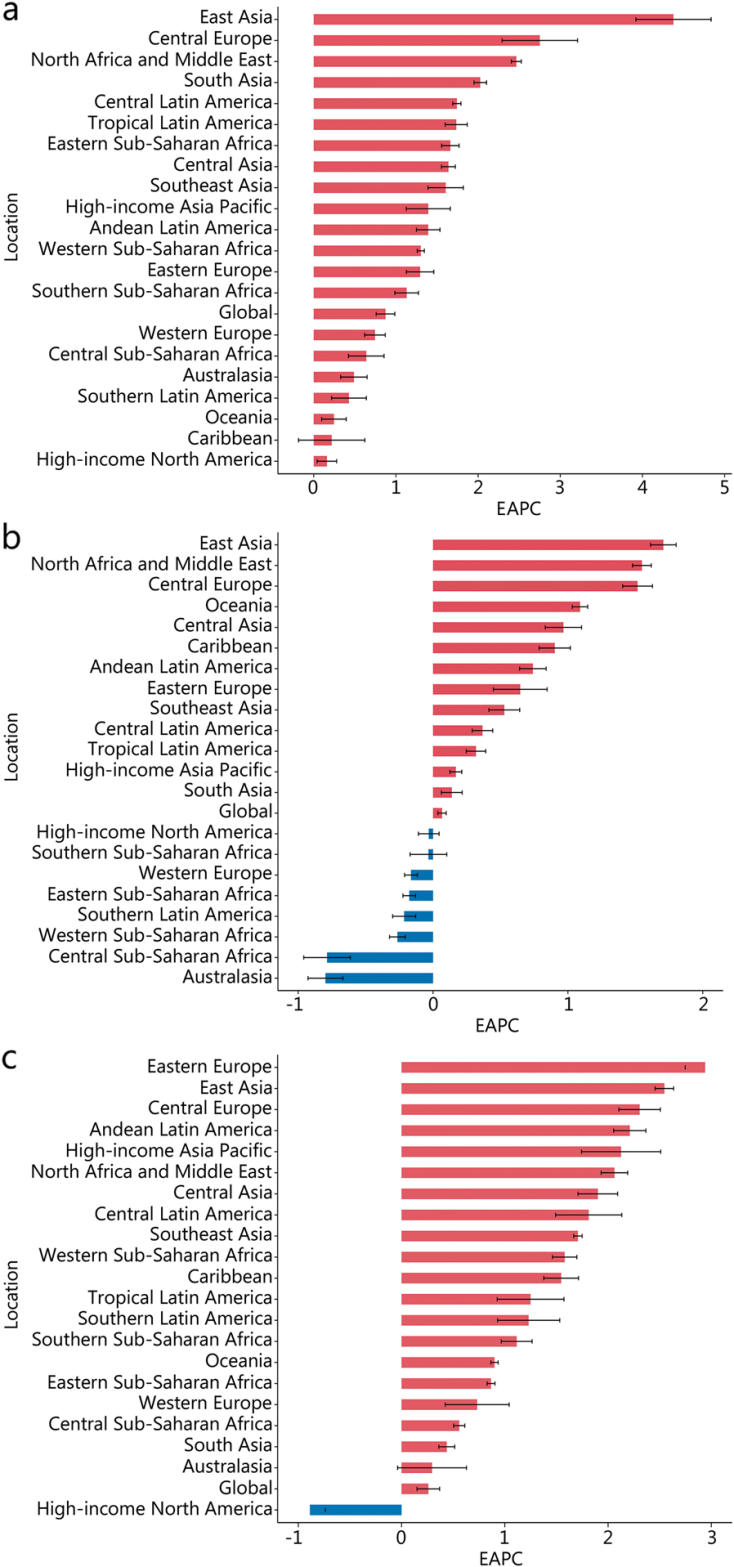
EAPC of ASIR for genitourinary cancers in global and 21 regions. **a** Kidney cancer. **b** Bladder cancer. **c** Prostate cancer. ASIR age-standardized incidence rate, EAPC estimated annual percentage change

In 2019, the regions with the most incident cases of prostate cancer were High-income North America (331.89 × 10^3^, 95% UI 262.39–494.58), Western Europe (325.49 × 10^3^, 95% UI 267.13–469.92), and East Asia (161.97 × 10^3^, 95% UI 126.34–213.69) (Additional file 1: Table S3). Most of the new cases of bladder and kidney cancers were also distributed in these three regions. In addition, the ASIR of bladder and kidney cancers showed the most significant upward trend in East Asia, whereas the ASIR of prostate cancer showed a downward trend only in High-income North America (Fig. 1).

Western Europe had the most deaths of prostate (95.77 × 10^3^, 95% UI 79.23–133.05), bladder (50.51 × 10^3^, 95% UI 45.16–54.46) and kidney cancers (34.36 × 10^3^, 95% UI 31.51–36.3) in 2019 (Additional file 1: Table S4). The highest ASMR of kidney, bladder, and prostate cancers were Southern Latin America (5.05, 95% UI 4.59–5.51), Central Europe (5.29, 95% UI 4.66–5.99), and Southern Sub-Saharan Africa (45.08, 95% UI 34.35–50.53). Stark regional differences were observed in ASMR (Additional file 2: Fig. S3). For example, the fastest increase of ASMR in kidney cancer was observed in East Asia, followed by Central Europe, North Africa and Middle East, and Eastern Sub-Saharan Africa.

The highest DALYs of kidney and bladder cancers were observed in East Asia, and prostate cancer was observed in High-income North America in 2019 (Additional file 1: Table S5). The ASDR of kidney, bladder, and prostate cancers were highest in Southern Latin America (124.32, 95% UI 113.32–135.49), Central Europe (107.36, 95% UI 93.95–122.07), and Southern Sub-Saharan Africa (726.31, 95% UI 574.70–822.50). From 1990 to 2019, East Asia, Oceania, and Western Sub-Saharan Africa had the fastest increase in ASDR for kidney, bladder and prostate cancers, respectively (Additional file 2: Fig. S4).

### National incidence, mortality and DALYs

In 2019, USA (61,541.43), China (100,020.20), and USA (308,584.29) had the most incident cases of kidney cancer, bladder cancer, and prostate cancer, respectively (Additional file 1: Table S6). China had the most deaths of kidney cancer (23,954.24), bladder cancer (40,094.24), and prostate cancer (54,390.88) among 204 countries and territories. The most DALYs caused by kidney cancer (642,799.34), bladder cancer (816,119.14), and prostate cancer (1,002,594.87) were also found in China, which were 1.5 times, 2.1 times, and 1.1 times higher than USA.

The trend of ASIR, ASMR, and ASDR varied significantly cross the 204 countries and territories (Table 2, Additional file 1: Table S7, and Additional file 1: Table S8). Bulgaria (EAPC = 6.24, 95% CI 5.32–7.16), Cabo Verde (EAPC = 3.88, 95% CI 3.19–4.57), and Estonia (EAPC = 4.31, 95% CI 3.94–4.69) had the highest increase rate in the ASIR of kidney, bladder, and prostate cancers, respectively. The ASMR of kidney, bladder, and prostate cancers showed the most significant increase in Bulgaria (EAPC = 5.83, 95% CI 4.92–6.75), Northern Mariana Islands (EAPC = 3.22, 95% CI 2.62–3.84), and Cabo Verde (EAPC = 2.53, 95% CI 1.74–3.33), respectively. Bulgaria (EAPC = 5.71, 95% CI 4.80–6.62), Northern Mariana Islands (EAPC = 3.37, 95% CI 2.73–4.02), and Georgia (EAPC = 2.67, 95% CI 2.07–3.27) had the most significant increase in ASDR for kidney, bladder, and prostate cancers, respectively.

**Table 2:** EAPC of ASIR for genitourinary cancers in 204 countries and territories from 1990 to 2019

Location	EAPC (95% CI)		
	Kidney cancer	Bladder cancer	Prostate cancer
Afghanistan	1.36 (1.10–1.62)	− 0.06 (− 0.14 to 0.02)	0.59 (0.50–0.68)
Albania	3.33 (2.99–3.67)	1.09 (0.95–1.24)	1.56 (1.47–1.65)
Algeria	1.77 (1.68–1.86)	0.49 (0.18–0.81)	0.72 (0.62–0.82)
American Samoa	0.26 (− 0.09 to 0.62)	2.11 (1.81–2.41)	0.79 (0.64–0.94)
Andorra	1.09 (0.92–1.26)	− 0.08 (− 0.18 to 0.02)	1.12 (0.91–1.33)
Angola	1.61 (1.48–1.74)	0.10 (0.05–0.15)	1.07 (1.04–1.10)
Antigua and Barbuda	− 0.01 (− 0.43 to 0.42)	1.03 (0.80–1.27)	0.90 (0.52–1.28)
Argentina	0.13 (− 0.13 to 0.39)	− 0.47 (− 0.59 to − 0.34)	0.91 (0.58–1.23)
Armenia	6.20 (5.32–7.08)	0.79 (0.54–1.04)	3.02 (2.90–3.13)
Australia	0.45 (0.24–0.65)	− 0.70 (− 0.84 to − 0.57)	0.51 (0.12–0.90)
Austria	− 0.69 (− 0.75 to − 0.63)	− 0.08 (− 0.14 to − 0.01)	0.27 (− 0.04 to 0.58)
Azerbaijan	0.72 (0.40–1.05)	1.17 (1.02–1.31)	1.63 (1.48–1.77)
Bahamas	− 0.15 (− 0.34 to 0.05)	0.55 (0.45–0.66)	0.61 (0.42–0.81)
Bahrain	0.41 (− 0.08 to 0.89)	− 0.51 (− 0.73 to − 0.29)	1.31 (1.04–1.58)
Bangladesh	1.32 (1.22–1.42)	0.37 (0.23–0.50)	0.28 (0.13–0.43)
Barbados	− 0.30 (− 0.61 to 0.01)	0.87 (0.77–0.98)	0.94 (0.61–1.27)
Belarus	4.78 (3.90–5.66)	− 0.30 (− 0.65 to 0.05)	3.04 (2.86–3.21)
Belgium	0.74 (0.46–1.02)	− 0.27 (− 0.48 to − 0.06)	0.03 (− 0.30 to 0.37)
Belize	0.32 (0.21–0.44)	1.30 (0.95–1.65)	1.46 (0.93–1.99)
Benin	1.84 (1.68–2.01)	− 1.64 (− 1.76 to − 1.52)	2.11 (1.92–2.29)
Bermuda	− 0.84 (− 1.20 to − 0.47)	0.30 (0.15–0.44)	1.68 (1.50–1.87)
Bhutan	3.00 (2.91–3.09)	1.60 (1.56–1.64)	1.98 (1.90–2.06)
Bolivia (Plurinational State of)	2.06 (1.99–2.14)	0.72 (0.61–0.83)	1.90 (1.81–1.99)
Bosnia and Herzegovina	3.07 (2.77–3.38)	2.70 (2.41–2.98)	2.85 (2.52–3.18)
Botswana	2.17 (1.79–2.56)	0.34 (0.06–0.62)	1.07 (0.67–1.47)
Brazil	1.75 (1.61–1.89)	0.30 (0.23–0.37)	1.22 (0.90–1.55)
Brunei Darussalam	2.29 (2.06–2.51)	0.25 (0.10–0.40)	2.66 (2.28–3.04)
Bulgaria	6.24 (5.32–7.16)	3.07 (2.65–3.49)	2.94 (2.65–3.23)
Burkina Faso	1.80 (1.70–1.90)	− 1.91 (− 2.15 to − 1.67)	2.22 (2.10–2.34)
Burundi	0.56 (0.49–0.64)	− 1.36 (− 1.48 to − 1.25)	0.14 (0.07–0.21)
Cabo Verde	5.40 (4.73–6.07)	3.88 (3.19–4.57)	3.63 (2.89–4.38)
Cambodia	2.14 (2.02–2.26)	0.69 (0.49–0.88)	1.99 (1.87–2.10)
Cameroon	1.30 (1.26–1.34)	− 0.70 (− 0.79 to − 0.61)	2.33 (2.18–2.47)
Canada	1.55 (1.29–1.82)	− 0.43 (− 0.56 to − 0.31)	− 1.14 (− 1.50 to − 0.78)
Central African Republic	0.40 (0.29–0.50)	− 0.87 (− 0.91 to − 0.83)	0.14 (0.08–0.19)
Chad	1.60 (1.54–1.66)	− 0.47 (− 0.59 to − 0.35)	2.42 (2.24–2.61)
Chile	1.63 (1.50–1.77)	1.26 (1.16–1.36)	2.14 (1.86–2.41)
China	4.46 (3.97–4.96)	1.74 (1.63–1.84)	2.54 (2.44–2.63)
Colombia	1.82 (1.73–1.92)	− 0.60 (− 0.71 to − 0.48)	1.09 (0.77–1.41)
Comoros	1.51 (1.40–1.63)	− 0.08 (− 0.20 to 0.05)	0.58 (0.50–0.65)
Congo	0.81 (0.62–1.00)	− 0.29 (− 0.52 to − 0.05)	0.33 (0.17–0.49)
Cook Islands	0.64 (0.54–0.74)	0.77 (0.61–0.93)	0.19 (0.12–0.27)
Costa Rica	2.69 (2.45–2.93)	0.19 (0.04–0.35)	2.36 (2.10–2.63)
Croatia	4.35 (3.69–5.00)	1.41 (1.28–1.54)	1.81 (1.61–2.02)
Cuba	− 0.31 (− 0.76 to 0.14)	1.30 (1.14–1.45)	2.02 (1.92–2.11)
Cyprus	4.24 (3.82–4.66)	1.98 (1.72–2.25)	2.92 (2.46–3.39)
Czechia	1.22 (0.70–1.74)	0.55 (0.40–0.69)	1.71 (1.27–2.15)
Cote d'Ivoire	1.04 (0.97–1.12)	− 1.62 (− 1.76 to − 1.48)	1.88 (1.73–2.03)
Democratic People's Republic of Korea	0.44 (0.21–0.68)	0.01 (− 0.16 to 0.17)	0.59 (0.44–0.74)
Democratic Republic of the Congo	0.05 (− 0.23 to 0.34)	− 1.11 (− 1.33 to − 0.89)	0.39 (0.33–0.44)
Denmark	1.71 (1.16–2.27)	− 0.01 (− 0.46 to 0.45)	2.26 (1.78–2.74)
Djibouti	2.69 (2.55–2.82)	0.59 (0.51–0.67)	0.99 (0.94–1.05)
Dominica	0.21 (− 0.06 to 0.48)	0.81 (0.72–0.89)	0.72 (0.48–0.96)
Dominican Republic	2.59 (2.08–3.11)	2.09 (1.95–2.24)	2.44 (1.88–3.00)
Ecuador	2.68 (2.30–3.05)	1.35 (1.13–1.57)	2.23 (1.95–2.50)
Egypt	2.44 (2.29–2.59)	1.53 (1.40–1.67)	1.53 (1.35–1.71)
El Salvador	2.19 (2.02–2.36)	1.25 (1.08–1.42)	2.92 (2.38–3.47)
Equatorial Guinea	4.64 (4.36–4.93)	1.83 (1.64–2.01)	2.02 (1.92–2.13)
Eritrea	2.14 (1.85–2.44)	0.39 (0.19–0.59)	0.46 (0.23–0.70)
Estonia	4.57 (3.81–5.34)	1.39 (1.21–1.57)	4.31 (3.94–4.69)
Eswatini	1.80 (1.17–2.43)	0.52 (0.36–0.68)	1.14 (0.96–1.32)
Ethiopia	0.79 (0.49–1.09)	− 0.26 (− 0.40 to − 0.12)	1.13 (0.98–1.29)
Fiji	0.59 (0.46–0.73)	1.49 (1.22–1.76)	0.96 (0.80–1.13)
Finland	0.57 (0.43–0.70)	− 0.44 (− 0.52 to − 0.35)	1.50 (0.99–2.01)
France	0.87 (0.80–0.94)	0.27 (0.16–0.37)	0.08 (− 0.26 to 0.43)
Gabon	1.92 (1.85–1.99)	0.01 (− 0.09 to 0.09)	1.04 (0.98–1.10)
Gambia	1.57 (1.38–1.76)	0.63 (0.48–0.78)	0.98 (0.87–1.08)
Georgia	2.02 (1.59–2.45)	2.04 (1.72–2.36)	2.94 (2.37–3.51)
Germany	0.37 (0.22–0.52)	− 0.62 (− 0.87 to − 0.37)	0.92 (0.58–1.26)
Ghana	− 0.02 (− 0.38 to 0.33)	− 0.82 (− 1.01 to − 0.64)	0.07 (− 0.13 to 0.27)
Greece	0.84 (0.62–1.05)	− 0.25 (− 0.33 to − 0.17)	0.40 (0.05–0.75)
Greenland	2.15 (1.77–2.53)	− 0.86 (− 1.00 to − 0.72)	0.85 (0.74–0.95)
Grenada	0.51 (0.06–0.96)	1.11 (0.96–1.26)	1.58 (0.84–2.33)
Guam	− 0.86 (− 1.20 to − 0.51)	0.87 (0.61–1.13)	− 0.34 (− 0.52 to − 0.16)
Guatemala	2.30 (1.77–2.83)	0.20 (0.08–0.33)	3.06 (2.31–3.81)
Guinea	1.33 (1.25–1.41)	0.63 (0.55–0.71)	1.21 (1.06–1.36)
Guinea-Bissau	0.82 (0.70–0.93)	− 1.20 (− 1.31 to − 1.09)	2.12 (1.98–2.27)
Guyana	0.04 (− 0.22 to 0.30)	0.51 (0.43–0.58)	0.57 (0.40–0.75)
Haiti	− 0.15 (− 0.44 to 0.15)	0.06 (− 0.07 to 0.20)	0.51 (0.47–0.54)
Honduras	2.82 (2.67–2.97)	2.15 (1.97–2.32)	2.93 (2.64–3.23)
Hungary	0.49 (0.14–0.84)	0.95 (0.68–1.22)	0.73 (0.48–0.98)
Iceland	0.73 (0.43–1.03)	− 0.64 (− 0.79 to − 0.50)	0.20 (− 0.18 to 0.58)
India	2.02 (1.93–2.11)	0.41 (0.31–0.52)	0.45 (0.36–0.54)
Indonesia	2.67 (2.63–2.71)	1.09 (1.02–1.17)	2.52 (2.46–2.59)
Iran (Islamic Republic of)	1.99 (1.88–2.11)	1.47 (1.36–1.58)	2.01 (1.80–2.21)
Iraq	2.71 (2.40–3.02)	2.18 (1.85–2.51)	1.98 (1.75–2.22)
Ireland	2.02 (1.67–2.36)	0.74 (0.62–0.87)	1.20 (0.81–1.59)
Israel	0.78 (0.43–1.12)	0.70 (0.44–0.95)	0.50 (0.09–0.91)
Italy	0.62 (0.45–0.80)	− 0.50 (− 0.62 to − 0.39)	0.62 (0.25–0.99)
Jamaica	− 0.32 (− 0.75 to 0.10)	0.17 (− 0.09 to 0.43)	2.63 (1.94–3.31)
Japan	1.16 (0.95–1.36)	0.20 (0.14–0.27)	2.10 (1.72–2.49)
Jordan	3.39 (3.18–3.60)	1.36 (1.25–1.48)	2.22 (1.97–2.46)
Kazakhstan	0.73 (0.40–1.06)	0.32 (0.03–0.61)	1.97 (1.65–2.29)
Kenya	2.78 (2.61–2.94)	1.36 (1.22–1.51)	2.32 (2.11–2.53)
Kiribati	0.30 (0.06–0.54)	0.08 (0.03–0.12)	− 0.26 (− 0.42 to − 0.09)
Kuwait	0.47 (− 0.05 to 1.00)	1.06 (0.73–1.39)	2.40 (2.13–2.67)
Kyrgyzstan	1.72 (1.30–2.15)	0.01 (− 0.26 to 0.28)	− 0.40 (− 0.69 to − 0.11)
Lao People's Democratic Republic	1.43 (1.35–1.51)	− 0.26 (− 0.44 to − 0.07)	0.95 (0.87–1.02)
Latvia	4.21 (3.52–4.90)	1.68 (1.46–1.91)	3.47 (3.06–3.87)
Lebanon	4.09 (3.85–4.34)	1.83 (1.71–1.94)	3.60 (3.43–3.77)
Lesotho	3.16 (3.01–3.31)	1.48 (1.37–1.60)	1.53 (1.36–1.70)
Liberia	1.34 (0.77–1.92)	− 1.60 (− 1.71 to − 1.49)	2.16 (1.94–2.39)
Libya	2.21 (1.99–2.44)	1.30 (1.10–1.50)	1.64 (1.46–1.81)
Lithuania	3.80 (3.05–4.55)	− 0.05 (− 0.28 to 0.18)	3.51 (2.91–4.11)
Luxembourg	0.02 (− 0.20 to 0.23)	− 0.07 (− 0.27 to 0.12)	− 0.06 (− 0.35 to 0.24)
Madagascar	1.01 (0.92–1.10)	− 1.03 (− 1.20 to − 0.86)	− 0.05 (− 0.19 to 0.09)
Malawi	1.31 (1.21–1.41)	0.03 (− 0.06 to 0.12)	1.30 (1.17–1.44)
Malaysia	2.43 (2.33–2.52)	0.73 (0.49–0.96)	1.66 (1.50–1.82)
Maldives	2.24 (1.96–2.52)	0.31 (0.19–0.42)	2.51 (2.43–2.59)
Mali	1.67 (1.56–1.77)	0.13 (0.03–0.22)	1.02 (0.96–1.08)
Malta	1.43 (1.25–1.61)	− 0.39 (− 0.46 to − 0.32)	0.75 (0.50–0.99)
Marshall Islands	0.78 (0.70–0.85)	0.94 (0.84–1.04)	0.28 (0.05–0.52)
Mauritania	0.90 (0.82–0.98)	− 1.56 (− 1.69 to − 1.43)	2.01 (1.86–2.15)
Mauritius	2.07 (1.83–2.31)	− 1.01 (− 1.24 to − 0.77)	1.09 (0.69–1.50)
Mexico	1.8 (1.67–1.93)	0.55 (0.46–0.64)	1.49 (1.22–1.77)
Micronesia (Federated States of)	0.69 (0.55–0.82)	1.22 (1.16–1.28)	1.1 (1.00–1.19)
Monaco	1.62 (1.39–1.85)	1.24 (1.21–1.27)	1.09 (0.91–1.27)
Mongolia	3.73 (3.43–4.03)	− 2.26 (− 2.68 to − 1.83)	0.80 (0.72–0.88)
Montenegro	1.75 (1.63–1.86)	1.10 (1.01–1.19)	1.83 (1.67–2.00)
Morocco	2.55 (2.41–2.70)	1.57 (1.29–1.85)	1.68 (1.31–2.05)
Mozambique	2.81 (2.66–2.96)	0.23 (0.11–0.36)	1.57 (1.51–1.64)
Myanmar	2.10 (2.03–2.17)	− 0.06 (− 0.16 to 0.04)	1.45 (1.38–1.51)
Namibia	2.34 (2.17–2.50)	0.85 (0.70–1.01)	2.93 (2.77–3.09)
Nauru	− 0.21 (− 0.45 to 0.03)	0.68 (0.58–0.78)	0.97 (0.90–1.03)
Nepal	2.91 (2.67–3.15)	1.01 (0.87–1.15)	1.55 (1.40–1.71)
Netherlands	1.12 (0.92–1.31)	0.42 (0.25–0.58)	1.00 (0.77–1.24)
New Zealand	0.72 (0.58–0.85)	− 1.36 (− 1.61 to − 1.10)	− 0.66 (− 0.88 to − 0.43)
Nicaragua	2.80 (2.57–3.03)	1.05 (0.80–1.30)	2.85 (2.72–2.97)
Niger	0.22 (0.12–0.31)	− 1.43 (− 1.56 to − 1.30)	2.36 (2.19–2.53)
Nigeria	1.56 (1.44–1.67)	0.99 (0.85–1.13)	1.61 (1.44–1.78)
Niue	1.29 (1.08–1.49)	1.31 (1.18–1.43)	1.80 (1.69–1.91)
North Macedonia	5.07 (4.51–5.63)	1.67 (1.43–1.91)	2.85 (2.65–3.06)
Northern Mariana Islands	− 0.27 (− 0.79 to 0.26)	3.67 (3.03–4.32)	1.76 (1.48–2.05)
Norway	1.35 (1.04–1.66)	− 0.29 (− 0.46 to − 0.13)	1.38 (0.97–1.79)
Oman	3.74 (3.30–4.19)	2.04 (1.73–2.35)	2.17 (1.87–2.47)
Pakistan	2.56 (2.35–2.77)	0.92 (0.84–1.01)	1.08 (0.97–1.19)
Palau	0.95 (0.75–1.16)	0.76 (0.64–0.88)	0.69 (0.64–0.74)
Palestine	1.65 (1.44–1.86)	0.60 (0.47–0.73)	0.81 (0.65–0.98)
Panama	3.35 (3.17–3.54)	− 0.59 (− 0.81 to − 0.36)	1.63 (1.17–2.09)
Papua New Guinea	0.63 (0.55–0.72)	1.15 (1.10–1.20)	1.24 (1.19–1.29)
Paraguay	0.90 (0.73–1.06)	1.27 (1.16–1.37)	2.44 (2.10–2.79)
Peru	0.82 (0.58–1.07)	0.48 (0.32–0.64)	2.33 (2.16–2.50)
Philippines	0.82 (0.59–1.05)	− 0.36 (− 0.60 to − 0.12)	0.26 (0.04–0.48)
Poland	4.47 (3.43–5.52)	1.61 (1.41–1.81)	2.39 (2.08–2.70)
Portugal	1.18 (0.95–1.40)	1.36 (1.26–1.45)	0.96 (0.70–1.22)
Puerto Rico	0.78 (0.58–0.98)	0.80 (0.61–0.98)	0.54 (0.41–0.67)
Qatar	1.91 (1.54–2.28)	3.63 (3.29–3.96)	3.23 (2.94–3.52)
Republic of Korea	3.27 (2.54–4.00)	0.64 (0.40–0.87)	3.46 (3.05–3.86)
Republic of Moldova	2.56 (2.20–2.93)	0.59 (0.33–0.86)	3.34 (2.80–3.88)
Romania	2.68 (2.59–2.77)	1.82 (1.67–1.96)	3.10 (2.96–3.24)
Russian Federation	0.87 (0.68–1.05)	0.51 (0.28–0.74)	3.47 (3.22–3.73)
Rwanda	1.05 (0.81–1.29)	− 1.14 (− 1.36 to − 0.91)	0.46 (0.27–0.65)
Saint Kitts and Nevis	− 1.18 (− 1.68 to − 0.67)	0.31 (0.16–0.46)	1.32 (1.06–1.58)
Saint Lucia	− 0.55 (− 0.93 to − 0.16)	0.07 (− 0.13 to 0.27)	0.46 (0.24–0.68)
Saint Vincent and the Grenadines	− 0.52 (− 0.96 to − 0.09)	0.77 (0.58–0.96)	0.66 (0.44–0.88)
Samoa	− 0.11 (− 0.26 to 0.05)	0.45 (0.38–0.52)	− 0.14 (− 0.27 to − 0.01)
San Marino	1.49 (1.40–1.59)	0.61 (0.55–0.68)	1.09 (1.00–1.18)
Sao Tome and Principe	1.36 (1.00–1.72)	1.62 (1.54–1.71)	2.02 (1.87–2.18)
Saudi Arabia	4.17 (3.96–4.38)	1.37 (1.10–1.64)	1.60 (1.37–1.83)
Senegal	1.09 (0.88–1.31)	− 1.08 (− 1.20 to − 0.97)	2.39 (2.19–2.59)
Serbia	2.12 (1.97–2.26)	1.95 (1.84–2.06)	2.87 (2.62–3.12)
Seychelles	1.28 (0.82–1.73)	0.93 (0.77–1.10)	2.19 (1.81–2.56)
Sierra Leone	1.20 (1.00–1.41)	− 1.97 (− 2.15 to − 1.79)	2.21 (2.04–2.39)
Singapore	1.35 (1.13–1.56)	− 0.99 (− 1.19 to − 0.8)	2.04 (1.79–2.29)
Slovakia	2.38 (2.01–2.75)	1.17 (0.91–1.42)	2.55 (2.37–2.72)
Slovenia	2.69 (2.35–3.03)	0.62 (0.43–0.81)	2.92 (2.59–3.25)
Solomon Islands	0.77 (0.54–0.99)	1.17 (1.08–1.26)	0.99 (0.92–1.07)
Somalia	0.92 (0.82–1.03)	− 0.31 (− 0.36 to − 0.26)	0.14 (0.08–0.19)
South Africa	0.93 (0.79–1.08)	0.04 (− 0.15 to 0.24)	1.18 (1.01–1.35)
South Sudan	0.91 (0.80–1.01)	− 0.29 (− 0.36 to − 0.23)	0.14 (0.11–0.17)
Spain	1.47 (1.35–1.58)	0.21 (0.05–0.36)	0.88 (0.47–1.30)
Sri Lanka	− 1.14 (− 2.1 to − 0.17)	2.72 (2.47–2.97)	2.03 (1.86–2.20)
Sudan	3.28 (3.00–3.56)	0.29 (0.17–0.41)	1.44 (1.34–1.53)
Suriname	0.16 (− 0.29 to 0.61)	1.00 (0.78–1.23)	1.52 (1.34–1.69)
Sweden	− 0.72 (− 0.83 to − 0.62)	0.61 (0.45–0.77)	0.23 (− 0.13 to 0.59)
Switzerland	1.22 (0.57–1.88)	0.79 (0.38–1.20)	− 0.45 (− 0.67 to − 0.23)
Syrian Arab Republic	2.52 (2.32–2.71)	1.78 (1.61–1.94)	1.75 (1.60–1.90)
Taiwan (Province of China)	4.51 (3.84–5.18)	1.32 (1.10–1.54)	3.47 (3.15–3.80)
Tajikistan	0.89 (0.52–1.25)	1.08 (0.89–1.27)	1.29 (1.14–1.45)
Thailand	0.93 (0.57–1.29)	− 1.50 (− 1.87 to − 1.12)	1.17 (1.04–1.30)
Timor-Leste	2.61 (2.29–2.92)	1.04 (0.77–1.31)	2.48 (2.30–2.66)
Togo	0.94 (0.88–1.01)	− 1.9 (− 2.06 to − 1.75)	2.25 (2.100–2.4)
Tokelau	1.40 (1.33–1.48)	1.17 (1.14–1.21)	1.19 (1.15–1.24)
Tonga	0.84 (0.38–1.30)	1.16 (0.98–1.34)	0.65 (0.45–0.84)
Trinidad and Tobago	− 1.61 (− 2.13 to − 1.09)	0.39 (0.02–0.76)	0.24 (0.08–0.40)
Tunisia	2.49 (2.45–2.53)	1.23 (1.13–1.34)	2.11 (2.04–2.18)
Turkey	2.15 (2.02–2.28)	2.20 (1.95–2.46)	2.45 (2.01–2.90)
Turkmenistan	4.38 (3.94–4.82)	2.60 (2.35–2.86)	1.06 (0.93–1.20)
Tuvalu	0.48 (0.34–0.63)	0.77 (0.71–0.83)	0.50 (0.42–0.58)
Uganda	3.52 (3.25–3.80)	0.39 (0.30–0.48)	1.45 (1.26–1.63)
Ukraine	1.64 (1.42–1.86)	1.19 (1.00–1.37)	1.07 (0.88–1.27)
United Arab Emirates	2.18 (1.86–2.50)	− 0.08 (− 0.51 to 0.35)	0.75 (0.36–1.14)
United Kingdom	1.20 (1.08–1.31)	− 0.85 (− 1.03 to − 0.68)	1.25 (1.07–1.43)
United Republic of Tanzania	2.24 (2.11–2.38)	− 0.20 (− 0.27 to − 0.13)	0.50 (0.46–0.54)
United States of America	0.05 (− 0.08 to 0.17)	0.01 (− 0.07 to 0.08)	− 0.84 (− 0.99 to − 0.70)
United States Virgin Islands	1.65 (1.42–1.89)	2.23 (1.84–2.62)	2.45 (2.11–2.79)
Uruguay	0.59 (0.45–0.73)	− 0.29 (− 0.41 to − 0.17)	1.12 (0.72–1.53)
Uzbekistan	3.30 (3.15–3.45)	2.09 (1.82–2.36)	2.14 (1.89–2.38)
Vanuatu	0.65 (0.50–0.80)	1.31 (1.18–1.43)	0.79 (0.74–0.85)
Venezuela (Bolivarian Republic of)	0.99 (0.65–1.32)	0.93 (0.70–1.16)	2.67 (2.06–3.28)
Viet Nam	3.22 (3.07–3.37)	2.20 (2.14–2.27)	2.46 (2.41–2.51)
Yemen	2.52 (2.33–2.70)	0.88 (0.78–0.97)	1.62 (1.50–1.75)
Zambia	1.78 (1.74–1.83)	− 0.01 (− 0.09 to 0.06)	0.75 (0.68–0.83)
Zimbabwe	1.00 (0.60–1.41)	0.22 (0.05–0.39)	0.84 (0.58–1.10)

*ASIR* age-standardized incidence rate, *EAPC* estimated annual percentage change, *CI* confidence interval

### Burden of genitourinary cancers by SDI

From 1990 to 2019, the high SDI quintile had the most incident cases and the highest ASIR in kidney, bladder, and prostate cancers (Additional file 2: Figs. S5–7). The ASMR of bladder cancer and prostate cancer decreased significantly in high and high-middle SDI quintiles, whereas the kidney cancer remained relatively stable. It was worth noting that the ASDR of prostate cancer in low SDI quintile surpassed high SDI quintile in 2011.

**Fig. 2:**
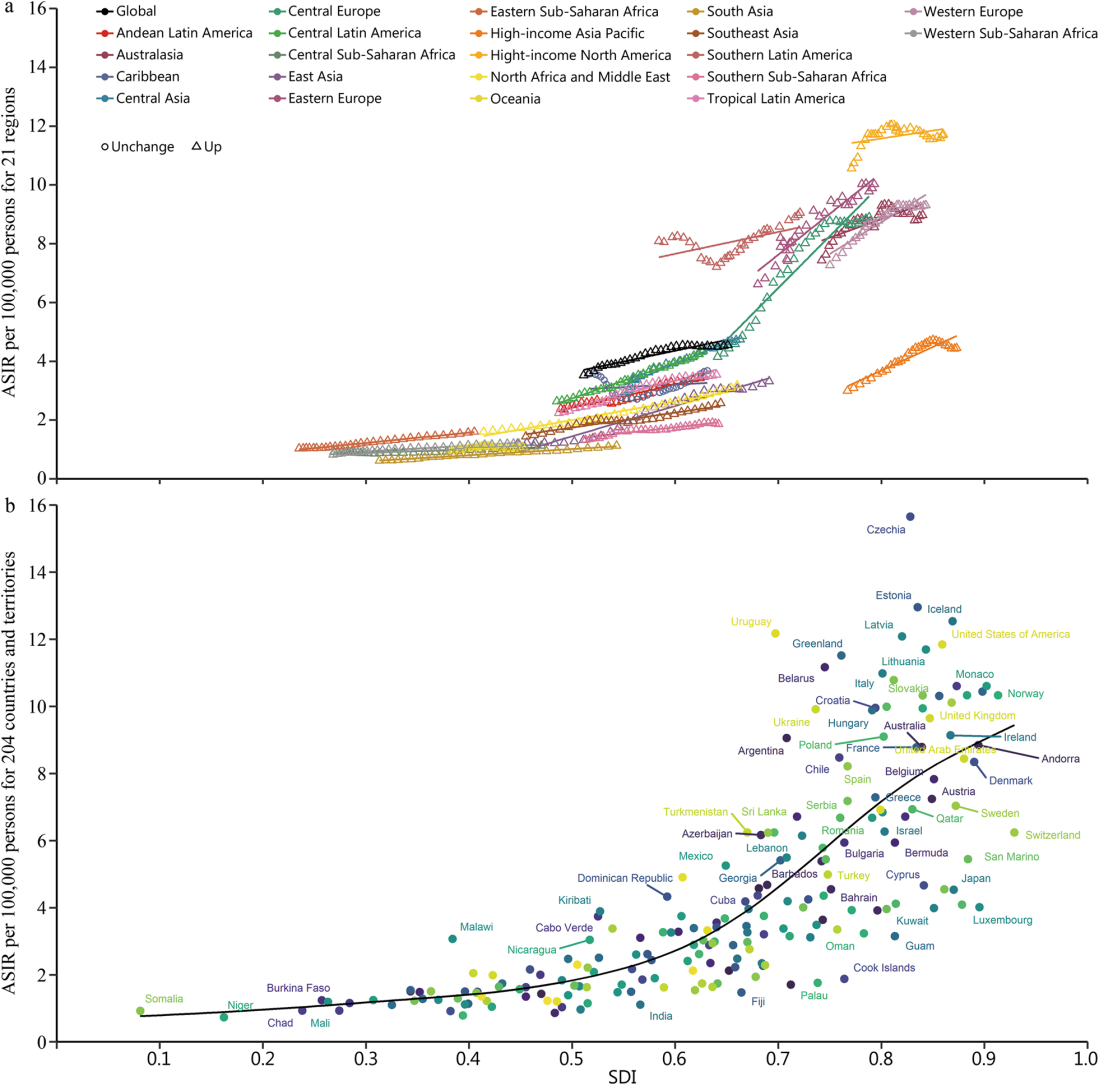
ASIR of kidney cancer for 21 regions and 204 countries and territories by SDI. **a** 21 regions by SDI from 1990 to 2019. **b** 204 countries and territories by SDI in 2019. ASIR age-standardized incidence rate, SDI sociodemographic index

High-level SDI regions had higher ASIR of kidney cancer (Fig. 2a). Similarly, the ASMR and ASDR of kidney cancer in high-level SDI regions were higher than low-level SDI regions (Additional file 2: Figs. S8–9). Among the 204 countries and territories, the ASIR of kidney cancer in Czechia was much higher than the expected levels in 2019 (Fig. 2b). Association between ASIR and SDI was also found at the regional and national level for bladder and prostate cancers (Additional file 2: Figs. S10–11). No associations among ASMR, ASDR and SDI were observed for bladder and prostate cancers (Additional file 2: Figs. S12–15).

### Burden of genitourinary cancers by age and sex

**Fig. 3:**
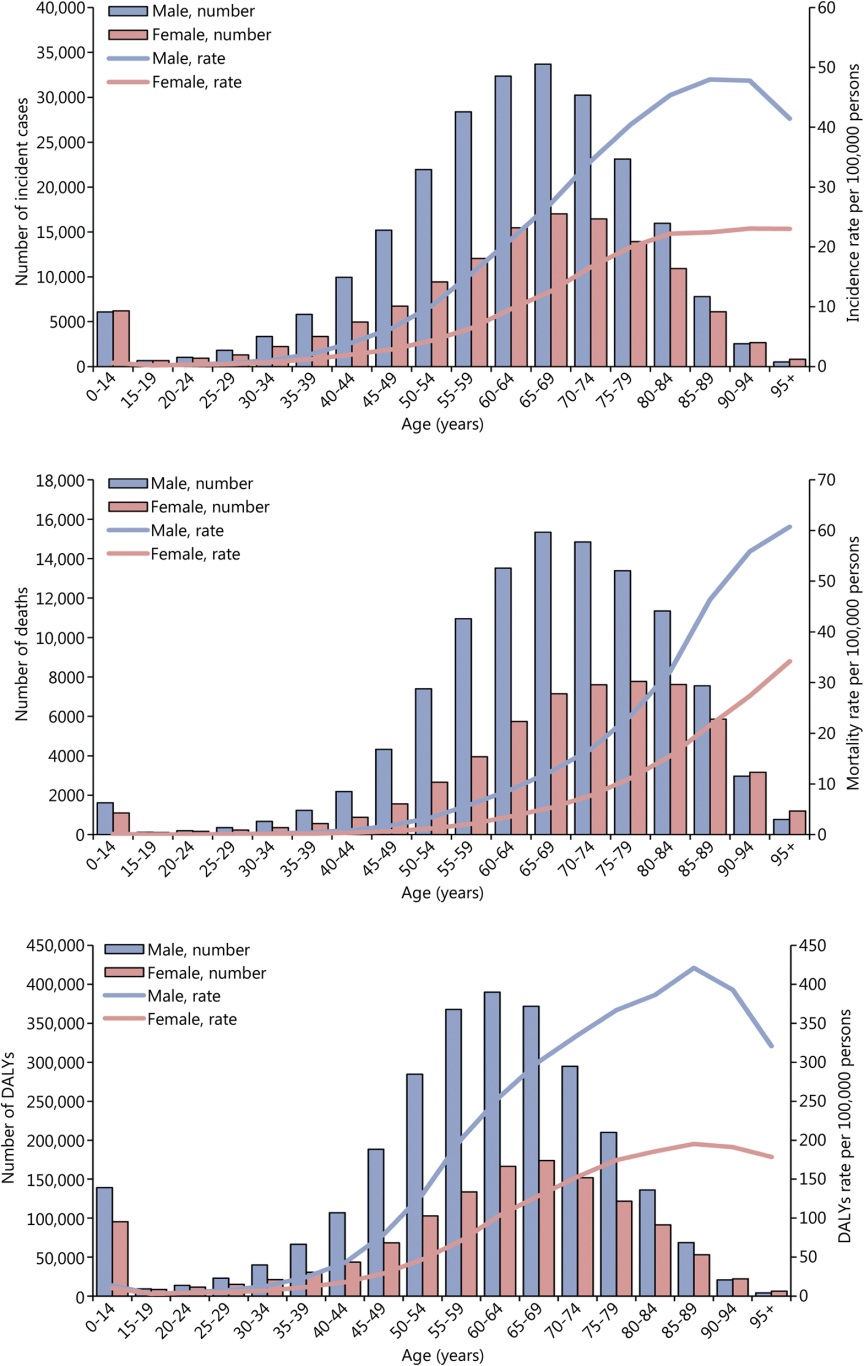
Global incidence, mortality, and DALYs of kidney cancer by age and sex in 2019. DALYs disability-adjusted life-years

The 65–69 years age group had the most incident cases of kidney cancer in 2019 (Fig. 3). The incidence rate of kidney cancer was highest in the 85–89 years age group in men (47.97, 95% UI 41.44–52.79) and 90–94 years age group in women (23.06, 95% UI 17.53–26.68). The highest mortality rate of kidney cancer was observed in the oldest age groups. The incident cases of bladder cancer increased with age, peaking at 70–74 years, and then decreased (Additional file 2: Fig. S16). In the 95 years or older age group, the incidence rate, mortality rate, and DALYs rate of bladder cancer in men were 3.4 times, 3.3 times, and 3.3 times higher than women, respectively. The incident cases of prostate cancer started increasing at 55–59 years, reached a peak at 70–74 years, and then decreased in the oldest age groups (Additional file 2: Fig. S17).

### Attributable risk factors

The percentage of kidney cancer DALYs that was attributable to the smoking decreased from 18.19% in 1990 to 16.95% in 2019; in contrast, the percentage of kidney cancer DALYs that was attributable to high BMI increased from 13.98% to 18.55% during the same period (Additional file 1: Table S9). Smoking and high FPG were main attributable risk factors of bladder cancer death and DALYs. In 2019, 6.01% of deaths and 6.60% of DALYs caused by prostate cancer were attributable to smoking.

## Discussion

This study demonstrated significant change in the global and regional burden of, and risk factors for genitourinary cancers in the past 30 years. Compared with 1990, the proportion of prostate cancer incident cases expanded and still accounted for the majority among these cancers. The proportion of bladder cancer decreased. The proportion of kidney cancer remained relatively stable. At the global level, the increase in ASIR is most prominent for kidney cancer (EAPC = 0.87), followed by prostate cancer (EAPC = 0.26) and bladder cancer (EAPC = 0.07). Kidney cancer accounted for the smallest percentage of genitourinary cancers, but the increase in ASIR is fastest among the three cancers. The observed change in global incidence of genitourinary cancers likely reflects the evolution of early detection methods, such as prostate-specific antigen (PSA) screening for prostate cancer and cross-sectional imaging for kidney cancer [13, 14]. The widespread use of routine PSA testing led to a rapid increase in the incidence of prostate cancer in the early 1990s in the USA; however, prostate cancer incidence has been declining in recent years with the reduction in routine  PSA testing [15–17]. Likewise, advances in imaging technology have led to an increase in the detection rate of kidney cancer in developed countries [13]. Aging population and lifestyle changes may have also contributed to increased incidence of genitourinary cancers. Deaths caused by genitourinary cancers increased, but ASMR decreased, especially for prostate and bladder cancers. Such a trend was also evident in DALYs and ASDR for all three genitourinary cancers, suggesting the effectiveness of current cancer prevention and treatment strategies.

The incidence, mortality, and DALYs varied significantly across different regions and countries. The incidence of bladder cancer showed a downward trend in 8 regions, whereas prostate cancer and kidney cancer showed an upward trend in 21 regions. These results were consistent with previous studies [2, 4, 18]. The prevalence of risk factors associated with genitourinary cancer and the spread of early detection could partially explain the incidence trend in these regions. For example, differences in dietary habits, tobacco use, and health care policy may affect the incidence and mortality of bladder cancer between Europe and Asia [19]. Mortality and DALYs of genitourinary cancers showed significant decrease in developed regions such as Australasia and High-income North America. Better health care policies and disease prevention awareness may play an important role in reducing the burden of genitourinary cancers. For instance, early detection of kidney cancer and prostate cancer, such as PSA testing, ultrasonography, and computed tomography, may provide more treatment opportunities, although the benefits of early detection are still debated.

High-level SDI regions and countries have higher incidence, mortality and DALYs for all three cancers. However, the mortality and DALYs of genitourinary cancers in high-level SDI regions and countries showed a downward trend, particularly for prostate cancer. Decreased incidence and mortality of prostate cancer in Europe and Northern America have been attributed to the changes of PSA screening guidelines and improvement of treatments [20]. Therefore, countries with different degrees of social development, especially underdeveloped and developing countries, need to formulate appropriate cancer prevention and intervention strategies based on local medical resources.

Consistent with previous studies [2–4, 21, 22], the burden of genitourinary cancers was highest in elderly men. The aging population clearly affects the burden of disease, but cancer-related risk factors also play an important role [23–27]. Smoking, a risk factor shared by all three genitourinary showed a decreasing contribution to cancer death and DALYs in the past 30 years, suggesting the past/current tobacco control strategies are effective. In contrast, the burden of disease associated with high FPG and high BMI increased during the same period, highlighting the need to strengthen/modify preventive measures and treatment regimens.

Significant advances have been made at the international level [28], including the Universal Health Coverage program led by the WHO. However, lack of multidisciplinary and international cooperation in the development of guidelines may explain the fact that disease burden decreased in developed countries but remain high in some low-resources countries. Our research also found the problem that the low-, low-middle, and middle SDI quintile had a steady or upward trend in ASIR, ASMR, and ASDR of genitourinary cancers. Limited access to medical services, low economic income, limited trained doctors and other reasons may limit the implementation of global standardized guidelines in developing or underdeveloped areas. As such, urologists and urological related academic associations could develop adapted tools to provide guidance for the implementation of cancer prevention, screening and treatment. The first Global Prostate Cancer Consensus Conference for Developing Countries and the Kidney Cancer Association has made new attempts to provide services for cancer patients, physicians, and healthcare professionals [29, 30].

Infectious diseases may change the model of clinical services, especially in cancer screening, diagnostics, and treatment [31]. A prospective cohort study that compared the epidemiology, clinical features and outcomes of influenza A (H1N1) 2009 pneumonia between the pandemic period and the first post-pandemic influenza season showed that patients during the post-pandemic period were older and more likely to have chronic obstructive pulmonary disease, chronic kidney disease and cancer than the others [32]. Another 8-year follow-up study found patients with malignancies had more severe influenza and presented worse outcomes after the H1N1 2009 pandemic [33]. The impact of the Corona Virus Disease 2019 (COVID-19) pandemic is unprecedented. A global survey showed COVID-19 had led to significant delays in outpatient care and surgery in urology, with an average delay of more than 8 weeks [34]. Delays in the normal medical order could also conceivably result in a large number of patients not being accounted for when morbidity and mortality data are collected [35].

The strength of this study is the most up-to-date epidemiological data of kidney, bladder, and prostate cancers in 204 countries and territories from 1990 to 2019. In addition, this study reveals the changes in the disease spectrum and disease burden of three common genitourinary cancers by geographic location, SDI, age and sex. Finally, this study indicates the changes of metabolic risks factors (high BMI and high FPG) and the behavioral risks factors (smoking) on disease burden of genitourinary cancers between 1990 and 2019.

This study has several limitations. First, the accuracy of the GBD data depends on the quality of the existing data in each country. Quality of the data for smaller countries or countries with low levels of development may not be as accurate, and thus should be interpreted with caution. Second, differences in reporting approaches and case definitions may have introduced to unknown biases. Third, risk factors for genitourinary cancers in GBD 2019 are limited and do not capture the full spectrum of disease etiologies.

## Conclusions

In conclusion, kidney, bladder, and prostate cancers remain major global public health challenges, but with distinct trend for different disease entity across different regions and socioeconomic status. More proactive intervention strategies, at both the administrative and academic levels, based on the dynamic changes, are needed.

## Supplementary Information

**Additional file 1.** **Table S1.** Socio-demographic index values for all estimated GBD 2019 locations, 1990–2019. **Table S2.** Five socio-demographic index quintiles in GBD 2019. **Table S3.** Regional incident cases and age-standardized incidence rate of genitourinary cancers in 2019. **Table S4.** Regional deaths and age-standardized mortality rate of genitourinary cancers in 2019. **Table S5.** Regional DALYs and age-standardized DALYs rate of genitourinary cancers in 2019. **Table S6.** Incidence, mortality and DALYs of genitourinary cancers among the top three and bottom three countries in 2019. **Table S7.** EAPC of ASMR for genitourinary cancers in 204 countries and territories from 1990 to 2019. **Table S8.** EAPC of ASDR for genitourinary cancers in 204 countries and territories from 1990 to 2019. **Table S9.** Percentage of genitourinary cancers deaths and DALYs attributable to risk factors in 1990 and 2019.

**Additional file 2.** **Fig. S1.** Percentage of incident cases, deaths, and DALYs for genitourinary cancers in 1990 and 2019. **a** Incident cases. **b** Deaths. **c** DALYs. DALYs disability-adjusted life-years. **Fig. S2.** Global ASIR, ASMR, and ASDR of genitourinary cancers from 1990 to 2019. **a** ASIR. **b** ASMR. **c** ASDR. ASIR age-standardized incidence rate, ASMR age-standardized mortality rate, ASDR age-standardized DALYs rate, DALYs disability-adjusted life-years. **Fig. S3.** EAPC of ASMR for genitourinary cancers in global and 21 regions. **a** Kidney cancer. **b** Bladder cancer. **c** Prostate cancer. ASMR age-standardized mortality rate, EAPC estimated annual percentage change. **Fig. S4.** EAPC of ASDR for genitourinary cancers in global and 21 regions. **a** Kidney cancer. **b** Bladder cancer. **c** Prostate cancer. ASDR age-standardized DALYs rate, EAPC estimated annual percentage change. **Fig. S5.** Burden of kidney cancer by SDI quintile from 1990 to 2019. DALYs disability-adjusted life-years, SDI sociodemographic index. **Fig. S6.** Burden of bladder cancer by SDI quintile from 1990 to 2019. DALYs disability-adjusted life-years, SDI sociodemographic index. **Fig. S7.** Burden of prostate cancer by SDI quintile from 1990 to 2019. DALYs disability-adjusted life-years, SDI sociodemographic index. **Fig. S8.** ASMR of kidney cancer for 21 regions and 204 countries and territories by SDI. **a** 21 regions by SDI from 1990 to 2019. **b** 204 countries and territories by SDI in 2019. ASMR age-standardized mortality rate, SDI sociodemographic index. **Fig. S9.** ASDR of kidney cancer for 21 regions and 204 countries and territories by SDI. **a** 21 regions by SDI from 1990 to 2019. **b** 204 countries and territories by SDI in 2019. ASDR age-standardized DALYs rate, SDI sociodemographic index. **Fig. S10.** ASIR of bladder cancer for 21 regions and 204 countries and territories by SDI. **a** 21 regions by SDI from 1990 to 2019. **b** 204 countries and territories by SDI in 2019. ASIR age-standardized incidence rate, SDI sociodemographic index. **Fig. S11.** ASIR of prostate cancer for 21 regions and 204 countries and territories by SDI. **a** 21 regions by SDI from 1990 to 2019. **b** 204 countries and territories by SDI in 2019. ASIR age-standardized incidence rate, SDI sociodemographic index. **Fig. S12.** ASMR of bladder cancer for 21 regions and 204 countries and territories by SDI. **a** 21 regions by SDI from 1990 to 2019. **b** 204 countries and territories by SDI in 2019. ASMR age-standardized mortality rate, SDI sociodemographic index. **Fig. S13.** ASDR of bladder cancer for 21 regions and 204 countries and territories by SDI. **a** 21 regions by SDI from 1990 to 2019. **b** 204 countries and territories by SDI in 2019. ASDR age-standardized DALYs rate, SDI sociodemographic index. **Fig. S14.** ASMR of prostate cancer for 21 regions and 204 countries and territories by SDI. **a** 21 regions by SDI from 1990 to 2019. **b** 204 countries and territories by SDI in 2019. ASMR age-standardized mortality rate, SDI sociodemographic index. **Fig. S15.** ASDR of prostate cancer for 21 regions and 204 countries and territories by SDI. **a** 21 regions by SDI from 1990 to 2019. **b** 204 countries and territories by SDI in 2019. ASDR age-standardized DALYs rate, SDI sociodemographic index. **Fig. S16.** Global incidence, mortality, and DALYs of bladder cancer by age and sex in 2019. DALYs disability-adjusted life-years. **Fig. S17.** Global incidence, mortality, and DALYs of prostate cancer by age and sex in 2019. DALYs disability-adjusted life-years.

## Data Availability

The datasets generated during the current study are available in the Global Health Data Exchange query tool (http://ghdx.healthdata.org/gbd-results-tool).

## Abbreviations

ASDR: Age-standardized DALYs rate
ASIR: Age-standardized incidence rate
ASMR: Age-standardized mortality rate
ASR: Age-standardized rates
BMI: Body mass index
CI: Confidence interval
CODEm: Cause of Death Ensemble model
COVID-19: Corona virus disease 2019
DALYs: Disability-adjusted life-years
EAPC: Estimated annual percentage changes
FPG: Fasting plasma glucose
GBD: Global Burden of Disease
ICD: International Classification of Diseases
PSA: Prostate-specific antigen
SDI: Socio-demographic index
UI: Uncertainty interval
