# Supplementary material for: Global, regional, and national burden of kidney, bladder, and prostate cancers and their attributable risk factors, 1990–2019
Source: Mil Med Res. 2021 Nov 24;8:60. doi: 10.1186/s40779-021-00354-z (PMC8611255; doi:10.1186/s40779-021-00354-z)
Supplement: Supplementary file 1 — Additional file 1. Table S1. Socio-demographic index values for all estimated GBD 2019 locations, 1990–2019. Table S2. Five socio-demographic index quintiles in GBD 2019. Table S3. Regional incident cases and age-standardized incidence rate of genitourinary cancers in 2019. Table S4. Regional deaths and age-standardized mortality rate of genitourinary cancers in 2019. Table S5. Regional DALYs and age-standardized DALYs rate of genitourinary cancers in 2019. Table S6. Incidence, mortality and DALYs of genitourinary cancers among the top three and bottom three countries in 2019. Table S7. EAPC of ASMR for genitourinary cancers in 204 countries and territories from 1990 to 2019. Table S8. EAPC of ASDR for genitourinary cancers in 204 countries and territories from 1990 to 2019. Table S9. Percentage of genitourinary cancers deaths and DALYs attributable to risk factors in 1990 and 2019. [file 40779_2021_354_MOESM1_ESM.pdf]

Table S1 Socio-demographic index values for all estimated GBD 2019 locations, 1990-201[illegible]

Location	1990	1991	1992	1993	1994	1995	1996	1997	1998	1999	2000	2001	2002	2003	2004	2005	2006	2007	2008	2009	2010	2011	2012	2013	2014	2015	2016	2017	2018	2019
Global	0·511	0·516	0·521	0·525	0·529	0·534	0·538	0·542	0·547	0·551	0·556	0·561	0·566	0·571	0·576	0·581	0·586	0·591	0·596	0·601	0·607	0·612	0·616	0·621	0·626	0·631	0·635	0·641	0·647	0·651
Central Europe, eastern Europe, and central Asia	0·648	0·654	0·662	0·666	0·669	0·672	0·675	0·678	0·681	0·684	0·687	0·69	0·694	0·698	0·704	0·71	0·714	0·719	0·724	0·728	0·732	0·735	0·738	0·742	0·745	0·748	0·751	0·754	0·758	0·76
Central Asia	0·551	0·555	0·557	0·558	0·559	0·559	0·56	0·56	0·561	0·563	0·566	0·569	0·574	0·579	0·585	0·591	0·598	0·605	0·611	0·617	0·622	0·627	0·632	0·637	0·642	0·647	0·651	0·655	0·659	0·663
Armenia	0·536	0·541	0·541	0·542	0·544	0·546	0·55	0·554	0·559	0·564	0·57	0·577	0·586	0·596	0·606	0·616	0·626	0·637	0·647	0·652	0·658	0·662	0·666	0·67	0·673	0·676	0·679	0·682	0·686	0·689
Azerbaijan	0·576	0·578	0·579	0·578	0·576	0·573	0·569	0·565	0·561	0·559	0·559	0·561	0·564	0·569	0·575	0·583	0·594	0·607	0·619	0·628	0·637	0·645	0·652	0·658	0·664	0·669	0·673	0·677	0·68	0·683
Georgia	0·654	0·658	0·657	0·654	0·65	0·644	0·638	0·633	0·628	0·625	0·624	0·626	0·63	0·636	0·641	0·646	0·651	0·656	0·66	0·663	0·665	0·668	0·672	0·676	0·68	0·684	0·688	0·693	0·697	0·702
Kazakhstan	0·602	0·606	0·611	0·615	0·619	0·622	0·625	0·628	0·63	0·632	0·635	0·639	0·644	0·649	0·655	0·661	0·667	0·674	0·679	0·683	0·688	0·692	0·696	0·7	0·704	0·708	0·712	0·716	0·72	0·723
Kyrgyzstan	0·532	0·537	0·541	0·543	0·542	0·541	0·539	0·537	0·536	0·534	0·534	0·535	0·537	0·54	0·544	0·546	0·549	0·552	0·555	0·558	0·56	0·563	0·565	0·569	0·574	0·578	0·583	0·588	0·592	0·596
Mongolia	0·465	0·47	0·475	0·48	0·484	0·49	0·495	0·501	0·506	0·512	0·517	0·523	0·528	0·534	0·539	0·545	0·55	0·555	0·56	0·563	0·566	0·57	0·575	0·579	0·584	0·588	0·592	0·597	0·601	0·606
Tajikistan	0·468	0·473	0·474	0·474	0·472	0·468	0·462	0·457	0·451	0·445	0·441	0·44	0·443	0·448	0·456	0·463	0·47	0·477	0·483	0·489	0·495	0·5	0·505	0·511	0·516	0·521	0·526	0·531	0·535	0·539
Turkmenistan	0·548	0·551	0·554	0·557	0·557	0·558	0·558	0·557	0·556	0·557	0·561	0·565	0·57	0·576	0·582	0·588	0·595	0·601	0·606	0·611	0·616	0·622	0·628	0·635	0·642	0·648	0·654	0·66	0·666	0·67
Uzbekistan	0·49	0·492	0·494	0·496	0·498	0·501	0·505	0·51	0·515	0·52	0·525	0·531	0·536	0·541	0·546	0·551	0·556	0·561	0·567	0·572	0·578	0·584	0·59	0·597	0·603	0·609	0·616	0·622	0·627	0·631
Central Europe	0·641	0·647	0·652	0·658	0·665	0·672	0·678	0·683	0·689	0·695	0·702	0·709	0·715	0·72	0·726	0·731	0·736	0·74	0·745	0·75	0·756	0·76	0·764	0·768	0·771	0·775	0·778	0·781	0·785	0·788
Albania	0·54	0·537	0·534	0·533	0·535	0·538	0·544	0·549	0·555	0·561	0·569	0·577	0·585	0·593	0·601	0·608	0·615	0·621	0·627	0·631	0·636	0·64	0·645	0·651	0·658	0·664	0·669	0·674	0·678	0·681
Bosnia and Herzegovina	0·533	0·534	0·532	0·529	0·527	0·528	0·54	0·558	0·576	0·591	0·604	0·616	0·626	0·636	0·644	0·651	0·658	0·665	0·671	0·677	0·682	0·686	0·691	0·695	0·698	0·702	0·706	0·71	0·714	0·718
Bulgaria	0·631	0·641	0·648	0·656	0·666	0·671	0·676	0·681	0·677	0·675	0·68	0·688	0·693	0·697	0·701	0·706	0·71	0·715	0·718	0·724	0·733	0·737	0·74	0·743	0·746	0·75	0·752	0·755	0·76	0·764
Croatia	0·68	0·688	0·692	0·692	0·692	0·691	0·691	0·697	0·703	0·707	0·713	0·719	0·725	0·73	0·734	0·739	0·745	0·748	0·753	0·758	0·763	0·767	0·77	0·774	0·777	0·781	0·784	0·788	0·791	0·794
Czech Republic	0·688	0·696	0·705	0·718	0·736	0·748	0·755	0·76	0·765	0·771	0·776	0·782	0·786	0·79	0·794	0·798	0·801	0·804	0·807	0·81	0·813	0·816	0·818	0·819	0·82	0·82	0·82	0·822	0·825	0·828
Hungary	0·659	0·663	0·671	0·678	0·685	0·693	0·7	0·707	0·713	0·718	0·724	0·73	0·735	0·741	0·746	0·751	0·756	0·76	0·763	0·768	0·772	0·773	0·774	0·774	0·775	0·778	0·781	0·784	0·788	0·791
Montenegro	0·701	0·701	0·699	0·695	0·69	0·687	0·686	0·687	0·69	0·692	0·696	0·701	0·706	0·712	0·717	0·723	0·729	0·736	0·743	0·749	0·754	0·759	0·764	0·768	0·773	0·777	0·78	0·784	0·788	0·791
North Macedonia	0·618	0·62	0·623	0·625	0·627	0·631	0·635	0·64	0·646	0·651	0·656	0·662	0·668	0·674	0·679	0·684	0·689	0·694	0·7	0·704	0·709	0·713	0·717	0·722	0·726	0·73	0·734	0·738	0·741	0·744
Poland	0·632	0·637	0·644	0·653	0·661	0·67	0·677	0·685	0·693	0·701	0·709	0·717	0·724	0·73	0·735	0·74	0·743	0·747	0·752	0·757	0·763	0·77	0·775	0·78	0·784	0·788	0·791	0·795	0·798	0·802
Romania	0·625	0·632	0·635	0·638	0·643	0·649	0·653	0·655	0·659	0·664	0·669	0·677	0·682	0·686	0·693	0·698	0·702	0·707	0·711	0·718	0·726	0·729	0·734	0·74	0·741	0·744	0·747	0·752	0·756	0·76
Serbia	0·626	0·635	0·639	0·639	0·64	0·64	0·644	0·647	0·651	0·657	0·661	0·665	0·67	0·676	0·685	0·694	0·702	0·709	0·716	0·723	0·729	0·735	0·739	0·744	0·748	0·753	0·756	0·76	0·763	0·767
Slovakia	0·656	0·662	0·668	0·679	0·693	0·702	0·709	0·716	0·724	0·731	0·739	0·746	0·752	0·756	0·76	0·766	0·772	0·777	0·781	0·784	0·789	0·794	0·798	0·801	0·803	0·803	0·804	0·805	0·808	0·812
Slovenia	0·726	0·731	0·736	0·741	0·746	0·751	0·756	0·762	0·768	0·774	0·78	0·787	0·793	0·797	0·802	0·807	0·811	0·814	0·818	0·82	0·822	0·824	0·825	0·827	0·829	0·831	0·833	0·835	0·838	0·84
Eastern Europe	0·68	0·687	0·697	0·702	0·702	0·705	0·707	0·708	0·709	0·711	0·711	0·713	0·716	0·72	0·727	0·734	0·74	0·745	0·751	0·757	0·762	0·765	0·768	0·772	0·777	0·781	0·785	0·788	0·791	0·793
Belarus	0·591	0·595	0·6	0·606	0·611	0·614	0·618	0·62	0·621	0·624	0·629	0·635	0·642	0·65	0·658	0·665	0·671	0·678	0·687	0·695	0·703	0·709	0·713	0·719	0·725	0·73	0·734	0·738	0·742	0·745
Estonia	0·665	0·676	0·687	0·696	0·7	0·705	0·711	0·717	0·721	0·726	0·733	0·741	0·748	0·753	0·759	0·765	0·771	0·777	0·785	0·792	0·798	0·804	0·809	0·813	0·817	0·821	0·825	0·829	0·833	0·835
Latvia	0·675	0·682	0·691	0·7	0·708	0·713	0·716	0·719	0·721	0·723	0·727	0·733	0·739	0·745	0·753	0·76	0·766	0·774	0·784	0·793	0·797	0·798	0·801	0·803	0·804	0·805	0·809	0·813	0·817	0·82
Lithuania	0·67	0·672	0·682	0·691	0·694	0·696	0·7	0·705	0·709	0·714	0·723	0·73	0·736	0·743	0·752	0·76	0·765	0·771	0·782	0·792	0·797	0·801	0·808	0·813	0·817	0·822	0·829	0·835	0·839	0·843
Moldova	0·585	0·589	0·591	0·594	0·594	0·595	0·594	0·593	0·591	0·587	0·585	0·585	0·588	0·593	0·6	0·607	0·615	0·622	0·63	0·637	0·644	0·651	0·658	0·665	0·672	0·677	0·683	0·688	0·693	0·696
Russia	0·695	0·703	0·716	0·72	0·719	0·722	0·724	0·725	0·726	0·728	0·728	0·728	0·73	0·734	0·741	0·749	0·754	0·759	0·764	0·77	0·775	0·777	0·779	0·784	0·788	0·793	0·797	0·801	0·803	0·805
Ukraine	0·653	0·657	0·661	0·665	0·666	0·667	0·667	0·667	0·666	0·665	0·664	0·665	0·668	0·672	0·679	0·685	0·692	0·699	0·706	0·71	0·713	0·718	0·721	0·725	0·727	0·729	0·73	0·732	0·734	0·736
High income	0·755	0·76	0·765	0·769	0·773	0·777	0·78	0·783	0·786	0·788	0·791	0·795	0·799	0·801	0·804	0·806	0·807	0·809	0·812	0·816	0·82	0·823	0·826	0·829	0·832	0·835	0·839	0·842	0·845	0·847
Australasia	0·742	0·746	0·749	0·753	0·757	0·761	0·765	0·769	0·773	0·777	0·781	0·785	0·789	0·793	0·797	0·799	0·799	0·8	0·803	0·807	0·81	0·812	0·816	0·821	0·825	0·828	0·832	0·835	0·837	0·84
Australia	0·738	0·741	0·745	0·749	0·753	0·757	0·761	0·766	0·77	0·774	0·778	0·782	0·787	0·791	0·795	0·797	0·798	0·799	0·802	0·806	0·809	0·812	0·815	0·82	0·824	0·828	0·832	0·834	0·837	0·839
New Zealand	0·757	0·762	0·765	0·769	0·772	0·774	0·778	0·782	0·785	0·787	0·79	0·794	0·796	0·798	0·802	0·803	0·8	0·8	0·803	0·807	0·809	0·812	0·816	0·821	0·825	0·828	0·832	0·835	0·838	0·84
High-income Asia Pacific	0·767	0·773	0·779	0·785	0·79	0·796	0·801	0·805	0·809	0·813	0·816	0·819	0·823	0·826	0·83	0·833	0·836	0·839	0·842	0·844	0·847	0·85	0·853	0·856	0·859	0·862	0·865	0·868	0·871	0·873
Brunei	0·676	0·682	0·688	0·694	0·7	0·706	0·712	0·717	0·723	0·729	0·735	0·741	0·747	0·753	0·758	0·764	0·769	0·774	0·779	0·784	0·789	0·793	0·797	0·801	0·806	0·809	0·813	0·817	0·82	0·823
Japan	0·791	0·796	0·801	0·805	0·809	0·813	0·817	0·82	0·822	0·824	0·826	0·828	0·83	0·833	0·836	0·838	0·84	0·842	0·844	0·846	0·848	0·85	0·853	0·855	0·857	0·86	0·862	0·865	0·867	0·87
Aichi	0·801	0·806	0·811	0·816	0·82	0·825	0·828	0·831	0·833	0·835	0·837	0·838	0·841	0·843	0·846	0·848	0·85	0·852	0·854	0·856	0·859	0·861	0·864	0·866	0·869	0·871	0·874	0·876	0·879	0·881
Akita	0·746	0·75	0·754	0·759	0·763	0·768	0·772	0·776	0·779	0·781	0·784	0·786	0·789	0·791	0·794	0·796	0·798	0·8	0·802	0·803	0·806	0·808	0·811	0·813	0·816	0·819	0·822	0·824	0·827	0·83
Aomori	0·736	0·74	0·745	0·749	0·754	0·758	0·762	0·766	0·768	0·771	0·773	0·775	0·779	0·782	0·786	0·789	0·791	0·794	0·796	0·798	0·8	0·803	0·806	0·809	0·811	0·814	0·817	0·82	0·823	0·826
Chiba	0·79	0·794	0·8	0·804	0·808	0·813	0·817	0·82	0·822	0·824	0·825	0·826	0·827	0·829	0·832	0·834	0·835	0·837	0·838	0·84	0·842	0·844	0·846	0·848	0·85	0·852	0·855	0·857	0·859	0·861
Ehime	0·757	0·761	0·766	0·771	0·775	0·78	0·784	0·788	0·791	0·795	0·797	0·8	0·802	0·805	0·807	0·809	0·811	0·812	0·814	0·816	0·818	0·82	0·822	0·824	0·827	0·83	0·833	0·836	0·838	0·841
Fukui	0·767	0·772	0·778	0·782	0·787	0·791	0·795	0·799	0·802	0·806	0·808	0·811	0·813	0·816	0·818	0·821	0·823	0·826	0·828	0·831	0·833	0·836	0·838	0·84	0·842	0·844	0·846	0·849	0·851	0·854
Fukuoka	0·783	0·786	0·791	0·795	0·799	0·803	0·807	0·81	0·813	0·815	0·818	0·82	0·822	0·825	0·828	0·829	0·831	0·832	0·833	0·835	0·837	0·84	0·842	0·844	0·846	0·848	0·85	0·853	0·855	0·858
Fukushima	0·751	0·755	0·76	0·764	0·768	0·773	0·776	0·779	0·781	0·784	0·785	0·787	0·79	0·794	0·797	0·8	0·803	0·805	0·807	0·809	0·812	0·814	0·816	0·818	0·821	0·823	0·826	0·83	0·833	0·836
Gifu	0·771	0·776	0·781	0·786	0·79	0·795	0·798	0·802	0·804	0·806	0·808	0·81	0·813	0·815	0·818	0·821	0·823	0·825	0·826	0·828	0·831	0·833	0·836	0·838	0·84	0·842	0·845	0·847	0·849	0·852
Gunma	0·774	0·778	0·783	0·788	0·792	0·796	0·8	0·803	0·805	0·807	0·808	0·81	0·813	0·816	0·819	0·821	0·823	0·825	0·828	0·83	0·833	0·836	0·838	0·841	0·844	0·847	0·85	0·852	0·855	0·858
Hiroshima	0·788	0·793	0·798	0·802	0·807	0·811	0·815	0·818	0·821	0·823	0·825	0·827	0·829	0·832	0·834	0·836	0·837	0·839	0·84	0·841	0·843	0·846	0·849	0·851	0·854	0·857	0·859	0·862	0·865	0·868
Hokkaidō	0·768	0·772	0·777	0·782	0·786	0·791	0·794	0·797	0·799	0·802	0·803	0·805	0·808	0·81	0·813	0·815	0·817	0·818	0·82	0·821	0·823	0·825	0·827	0·829	0·831	0·833	0·836	0·838	0·841	0·843
Hyōgo	0·794	0·798	0·804	0·808	0·812	0·817	0·82	0·823	0·826	0·828	0·83	0·832	0·833	0·835	0·837	0·838	0·839	0·841	0·842	0·844	0·845	0·848	0·85	0·852	0·854	0·857	0·859	0·862	0·864	0·866
Ibaraki	0·773	0·777	0·782	0·787	0·791	0·796	0·8	0·803	0·805	0·807	0·808	0·81	0·812	0·815	0·818	0·821	0·823	0·826	0·828	0·831	0·834	0·837	0·839	0·842	0·844	0·847	0·85	0·852	0·855	0·858
Ishikawa	0·774	0·78	0·785	0·79	0·795	0·799	0·803	0·807	0·81	0·813	0·816	0·818	0·821	0·824	0·826	0·829	0·83	0·832	0·833	0·834	0·835	0·837	0·839	0·841	0·843	0·846	0·848	0·851	0·854	0·856
Iwate	0·738	0·742	0·747	0·752	0·757	0·762	0·766	0·769	0·772	0·775	0·777	0·779	0·782	0·785	0·789	0·791	0·793	0·796	0·798	0·8	0·803	0·806	0·808	0·811	0·814	0·818	0·821	0·824	0·828	0·831
Kagawa	0·772	0·777	0·783	0·788	0·792	0·796	0·8	0·803	0·805	0·807	0·809	0·812	0·814	0·817	0·82	0·823	0·824	0·826	0·827	0·829	0·831	0·834	0·837	0·839	0·842	0·844	0·847	0·85	0·853	0·855
Kagoshima	0·744	0·748	0·753	0·757	0·761	0·766	0·77	0·773	0·776	0·779	0·781	0·784	0·787	0·79	0·793	0·795	0·797	0·799	0·801	0·803	0·806	0·808	0·811	0·813	0·816	0·819	0·822	0·825	0·828	0·831
Kanagawa	0·82	0·824	0·828	0·832	0·836	0·84	0·843	0·846	0·848	0·849	0·85	0·851	0·853	0·855	0·857	0·859	0·86	0·862	0·864	0·865	0·867	0·869	0·87	0·872	0·873	0·875	0·877	0·878	0·88	0·882
Kōchi	0·744	0·748	0·752	0·757	0·761	0·766	0·77	0·774	0·777	0·781	0·784	0·787	0·79	0·793	0·796	0·798	0·799	0·801	0·803	0·805	0·807	0·81	0·812	0·815	0·817	0·82	0·823	0·826	0·829	0·832
Kumamoto	0·754	0·758	0·763	0·767	0·771	0·775	0·778	0·78	0·782	0·784	0·786	0·788	0·791	0·794	0·797	0·8	0·801	0·803	0·804	0·806	0·808	0·811	0·813	0·816	0·819	0·822	0·825	0·828	0·831	0·834
Kyōto	0·801	0·805	0·81	0·814	0·818	0·822	0·825	0·828	0·831	0·833	0·836	0·838	0·84	0·842	0·845	0·847	0·849	0·85	0·852	0·854	0·855	0·857	0·859	0·861	0·863	0·865	0·868	0·87	0·872	0·875
Mie	0·765	0·77	0·776	0·781	0·785	0·79	0·794	0·797	0·8	0·802	0·805	0·808	0·811	0·814	0·818	0·821	0·823	0·825	0·828	0·83	0·832	0·835	0·838	0·841	0·844	0·847	0·85	0·853	0·856	0·859
Miyagi	0·774	0·779	0·784	0·789	0·793	0·798	0·801	0·803	0·805	0·807	0·808	0·81	0·813	0·816	0·819	0·822	0·823	0·826	0·827	0·829	0·831	0·834	0·837	0·839	0·842	0·845	0·848	0·851	0·854	0·857
Miyazaki	0·741	0·744	0·749	0·752	0·755	0·759	0·763	0·767	0·771	0·774	0·777	0·779	0·782	0·785	0·788	0·791	0·792	0·794	0·795	0·797	0·799	0·802	0·805	0·807	0·81	0·814	0·817	0·82	0·823	0·826
Nagano	0·778	0·782	0·787	0·791	0·795	0·799	0·803	0·806	0·808	0·811	0·813	0·815	0·817	0·82	0·822	0·825	0·827	0·829	0·831	0·833	0·835	0·837	0·839	0·841	0·843	0·845	0·848	0·851	0·853	0·856
Nagasaki	0·743	0·747	0·752	0·756	0·761	0·765	0·769	0·772	0·775	0·778	0·78	0·783	0·785	0·787	0·79	0·792	0·794	0·796	0·797	0·799	0·801	0·804	0·807	0·81	0·813	0·815	0·818	0·821	0·824	0·827
Nara	0·779	0·784	0·789	0·794	0·799	0·803	0·807	0·81	0·812	0·815	0·817	0·819	0·822	0·824	0·826	0·827	0·829	0·83	0·831	0·833	0·834	0·836	0·837	0·839	0·841	0·843	0·845	0·847	0·849	0·851
Niigata	0·758	0·762	0·767	0·772	0·776	0·78	0·784	0·788	0·791	0·794	0·796	0·799	0·803	0·805	0·808	0·811	0·813	0·815	0·817	0·819	0·821	0·823	0·826	0·829	0·832	0·834	0·837	0·84	0·842	0·845
Ōita	0·765	0·769	0·774	0·779	0·783	0·787	0·791	0·795	0·798	0·8	0·803	0·805	0·808	0·811	0·814	0·817	0·818	0·82	0·821	0·823	0·825	0·827	0·83	0·832	0·834	0·836	0·839	0·841	0·844	0·847
Okayama	0·778	0·783	0·788	0·792	0·796	0·8	0·804	0·807	0·809	0·812	0·814	0·816	0·819	0·822	0·825	0·827	0·829	0·831	0·833	0·836	0·838	0·841	0·843	0·845	0·848	0·85	0·853	0·855	0·858	0·861
Okinawa	0·736	0·74	0·745	0·749	0·753	0·757	0·76	0·763	0·764	0·766	0·768	0·77	0·774	0·778	0·782	0·785	0·787	0·789	0·791	0·793	0·795	0·798	0·8	0·802	0·804	0·807	0·81	0·813	0·816	0·819
Ōsaka	0·806	0·81	0·816	0·82	0·824	0·828	0·832	0·834	0·837	0·839	0·841	0·842	0·844	0·846	0·848	0·849	0·85	0·852	0·853	0·854	0·856	0·858	0·86	0·862	0·864	0·866	0·869	0·871	0·873	0·875
Saga	0·752	0·756	0·761	0·765	0·77	0·774	0·778	0·781	0·783	0·786	0·788	0·79	0·794	0·797	0·8	0·802	0·804	0·806	0·808	0·81	0·812	0·814	0·816	0·818	0·82	0·822	0·825	0·828	0·831	0·834
Saitama	0·783	0·788	0·793	0·798	0·802	0·806	0·81	0·813	0·816	0·817	0·818	0·819	0·821	0·823	0·826	0·828	0·829	0·831	0·833	0·835	0·837	0·839	0·841	0·843	0·845	0·847	0·849	0·851	0·854	0·856
Shiga	0·794	0·799	0·805	0·81	0·814	0·819	0·823	0·826	0·829	0·831	0·833	0·835	0·837	0·84	0·843	0·845	0·847	0·849	0·85	0·852	0·853	0·855	0·858	0·86	0·862	0·864	0·867	0·869	0·872	0·874
Shimane	0·74	0·745	0·75	0·754	0·759	0·764	0·768	0·773	0·776	0·78	0·783	0·785	0·788	0·792	0·795	0·798	0·801	0·803	0·805	0·807	0·809	0·812	0·815	0·818	0·821	0·824	0·827	0·83	0·833	0·836
Shizuoka	0·781	0·785	0·79	0·794	0·799	0·803	0·807	0·81	0·812	0·814	0·816	0·817	0·82	0·823	0·827	0·83	0·831	0·834	0·836	0·838	0·84	0·843	0·845	0·848	0·851	0·853	0·856	0·858	0·861	0·864
Tochigi	0·773	0·778	0·783	0·788	0·793	0·797	0·8	0·803	0·805	0·807	0·809	0·811	0·813	0·816	0·819	0·822	0·824	0·827	0·829	0·831	0·834	0·836	0·839	0·841	0·844	0·847	0·851	0·854	0·857	0·859
Tokushima	0·755	0·76	0·766	0·771	0·775	0·779	0·783	0·787	0·79	0·794	0·797	0·8	0·804	0·808	0·812	0·816	0·818	0·821	0·823	0·825	0·828	0·831	0·833	0·836	0·839	0·842	0·845	0·848	0·851	0·854
Tōkyō	0·867	0·872	0·877	0·881	0·884	0·888	0·891	0·894	0·896	0·897	0·898	0·899	0·9	0·902	0·904	0·906	0·907	0·908	0·91	0·912	0·913	0·915	0·917	0·918	0·92	0·921	0·923	0·924	0·925	0·927
Tottori	0·758	0·762	0·767	0·772	0·776	0·781	0·785	0·788	0·791	0·794	0·796	0·798	0·8	0·802	0·805	0·807	0·808	0·81	0·812	0·813	0·815	0·816	0·818	0·82	0·822	0·825	0·827	0·83	0·833	0·835
Toyama	0·778	0·783	0·789	0·793	0·797	0·801	0·805	0·808	0·811	0·814	0·817	0·819	0·822	0·825	0·829	0·831	0·833	0·836	0·838	0·839	0·842	0·844	0·846	0·848	0·85	0·853	0·855	0·858	0·861	0·863
Wakayama	0·755	0·759	0·764	0·769	0·772	0·777	0·78	0·783	0·786	0·789	0·792	0·794	0·798	0·801	0·804	0·807	0·81	0·812	0·814	0·816	0·819	0·822	0·825	0·828	0·832	0·835	0·838	0·842	0·845	0·848
Yamagata	0·748	0·752	0·757	0·761	0·765	0·769	0·772	0·775	0·778	0·78	0·782	0·784	0·787	0·79	0·794	0·797	0·799	0·802	0·805	0·807	0·81	0·813	0·816	0·818	0·821	0·824	0·827	0·83	0·833	0·835
Yamaguchi	0·771	0·775	0·78	0·783	0·787	0·791	0·795	0·798	0·801	0·804	0·806	0·808	0·811	0·814	0·817	0·82	0·821	0·824	0·825	0·828	0·83	0·833	0·836	0·839	0·841	0·844	0·847	0·849	0·852	0·855
Yamanashi	0·777	0·782	0·787	0·791	0·795	0·799	0·803	0·806	0·808	0·81	0·812	0·814	0·816	0·818	0·821	0·824	0·827	0·83	0·832	0·834	0·836	0·839	0·841	0·843	0·846	0·848	0·851	0·853	0·856	0·858
South Korea	0·686	0·697	0·708	0·719	0·73	0·74	0·75	0·76	0·767	0·776	0·784	0·791	0·799	0·805	0·811	0·817	0·823	0·828	0·833	0·837	0·842	0·846	0·851	0·855	0·859	0·863	0·867	0·871	0·875	0·878
Singapore	0·688	0·697	0·705	0·714	0·723	0·731	0·74	0·749	0·756	0·762	0·769	0·776	0·783	0·789	0·794	0·801	0·808	0·814	0·822	0·828	0·835	0·839	0·843	0·847	0·85	0·852	0·855	0·858	0·86	0·861
High-income North America	0·771	0·773	0·777	0·78	0·784	0·787	0·79	0·792	0·794	0·797	0·8	0·805	0·809	0·811	0·814	0·815	0·814	0·817	0·822	0·828	0·834	0·837	0·841	0·844	0·847	0·85	0·854	0·857	0·859	0·86
Canada	0·79	0·792	0·795	0·797	0·8	0·804	0·809	0·812	0·815	0·819	0·824	0·828	0·832	0·835	0·838	0·84	0·842	0·843	0·845	0·848	0·851	0·853	0·856	0·859	0·861	0·864	0·867	0·869	0·871	0·873
Greenland	0·655	0·652	0·651	0·651	0·653	0·653	0·653	0·655	0·661	0·664	0·667	0·671	0·678	0·684	0·689	0·696	0·704	0·71	0·715	0·721	0·728	0·734	0·737	0·74	0·743	0·747	0·751	0·756	0·759	0·761
USA	0·768	0·771	0·775	0·778	0·782	0·785	0·788	0·789	0·791	0·794	0·797	0·802	0·806	0·809	0·811	0·812	0·811	0·814	0·819	0·826	0·832	0·835	0·839	0·842	0·845	0·849	0·853	0·856	0·858	0·859
Alabama	0·732	0·736	0·741	0·745	0·749	0·751	0·753	0·753	0·754	0·757	0·761	0·767	0·772	0·776	0·779	0·779	0·777	0·78	0·786	0·794	0·801	0·805	0·809	0·811	0·812	0·814	0·816	0·818	0·819	0·819
Alaska	0·748	0·75	0·754	0·76	0·766	0·771	0·776	0·779	0·782	0·784	0·787	0·793	0·796	0·799	0·801	0·801	0·798	0·8	0·804	0·811	0·817	0·821	0·826	0·83	0·834	0·839	0·844	0·848	0·85	0·851
Arizona	0·739	0·742	0·745	0·747	0·75	0·752	0·754	0·755	0·757	0·758	0·761	0·766	0·769	0·771	0·774	0·775	0·775	0·782	0·791	0·801	0·81	0·814	0·818	0·822	0·825	0·829	0·834	0·838	0·84	0·842
Arkansas	0·711	0·716	0·723	0·727	0·731	0·734	0·735	0·736	0·737	0·739	0·742	0·748	0·752	0·755	0·758	0·757	0·756	0·76	0·766	0·774	0·783	0·787	0·79	0·794	0·796	0·799	0·803	0·807	0·809	0·811
California	0·757	0·757	0·76	0·762	0·767	0·772	0·778	0·783	0·788	0·793	0·798	0·804	0·809	0·811	0·814	0·815	0·816	0·82	0·825	0·831	0·838	0·841	0·845	0·848	0·852	0·856	0·861	0·865	0·868	0·87
Colorado	0·788	0·791	0·794	0·798	0·801	0·803	0·805	0·806	0·807	0·808	0·81	0·815	0·818	0·821	0·824	0·825	0·825	0·829	0·834	0·84	0·847	0·851	0·855	0·859	0·863	0·867	0·871	0·874	0·876	0·877
Connecticut	0·829	0·832	0·835	0·838	0·841	0·843	0·845	0·847	0·849	0·852	0·855	0·86	0·864	0·865	0·867	0·868	0·868	0·87	0·873	0·877	0·881	0·884	0·887	0·89	0·893	0·896	0·899	0·901	0·902	0·902
Delaware	0·787	0·791	0·796	0·798	0·801	0·803	0·805	0·805	0·806	0·808	0·81	0·815	0·818	0·82	0·822	0·822	0·822	0·826	0·831	0·838	0·843	0·847	0·85	0·852	0·854	0·857	0·859	0·861	0·862	0·863
Washington, DC	0·784	0·788	0·793	0·798	0·805	0·812	0·82	0·826	0·833	0·839	0·846	0·853	0·859	0·862	0·865	0·865	0·865	0·867	0·869	0·872	0·876	0·877	0·879	0·88	0·881	0·883	0·884	0·886	0·886	0·886
Florida	0·759	0·763	0·768	0·773	0·777	0·78	0·783	0·784	0·786	0·789	0·792	0·798	0·802	0·804	0·807	0·807	0·807	0·812	0·818	0·826	0·832	0·836	0·839	0·842	0·844	0·846	0·85	0·852	0·854	0·856
Georgia	0·742	0·747	0·752	0·757	0·761	0·764	0·767	0·768	0·768	0·769	0·772	0·777	0·78	0·783	0·785	0·785	0·784	0·789	0·796	0·804	0·812	0·817	0·821	0·825	0·828	0·832	0·835	0·838	0·84	0·841
Hawaii	0·769	0·771	0·775	0·78	0·785	0·791	0·796	0·799	0·802	0·804	0·806	0·811	0·814	0·817	0·82	0·819	0·818	0·821	0·825	0·83	0·836	0·84	0·843	0·847	0·85	0·853	0·856	0·858	0·859	0·86
Idaho	0·744	0·748	0·753	0·758	0·762	0·765	0·767	0·768	0·769	0·77	0·772	0·777	0·78	0·781	0·784	0·783	0·782	0·786	0·792	0·8	0·807	0·81	0·813	0·815	0·817	0·82	0·823	0·825	0·827	0·827
Illinois	0·775	0·777	0·781	0·784	0·788	0·792	0·796	0·799	0·802	0·805	0·809	0·815	0·819	0·823	0·826	0·828	0·828	0·832	0·837	0·842	0·848	0·851	0·854	0·858	0·86	0·863	0·867	0·869	0·871	0·872
Indiana	0·758	0·761	0·766	0·769	0·772	0·774	0·775	0·776	0·777	0·779	0·782	0·787	0·791	0·793	0·795	0·794	0·792	0·795	0·8	0·806	0·812	0·816	0·819	0·823	0·826	0·829	0·833	0·835	0·837	0·838
Iowa	0·781	0·784	0·788	0·791	0·794	0·796	0·798	0·8	0·802	0·803	0·806	0·81	0·813	0·815	0·817	0·816	0·816	0·818	0·822	0·828	0·833	0·836	0·84	0·843	0·847	0·851	0·856	0·859	0·862	0·864
Kansas	0·771	0·774	0·778	0·781	0·784	0·786	0·788	0·789	0·79	0·792	0·794	0·799	0·803	0·805	0·807	0·806	0·804	0·806	0·81	0·815	0·822	0·826	0·83	0·835	0·84	0·845	0·85	0·854	0·857	0·858
Kentucky	0·729	0·733	0·738	0·743	0·747	0·749	0·752	0·753	0·754	0·756	0·76	0·766	0·77	0·773	0·776	0·775	0·772	0·774	0·778	0·784	0·79	0·794	0·797	0·8	0·803	0·806	0·81	0·812	0·814	0·815
Louisiana	0·725	0·728	0·733	0·738	0·742	0·745	0·747	0·748	0·75	0·752	0·755	0·762	0·768	0·771	0·775	0·775	0·773	0·776	0·781	0·788	0·794	0·798	0·801	0·805	0·808	0·812	0·817	0·82	0·822	0·823
Maine	0·774	0·78	0·787	0·792	0·796	0·8	0·802	0·803	0·805	0·807	0·81	0·814	0·818	0·82	0·823	0·824	0·824	0·827	0·831	0·835	0·84	0·842	0·845	0·848	0·85	0·853	0·856	0·858	0·86	0·862
Maryland	0·801	0·805	0·809	0·813	0·817	0·82	0·823	0·824	0·826	0·828	0·831	0·836	0·84	0·844	0·847	0·848	0·849	0·852	0·856	0·861	0·867	0·87	0·873	0·876	0·878	0·881	0·884	0·885	0·886	0·887
Massachusetts	0·83	0·834	0·837	0·84	0·843	0·846	0·849	0·852	0·854	0·858	0·861	0·866	0·869	0·872	0·874	0·876	0·877	0·879	0·882	0·886	0·889	0·892	0·894	0·897	0·899	0·902	0·904	0·906	0·907	0·907
Michigan	0·775	0·778	0·783	0·787	0·792	0·795	0·798	0·8	0·802	0·805	0·808	0·814	0·817	0·82	0·823	0·823	0·822	0·825	0·828	0·832	0·836	0·838	0·84	0·843	0·845	0·849	0·854	0·858	0·861	0·863
Minnesota	0·804	0·808	0·812	0·815	0·818	0·82	0·822	0·824	0·826	0·828	0·83	0·834	0·837	0·84	0·842	0·843	0·843	0·846	0·85	0·855	0·86	0·863	0·866	0·87	0·873	0·876	0·879	0·882	0·884	0·886
Mississippi	0·708	0·712	0·717	0·721	0·725	0·729	0·731	0·732	0·733	0·735	0·738	0·743	0·747	0·748	0·75	0·748	0·746	0·749	0·756	0·765	0·774	0·779	0·784	0·789	0·792	0·796	0·8	0·803	0·804	0·805
Missouri	0·758	0·761	0·766	0·77	0·774	0·777	0·779	0·78	0·782	0·784	0·787	0·792	0·796	0·797	0·799	0·799	0·797	0·8	0·805	0·811	0·818	0·821	0·825	0·828	0·831	0·834	0·838	0·841	0·843	0·844
Montana	0·764	0·767	0·773	0·778	0·783	0·787	0·79	0·792	0·793	0·794	0·796	0·8	0·803	0·805	0·808	0·808	0·807	0·809	0·813	0·818	0·823	0·826	0·829	0·833	0·837	0·842	0·847	0·851	0·854	0·856
Nebraska	0·781	0·785	0·789	0·793	0·796	0·799	0·801	0·802	0·803	0·804	0·806	0·809	0·811	0·813	0·816	0·816	0·815	0·817	0·821	0·827	0·833	0·836	0·84	0·845	0·849	0·853	0·857	0·86	0·862	0·862
Nevada	0·749	0·75	0·752	0·755	0·758	0·762	0·765	0·768	0·771	0·775	0·779	0·785	0·789	0·791	0·793	0·793	0·793	0·799	0·806	0·814	0·822	0·825	0·827	0·829	0·829	0·83	0·832	0·834	0·834	0·835
New Hampshire	0·813	0·818	0·823	0·826	0·828	0·83	0·832	0·834	0·837	0·841	0·845	0·849	0·853	0·856	0·859	0·86	0·86	0·863	0·866	0·87	0·874	0·877	0·88	0·883	0·886	0·89	0·893	0·895	0·897	0·898
New Jersey	0·816	0·819	0·823	0·826	0·83	0·833	0·835	0·837	0·838	0·84	0·843	0·847	0·85	0·853	0·856	0·857	0·858	0·86	0·863	0·868	0·872	0·875	0·878	0·88	0·883	0·886	0·888	0·89	0·892	0·892
New Mexico	0·721	0·724	0·729	0·733	0·739	0·743	0·747	0·748	0·75	0·751	0·753	0·759	0·762	0·765	0·767	0·767	0·765	0·769	0·775	0·784	0·793	0·798	0·803	0·807	0·811	0·815	0·819	0·823	0·825	0·826
New York	0·802	0·805	0·808	0·811	0·815	0·818	0·821	0·823	0·826	0·828	0·832	0·837	0·84	0·843	0·846	0·847	0·847	0·85	0·854	0·858	0·863	0·866	0·869	0·872	0·875	0·878	0·881	0·883	0·884	0·884
North Carolina	0·747	0·752	0·757	0·761	0·764	0·767	0·768	0·768	0·769	0·771	0·774	0·78	0·784	0·787	0·79	0·79	0·789	0·792	0·798	0·806	0·813	0·817	0·822	0·826	0·829	0·832	0·835	0·836	0·837	0·838
North Dakota	0·778	0·782	0·785	0·788	0·791	0·795	0·798	0·802	0·806	0·809	0·813	0·816	0·818	0·819	0·82	0·819	0·818	0·82	0·823	0·827	0·832	0·835	0·84	0·846	0·853	0·861	0·868	0·873	0·876	0·876
Ohio	0·766	0·769	0·773	0·777	0·78	0·783	0·785	0·786	0·788	0·79	0·793	0·798	0·802	0·804	0·807	0·807	0·806	0·808	0·812	0·817	0·823	0·826	0·829	0·832	0·835	0·838	0·841	0·843	0·845	0·846
Oklahoma	0·738	0·74	0·745	0·748	0·751	0·753	0·754	0·753	0·753	0·754	0·757	0·763	0·767	0·77	0·773	0·772	0·77	0·772	0·778	0·785	0·793	0·797	0·801	0·805	0·808	0·813	0·818	0·823	0·826	0·827
Oregon	0·773	0·776	0·78	0·784	0·787	0·79	0·793	0·795	0·797	0·8	0·804	0·81	0·814	0·817	0·82	0·82	0·82	0·823	0·827	0·833	0·838	0·842	0·845	0·848	0·852	0·856	0·86	0·864	0·866	0·868
Pennsylvania	0·787	0·791	0·795	0·8	0·803	0·807	0·809	0·811	0·813	0·815	0·818	0·822	0·826	0·828	0·83	0·831	0·831	0·833	0·837	0·841	0·846	0·849	0·852	0·855	0·857	0·861	0·864	0·866	0·868	0·87
Rhode Island	0·8	0·804	0·808	0·811	0·814	0·816	0·818	0·82	0·822	0·825	0·828	0·832	0·836	0·838	0·841	0·842	0·843	0·846	0·85	0·855	0·86	0·863	0·866	0·869	0·872	0·875	0·877	0·879	0·881	0·882
South Carolina	0·736	0·742	0·749	0·755	0·759	0·763	0·765	0·766	0·766	0·768	0·77	0·776	0·78	0·782	0·785	0·784	0·783	0·786	0·792	0·799	0·807	0·811	0·816	0·82	0·823	0·826	0·828	0·83	0·831	0·832
South Dakota	0·756	0·76	0·766	0·771	0·776	0·78	0·784	0·786	0·789	0·79	0·793	0·796	0·798	0·799	0·8	0·799	0·798	0·8	0·804	0·81	0·817	0·82	0·824	0·828	0·833	0·838	0·843	0·847	0·849	0·85
Tennessee	0·735	0·739	0·744	0·748	0·752	0·756	0·758	0·76	0·761	0·763	0·766	0·77	0·772	0·773	0·774	0·773	0·771	0·774	0·78	0·788	0·795	0·8	0·805	0·81	0·815	0·82	0·823	0·826	0·827	0·827
Texas	0·732	0·734	0·737	0·739	0·742	0·744	0·747	0·748	0·749	0·751	0·754	0·76	0·763	0·766	0·769	0·768	0·767	0·771	0·777	0·786	0·794	0·799	0·803	0·808	0·812	0·818	0·824	0·83	0·833	0·835
Utah	0·751	0·755	0·76	0·764	0·767	0·77	0·772	0·774	0·776	0·779	0·782	0·787	0·79	0·793	0·795	0·794	0·793	0·797	0·803	0·81	0·818	0·822	0·827	0·831	0·834	0·839	0·843	0·847	0·849	0·851
Vermont	0·801	0·806	0·81	0·814	0·817	0·82	0·822	0·824	0·827	0·829	0·833	0·838	0·841	0·845	0·848	0·85	0·851	0·853	0·856	0·86	0·864	0·866	0·869	0·872	0·875	0·879	0·883	0·885	0·887	0·889
Virginia	0·788	0·792	0·796	0·8	0·803	0·806	0·809	0·81	0·812	0·813	0·816	0·821	0·825	0·828	0·831	0·833	0·834	0·837	0·843	0·849	0·854	0·858	0·861	0·864	0·867	0·869	0·872	0·874	0·876	0·877
Washington	0·784	0·787	0·791	0·795	0·799	0·802	0·806	0·809	0·812	0·815	0·82	0·825	0·829	0·831	0·834	0·834	0·833	0·835	0·839	0·844	0·85	0·853	0·856	0·86	0·863	0·866	0·87	0·873	0·875	0·876
West Virginia	0·734	0·738	0·743	0·748	0·752	0·755	0·757	0·758	0·759	0·76	0·762	0·767	0·77	0·771	0·772	0·771	0·768	0·769	0·772	0·777	0·783	0·786	0·789	0·793	0·797	0·802	0·806	0·809	0·811	0·812

Table S1 Socio-demographic index values for all estimated GBD 2019 locations, 1990-201

Location	1990	1991	1992	1993	1994	1995	1996	1997	1998	1999	2000	2001	2002	2003	2004	2005	2006	2007	2008	2009	2010	2011	2012	2013	2014	2015	2016	2017	2018	2019
Wisconsin	0·79	0·793	0·797	0·801	0·804	0·807	0·81	0·811	0·813	0·815	0·818	0·822	0·825	0·827	0·83	0·83	0·829	0·831	0·835	0·839	0·844	0·848	0·851	0·855	0·858	0·861	0·864	0·866	0·868	0·869
Wyoming	0·758	0·763	0·769	0·774	0·779	0·782	0·785	0·786	0·788	0·789	0·792	0·796	0·799	0·802	0·805	0·805	0·805	0·809	0·815	0·823	0·829	0·833	0·837	0·84	0·843	0·847	0·85	0·853	0·855	0·856
Southern Latin America	0·584	0·589	0·597	0·602	0·608	0·614	0·62	0·625	0·63	0·634	0·64	0·644	0·648	0·651	0·653	0·658	0·663	0·664	0·667	0·671	0·676	0·681	0·686	0·689	0·692	0·701	0·71	0·716	0·719	0·721
Argentina	0·581	0·585	0·593	0·599	0·605	0·611	0·617	0·622	0·625	0·628	0·634	0·637	0·64	0·641	0·642	0·649	0·653	0·655	0·657	0·661	0·665	0·67	0·674	0·677	0·679	0·687	0·696	0·702	0·706	0·708
Chile	0·592	0·6	0·606	0·611	0·617	0·624	0·63	0·637	0·644	0·651	0·657	0·663	0·671	0·678	0·683	0·686	0·689	0·692	0·695	0·7	0·706	0·712	0·719	0·724	0·728	0·738	0·747	0·753	0·756	0·759
Uruguay	0·581	0·584	0·588	0·591	0·594	0·597	0·6	0·606	0·612	0·618	0·622	0·626	0·628	0·631	0·633	0·636	0·639	0·642	0·645	0·649	0·653	0·658	0·663	0·668	0·673	0·678	0·684	0·688	0·693	0·697
Western Europe	0·75	0·756	0·762	0·767	0·772	0·775	0·779	0·782	0·784	0·787	0·79	0·794	0·797	0·8	0·802	0·805	0·807	0·81	0·812	0·815	0·817	0·821	0·824	0·827	0·83	0·832	0·835	0·838	0·841	0·843
Andorra	0·834	0·838	0·84	0·841	0·841	0·841	0·843	0·845	0·847	0·849	0·851	0·854	0·855	0·859	0·862	0·865	0·867	0·869	0·872	0·874	0·876	0·879	0·881	0·883	0·885	0·887	0·889	0·891	0·892	0·894
Austria	0·753	0·754	0·757	0·761	0·768	0·773	0·778	0·783	0·787	0·791	0·795	0·799	0·803	0·805	0·808	0·811	0·815	0·818	0·821	0·824	0·826	0·83	0·833	0·835	0·838	0·839	0·841	0·844	0·847	0·849
Belgium	0·746	0·75	0·756	0·762	0·767	0·771	0·775	0·779	0·782	0·784	0·787	0·792	0·796	0·799	0·802	0·805	0·808	0·81	0·813	0·816	0·82	0·824	0·829	0·834	0·837	0·841	0·843	0·846	0·849	0·851
Cyprus	0·662	0·67	0·68	0·691	0·702	0·713	0·723	0·732	0·741	0·75	0·758	0·767	0·774	0·78	0·786	0·791	0·797	0·804	0·81	0·816	0·82	0·824	0·827	0·829	0·831	0·832	0·834	0·836	0·838	0·841
Denmark	0·806	0·809	0·813	0·816	0·82	0·824	0·828	0·833	0·836	0·84	0·844	0·848	0·852	0·855	0·858	0·86	0·862	0·864	0·865	0·867	0·87	0·873	0·875	0·878	0·88	0·882	0·884	0·886	0·888	0·89
Finland	0·757	0·759	0·762	0·765	0·769	0·773	0·777	0·782	0·785	0·788	0·792	0·797	0·802	0·805	0·808	0·812	0·815	0·818	0·821	0·824	0·828	0·831	0·834	0·837	0·84	0·844	0·848	0·851	0·853	0·856
France	0·738	0·743	0·75	0·755	0·759	0·763	0·767	0·77	0·773	0·775	0·777	0·781	0·785	0·787	0·79	0·793	0·796	0·799	0·801	0·803	0·806	0·809	0·812	0·815	0·819	0·822	0·826	0·829	0·832	0·834
Germany	0·819	0·83	0·834	0·838	0·841	0·843	0·844	0·844	0·844	0·844	0·847	0·853	0·856	0·858	0·861	0·863	0·866	0·869	0·873	0·875	0·878	0·881	0·883	0·886	0·888	0·89	0·892	0·894	0·896	0·898
Greece	0·682	0·688	0·695	0·702	0·707	0·713	0·717	0·723	0·729	0·735	0·74	0·743	0·748	0·753	0·758	0·761	0·765	0·768	0·771	0·775	0·779	0·782	0·785	0·786	0·786	0·787	0·788	0·79	0·792	0·794
Iceland	0·764	0·77	0·774	0·778	0·782	0·785	0·788	0·79	0·794	0·799	0·806	0·813	0·818	0·822	0·824	0·827	0·83	0·834	0·838	0·842	0·846	0·847	0·847	0·848	0·85	0·854	0·858	0·863	0·866	0·869
Ireland	0·73	0·735	0·741	0·747	0·753	0·758	0·763	0·768	0·774	0·78	0·786	0·793	0·799	0·806	0·812	0·816	0·819	0·821	0·824	0·827	0·831	0·835	0·839	0·842	0·845	0·85	0·854	0·859	0·864	0·867
Israel	0·717	0·721	0·726	0·73	0·734	0·738	0·742	0·745	0·749	0·752	0·756	0·76	0·762	0·765	0·769	0·773	0·776	0·778	0·778	0·779	0·781	0·784	0·787	0·79	0·792	0·794	0·796	0·798	0·8	0·803
Italy	0·712	0·717	0·722	0·727	0·732	0·737	0·74	0·744	0·747	0·75	0·753	0·757	0·761	0·763	0·766	0·768	0·771	0·773	0·775	0·777	0·78	0·782	0·784	0·787	0·789	0·791	0·794	0·796	0·798	0·801
Luxembourg	0·815	0·818	0·82	0·823	0·828	0·833	0·836	0·839	0·842	0·844	0·847	0·85	0·853	0·855	0·857	0·858	0·862	0·866	0·869	0·871	0·872	0·874	0·877	0·88	0·883	0·886	0·889	0·892	0·894	0·895
Malta	0·666	0·67	0·675	0·682	0·69	0·695	0·696	0·7	0·708	0·715	0·722	0·729	0·733	0·737	0·741	0·745	0·749	0·753	0·757	0·761	0·764	0·768	0·772	0·775	0·779	0·784	0·788	0·793	0·797	0·801
Monaco	0·834	0·837	0·84	0·843	0·846	0·849	0·852	0·855	0·857	0·86	0·862	0·865	0·867	0·87	0·872	0·875	0·877	0·879	0·881	0·883	0·886	0·888	0·89	0·892	0·893	0·895	0·897	0·899	0·901	0·902
Netherlands	0·796	0·801	0·806	0·81	0·814	0·818	0·821	0·824	0·827	0·83	0·832	0·836	0·839	0·842	0·845	0·848	0·851	0·853	0·856	0·858	0·861	0·864	0·866	0·869	0·871	0·874	0·876	0·878	0·881	0·883
Norway	0·807	0·812	0·818	0·823	0·828	0·832	0·837	0·843	0·847	0·851	0·856	0·861	0·866	0·869	0·872	0·874	0·876	0·877	0·879	0·881	0·885	0·889	0·893	0·896	0·9	0·903	0·907	0·91	0·912	0·913
Portugal	0·607	0·615	0·622	0·629	0·636	0·641	0·647	0·651	0·656	0·661	0·666	0·671	0·676	0·681	0·686	0·69	0·694	0·698	0·701	0·705	0·709	0·714	0·718	0·722	0·726	0·729	0·732	0·736	0·739	0·743
San Marino	0·814	0·817	0·82	0·824	0·828	0·832	0·837	0·841	0·846	0·85	0·852	0·854	0·856	0·859	0·861	0·862	0·864	0·866	0·868	0·87	0·872	0·874	0·876	0·877	0·879	0·881	0·882	0·882	0·883	0·884
Spain	0·647	0·655	0·662	0·669	0·675	0·681	0·686	0·691	0·696	0·7	0·705	0·709	0·713	0·717	0·721	0·725	0·728	0·73	0·734	0·739	0·743	0·746	0·749	0·752	0·754	0·756	0·759	0·761	0·764	0·767
Sweden	0·769	0·775	0·782	0·788	0·794	0·801	0·806	0·811	0·815	0·819	0·823	0·826	0·829	0·832	0·835	0·838	0·84	0·842	0·844	0·846	0·849	0·852	0·855	0·858	0·86	0·863	0·865	0·868	0·87	0·872
Stockholm	0·813	0·818	0·824	0·829	0·834	0·839	0·844	0·848	0·852	0·855	0·859	0·862	0·865	0·867	0·87	0·873	0·875	0·878	0·88	0·883	0·886	0·889	0·891	0·893	0·895	0·897	0·899	0·901	0·903	0·904
Sweden except Stockholm	0·758	0·764	0·771	0·777	0·784	0·79	0·796	0·801	0·805	0·809	0·813	0·816	0·819	0·822	0·825	0·827	0·83	0·832	0·834	0·835	0·838	0·841	0·844	0·846	0·849	0·851	0·853	0·856	0·858	0·86
Switzerland	0·868	0·869	0·873	0·876	0·878	0·88	0·881	0·883	0·884	0·886	0·889	0·893	0·894	0·896	0·898	0·9	0·902	0·905	0·908	0·909	0·912	0·914	0·917	0·919	0·921	0·922	0·924	0·926	0·928	0·929
UK	0·745	0·749	0·755	0·761	0·766	0·769	0·771	0·774	0·778	0·784	0·789	0·793	0·797	0·799	0·802	0·804	0·806	0·808	0·811	0·813	0·816	0·82	0·825	0·83	0·834	0·837	0·839	0·842	0·845	0·847
England	0·75	0·755	0·76	0·765	0·77	0·774	0·776	0·779	0·783	0·788	0·793	0·797	0·801	0·803	0·805	0·807	0·81	0·812	0·815	0·817	0·82	0·823	0·828	0·833	0·837	0·84	0·842	0·845	0·848	0·85
East Midlands	0·725	0·73	0·735	0·74	0·745	0·749	0·751	0·754	0·758	0·764	0·769	0·773	0·776	0·779	0·781	0·784	0·786	0·788	0·791	0·793	0·796	0·8	0·804	0·809	0·813	0·816	0·819	0·821	0·824	0·826
Derby	0·736	0·741	0·747	0·753	0·759	0·762	0·764	0·767	0·771	0·776	0·781	0·785	0·789	0·791	0·793	0·795	0·797	0·798	0·8	0·802	0·805	0·809	0·814	0·821	0·825	0·829	0·832	0·835	0·838	0·841
Derbyshire	0·716	0·72	0·725	0·73	0·735	0·738	0·74	0·743	0·746	0·751	0·756	0·759	0·762	0·764	0·766	0·768	0·77	0·772	0·774	0·776	0·779	0·783	0·789	0·795	0·799	0·803	0·806	0·809	0·811	0·814
Leicester	0·724	0·729	0·734	0·74	0·745	0·748	0·751	0·754	0·758	0·763	0·767	0·771	0·775	0·777	0·78	0·783	0·786	0·789	0·792	0·794	0·797	0·801	0·805	0·809	0·812	0·815	0·817	0·819	0·821	0·823
Leicestershire	0·754	0·758	0·763	0·768	0·773	0·776	0·778	0·781	0·784	0·789	0·793	0·797	0·8	0·802	0·805	0·807	0·81	0·812	0·815	0·817	0·819	0·822	0·826	0·83	0·833	0·835	0·838	0·84	0·842	0·845
Lincolnshire	0·717	0·721	0·726	0·731	0·736	0·739	0·741	0·743	0·747	0·753	0·757	0·761	0·764	0·765	0·767	0·769	0·77	0·771	0·773	0·774	0·777	0·781	0·786	0·791	0·795	0·799	0·802	0·804	0·807	0·81
Northamptonshire	0·734	0·739	0·745	0·75	0·756	0·758	0·76	0·762	0·765	0·77	0·774	0·777	0·779	0·781	0·782	0·784	0·786	0·788	0·79	0·792	0·795	0·799	0·804	0·81	0·814	0·817	0·82	0·822	0·825	0·827
Nottingham	0·754	0·759	0·764	0·769	0·774	0·777	0·781	0·784	0·789	0·794	0·799	0·804	0·807	0·81	0·813	0·815	0·818	0·82	0·823	0·826	0·828	0·832	0·836	0·84	0·842	0·844	0·846	0·848	0·85	0·852
Nottinghamshire	0·718	0·722	0·727	0·732	0·737	0·74	0·742	0·745	0·749	0·754	0·759	0·763	0·766	0·768	0·77	0·772	0·774	0·775	0·777	0·779	0·781	0·784	0·789	0·794	0·797	0·8	0·803	0·806	0·809	0·811
Rutland	0·764	0·769	0·773	0·778	0·782	0·784	0·786	0·788	0·79	0·794	0·797	0·8	0·802	0·803	0·806	0·808	0·81	0·813	0·816	0·818	0·821	0·824	0·827	0·831	0·834	0·836	0·838	0·84	0·842	0·844
East of England	0·744	0·749	0·754	0·759	0·764	0·767	0·77	0·773	0·777	0·782	0·787	0·791	0·794	0·797	0·799	0·801	0·804	0·806	0·809	0·811	0·814	0·817	0·822	0·827	0·83	0·833	0·836	0·839	0·841	0·844
Bedford	0·754	0·759	0·763	0·768	0·773	0·776	0·778	0·781	0·785	0·79	0·795	0·798	0·801	0·803	0·806	0·808	0·81	0·811	0·814	0·815	0·818	0·821	0·825	0·83	0·834	0·836	0·839	0·842	0·844	0·847
Cambridgeshire	0·784	0·789	0·794	0·798	0·803	0·806	0·809	0·812	0·816	0·82	0·825	0·829	0·832	0·835	0·837	0·839	0·842	0·844	0·846	0·848	0·851	0·854	0·858	0·862	0·865	0·868	0·87	0·872	0·875	0·877
Central Bedfordshire	0·751	0·755	0·76	0·765	0·77	0·773	0·775	0·778	0·781	0·785	0·79	0·793	0·796	0·798	0·8	0·802	0·804	0·805	0·808	0·809	0·811	0·815	0·819	0·824	0·827	0·83	0·833	0·836	0·839	0·841
Essex	0·735	0·74	0·745	0·751	0·756	0·759	0·762	0·765	0·768	0·773	0·778	0·782	0·786	0·788	0·791	0·794	0·796	0·798	0·801	0·803	0·805	0·809	0·813	0·817	0·821	0·823	0·825	0·828	0·83	0·833
Hertfordshire	0·783	0·788	0·793	0·798	0·802	0·806	0·808	0·811	0·815	0·82	0·826	0·83	0·834	0·837	0·839	0·842	0·844	0·846	0·848	0·849	0·851	0·854	0·857	0·862	0·865	0·867	0·87	0·872	0·875	0·877
Luton	0·729	0·734	0·739	0·745	0·75	0·753	0·756	0·759	0·762	0·767	0·772	0·775	0·778	0·78	0·783	0·786	0·789	0·793	0·797	0·8	0·803	0·807	0·812	0·817	0·82	0·823	0·825	0·828	0·83	0·832
Norfolk	0·726	0·73	0·735	0·74	0·745	0·749	0·751	0·754	0·758	0·763	0·768	0·772	0·776	0·778	0·78	0·782	0·784	0·786	0·788	0·79	0·793	0·796	0·801	0·806	0·81	0·813	0·815	0·818	0·821	0·823
Peterborough	0·722	0·726	0·73	0·735	0·74	0·743	0·745	0·748	0·752	0·757	0·761	0·765	0·767	0·768	0·769	0·77	0·772	0·773	0·776	0·777	0·78	0·785	0·791	0·798	0·802	0·806	0·809	0·813	0·816	0·818
Southend-on-Sea	0·714	0·718	0·722	0·727	0·732	0·735	0·737	0·74	0·745	0·751	0·756	0·761	0·765	0·768	0·77	0·772	0·774	0·776	0·778	0·78	0·783	0·787	0·792	0·797	0·8	0·803	0·805	0·807	0·81	0·812
Suffolk	0·731	0·736	0·741	0·747	0·751	0·754	0·756	0·758	0·762	0·767	0·771	0·775	0·777	0·779	0·781	0·782	0·785	0·786	0·789	0·791	0·794	0·799	0·804	0·81	0·813	0·816	0·819	0·822	0·824	0·827
Thurrock	0·717	0·722	0·727	0·733	0·738	0·741	0·744	0·747	0·751	0·756	0·759	0·762	0·764	0·765	0·766	0·767	0·768	0·77	0·772	0·774	0·776	0·78	0·784	0·789	0·792	0·794	0·796	0·799	0·801	0·804
Greater London	0·804	0·808	0·813	0·818	0·823	0·826	0·829	0·832	0·836	0·841	0·845	0·849	0·852	0·854	0·857	0·859	0·861	0·863	0·866	0·868	0·871	0·874	0·878	0·883	0·886	0·888	0·89	0·892	0·894	0·895
Barking and Dagenham	0·689	0·693	0·699	0·704	0·71	0·714	0·717	0·721	0·726	0·732	0·736	0·74	0·743	0·743	0·744	0·745	0·745	0·746	0·747	0·749	0·751	0·756	0·761	0·768	0·773	0·777	0·78	0·783	0·787	0·79
Barnet	0·786	0·791	0·796	0·8	0·805	0·808	0·811	0·815	0·819	0·824	0·829	0·833	0·837	0·839	0·841	0·843	0·844	0·846	0·848	0·849	0·852	0·855	0·859	0·863	0·866	0·869	0·872	0·874	0·876	0·878
Bexley	0·729	0·734	0·74	0·745	0·75	0·753	0·755	0·758	0·762	0·767	0·772	0·777	0·78	0·783	0·786	0·788	0·79	0·792	0·795	0·797	0·799	0·803	0·807	0·812	0·817	0·82	0·824	0·827	0·83	0·833
Brent	0·764	0·769	0·773	0·778	0·783	0·787	0·789	0·792	0·796	0·802	0·806	0·81	0·813	0·814	0·816	0·817	0·819	0·82	0·821	0·822	0·824	0·827	0·832	0·837	0·84	0·842	0·844	0·846	0·848	0·851
Bromley	0·77	0·774	0·779	0·784	0·788	0·792	0·794	0·797	0·802	0·807	0·812	0·816	0·82	0·822	0·825	0·827	0·829	0·83	0·832	0·833	0·835	0·837	0·841	0·844	0·847	0·849	0·852	0·854	0·856	0·858
Camden	0·872	0·875	0·879	0·882	0·885	0·888	0·89	0·892	0·895	0·898	0·9	0·903	0·904	0·906	0·907	0·909	0·911	0·912	0·914	0·915	0·917	0·92	0·922	0·925	0·926	0·928	0·929	0·93	0·931	0·931
Croydon	0·753	0·758	0·764	0·769	0·774	0·777	0·78	0·782	0·786	0·792	0·797	0·801	0·804	0·806	0·808	0·809	0·811	0·812	0·814	0·815	0·816	0·818	0·822	0·826	0·829	0·831	0·833	0·835	0·838	0·84
Ealing	0·783	0·787	0·792	0·797	0·802	0·806	0·809	0·812	0·816	0·821	0·826	0·83	0·833	0·835	0·836	0·837	0·838	0·839	0·84	0·841	0·842	0·845	0·849	0·854	0·858	0·862	0·865	0·868	0·87	0·872
Enfield	0·74	0·745	0·75	0·756	0·761	0·764	0·766	0·77	0·774	0·779	0·784	0·788	0·791	0·793	0·795	0·796	0·798	0·8	0·802	0·804	0·806	0·81	0·815	0·82	0·823	0·826	0·829	0·831	0·834	0·836
Greenwich	0·732	0·737	0·743	0·749	0·755	0·759	0·762	0·765	0·769	0·775	0·78	0·784	0·787	0·788	0·791	0·793	0·795	0·797	0·799	0·802	0·805	0·808	0·813	0·818	0·822	0·825	0·827	0·83	0·832	0·834
Hackney	0·787	0·793	0·799	0·805	0·809	0·812	0·812	0·813	0·815	0·819	0·823	0·826	0·828	0·83	0·832	0·835	0·838	0·841	0·845	0·849	0·853	0·858	0·864	0·87	0·873	0·876	0·877	0·879	0·881	0·882
Hammersmith and Fulha	0·854	0·859	0·864	0·869	0·873	0·876	0·879	0·882	0·886	0·89	0·893	0·895	0·897	0·898	0·9	0·902	0·903	0·905	0·907	0·909	0·912	0·914	0·917	0·92	0·922	0·924	0·925	0·927	0·928	0·929
Haringey	0·77	0·775	0·779	0·784	0·788	0·791	0·793	0·795	0·799	0·803	0·808	0·812	0·815	0·817	0·82	0·822	0·824	0·826	0·828	0·829	0·832	0·835	0·84	0·845	0·848	0·851	0·854	0·856	0·858	0·86
Harrow	0·772	0·777	0·782	0·787	0·791	0·794	0·797	0·8	0·804	0·81	0·814	0·819	0·822	0·824	0·826	0·828	0·83	0·831	0·832	0·833	0·834	0·836	0·839	0·843	0·845	0·847	0·848	0·85	0·852	0·854
Havering	0·722	0·727	0·732	0·737	0·741	0·744	0·747	0·75	0·754	0·759	0·764	0·769	0·772	0·775	0·778	0·781	0·784	0·786	0·788	0·79	0·793	0·796	0·8	0·805	0·808	0·811	0·814	0·817	0·82	0·823
Hillingdon	0·791	0·796	0·801	0·806	0·811	0·814	0·817	0·82	0·824	0·83	0·835	0·839	0·843	0·845	0·846	0·848	0·849	0·85	0·851	0·853	0·856	0·859	0·863	0·868	0·871	0·874	0·877	0·879	0·881	0·882
Hounslow	0·784	0·789	0·794	0·799	0·804	0·807	0·809	0·812	0·816	0·822	0·827	0·832	0·835	0·837	0·839	0·841	0·843	0·844	0·846	0·847	0·85	0·854	0·858	0·864	0·869	0·873	0·876	0·88	0·883	0·885
Islington	0·842	0·846	0·85	0·855	0·859	0·863	0·865	0·868	0·871	0·875	0·878	0·881	0·883	0·885	0·887	0·889	0·891	0·893	0·895	0·898	0·9	0·903	0·906	0·909	0·911	0·913	0·914	0·916	0·917	0·918
Kensington and Chelsea	0·882	0·886	0·89	0·894	0·897	0·9	0·903	0·905	0·907	0·909	0·91	0·912	0·914	0·915	0·916	0·917	0·919	0·921	0·923	0·924	0·926	0·929	0·931	0·934	0·935	0·937	0·938	0·939	0·94	0·941
Kingston upon Thames	0·816	0·821	0·826	0·83	0·835	0·838	0·841	0·845	0·849	0·854	0·859	0·863	0·866	0·869	0·872	0·874	0·877	0·879	0·881	0·882	0·884	0·886	0·888	0·891	0·893	0·895	0·896	0·898	0·9	0·902
Lambeth	0·799	0·805	0·81	0·815	0·82	0·824	0·827	0·83	0·834	0·839	0·844	0·848	0·851	0·854	0·857	0·861	0·864	0·867	0·871	0·874	0·878	0·881	0·886	0·89	0·894	0·897	0·899	0·902	0·904	0·906
Lewisham	0·747	0·753	0·758	0·763	0·768	0·772	0·775	0·778	0·782	0·787	0·792	0·797	0·8	0·802	0·804	0·806	0·808	0·81	0·812	0·814	0·816	0·82	0·824	0·829	0·833	0·836	0·839	0·842	0·844	0·846
Merton	0·777	0·783	0·788	0·793	0·798	0·802	0·805	0·809	0·814	0·819	0·824	0·828	0·831	0·833	0·835	0·836	0·837	0·839	0·841	0·842	0·845	0·848	0·853	0·858	0·862	0·866	0·869	0·872	0·874	0·876
Newham	0·717	0·722	0·727	0·733	0·738	0·741	0·743	0·746	0·751	0·757	0·763	0·768	0·773	0·776	0·779	0·783	0·786	0·788	0·792	0·794	0·798	0·803	0·808	0·814	0·818	0·822	0·824	0·827	0·829	0·831
Redbridge	0·748	0·752	0·757	0·762	0·767	0·77	0·773	0·776	0·78	0·785	0·79	0·794	0·797	0·8	0·802	0·804	0·806	0·808	0·81	0·811	0·813	0·816	0·82	0·825	0·828	0·831	0·834	0·836	0·839	0·841
Richmond upon Thames	0·836	0·841	0·845	0·85	0·854	0·857	0·86	0·863	0·867	0·872	0·876	0·88	0·883	0·886	0·888	0·89	0·892	0·894	0·895	0·897	0·899	0·901	0·904	0·908	0·911	0·914	0·917	0·92	0·922	0·924
Southwark	0·81	0·815	0·821	0·826	0·831	0·835	0·839	0·842	0·847	0·853	0·858	0·863	0·867	0·87	0·872	0·874	0·876	0·878	0·881	0·884	0·887	0·891	0·895	0·899	0·902	0·904	0·906	0·908	0·909	0·91
Sutton	0·748	0·753	0·758	0·763	0·769	0·773	0·776	0·78	0·784	0·79	0·795	0·8	0·803	0·806	0·808	0·81	0·812	0·813	0·815	0·816	0·819	0·822	0·826	0·831	0·835	0·838	0·841	0·844	0·847	0·849
Tower Hamlets	0·787	0·792	0·798	0·804	0·81	0·814	0·818	0·821	0·826	0·832	0·837	0·841	0·845	0·848	0·852	0·856	0·859	0·863	0·866	0·869	0·873	0·877	0·881	0·885	0·888	0·89	0·892	0·893	0·895	0·896
Waltham Forest	0·724	0·729	0·734	0·74	0·745	0·749	0·752	0·756	0·76	0·765	0·77	0·774	0·777	0·779	0·781	0·783	0·785	0·786	0·788	0·79	0·792	0·796	0·801	0·807	0·811	0·815	0·818	0·822	0·825	0·827
Wandsworth	0·828	0·833	0·838	0·843	0·848	0·851	0·855	0·858	0·863	0·868	0·872	0·877	0·88	0·883	0·886	0·888	0·891	0·893	0·895	0·897	0·899	0·901	0·904	0·907	0·909	0·911	0·913	0·915	0·916	0·918
Westminster	0·867	0·87	0·874	0·878	0·881	0·884	0·886	0·888	0·891	0·894	0·896	0·899	0·9	0·902	0·903	0·905	0·907	0·908	0·91	0·911	0·914	0·916	0·919	0·922	0·924	0·925	0·927	0·928	0·929	0·93
North East England	0·709	0·714	0·72	0·726	0·732	0·736	0·738	0·742	0·747	0·753	0·758	0·763	0·767	0·769	0·772	0·775	0·778	0·78	0·783	0·785	0·788	0·792	0·797	0·802	0·805	0·808	0·811	0·814	0·817	0·819
County Durham	0·705	0·71	0·715	0·721	0·726	0·729	0·732	0·735	0·739	0·744	0·749	0·754	0·757	0·759	0·762	0·764	0·767	0·769	0·772	0·774	0·777	0·78	0·785	0·79	0·793	0·796	0·798	0·801	0·803	0·805
Darlington	0·718	0·723	0·728	0·734	0·74	0·743	0·745	0·747	0·751	0·756	0·761	0·766	0·769	0·772	0·775	0·778	0·78	0·783	0·786	0·788	0·791	0·796	0·801	0·807	0·812	0·815	0·818	0·821	0·824	0·826
Gateshead	0·712	0·717	0·723	0·728	0·733	0·736	0·738	0·741	0·745	0·751	0·756	0·761	0·765	0·768	0·772	0·775	0·778	0·781	0·784	0·786	0·79	0·794	0·799	0·805	0·809	0·812	0·815	0·817	0·82	0·822
Hartlepool	0·678	0·684	0·689	0·695	0·7	0·704	0·705	0·708	0·712	0·717	0·722	0·726	0·729	0·73	0·732	0·735	0·737	0·739	0·742	0·745	0·749	0·753	0·759	0·766	0·771	0·774	0·778	0·781	0·784	0·786
Middlesbrough	0·692	0·698	0·703	0·709	0·714	0·718	0·72	0·723	0·727	0·733	0·738	0·742	0·745	0·748	0·751	0·753	0·756	0·758	0·761	0·763	0·765	0·769	0·774	0·78	0·783	0·786	0·788	0·791	0·793	0·796
Newcastle upon Tyne	0·763	0·768	0·773	0·778	0·783	0·787	0·79	0·794	0·798	0·803	0·808	0·813	0·818	0·822	0·826	0·83	0·834	0·838	0·841	0·844	0·846	0·849	0·853	0·856	0·858	0·86	0·861	0·863	0·865	0·866
North Tyneside	0·713	0·719	0·725	0·731	0·736	0·739	0·742	0·744	0·748	0·754	0·759	0·764	0·767	0·77	0·774	0·777	0·781	0·783	0·787	0·789	0·793	0·797	0·802	0·808	0·812	0·815	0·818	0·821	0·824	0·826
Northumberland	0·713	0·718	0·723	0·728	0·733	0·737	0·739	0·742	0·746	0·751	0·756	0·76	0·764	0·766	0·769	0·771	0·774	0·776	0·779	0·781	0·784	0·787	0·792	0·797	0·801	0·804	0·807	0·809	0·812	0·814
Redcar and Cleveland	0·686	0·691	0·697	0·703	0·708	0·712	0·714	0·717	0·721	0·726	0·731	0·735	0·737	0·739	0·741	0·744	0·746	0·748	0·751	0·753	0·756	0·76	0·765	0·772	0·776	0·78	0·783	0·786	0·789	0·791
South Tyneside	0·678	0·683	0·689	0·695	0·7	0·704	0·706	0·709	0·714	0·72	0·725	0·73	0·734	0·736	0·74	0·743	0·746	0·748	0·751	0·753	0·756	0·76	0·766	0·772	0·776	0·78	0·783	0·786	0·789	0·792
Stockton-on-Tees	0·721	0·727	0·733	0·74	0·746	0·75	0·752	0·755	0·76	0·765	0·769	0·773	0·775	0·777	0·779	0·781	0·783	0·784	0·787	0·788	0·791	0·795	0·8	0·805	0·809	0·812	0·815	0·817	0·82	0·822
Sunderland	0·699	0·705	0·712	0·718	0·724	0·728	0·731	0·734	0·738	0·744	0·749	0·753	0·757	0·759	0·762	0·765	0·768	0·77	0·772	0·775	0·777	0·781	0·786	0·792	0·796	0·799	0·802	0·805	0·807	0·81
North West England	0·73	0·735	0·74	0·745	0·75	0·754	0·756	0·759	0·763	0·768	0·773	0·777	0·781	0·783	0·785	0·788	0·79	0·792	0·795	0·797	0·8	0·804	0·809	0·815	0·819	0·822	0·825	0·827	0·83	0·832
Blackburn with Darwen	0·69	0·694	0·698	0·703	0·707	0·709	0·711	0·713	0·717	0·723	0·728	0·732	0·735	0·736	0·739	0·741	0·744	0·746	0·749	0·752	0·755	0·761	0·767	0·775	0·78	0·785	0·789	0·792	0·795	0·798
Blackpool	0·681	0·685	0·69	0·695	0·699	0·702	0·703	0·705	0·709	0·715	0·719	0·722	0·725	0·725	0·727	0·729	0·73	0·731	0·733	0·735	0·737	0·741	0·746	0·753	0·757	0·76	0·763	0·766	0·769	0·772
Bolton	0·708	0·713	0·718	0·723	0·728	0·731	0·732	0·734	0·737	0·741	0·746	0·749	0·751	0·752	0·754	0·756	0·757	0·759	0·761	0·763	0·766	0·77	0·775	0·782	0·786	0·789	0·792	0·795	0·797	0·8
Bury	0·722	0·727	0·733	0·738	0·743	0·746	0·748	0·75	0·754	0·759	0·763	0·766	0·769	0·77	0·772	0·774	0·776	0·778	0·78	0·781	0·784	0·788	0·793	0·8	0·804	0·808	0·811	0·814	0·817	0·819
Cheshire East	0·774	0·779	0·784	0·789	0·794	0·797	0·799	0·801	0·805	0·81	0·815	0·819	0·822	0·824	0·826	0·828	0·83	0·831	0·833	0·835	0·838	0·842	0·847	0·852	0·856	0·86	0·863	0·865	0·868	0·87
Cheshire West and Ches	0·766	0·771	0·776	0·781	0·786	0·789	0·791	0·794	0·798	0·802	0·807	0·811	0·814	0·816	0·819	0·821	0·824	0·826	0·829	0·831	0·834	0·837	0·842	0·847	0·85	0·853	0·855	0·858	0·86	0·862
Cumbria	0·729	0·733	0·737	0·742	0·747	0·75	0·752	0·754	0·758	0·763	0·767	0·771	0·773	0·775	0·777	0·779	0·781	0·783	0·786	0·789	0·793	0·797	0·803	0·81	0·815	0·819	0·822	0·825	0·828	0·83
Halton	0·711	0·716	0·722	0·727	0·733	0·736	0·737	0·739	0·742	0·747	0·751	0·755	0·758	0·759	0·761	0·763	0·766	0·768	0·771	0·774	0·778	0·783	0·789	0·797	0·802	0·806	0·81	0·813	0·817	0·819
Knowsley	0·69	0·694	0·699	0·704	0·708	0·711	0·713	0·715	0·72	0·725	0·731	0·736	0·739	0·742	0·744	0·747	0·75	0·752	0·755	0·758	0·762	0·766	0·772	0·779	0·783	0·787	0·79	0·794	0·797	0·8
Lancashire	0·736	0·741	0·746	0·752	0·757	0·76	0·762	0·765	0·769	0·773	0·778	0·781	0·784	0·786	0·788	0·79	0·792	0·794	0·796	0·799	0·801	0·805	0·81	0·815	0·819	0·822	0·824	0·827	0·829	0·831
Liverpool	0·738	0·742	0·747	0·752	0·757	0·76	0·763	0·766	0·771	0·777	0·782	0·787	0·792	0·796	0·8	0·804	0·808	0·811	0·815	0·818	0·821	0·824	0·827	0·831	0·833	0·835	0·836	0·838	0·84	0·842
Manchester	0·764	0·768	0·773	0·778	0·783	0·787	0·79	0·794	0·799	0·804	0·81	0·815	0·819	0·823	0·827	0·83	0·834	0·838	0·842	0·845	0·848	0·852	0·856	0·86	0·863	0·865	0·867	0·869	0·872	0·873
Oldham	0·686	0·691	0·696	0·701	0·705	0·707	0·707	0·708	0·711	0·715	0·719	0·722	0·725	0·726	0·728	0·73	0·733	0·735	0·738	0·741	0·744	0·749	0·755	0·762	0·766	0·77	0·773	0·776	0·779	0·781
Rochdale	0·689	0·693	0·698	0·704	0·709	0·712	0·713	0·716	0·719	0·725	0·729	0·733	0·736	0·737	0·739	0·741	0·743	0·745	0·747	0·749	0·753	0·757	0·763	0·77	0·774	0·778	0·781	0·784	0·787	0·79
Salford	0·719	0·723	0·728	0·734	0·739	0·742	0·744	0·747	0·752	0·758	0·763	0·769	0·773	0·775	0·778	0·781	0·784	0·786	0·788	0·79	0·793	0·798	0·803	0·81	0·814	0·817	0·82	0·822	0·825	0·827
Sefton	0·724	0·729	0·735	0·74	0·745	0·749	0·751	0·754	0·758	0·762	0·767	0·771	0·774	0·777	0·779	0·782	0·784	0·785	0·787	0·788	0·789	0·792	0·795	0·799	0·802	0·804	0·806	0·808	0·81	0·812
St Helens	0·7	0·704	0·709	0·714	0·719	0·721	0·723	0·725	0·729	0·734	0·739	0·743	0·746	0·748	0·75	0·752	0·755	0·757	0·759	0·761	0·765	0·769	0·774	0·78	0·784	0·787	0·789	0·792	0·794	0·797
Stockport	0·754	0·759	0·764	0·77	0·775	0·777	0·779	0·782	0·786	0·791	0·796	0·8	0·803	0·805	0·808	0·81	0·812	0·814	0·816	0·818	0·82	0·824	0·828	0·834	0·837	0·84	0·843	0·845	0·848	0·85
Tameside	0·697	0·702	0·707	0·712	0·717	0·721	0·723	0·725	0·729	0·735	0·739	0·743	0·746	0·747	0·749	0·75	0·751	0·752	0·753	0·754	0·757	0·76	0·766	0·772	0·776	0·779	0·782	0·785	0·788	0·79
Trafford	0·783	0·787	0·793	0·798	0·802	0·805	0·807	0·81	0·813	0·818	0·823	0·827	0·831	0·834	0·837	0·839	0·842	0·844	0·846	0·848	0·851	0·854	0·859	0·864	0·867	0·871	0·874	0·876	0·879	0·881
Warrington	0·762	0·767	0·772	0·777	0·781	0·784	0·785	0·787	0·791	0·796	0·801	0·806	0·81	0·812	0·815	0·818	0·821	0·823	0·825	0·827	0·83	0·834	0·84	0·846	0·85	0·854	0·858	0·861	0·864	0·866
Wigan	0·7	0·705	0·709	0·714	0·719	0·722	0·723	0·726	0·729	0·734	0·738	0·741	0·744	0·745	0·747	0·749	0·751	0·752	0·754	0·756	0·759	0·763	0·769	0·775	0·779	0·782	0·785	0·788	0·791	0·793
Wirral	0·711	0·716	0·721	0·726	0·731	0·734	0·736	0·739	0·742	0·747	0·752	0·755	0·758	0·76	0·762	0·764	0·766	0·767	0·769	0·771	0·774	0·777	0·783	0·788	0·792	0·796	0·798	0·801	0·804	0·806
South East England	0·777	0·782	0·786	0·791	0·795	0·798	0·8	0·803	0·806	0·811	0·815	0·819	0·822	0·823	0·826	0·828	0·83	0·832	0·834	0·836	0·838	0·841	0·845	0·85	0·853	0·855	0·858	0·86	0·862	0·864
Bracknell Forest	0·79	0·794	0·798	0·802	0·805	0·807	0·809	0·812	0·815	0·821	0·826	0·83	0·833	0·836	0·838	0·84	0·842	0·843	0·845	0·847	0·849	0·852	0·857	0·861	0·865	0·868	0·871	0·873	0·876	0·878
Brighton and Hove	0·799	0·804	0·808	0·813	0·817	0·821	0·824	0·828	0·832	0·836	0·841	0·845	0·849	0·852	0·856	0·859	0·862	0·865	0·867	0·869	0·871	0·873	0·876	0·879	0·881	0·883	0·885	0·887	0·889	0·891
Buckinghamshire	0·799	0·803	0·807	0·811	0·816	0·819	0·821	0·823	0·827	0·831	0·835	0·839	0·841	0·843	0·845	0·846	0·848	0·849	0·851	0·852	0·853	0·856	0·86	0·864	0·866	0·869	0·871	0·873	0·875	0·877
East Sussex	0·732	0·737	0·742	0·747	0·752	0·755	0·757	0·76	0·763	0·768	0·773	0·777	0·78	0·782	0·784	0·786	0·788	0·789	0·791	0·793	0·795	0·799	0·803	0·809	0·812	0·816	0·819	0·822	0·825	0·827
Hampshire	0·774	0·779	0·783	0·787	0·792	0·794	0·796	0·799	0·802	0·807	0·811	0·815	0·818	0·819	0·821	0·823	0·825	0·827	0·829	0·83	0·833	0·836	0·841	0·846	0·85	0·853	0·855	0·858	0·86	0·862
Isle of Wight	0·712	0·716	0·721	0·727	0·732	0·735	0·738	0·741	0·745	0·751	0·755	0·76	0·763	0·764	0·766	0·768	0·771	0·772	0·775	0·776	0·778	0·781	0·785	0·79	0·793	0·796	0·798	0·8	0·803	0·805
Kent	0·744	0·748	0·753	0·757	0·762	0·765	0·767	0·769	0·773	0·777	0·781	0·785	0·788	0·79	0·792	0·794	0·796	0·798	0·8	0·802	0·805	0·808	0·813	0·818	0·821	0·824	0·826	0·828	0·831	0·833
Medway	0·72	0·725	0·73	0·735	0·74	0·743	0·745	0·747	0·75	0·754	0·758	0·762	0·764	0·766	0·768	0·77	0·772	0·774	0·776	0·778	0·78	0·783	0·788	0·792	0·796	0·798	0·8	0·802	0·805	0·807
Milton Keynes	0·777	0·782	0·787	0·792	0·797	0·8	0·801	0·803	0·806	0·81	0·814	0·817	0·82	0·821	0·823	0·824	0·826	0·827	0·829	0·831	0·834	0·838	0·844	0·85	0·855	0·859	0·862	0·866	0·869	0·87
Oxfordshire	0·805	0·809	0·813	0·817	0·822	0·824	0·827	0·829	0·832	0·837	0·841	0·844	0·847	0·849	0·852	0·854	0·856	0·858	0·86	0·862	0·864	0·867	0·871	0·875	0·878	0·881	0·883	0·886	0·888	0·89
Portsmouth	0·772	0·776	0·781	0·786	0·79	0·794	0·797	0·8	0·804	0·808	0·813	0·816	0·819	0·822	0·824	0·826	0·828	0·83	0·832	0·834	0·836	0·839	0·842	0·846	0·849	0·85	0·852	0·854	0·856	0·858
Reading	0·819	0·823	0·827	0·832	0·836	0·839	0·841	0·845	0·848	0·853	0·858	0·862	0·865	0·868	0·871	0·872	0·873	0·874	0·875	0·876	0·878	0·88	0·883	0·886	0·889	0·89	0·891	0·893	0·894	0·896
Slough	0·78	0·785	0·79	0·795	0·8	0·804	0·806	0·809	0·813	0·816	0·819	0·821	0·823	0·823	0·823	0·824	0·824	0·824	0·826	0·828	0·831	0·835	0·841	0·847	0·851	0·854	0·857	0·86	0·862	0·864
Southampton	0·772	0·777	0·782	0·787	0·791	0·795	0·799	0·803	0·807	0·812	0·816	0·82	0·823	0·826	0·828	0·83	0·831	0·833	0·834	0·835	0·837	0·839	0·841	0·844	0·846	0·848	0·849	0·851	0·853	0·855
Surrey	0·812	0·816	0·82	0·824	0·828	0·831	0·833	0·836	0·84	0·845	0·849	0·853	0·856	0·859	0·861	0·863	0·865	0·866	0·868	0·87	0·871	0·874	0·877	0·881	0·884	0·886	0·889	0·891	0·893	0·895
West Berkshire	0·807	0·812	0·817	0·822	0·826	0·829	0·831	0·833	0·836	0·841	0·845	0·849	0·851	0·851	0·851	0·851	0·852	0·852	0·853	0·855	0·857	0·861	0·865	0·87	0·874	0·876	0·878	0·88	0·882	0·883

Table S1 Socio-demographic index values for all estimated GBD 2019 locations, 1990-2019

Location	1990	1991	1992	1993	1994	1995	1996	1997	1998	1999	2000	2001	2002	2003	2004	2005	2006	2007	2008	2009	2010	2011	2012	2013	2014	2015	2016	2017	2018	2019
West Sussex	0·765	0·769	0·774	0·779	0·783	0·786	0·788	0·79	0·794	0·798	0·803	0·807	0·81	0·811	0·814	0·815	0·817	0·819	0·821	0·823	0·825	0·829	0·833	0·838	0·841	0·844	0·847	0·849	0·852	0·854
Windsor and Maidenhea	0·82	0·823	0·827	0·831	0·834	0·837	0·839	0·841	0·845	0·849	0·854	0·858	0·861	0·863	0·866	0·868	0·87	0·872	0·874	0·875	0·877	0·88	0·884	0·889	0·892	0·895	0·898	0·9	0·903	0·905
Wokingham	0·826	0·831	0·835	0·839	0·843	0·846	0·848	0·851	0·854	0·859	0·863	0·866	0·869	0·871	0·874	0·876	0·878	0·879	0·881	0·881	0·883	0·885	0·887	0·891	0·893	0·896	0·898	0·9	0·902	0·904
South West England	0·751	0·755	0·76	0·765	0·77	0·773	0·776	0·779	0·783	0·788	0·793	0·797	0·8	0·802	0·805	0·807	0·81	0·812	0·814	0·816	0·819	0·823	0·827	0·832	0·835	0·838	0·841	0·843	0·846	0·848
Bath and North East Sor	0·793	0·797	0·801	0·805	0·809	0·812	0·815	0·818	0·822	0·827	0·831	0·836	0·84	0·844	0·848	0·851	0·855	0·858	0·861	0·863	0·865	0·868	0·871	0·874	0·876	0·878	0·88	0·882	0·884	0·886
Bournemouth	0·761	0·766	0·771	0·776	0·781	0·785	0·788	0·792	0·796	0·801	0·806	0·811	0·815	0·818	0·822	0·825	0·828	0·831	0·833	0·835	0·838	0·84	0·844	0·847	0·85	0·852	0·854	0·856	0·858	0·861
Bristol, City of	0·792	0·796	0·801	0·806	0·811	0·814	0·817	0·821	0·825	0·829	0·834	0·837	0·841	0·843	0·845	0·848	0·85	0·853	0·856	0·858	0·861	0·864	0·868	0·872	0·875	0·877	0·88	0·882	0·884	0·886
Cornwall	0·727	0·732	0·738	0·743	0·748	0·752	0·754	0·756	0·76	0·765	0·77	0·774	0·777	0·779	0·782	0·784	0·787	0·789	0·791	0·793	0·796	0·799	0·804	0·809	0·812	0·815	0·818	0·82	0·823	0·825
Devon	0·747	0·752	0·757	0·762	0·766	0·769	0·771	0·774	0·778	0·783	0·788	0·792	0·796	0·798	0·802	0·805	0·807	0·81	0·812	0·814	0·817	0·821	0·825	0·829	0·832	0·835	0·837	0·839	0·841	0·843
Dorset	0·741	0·746	0·751	0·755	0·76	0·763	0·765	0·768	0·772	0·777	0·782	0·785	0·788	0·79	0·792	0·794	0·795	0·797	0·8	0·802	0·805	0·808	0·813	0·818	0·822	0·825	0·827	0·83	0·833	0·835
Gloucestershire	0·764	0·768	0·773	0·778	0·783	0·786	0·788	0·79	0·794	0·799	0·803	0·808	0·811	0·814	0·816	0·819	0·821	0·823	0·825	0·827	0·829	0·833	0·837	0·842	0·846	0·849	0·852	0·855	0·857	0·859
North Somerset	0·744	0·749	0·754	0·759	0·764	0·766	0·768	0·77	0·773	0·778	0·782	0·786	0·789	0·791	0·794	0·796	0·799	0·801	0·804	0·806	0·81	0·814	0·819	0·825	0·829	0·833	0·836	0·838	0·841	0·843
Plymouth	0·746	0·751	0·755	0·76	0·765	0·768	0·77	0·773	0·776	0·781	0·785	0·789	0·791	0·793	0·796	0·798	0·8	0·802	0·805	0·807	0·809	0·812	0·816	0·821	0·824	0·826	0·828	0·83	0·832	0·834
Poole	0·755	0·76	0·765	0·77	0·775	0·778	0·78	0·783	0·787	0·792	0·797	0·801	0·803	0·805	0·807	0·809	0·811	0·813	0·815	0·817	0·819	0·823	0·828	0·833	0·837	0·84	0·843	0·846	0·848	0·85
Somerset	0·732	0·736	0·742	0·747	0·752	0·755	0·757	0·759	0·763	0·768	0·772	0·775	0·778	0·78	0·782	0·784	0·786	0·787	0·79	0·791	0·794	0·798	0·802	0·808	0·812	0·815	0·818	0·821	0·824	0·826
South Gloucestershire	0·776	0·78	0·784	0·788	0·792	0·795	0·797	0·801	0·805	0·81	0·815	0·819	0·823	0·825	0·828	0·831	0·833	0·836	0·839	0·841	0·843	0·847	0·851	0·856	0·86	0·863	0·865	0·868	0·871	0·873
Swindon	0·765	0·77	0·775	0·781	0·785	0·788	0·789	0·791	0·794	0·798	0·802	0·806	0·808	0·809	0·81	0·812	0·813	0·814	0·817	0·818	0·821	0·825	0·83	0·835	0·839	0·842	0·844	0·846	0·849	0·85
Torbay	0·71	0·715	0·72	0·725	0·73	0·733	0·735	0·738	0·741	0·746	0·75	0·753	0·755	0·755	0·757	0·758	0·76	0·761	0·762	0·763	0·765	0·768	0·772	0·777	0·78	0·782	0·785	0·787	0·79	0·793
Wiltshire	0·751	0·755	0·759	0·764	0·769	0·771	0·773	0·776	0·779	0·784	0·789	0·793	0·796	0·798	0·8	0·802	0·804	0·805	0·807	0·809	0·811	0·815	0·82	0·825	0·829	0·833	0·836	0·839	0·841	0·844
West Midlands	0·716	0·721	0·727	0·733	0·738	0·742	0·744	0·747	0·752	0·757	0·763	0·767	0·771	0·773	0·776	0·778	0·781	0·783	0·786	0·789	0·792	0·796	0·801	0·807	0·811	0·814	0·817	0·82	0·823	0·825
Birmingham	0·719	0·724	0·73	0·736	0·741	0·744	0·746	0·749	0·754	0·759	0·764	0·769	0·773	0·775	0·778	0·781	0·784	0·787	0·79	0·793	0·797	0·801	0·806	0·811	0·815	0·818	0·82	0·823	0·825	0·827
Coventry	0·738	0·744	0·75	0·756	0·761	0·765	0·769	0·772	0·777	0·782	0·787	0·791	0·794	0·796	0·799	0·801	0·802	0·804	0·806	0·807	0·81	0·813	0·818	0·823	0·826	0·829	0·832	0·835	0·837	0·84
Dudley	0·699	0·704	0·708	0·714	0·718	0·721	0·723	0·726	0·73	0·735	0·74	0·744	0·747	0·748	0·751	0·752	0·754	0·756	0·757	0·758	0·76	0·763	0·768	0·773	0·777	0·779	0·782	0·785	0·788	0·79
Herefordshire, County o	0·722	0·727	0·733	0·739	0·745	0·748	0·751	0·755	0·759	0·765	0·771	0·775	0·779	0·781	0·783	0·786	0·788	0·789	0·792	0·793	0·796	0·8	0·805	0·811	0·816	0·819	0·823	0·826	0·829	0·832
Sandwell	0·677	0·682	0·687	0·692	0·698	0·701	0·702	0·705	0·709	0·714	0·719	0·723	0·726	0·728	0·73	0·731	0·733	0·734	0·737	0·738	0·741	0·746	0·752	0·759	0·764	0·768	0·772	0·775	0·778	0·781
Shropshire	0·728	0·733	0·738	0·744	0·749	0·752	0·754	0·757	0·762	0·768	0·773	0·778	0·781	0·784	0·787	0·79	0·792	0·795	0·797	0·799	0·802	0·806	0·81	0·815	0·819	0·822	0·825	0·827	0·83	0·832
Solihull	0·756	0·762	0·767	0·773	0·778	0·782	0·785	0·788	0·792	0·798	0·803	0·808	0·811	0·813	0·816	0·818	0·82	0·822	0·823	0·825	0·827	0·83	0·834	0·839	0·842	0·846	0·849	0·852	0·855	0·858
Staffordshire	0·725	0·73	0·736	0·742	0·747	0·75	0·752	0·755	0·759	0·765	0·769	0·773	0·776	0·778	0·78	0·782	0·784	0·786	0·789	0·79	0·793	0·797	0·801	0·805	0·808	0·811	0·813	0·815	0·817	0·819
Stoke-on-Trent	0·687	0·692	0·698	0·703	0·709	0·712	0·714	0·717	0·72	0·725	0·729	0·732	0·734	0·734	0·735	0·736	0·737	0·737	0·74	0·742	0·745	0·75	0·756	0·764	0·769	0·773	0·776	0·78	0·783	0·786
Telford and Wrekin	0·718	0·723	0·729	0·735	0·741	0·744	0·747	0·75	0·754	0·759	0·764	0·767	0·769	0·769	0·77	0·771	0·772	0·773	0·774	0·775	0·778	0·782	0·787	0·794	0·798	0·801	0·804	0·807	0·81	0·813
Walsall	0·681	0·685	0·689	0·694	0·699	0·701	0·702	0·703	0·706	0·711	0·716	0·72	0·723	0·724	0·726	0·728	0·73	0·732	0·735	0·737	0·74	0·745	0·751	0·758	0·762	0·766	0·77	0·773	0·776	0·779
Warwickshire	0·754	0·76	0·765	0·771	0·776	0·779	0·782	0·785	0·789	0·795	0·799	0·803	0·807	0·808	0·811	0·813	0·815	0·817	0·82	0·822	0·825	0·829	0·834	0·839	0·843	0·846	0·849	0·852	0·855	0·857
Wolverhampton	0·695	0·7	0·706	0·712	0·717	0·721	0·723	0·726	0·73	0·736	0·741	0·745	0·748	0·75	0·752	0·754	0·756	0·758	0·76	0·761	0·764	0·768	0·774	0·78	0·784	0·787	0·79	0·793	0·796	0·799
Worcestershire	0·731	0·736	0·741	0·747	0·752	0·755	0·757	0·761	0·765	0·772	0·777	0·782	0·785	0·788	0·79	0·792	0·794	0·796	0·798	0·799	0·802	0·805	0·809	0·814	0·818	0·82	0·823	0·826	0·828	0·831
Yorkshire and the Humber	0·721	0·726	0·732	0·737	0·742	0·745	0·748	0·751	0·755	0·76	0·765	0·77	0·773	0·775	0·778	0·78	0·783	0·785	0·788	0·79	0·793	0·797	0·802	0·808	0·812	0·815	0·818	0·821	0·823	0·826
Barnsley	0·677	0·681	0·685	0·69	0·695	0·697	0·699	0·702	0·706	0·711	0·716	0·719	0·722	0·723	0·725	0·727	0·729	0·73	0·732	0·734	0·737	0·741	0·746	0·753	0·758	0·761	0·765	0·768	0·771	0·774
Bradford	0·698	0·703	0·708	0·713	0·718	0·72	0·721	0·722	0·725	0·729	0·733	0·737	0·74	0·741	0·744	0·746	0·749	0·751	0·754	0·757	0·761	0·766	0·773	0·78	0·784	0·788	0·791	0·794	0·797	0·8
Calderdale	0·719	0·724	0·73	0·736	0·741	0·743	0·745	0·747	0·75	0·755	0·76	0·763	0·766	0·767	0·769	0·771	0·773	0·775	0·777	0·78	0·783	0·788	0·794	0·802	0·806	0·81	0·813	0·816	0·819	0·821
Doncaster	0·677	0·681	0·686	0·691	0·696	0·698	0·699	0·701	0·705	0·71	0·715	0·719	0·722	0·724	0·727	0·729	0·732	0·734	0·737	0·739	0·743	0·748	0·754	0·761	0·766	0·77	0·774	0·777	0·781	0·783
East Riding of Yorkshire	0·732	0·737	0·742	0·748	0·753	0·756	0·758	0·76	0·764	0·769	0·773	0·777	0·78	0·781	0·784	0·786	0·788	0·789	0·791	0·792	0·794	0·798	0·802	0·806	0·81	0·812	0·814	0·817	0·819	0·821
Kingston upon Hull, City	0·692	0·696	0·702	0·707	0·712	0·716	0·718	0·722	0·727	0·733	0·738	0·743	0·747	0·749	0·751	0·754	0·756	0·758	0·76	0·762	0·764	0·768	0·772	0·778	0·781	0·783	0·785	0·787	0·79	0·792
Kirklees	0·715	0·72	0·726	0·731	0·736	0·738	0·74	0·742	0·745	0·75	0·754	0·758	0·761	0·762	0·764	0·767	0·769	0·77	0·773	0·775	0·777	0·782	0·787	0·793	0·797	0·8	0·803	0·805	0·808	0·81
Leeds	0·762	0·767	0·773	0·778	0·783	0·787	0·79	0·793	0·798	0·803	0·808	0·812	0·816	0·819	0·822	0·826	0·829	0·831	0·834	0·836	0·838	0·842	0·845	0·849	0·853	0·855	0·858	0·86	0·862	0·864
North East Lincolnshire	0·682	0·688	0·694	0·7	0·705	0·708	0·709	0·711	0·714	0·719	0·723	0·726	0·729	0·729	0·731	0·733	0·735	0·737	0·741	0·743	0·746	0·751	0·757	0·764	0·768	0·771	0·774	0·778	0·781	0·784
North Lincolnshire	0·722	0·727	0·732	0·737	0·742	0·745	0·746	0·748	0·752	0·756	0·76	0·762	0·764	0·765	0·767	0·768	0·77	0·772	0·775	0·777	0·781	0·785	0·79	0·796	0·799	0·8	0·801	0·803	0·805	0·806
North Yorkshire	0·746	0·75	0·755	0·76	0·765	0·767	0·769	0·772	0·775	0·781	0·786	0·79	0·793	0·796	0·798	0·801	0·803	0·804	0·807	0·808	0·811	0·815	0·819	0·824	0·827	0·83	0·832	0·834	0·836	0·838
Rotherham	0·686	0·69	0·695	0·701	0·706	0·708	0·71	0·712	0·715	0·72	0·725	0·729	0·732	0·734	0·737	0·739	0·741	0·743	0·745	0·747	0·75	0·754	0·76	0·766	0·77	0·773	0·775	0·778	0·781	0·784
Sheffield	0·749	0·753	0·758	0·762	0·767	0·77	0·773	0·776	0·78	0·785	0·789	0·794	0·798	0·801	0·804	0·807	0·811	0·814	0·817	0·819	0·822	0·826	0·83	0·834	0·837	0·84	0·842	0·844	0·847	0·848
Wakefield	0·694	0·699	0·704	0·71	0·715	0·718	0·72	0·722	0·726	0·731	0·735	0·739	0·742	0·743	0·745	0·746	0·748	0·749	0·751	0·753	0·755	0·76	0·765	0·772	0·777	0·78	0·783	0·786	0·789	0·792
York	0·795	0·799	0·804	0·809	0·813	0·816	0·819	0·822	0·826	0·83	0·834	0·839	0·842	0·845	0·849	0·852	0·855	0·857	0·859	0·861	0·863	0·865	0·868	0·871	0·873	0·875	0·877	0·879	0·881	0·883
Northern Ireland	0·701	0·707	0·716	0·726	0·733	0·737	0·738	0·74	0·744	0·751	0·759	0·765	0·77	0·774	0·777	0·779	0·781	0·783	0·786	0·789	0·792	0·796	0·802	0·808	0·811	0·815	0·818	0·821	0·823	0·825
Scotland	0·724	0·728	0·735	0·743	0·75	0·754	0·754	0·756	0·76	0·768	0·774	0·78	0·784	0·787	0·789	0·791	0·792	0·794	0·797	0·801	0·805	0·809	0·814	0·819	0·823	0·826	0·828	0·831	0·833	0·834
Wales	0·706	0·711	0·717	0·723	0·729	0·733	0·736	0·74	0·745	0·751	0·757	0·762	0·766	0·769	0·772	0·775	0·778	0·78	0·783	0·785	0·788	0·792	0·797	0·802	0·806	0·809	0·812	0·815	0·818	0·82
Latin America and Caribbean	0·491	0·496	0·501	0·506	0·511	0·516	0·521	0·526	0·531	0·536	0·541	0·546	0·55	0·554	0·558	0·563	0·568	0·573	0·578	0·583	0·589	0·594	0·6	0·605	0·611	0·616	0·62	0·625	0·629	0·633
Andean Latin America	0·489	0·492	0·496	0·5	0·504	0·509	0·514	0·519	0·524	0·529	0·534	0·538	0·543	0·548	0·553	0·558	0·564	0·569	0·575	0·58	0·585	0·591	0·596	0·602	0·608	0·613	0·618	0·624	0·628	0·632
Bolivia	0·412	0·417	0·422	0·428	0·434	0·441	0·448	0·455	0·462	0·469	0·474	0·48	0·485	0·491	0·496	0·501	0·506	0·51	0·514	0·518	0·521	0·525	0·528	0·533	0·538	0·544	0·55	0·556	0·562	0·566
Ecuador	0·503	0·506	0·51	0·514	0·517	0·521	0·524	0·528	0·532	0·535	0·539	0·544	0·549	0·554	0·559	0·565	0·57	0·575	0·581	0·586	0·591	0·597	0·603	0·609	0·615	0·621	0·626	0·632	0·636	0·64
Peru	0·501	0·505	0·508	0·511	0·516	0·521	0·526	0·532	0·537	0·543	0·548	0·552	0·557	0·562	0·566	0·571	0·577	0·583	0·589	0·595	0·601	0·608	0·614	0·62	0·625	0·63	0·635	0·64	0·645	0·648
Caribbean	0·517	0·522	0·525	0·528	0·531	0·533	0·536	0·539	0·542	0·547	0·552	0·557	0·563	0·569	0·573	0·579	0·585	0·588	0·591	0·594	0·598	0·601	0·605	0·609	0·612	0·616	0·621	0·625	0·628	0·631
Antigua and Barbuda	0·579	0·586	0·592	0·598	0·604	0·61	0·616	0·623	0·631	0·638	0·645	0·652	0·659	0·665	0·672	0·679	0·686	0·694	0·7	0·705	0·709	0·713	0·716	0·719	0·723	0·727	0·731	0·735	0·739	0·743
The Bahamas	0·692	0·684	0·676	0·679	0·686	0·692	0·697	0·702	0·71	0·721	0·731	0·735	0·737	0·739	0·742	0·749	0·755	0·759	0·762	0·767	0·774	0·779	0·78	0·782	0·784	0·786	0·789	0·791	0·794	0·796
Barbados	0·649	0·653	0·658	0·663	0·667	0·67	0·672	0·674	0·676	0·677	0·68	0·683	0·687	0·691	0·695	0·699	0·703	0·707	0·711	0·714	0·718	0·721	0·725	0·728	0·73	0·733	0·735	0·737	0·74	0·742
Belize	0·428	0·437	0·447	0·457	0·466	0·475	0·482	0·488	0·494	0·499	0·505	0·51	0·515	0·521	0·528	0·534	0·541	0·547	0·553	0·559	0·564	0·569	0·574	0·579	0·583	0·588	0·592	0·596	0·6	0·603
Bermuda	0·685	0·689	0·693	0·697	0·701	0·704	0·707	0·71	0·714	0·719	0·724	0·731	0·737	0·743	0·749	0·755	0·76	0·766	0·773	0·779	0·785	0·79	0·795	0·799	0·802	0·805	0·807	0·809	0·811	0·813
Cuba	0·578	0·586	0·589	0·588	0·586	0·583	0·583	0·582	0·579	0·581	0·586	0·588	0·595	0·602	0·605	0·611	0·618	0·618	0·616	0·618	0·62	0·624	0·631	0·636	0·64	0·645	0·653	0·66	0·665	0·668
Dominica	0·579	0·585	0·591	0·597	0·604	0·61	0·617	0·623	0·63	0·637	0·643	0·649	0·655	0·66	0·666	0·672	0·677	0·683	0·689	0·695	0·7	0·705	0·709	0·713	0·717	0·721	0·724	0·727	0·728	0·729
Dominican Republic	0·425	0·427	0·43	0·434	0·437	0·442	0·446	0·452	0·458	0·465	0·471	0·478	0·485	0·492	0·499	0·508	0·516	0·524	0·532	0·538	0·544	0·549	0·554	0·559	0·563	0·569	0·574	0·58	0·587	0·592
Grenada	0·463	0·474	0·485	0·495	0·505	0·515	0·524	0·533	0·543	0·553	0·562	0·57	0·577	0·585	0·592	0·6	0·606	0·613	0·619	0·624	0·628	0·633	0·637	0·641	0·645	0·65	0·654	0·659	0·664	0·669
Guyana	0·452	0·456	0·461	0·467	0·474	0·481	0·488	0·496	0·503	0·51	0·516	0·522	0·527	0·532	0·536	0·54	0·544	0·549	0·554	0·559	0·565	0·571	0·577	0·583	0·59	0·596	0·602	0·608	0·614	0·618
Haiti	0·307	0·311	0·314	0·317	0·32	0·323	0·328	0·333	0·338	0·344	0·35	0·356	0·361	0·366	0·371	0·375	0·379	0·383	0·387	0·392	0·395	0·399	0·403	0·407	0·412	0·416	0·42	0·424	0·428	0·432
Jamaica	0·542	0·547	0·553	0·56	0·566	0·573	0·58	0·586	0·592	0·598	0·603	0·609	0·614	0·62	0·625	0·63	0·635	0·64	0·645	0·649	0·653	0·657	0·661	0·664	0·668	0·671	0·675	0·678	0·681	0·684
Puerto Rico	0·67	0·676	0·679	0·684	0·689	0·693	0·699	0·705	0·711	0·714	0·72	0·731	0·738	0·742	0·744	0·748	0·752	0·756	0·76	0·764	0·769	0·774	0·779	0·785	0·793	0·802	0·808	0·811	0·813	0·814
Saint Kitts and Nevis	0·583	0·592	0·599	0·607	0·614	0·62	0·626	0·633	0·638	0·644	0·65	0·657	0·663	0·668	0·673	0·679	0·685	0·69	0·696	0·701	0·706	0·71	0·714	0·719	0·724	0·728	0·733	0·738	0·742	0·746
Saint Lucia	0·483	0·493	0·504	0·514	0·524	0·534	0·543	0·551	0·56	0·568	0·575	0·581	0·587	0·593	0·6	0·606	0·612	0·617	0·623	0·629	0·634	0·639	0·643	0·648	0·652	0·656	0·659	0·663	0·667	0·67
Saint Vincent and the Grenadines	0·462	0·471	0·479	0·487	0·495	0·502	0·509	0·515	0·522	0·528	0·534	0·539	0·545	0·551	0·557	0·562	0·568	0·574	0·58	0·585	0·589	0·593	0·598	0·602	0·606	0·61	0·615	0·619	0·623	0·627
Suriname	0·498	0·503	0·507	0·511	0·513	0·515	0·518	0·523	0·528	0·533	0·538	0·545	0·552	0·56	0·568	0·574	0·579	0·584	0·588	0·593	0·598	0·602	0·607	0·611	0·616	0·62	0·625	0·629	0·633	0·636
Trinidad and Tobago	0·618	0·622	0·626	0·63	0·634	0·639	0·644	0·65	0·656	0·662	0·669	0·675	0·682	0·689	0·696	0·704	0·711	0·718	0·724	0·728	0·732	0·736	0·739	0·742	0·745	0·748	0·751	0·753	0·755	0·757
Virgin Islands	0·667	0·68	0·689	0·697	0·704	0·71	0·715	0·72	0·724	0·728	0·731	0·734	0·742	0·749	0·756	0·762	0·768	0·773	0·778	0·782	0·785	0·788	0·79	0·791	0·792	0·794	0·795	0·796	0·798	0·799
Central Latin America	0·485	0·49	0·495	0·501	0·507	0·512	0·517	0·523	0·528	0·533	0·538	0·543	0·547	0·551	0·554	0·559	0·564	0·569	0·575	0·579	0·584	0·589	0·594	0·599	0·604	0·609	0·614	0·618	0·623	0·626
Colombia	0·478	0·48	0·482	0·486	0·492	0·498	0·503	0·509	0·514	0·518	0·522	0·526	0·529	0·533	0·536	0·54	0·546	0·552	0·559	0·567	0·574	0·582	0·589	0·596	0·603	0·609	0·616	0·622	0·628	0·633
Costa Rica	0·532	0·538	0·544	0·55	0·555	0·56	0·565	0·571	0·577	0·584	0·592	0·598	0·603	0·607	0·611	0·615	0·619	0·623	0·627	0·632	0·637	0·642	0·647	0·652	0·657	0·662	0·667	0·672	0·676	0·68
El Salvador	0·39	0·393	0·397	0·402	0·408	0·415	0·423	0·431	0·441	0·45	0·46	0·469	0·478	0·486	0·493	0·499	0·505	0·511	0·517	0·521	0·526	0·531	0·536	0·542	0·547	0·552	0·558	0·563	0·568	0·573
Guatemala	0·315	0·32	0·328	0·336	0·345	0·352	0·36	0·369	0·378	0·387	0·395	0·403	0·412	0·421	0·43	0·439	0·446	0·453	0·459	0·465	0·472	0·478	0·485	0·491	0·498	0·504	0·51	0·516	0·522	0·526
Honduras	0·33	0·335	0·34	0·345	0·351	0·357	0·363	0·37	0·377	0·384	0·391	0·397	0·404	0·411	0·417	0·424	0·43	0·437	0·443	0·448	0·454	0·459	0·464	0·469	0·473	0·478	0·482	0·487	0·492	0·496
Mexico	0·507	0·514	0·52	0·526	0·532	0·537	0·542	0·547	0·553	0·558	0·563	0·569	0·574	0·578	0·583	0·588	0·592	0·597	0·601	0·605	0·608	0·613	0·617	0·621	0·626	0·631	0·636	0·64	0·645	0·649
Aguascalientes	0·532	0·538	0·544	0·549	0·554	0·559	0·564	0·569	0·574	0·58	0·585	0·591	0·597	0·602	0·607	0·611	0·616	0·621	0·625	0·629	0·633	0·637	0·641	0·646	0·65	0·655	0·66	0·664	0·669	0·673
Baja California	0·565	0·57	0·575	0·58	0·585	0·589	0·593	0·598	0·604	0·609	0·615	0·619	0·624	0·628	0·632	0·636	0·64	0·644	0·647	0·65	0·653	0·656	0·66	0·663	0·667	0·671	0·675	0·68	0·685	0·688
Baja California Sur	0·546	0·553	0·559	0·565	0·571	0·576	0·582	0·588	0·594	0·599	0·604	0·609	0·614	0·619	0·625	0·63	0·636	0·641	0·646	0·649	0·653	0·658	0·662	0·666	0·67	0·675	0·68	0·684	0·689	0·693
Campeche	0·481	0·488	0·495	0·502	0·509	0·515	0·522	0·529	0·536	0·542	0·548	0·554	0·56	0·565	0·571	0·576	0·581	0·586	0·591	0·595	0·6	0·606	0·611	0·617	0·623	0·629	0·634	0·639	0·644	0·648
Chiapas	0·413	0·419	0·424	0·43	0·437	0·442	0·446	0·451	0·456	0·461	0·466	0·47	0·475	0·48	0·486	0·491	0·497	0·503	0·508	0·512	0·517	0·522	0·527	0·532	0·536	0·541	0·545	0·55	0·554	0·557
Chihuahua	0·529	0·534	0·538	0·542	0·546	0·549	0·553	0·558	0·563	0·568	0·573	0·578	0·584	0·588	0·593	0·598	0·602	0·607	0·611	0·614	0·617	0·62	0·623	0·627	0·632	0·637	0·643	0·648	0·654	0·659
Coahuila	0·529	0·536	0·543	0·549	0·556	0·561	0·566	0·571	0·576	0·581	0·586	0·591	0·595	0·6	0·604	0·609	0·613	0·617	0·62	0·623	0·625	0·628	0·631	0·634	0·639	0·644	0·648	0·653	0·658	0·663
Colima	0·535	0·543	0·551	0·557	0·563	0·568	0·574	0·58	0·585	0·59	0·596	0·601	0·606	0·611	0·615	0·62	0·625	0·629	0·634	0·638	0·642	0·646	0·65	0·654	0·659	0·664	0·669	0·673	0·678	0·682
Durango	0·453	0·46	0·466	0·472	0·478	0·483	0·489	0·496	0·504	0·511	0·517	0·524	0·53	0·537	0·543	0·548	0·553	0·558	0·563	0·566	0·571	0·575	0·58	0·585	0·591	0·596	0·602	0·608	0·614	0·618
Guanajuato	0·452	0·459	0·467	0·474	0·481	0·488	0·495	0·502	0·51	0·517	0·525	0·532	0·538	0·544	0·55	0·556	0·562	0·567	0·572	0·577	0·582	0·588	0·594	0·6	0·606	0·612	0·618	0·624	0·63	0·634
Guerrero	0·408	0·414	0·419	0·424	0·429	0·434	0·44	0·446	0·452	0·457	0·464	0·47	0·476	0·481	0·487	0·493	0·498	0·504	0·51	0·516	0·522	0·529	0·535	0·541	0·548	0·553	0·558	0·563	0·568	0·572
Hidalgo	0·43	0·438	0·445	0·453	0·46	0·467	0·474	0·481	0·488	0·494	0·501	0·507	0·513	0·518	0·524	0·53	0·536	0·542	0·548	0·553	0·559	0·565	0·571	0·577	0·584	0·59	0·596	0·601	0·606	0·611
Jalisco	0·523	0·53	0·537	0·544	0·551	0·556	0·561	0·566	0·572	0·577	0·582	0·587	0·591	0·596	0·6	0·604	0·608	0·613	0·617	0·621	0·625	0·629	0·634	0·638	0·643	0·647	0·652	0·657	0·661	0·665
México	0·536	0·544	0·551	0·558	0·565	0·569	0·573	0·577	0·58	0·583	0·587	0·591	0·595	0·599	0·604	0·608	0·612	0·616	0·62	0·622	0·625	0·629	0·632	0·636	0·64	0·644	0·648	0·652	0·657	0·66
Mexico City	0·601	0·607	0·614	0·621	0·627	0·633	0·638	0·644	0·65	0·656	0·662	0·667	0·671	0·675	0·679	0·683	0·687	0·69	0·693	0·695	0·697	0·7	0·704	0·708	0·712	0·716	0·72	0·724	0·729	0·732
Michoacán de Ocampo	0·459	0·467	0·475	0·482	0·487	0·492	0·497	0·503	0·508	0·514	0·519	0·524	0·529	0·534	0·54	0·545	0·55	0·555	0·56	0·564	0·568	0·572	0·576	0·581	0·585	0·59	0·594	0·598	0·602	0·606
Morelos	0·522	0·53	0·536	0·542	0·548	0·553	0·559	0·564	0·57	0·575	0·581	0·586	0·59	0·594	0·597	0·601	0·604	0·608	0·611	0·614	0·617	0·621	0·624	0·628	0·632	0·637	0·641	0·646	0·65	0·654
Nayarit	0·489	0·495	0·501	0·506	0·511	0·516	0·522	0·529	0·535	0·541	0·548	0·555	0·561	0·567	0·574	0·58	0·586	0·591	0·595	0·599	0·603	0·607	0·612	0·617	0·621	0·626	0·63	0·635	0·639	0·643
Nuevo León	0·577	0·583	0·589	0·594	0·599	0·602	0·606	0·611	0·615	0·62	0·625	0·629	0·634	0·638	0·642	0·645	0·649	0·652	0·656	0·658	0·661	0·665	0·669	0·673	0·677	0·682	0·686	0·691	0·696	0·699
Oaxaca	0·426	0·429	0·433	0·437	0·441	0·445	0·449	0·453	0·458	0·462	0·467	0·472	0·477	0·483	0·49	0·496	0·502	0·509	0·516	0·522	0·528	0·534	0·54	0·545	0·55	0·555	0·561	0·566	0·571	0·574
Puebla	0·46	0·467	0·473	0·48	0·486	0·49	0·495	0·5	0·504	0·509	0·514	0·52	0·525	0·53	0·536	0·541	0·546	0·552	0·557	0·561	0·567	0·572	0·577	0·583	0·588	0·593	0·598	0·603	0·608	0·612
Querétaro	0·507	0·514	0·52	0·526	0·533	0·539	0·545	0·552	0·559	0·567	0·575	0·584	0·591	0·597	0·603	0·608	0·613	0·618	0·622	0·626	0·63	0·634	0·639	0·644	0·649	0·653	0·658	0·663	0·667	0·671
Quintana Roo	0·494	0·504	0·513	0·522	0·531	0·54	0·549	0·557	0·565	0·572	0·579	0·585	0·591	0·596	0·601	0·606	0·61	0·615	0·62	0·624	0·629	0·634	0·638	0·643	0·647	0·652	0·657	0·662	0·666	0·67
San Luis Potosí	0·451	0·457	0·464	0·471	0·478	0·486	0·493	0·501	0·508	0·516	0·523	0·53	0·536	0·543	0·55	0·557	0·564	0·571	0·578	0·584	0·59	0·596	0·602	0·607	0·612	0·617	0·622	0·626	0·631	0·635
Sinaloa	0·519	0·525	0·53	0·536	0·542	0·547	0·553	0·559	0·565	0·57	0·576	0·58	0·585	0·589	0·594	0·599	0·604	0·608	0·613	0·617	0·621	0·626	0·631	0·636	0·641	0·647	0·652	0·657	0·662	0·667
Sonora	0·546	0·552	0·558	0·564	0·569	0·574	0·579	0·584	0·59	0·596	0·602	0·607	0·612	0·616	0·621	0·626	0·631	0·635	0·639	0·642	0·645	0·649	0·653	0·658	0·662	0·668	0·673	0·678	0·683	0·687
Tabasco	0·485	0·492	0·499	0·505	0·511	0·516	0·523	0·529	0·536	0·542	0·548	0·553	0·558	0·562	0·566	0·571	0·575	0·58	0·584	0·588	0·592	0·597	0·602	0·608	0·613	0·619	0·624	0·629	0·634	0·638
Tamaulipas	0·529	0·535	0·54	0·546	0·551	0·557	0·563	0·57	0·576	0·581	0·585	0·589	0·594	0·598	0·602	0·606	0·61	0·614	0·618	0·621	0·624	0·628	0·632	0·636	0·641	0·647	0·652	0·657	0·662	0·666
Tlaxcala	0·474	0·483	0·492	0·501	0·509	0·516	0·523	0·529	0·536	0·542	0·549	0·555	0·561	0·566	0·572	0·577	0·582	0·587	0·592	0·596	0·601	0·605	0·61	0·614	0·619	0·623	0·628	0·632	0·636	0·64
Veracruz de Ignacio de la Llave	0·471	0·475	0·48	0·485	0·489	0·493	0·497	0·502	0·507	0·512	0·517	0·522	0·527	0·532	0·537	0·542	0·548	0·553	0·558	0·562	0·567	0·572	0·578	0·582	0·587	0·592	0·596	0·601	0·605	0·609
Yucatán	0·478	0·484	0·49	0·497	0·504	0·511	0·517	0·524	0·53	0·535	0·54	0·545	0·549	0·553	0·559	0·564	0·571	0·577	0·583	0·588	0·594	0·599	0·605	0·611	0·616	0·622	0·627	0·631	0·636	0·64
Zacatecas	0·493	0·499	0·505	0·511	0·516	0·52	0·525	0·529	0·534	0·539	0·544	0·55	0·555	0·56	0·564	0·568	0·572	0·576	0·581	0·584	0·587	0·591	0·595	0·599	0·604	0·609	0·615	0·62	0·625	0·63
Nicaragua	0·338	0·345	0·353	0·36	0·368	0·376	0·385	0·393	0·402	0·411	0·419	0·426	0·433	0·438	0·444	0·449	0·453	0·458	0·462	0·466	0·47	0·474	0·479	0·484	0·489	0·495	0·5	0·506	0·512	0·517
Panama	0·544	0·549	0·555	0·559	0·562	0·565	0·568	0·573	0·579	0·586	0·592	0·597	0·602	0·605	0·607	0·61	0·613	0·616	0·62	0·623	0·627	0·63	0·635	0·642	0·65	0·658	0·666	0·674	0·68	0·686
Venezuela	0·509	0·514	0·522	0·53	0·537	0·542	0·546	0·551	0·554	0·557	0·559	0·56	0·558	0·552	0·548	0·55	0·557	0·566	0·575	0·582	0·586	0·591	0·596	0·6	0·604	0·607	0·608	0·608	0·608	0·607
Tropical Latin America	0·487	0·492	0·497	0·503	0·508	0·513	0·519	0·524	0·529	0·533	0·538	0·543	0·547	0·552	0·556	0·561	0·566	0·572	0·578	0·584	0·59	0·597	0·604	0·61	0·617	0·622	0·627	0·632	0·636	0·64
Brazil	0·487	0·492	0·498	0·503	0·508	0·513	0·519	0·524	0·529	0·533	0·538	0·543	0·547	0·551	0·556	0·561	0·566	0·572	0·577	0·583	0·59	0·597	0·603	0·61	0·616	0·622	0·627	0·632	0·636	0·64
Acre	0·364	0·371	0·379	0·386	0·394	0·402	0·41	0·417	0·423	0·429	0·435	0·441	0·446	0·452	0·458	0·464	0·47	0·477	0·485	0·493	0·501	0·509	0·518	0·526	0·533	0·54	0·547	0·552	0·558	0·562
Alagoas	0·356	0·362	0·367	0·372	0·377	0·382	0·387	0·391	0·396	0·4	0·404	0·408	0·413	0·417	0·422	0·427	0·433	0·439	0·446	0·453	0·461	0·469	0·477	0·485	0·492	0·498	0·504	0·509	0·514	0·518
Amapá	0·494	0·501	0·508	0·515	0·521	0·528	0·534	0·54	0·545	0·549	0·553	0·557	0·56	0·563	0·566	0·569	0·573	0·578	0·583	0·588	0·594	0·6	0·606	0·612	0·618	0·624	0·629	0·633	0·637	0·641
Amazonas	0·459	0·467	0·474	0·481	0·486	0·491	0·495	0·498	0·501	0·503	0·505	0·507	0·51	0·512	0·516	0·52	0·524	0·53	0·536	0·541	0·548	0·555	0·562	0·569	0·576	0·582	0·588	0·593	0·598	0·602
Bahia	0·397	0·404	0·409	0·415	0·421	0·426	0·431	0·435	0·44	0·444	0·448	0·452	0·456	0·461	0·466	0·471	0·477	0·483	0·491	0·498	0·505	0·514	0·521	0·529	0·536	0·542	0·548	0·553	0·558	0·562
Ceará	0·386	0·392	0·398	0·404	0·41	0·416	0·422	0·428	0·433	0·437	0·442	0·447	0·451	0·456	0·461	0·466	0·472	0·479	0·486	0·493	0·501	0·51	0·518	0·525	0·533	0·539	0·544	0·549	0·554	0·558
Distrito Federal	0·587	0·594	0·602	0·61	0·619	0·63	0·641	0·652	0·661	0·669	0·676	0·683	0·689	0·694	0·699	0·704	0·709	0·715	0·72	0·726	0·732	0·739	0·745	0·751	0·756	0·761	0·766	0·77	0·774	0·777
Espírito Santo	0·479	0·485	0·491	0·498	0·504	0·511	0·518	0·525	0·531	0·537	0·543	0·548	0·554	0·56	0·566	0·572	0·579	0·586	0·593	0·599	0·607	0·614	0·622	0·629	0·636	0·642	0·647	0·652	0·657	0·66
Goiás	0·442	0·449	0·455	0·462	0·469	0·476	0·483	0·49	0·496	0·502	0·508	0·514	0·52	0·526	0·532	0·538	0·545	0·551	0·558	0·565	0·573	0·581	0·588	0·596	0·603	0·609	0·614	0·619	0·624	0·628
Maranhão	0·274	0·281	0·287	0·293	0·299	0·305	0·311	0·316	0·32	0·324	0·327	0·33	0·333	0·336	0·34	0·344	0·349	0·355	0·361	0·368	0·376	0·385	0·394	0·403	0·412	0·42	0·427	0·433	0·439	0·444
Mato Grosso	0·448	0·455	0·462	0·469	0·477	0·484	0·491	0·497	0·503	0·509	0·516	0·522	0·528	0·535	0·543	0·55	0·557	0·564	0·572	0·579	0·587	0·595	0·603	0·61	0·617	0·623	0·629	0·634	0·638	0·642
Mato Grosso do Sul	0·471	0·477	0·483	0·488	0·494	0·5	0·505	0·511	0·516	0·521	0·526	0·531	0·536	0·542	0·547	0·553	0·558	0·565	0·571	0·578	0·585	0·593	0·601	0·608	0·615	0·621	0·626	0·631	0·636	0·639
Minas Gerais	0·487	0·493	0·498	0·504	0·51	0·515	0·52	0·525	0·529	0·534	0·538	0·542	0·547	0·552	0·558	0·563	0·569	0·576	0·582	0·589	0·596	0·603	0·61	0·616	0·622	0·627	0·632	0·636	0·64	0·643
Pará	0·425	0·432	0·438	0·444	0·449	0·454	0·457	0·46	0·462	0·464	0·465	0·467	0·469	0·472	0·475	0·479	0·484	0·489	0·496	0·502	0·51	0·518	0·526	0·534	0·542	0·549	0·554	0·56	0·565	0·569
Paraíba	0·378	0·385	0·391	0·396	0·402	0·408	0·413	0·418	0·422	0·426	0·431	0·435	0·439	0·444	0·449	0·454	0·46	0·467	0·474	0·482	0·49	0·499	0·507	0·515	0·522	0·528	0·534	0·539	0·544	0·548
Paraná	0·499	0·504	0·509	0·514	0·519	0·525	0·531	0·537	0·543	0·549	0·555	0·561	0·567	0·573	0·579	0·584	0·59	0·596	0·603	0·609	0·615	0·621	0·628	0·634	0·64	0·645	0·65	0·654	0·659	0·662
Pernambuco	0·407	0·414	0·419	0·424	0·429	0·434	0·438	0·442	0·446	0·45	0·453	0·457	0·461	0·465	0·47	0·475	0·481	0·488	0·495	0·502	0·51	0·519	0·527	0·536	0·543	0·55	0·556	0·561	0·567	0·571
Piauí	0·334	0·34	0·345	0·351	0·357	0·363	0·369	0·375	0·38	0·384	0·388	0·392	0·396	0·4	0·405	0·41	0·416	0·423	0·431	0·439	0·448	0·457	0·466	0·474	0·482	0·489	0·494	0·5	0·505	0·509
Rio de Janeiro	0·569	0·574	0·578	0·582	0·586	0·59	0·595	0·6	0·604	0·609	0·613	0·618	0·622	0·626	0·63	0·634	0·639	0·643	0·648	0·653	0·658	0·664	0·67	0·675	0·681	0·686	0·69	0·694	0·698	0·702
Rio Grande do Norte	0·402	0·408	0·413	0·419	0·424	0·429	0·434	0·44	0·444	0·449	0·454	0·459	0·464	0·469	0·475	0·481	0·488	0·495	0·503	0·51	0·519	0·527	0·535	0·543	0·551	0·557	0·562	0·567	0·572	0·576
Rio Grande do Sul	0·549	0·554	0·558	0·563	0·567	0·572	0·576	0·58	0·584	0·588	0·593	0·597	0·602	0·606	0·611	0·616	0·62	0·626	0·631	0·636	0·642	0·648	0·653	0·659	0·664	0·669	0·673	0·677	0·681	0·684

Table S1 Socio-demographic index values for all estimated GBD 2019 locations, 1990-201

Location	1990	1991	1992	1993	1994	1995	1996	1997	1998	1999	2000	2001	2002	2003	2004	2005	2006	2007	2008	2009	2010	2011	2012	2013	2014	2015	2016	2017	2018	2019
Rondônia	0·43	0·436	0·441	0·446	0·451	0·455	0·46	0·465	0·47	0·474	0·48	0·485	0·49	0·496	0·503	0·509	0·516	0·524	0·531	0·539	0·547	0·556	0·565	0·573	0·58	0·587	0·592	0·598	0·603	0·606
Roraima	0·425	0·431	0·437	0·441	0·446	0·452	0·458	0·465	0·471	0·476	0·482	0·488	0·494	0·5	0·506	0·512	0·519	0·526	0·534	0·541	0·55	0·558	0·566	0·575	0·582	0·589	0·595	0·601	0·606	0·61
Santa Catarina	0·539	0·544	0·549	0·554	0·559	0·564	0·569	0·574	0·579	0·584	0·589	0·594	0·6	0·605	0·611	0·616	0·622	0·628	0·634	0·64	0·646	0·652	0·659	0·665	0·67	0·676	0·68	0·684	0·688	0·691
São Paulo	0·554	0·559	0·564	0·569	0·575	0·581	0·587	0·593	0·599	0·604	0·61	0·615	0·62	0·624	0·629	0·633	0·638	0·643	0·648	0·652	0·658	0·663	0·669	0·675	0·68	0·686	0·69	0·694	0·698	0·702
Sergipe	0·424	0·43	0·436	0·441	0·446	0·451	0·456	0·46	0·465	0·469	0·473	0·478	0·483	0·488	0·493	0·498	0·504	0·511	0·518	0·524	0·532	0·539	0·546	0·553	0·56	0·566	0·57	0·575	0·579	0·583
Tocantins	0·361	0·366	0·37	0·374	0·38	0·386	0·393	0·4	0·408	0·415	0·422	0·43	0·438	0·447	0·456	0·465	0·474	0·484	0·494	0·504	0·514	0·524	0·534	0·544	0·552	0·56	0·567	0·573	0·579	0·583
Paraguay	0·465	0·471	0·477	0·484	0·491	0·498	0·505	0·512	0·519	0·524	0·529	0·534	0·539	0·544	0·549	0·554	0·559	0·564	0·57	0·575	0·582	0·588	0·594	0·601	0·608	0·615	0·621	0·627	0·633	0·638
North Africa and Middle East	0·414	0·423	0·432	0·441	0·45	0·46	0·469	0·478	0·487	0·497	0·506	0·515	0·524	0·532	0·541	0·55	0·56	0·569	0·578	0·587	0·595	0·603	0·611	0·619	0·626	0·633	0·64	0·647	0·654	0·66
North Africa and Middle East	0·414	0·423	0·432	0·441	0·45	0·46	0·469	0·478	0·487	0·497	0·506	0·515	0·524	0·532	0·541	0·55	0·56	0·569	0·578	0·587	0·595	0·603	0·611	0·619	0·626	0·633	0·64	0·647	0·654	0·66
Afghanistan	0·187	0·191	0·195	0·196	0·194	0·194	0·193	0·192	0·19	0·189	0·188	0·188	0·194	0·202	0·209	0·216	0·224	0·234	0·243	0·253	0·264	0·274	0·285	0·295	0·304	0·313	0·321	0·329	0·337	0·343
Algeria	0·436	0·446	0·456	0·465	0·474	0·483	0·492	0·5	0·509	0·518	0·526	0·534	0·542	0·55	0·558	0·566	0·573	0·58	0·587	0·593	0·599	0·605	0·611	0·617	0·623	0·628	0·634	0·64	0·646	0·652
Bahrain	0·553	0·56	0·566	0·573	0·58	0·587	0·595	0·602	0·61	0·618	0·626	0·634	0·643	0·653	0·662	0·672	0·68	0·688	0·697	0·705	0·711	0·715	0·72	0·726	0·731	0·735	0·739	0·743	0·747	0·751
Egypt	0·403	0·415	0·426	0·437	0·448	0·459	0·469	0·478	0·487	0·496	0·504	0·512	0·52	0·527	0·535	0·542	0·549	0·557	0·565	0·573	0·582	0·591	0·6	0·609	0·617	0·626	0·635	0·643	0·651	0·658
Iran	0·404	0·419	0·433	0·446	0·457	0·468	0·477	0·486	0·496	0·506	0·517	0·529	0·541	0·552	0·562	0·571	0·581	0·593	0·603	0·613	0·622	0·63	0·635	0·64	0·645	0·649	0·654	0·659	0·665	0·67
Iraq	0·392	0·397	0·402	0·407	0·412	0·417	0·422	0·429	0·439	0·451	0·462	0·475	0·485	0·493	0·504	0·514	0·525	0·535	0·547	0·558	0·57	0·583	0·597	0·61	0·622	0·632	0·644	0·654	0·663	0·671
Jordan	0·52	0·529	0·537	0·546	0·554	0·562	0·57	0·577	0·585	0·592	0·6	0·607	0·614	0·621	0·63	0·639	0·648	0·656	0·664	0·673	0·681	0·688	0·695	0·702	0·707	0·713	0·718	0·723	0·727	0·731
Kuwait	0·655	0·659	0·662	0·667	0·673	0·68	0·689	0·699	0·709	0·717	0·724	0·729	0·735	0·742	0·75	0·76	0·769	0·777	0·785	0·793	0·801	0·808	0·815	0·822	0·828	0·834	0·839	0·844	0·848	0·851
Lebanon	0·462	0·47	0·477	0·485	0·493	0·502	0·511	0·52	0·53	0·54	0·548	0·557	0·565	0·574	0·582	0·591	0·6	0·609	0·618	0·628	0·639	0·649	0·66	0·67	0·677	0·685	0·691	0·698	0·704	0·708
Libya	0·405	0·422	0·438	0·455	0·472	0·489	0·506	0·522	0·538	0·552	0·566	0·58	0·593	0·606	0·619	0·632	0·645	0·658	0·67	0·681	0·691	0·695	0·703	0·707	0·707	0·707	0·705	0·705	0·707	0·709
Morocco	0·347	0·354	0·361	0·367	0·374	0·38	0·386	0·392	0·398	0·403	0·409	0·414	0·42	0·426	0·432	0·439	0·445	0·452	0·46	0·467	0·475	0·483	0·491	0·499	0·508	0·516	0·524	0·533	0·541	0·548
Oman	0·441	0·455	0·469	0·485	0·5	0·514	0·528	0·543	0·558	0·573	0·588	0·601	0·615	0·628	0·641	0·653	0·664	0·678	0·692	0·704	0·715	0·726	0·737	0·747	0·754	0·76	0·767	0·773	0·778	0·783
Palestine	0·314	0·32	0·329	0·337	0·347	0·356	0·365	0·375	0·386	0·397	0·407	0·415	0·422	0·43	0·439	0·449	0·458	0·467	0·476	0·486	0·497	0·509	0·521	0·533	0·543	0·553	0·564	0·573	0·582	0·588
Qatar	0·585	0·598	0·61	0·621	0·632	0·643	0·654	0·666	0·676	0·685	0·694	0·703	0·711	0·719	0·727	0·735	0·743	0·75	0·757	0·764	0·772	0·779	0·786	0·793	0·799	0·806	0·812	0·818	0·825	0·83
Saudi Arabia	0·48	0·491	0·504	0·516	0·529	0·541	0·554	0·566	0·578	0·59	0·602	0·614	0·625	0·637	0·65	0·664	0·677	0·69	0·703	0·715	0·726	0·738	0·75	0·76	0·769	0·778	0·786	0·793	0·8	0·805
Sudan	0·227	0·234	0·241	0·248	0·256	0·263	0·271	0·28	0·289	0·298	0·308	0·318	0·327	0·337	0·348	0·358	0·37	0·381	0·393	0·404	0·416	0·428	0·44	0·451	0·462	0·474	0·485	0·497	0·507	0·515
Syria	0·367	0·376	0·387	0·398	0·409	0·421	0·432	0·443	0·454	0·465	0·476	0·486	0·497	0·508	0·521	0·535	0·548	0·56	0·572	0·583	0·594	0·601	0·605	0·606	0·607	0·608	0·61	0·613	0·616	0·619
Tunisia	0·434	0·444	0·455	0·466	0·476	0·487	0·498	0·508	0·518	0·528	0·538	0·548	0·556	0·565	0·574	0·582	0·591	0·599	0·607	0·614	0·622	0·628	0·634	0·64	0·646	0·651	0·657	0·662	0·667	0·672
Turkey	0·473	0·483	0·493	0·504	0·513	0·523	0·534	0·545	0·556	0·566	0·577	0·587	0·597	0·607	0·619	0·63	0·641	0·652	0·663	0·671	0·68	0·689	0·698	0·707	0·715	0·723	0·729	0·736	0·743	0·748
United Arab Emirates	0·621	0·637	0·653	0·669	0·685	0·7	0·714	0·727	0·74	0·751	0·762	0·773	0·783	0·792	0·801	0·81	0·819	0·829	0·838	0·846	0·853	0·859	0·863	0·868	0·87	0·872	0·874	0·876	0·879	0·88
Yemen	0·176	0·183	0·191	0·198	0·207	0·215	0·224	0·234	0·243	0·253	0·263	0·273	0·283	0·293	0·303	0·314	0·325	0·335	0·346	0·356	0·366	0·375	0·384	0·393	0·402	0·407	0·41	0·412	0·413	0·412
South Asia	0·313	0·319	0·325	0·331	0·337	0·344	0·351	0·357	0·364	0·371	0·378	0·385	0·391	0·398	0·405	0·413	0·421	0·43	0·438	0·447	0·456	0·465	0·475	0·485	0·495	0·505	0·515	0·525	0·535	0·543
South Asia	0·313	0·319	0·325	0·331	0·337	0·344	0·351	0·357	0·364	0·371	0·378	0·385	0·391	0·398	0·405	0·413	0·421	0·43	0·438	0·447	0·456	0·465	0·475	0·485	0·495	0·505	0·515	0·525	0·535	0·543
Bangladesh	0·267	0·275	0·283	0·29	0·297	0·304	0·311	0·317	0·324	0·33	0·336	0·343	0·349	0·355	0·362	0·369	0·376	0·384	0·392	0·4	0·408	0·416	0·425	0·433	0·441	0·449	0·457	0·466	0·475	0·483
Bhutan	0·228	0·232	0·237	0·243	0·251	0·258	0·266	0·275	0·282	0·29	0·298	0·306	0·314	0·322	0·33	0·338	0·347	0·356	0·365	0·375	0·384	0·394	0·403	0·411	0·419	0·426	0·434	0·442	0·449	0·455
India	0·327	0·333	0·339	0·345	0·351	0·358	0·364	0·371	0·378	0·386	0·393	0·4	0·407	0·414	0·421	0·429	0·437	0·446	0·455	0·463	0·473	0·483	0·493	0·504	0·515	0·526	0·537	0·547	0·558	0·566
Andhra Pradesh	0·287	0·294	0·301	0·309	0·316	0·324	0·332	0·34	0·348	0·356	0·364	0·373	0·381	0·388	0·395	0·404	0·413	0·422	0·431	0·441	0·451	0·461	0·471	0·481	0·492	0·504	0·515	0·527	0·537	0·546
Arunachal Pradesh	0·314	0·322	0·329	0·337	0·344	0·351	0·358	0·364	0·37	0·376	0·382	0·389	0·395	0·403	0·411	0·419	0·428	0·438	0·448	0·458	0·469	0·481	0·492	0·503	0·515	0·526	0·537	0·548	0·558	0·566
Assam	0·331	0·337	0·342	0·348	0·354	0·36	0·366	0·373	0·38	0·388	0·396	0·404	0·411	0·418	0·425	0·432	0·439	0·446	0·453	0·46	0·467	0·476	0·484	0·493	0·502	0·512	0·522	0·532	0·542	0·551
Bihar	0·261	0·264	0·266	0·267	0·267	0·267	0·267	0·267	0·269	0·272	0·276	0·28	0·285	0·291	0·296	0·303	0·31	0·318	0·326	0·335	0·346	0·356	0·367	0·378	0·389	0·4	0·412	0·423	0·434	0·444
Chhattisgarh	0·287	0·294	0·3	0·306	0·312	0·317	0·323	0·329	0·335	0·342	0·349	0·355	0·362	0·37	0·378	0·386	0·396	0·406	0·415	0·425	0·436	0·447	0·458	0·469	0·48	0·492	0·503	0·514	0·524	0·533
Delhi	0·497	0·503	0·51	0·517	0·524	0·53	0·536	0·542	0·547	0·553	0·56	0·566	0·573	0·579	0·586	0·593	0·6	0·608	0·617	0·625	0·634	0·643	0·652	0·661	0·671	0·682	0·692	0·701	0·71	0·717
Goa	0·502	0·509	0·516	0·524	0·531	0·539	0·547	0·555	0·563	0·571	0·579	0·586	0·592	0·599	0·607	0·614	0·622	0·631	0·639	0·647	0·655	0·663	0·669	0·676	0·682	0·689	0·697	0·704	0·711	0·717
Gujarat	0·364	0·369	0·375	0·382	0·389	0·397	0·405	0·414	0·423	0·431	0·438	0·445	0·45	0·456	0·462	0·47	0·478	0·487	0·496	0·505	0·515	0·524	0·534	0·545	0·557	0·569	0·58	0·591	0·601	0·609
Haryana	0·355	0·36	0·366	0·372	0·378	0·384	0·391	0·398	0·405	0·412	0·419	0·426	0·433	0·439	0·447	0·455	0·463	0·473	0·483	0·494	0·506	0·518	0·53	0·542	0·555	0·567	0·579	0·59	0·601	0·609
Himachal Pradesh	0·346	0·354	0·362	0·371	0·381	0·391	0·402	0·412	0·423	0·435	0·446	0·457	0·466	0·476	0·485	0·495	0·505	0·514	0·524	0·534	0·544	0·555	0·566	0·577	0·588	0·599	0·61	0·62	0·63	0·638
Jammu and Kashmir	0·343	0·348	0·353	0·36	0·366	0·373	0·381	0·389	0·398	0·408	0·418	0·429	0·439	0·449	0·459	0·469	0·479	0·489	0·498	0·508	0·518	0·529	0·539	0·549	0·558	0·568	0·578	0·587	0·596	0·605
Jharkhand	0·263	0·269	0·276	0·282	0·288	0·294	0·3	0·307	0·313	0·32	0·327	0·334	0·341	0·348	0·356	0·365	0·374	0·384	0·394	0·404	0·415	0·426	0·437	0·448	0·46	0·47	0·481	0·491	0·501	0·51
Karnataka	0·335	0·343	0·351	0·359	0·367	0·375	0·383	0·391	0·399	0·407	0·415	0·422	0·429	0·436	0·443	0·451	0·459	0·468	0·477	0·486	0·496	0·506	0·518	0·529	0·541	0·554	0·566	0·578	0·589	0·598
Kerala	0·43	0·437	0·444	0·451	0·458	0·465	0·472	0·48	0·489	0·498	0·507	0·515	0·522	0·53	0·539	0·547	0·556	0·564	0·571	0·579	0·587	0·595	0·604	0·614	0·625	0·636	0·646	0·655	0·664	0·671
Madhya Pradesh	0·296	0·301	0·305	0·309	0·313	0·317	0·321	0·326	0·33	0·336	0·342	0·348	0·353	0·359	0·364	0·37	0·376	0·382	0·389	0·397	0·405	0·415	0·425	0·436	0·448	0·46	0·472	0·484	0·495	0·505
Maharashtra	0·373	0·381	0·388	0·396	0·404	0·412	0·42	0·428	0·436	0·444	0·451	0·459	0·466	0·474	0·482	0·49	0·499	0·509	0·517	0·526	0·536	0·545	0·555	0·566	0·576	0·587	0·599	0·609	0·619	0·628
Manipur	0·389	0·396	0·403	0·409	0·416	0·422	0·429	0·436	0·444	0·452	0·459	0·467	0·474	0·48	0·487	0·494	0·501	0·508	0·514	0·521	0·527	0·534	0·541	0·548	0·556	0·564	0·572	0·58	0·588	0·595
Meghalaya	0·332	0·339	0·346	0·353	0·36	0·367	0·374	0·382	0·389	0·397	0·405	0·413	0·421	0·428	0·436	0·444	0·453	0·462	0·47	0·479	0·488	0·497	0·506	0·515	0·523	0·532	0·54	0·549	0·557	0·564
Mizoram	0·405	0·412	0·419	0·426	0·432	0·439	0·445	0·451	0·458	0·464	0·471	0·478	0·484	0·49	0·495	0·501	0·507	0·514	0·52	0·527	0·535	0·543	0·552	0·561	0·571	0·582	0·592	0·603	0·612	0·621
Nagaland	0·394	0·402	0·409	0·417	0·425	0·432	0·44	0·447	0·453	0·46	0·467	0·474	0·481	0·489	0·496	0·504	0·512	0·52	0·528	0·536	0·544	0·551	0·559	0·567	0·576	0·584	0·593	0·602	0·61	0·618
Odisha	0·295	0·301	0·307	0·312	0·318	0·325	0·332	0·339	0·346	0·355	0·362	0·37	0·377	0·385	0·394	0·403	0·413	0·423	0·433	0·442	0·452	0·462	0·472	0·482	0·493	0·504	0·514	0·524	0·534	0·542
Punjab	0·397	0·402	0·408	0·414	0·421	0·428	0·436	0·443	0·45	0·458	0·465	0·472	0·479	0·486	0·493	0·501	0·51	0·518	0·527	0·535	0·544	0·553	0·561	0·57	0·58	0·589	0·598	0·607	0·615	0·623
Rajasthan	0·261	0·267	0·273	0·279	0·286	0·293	0·3	0·307	0·314	0·322	0·33	0·337	0·344	0·351	0·358	0·366	0·374	0·384	0·393	0·403	0·415	0·427	0·439	0·451	0·464	0·476	0·489	0·501	0·512	0·521
Sikkim	0·336	0·344	0·352	0·36	0·369	0·378	0·387	0·396	0·406	0·416	0·425	0·435	0·445	0·454	0·463	0·473	0·484	0·495	0·507	0·52	0·535	0·55	0·564	0·577	0·589	0·601	0·612	0·622	0·632	0·64
Tamil Nadu	0·374	0·38	0·386	0·392	0·399	0·406	0·414	0·421	0·429	0·437	0·445	0·453	0·46	0·468	0·476	0·485	0·493	0·502	0·511	0·519	0·528	0·538	0·548	0·558	0·57	0·582	0·593	0·603	0·613	0·621
Telangana	0·297	0·304	0·311	0·319	0·326	0·334	0·342	0·35	0·358	0·366	0·374	0·383	0·39	0·398	0·407	0·415	0·425	0·435	0·445	0·456	0·467	0·479	0·491	0·503	0·516	0·528	0·54	0·552	0·563	0·572
Tripura	0·344	0·35	0·356	0·363	0·369	0·377	0·384	0·392	0·4	0·409	0·417	0·425	0·433	0·44	0·446	0·453	0·46	0·466	0·473	0·479	0·486	0·493	0·499	0·507	0·515	0·523	0·532	0·541	0·549	0·557
Union Territories other than Delhi	0·423	0·43	0·437	0·444	0·451	0·459	0·467	0·476	0·485	0·495	0·504	0·514	0·523	0·532	0·541	0·55	0·559	0·568	0·577	0·585	0·593	0·601	0·609	0·617	0·625	0·633	0·641	0·649	0·657	0·664
Uttar Pradesh	0·274	0·279	0·284	0·29	0·295	0·301	0·307	0·314	0·32	0·327	0·334	0·341	0·348	0·355	0·362	0·37	0·379	0·388	0·397	0·407	0·417	0·428	0·439	0·45	0·461	0·472	0·483	0·494	0·505	0·513
Uttarakhand	0·309	0·315	0·321	0·328	0·334	0·341	0·349	0·357	0·365	0·375	0·385	0·396	0·408	0·419	0·431	0·444	0·458	0·472	0·487	0·501	0·516	0·531	0·545	0·559	0·572	0·585	0·597	0·608	0·619	0·628
West Bengal	0·342	0·347	0·352	0·358	0·363	0·369	0·375	0·382	0·388	0·395	0·402	0·409	0·415	0·421	0·427	0·434	0·441	0·448	0·455	0·462	0·469	0·476	0·484	0·491	0·5	0·509	0·518	0·528	0·537	0·545
Nepal	0·198	0·203	0·208	0·215	0·221	0·228	0·236	0·244	0·251	0·259	0·267	0·276	0·284	0·291	0·299	0·307	0·315	0·322	0·33	0·339	0·347	0·356	0·365	0·373	0·382	0·391	0·399	0·408	0·416	0·422
Pakistan	0·247	0·253	0·259	0·265	0·271	0·277	0·283	0·289	0·295	0·301	0·307	0·313	0·32	0·326	0·333	0·34	0·347	0·355	0·363	0·371	0·379	0·387	0·394	0·402	0·41	0·418	0·426	0·434	0·442	0·449
Southeast Asia, east Asia, and Oceania	0·452	0·46	0·468	0·476	0·485	0·494	0·503	0·511	0·519	0·526	0·533	0·541	0·548	0·556	0·563	0·571	0·58	0·589	0·597	0·605	0·614	0·622	0·629	0·636	0·643	0·647	0·651	0·659	0·667	0·673
East Asia	0·447	0·456	0·464	0·473	0·483	0·492	0·502	0·511	0·52	0·528	0·537	0·545	0·554	0·562	0·571	0·58	0·59	0·6	0·609	0·618	0·628	0·637	0·644	0·652	0·66	0·662	0·665	0·675	0·684	0·691
China	0·433	0·441	0·45	0·459	0·469	0·479	0·489	0·499	0·508	0·516	0·525	0·534	0·543	0·552	0·561	0·571	0·581	0·591	0·601	0·611	0·621	0·631	0·638	0·646	0·654	0·657	0·659	0·669	0·679	0·686
North Korea	0·431	0·436	0·439	0·442	0·445	0·447	0·45	0·451	0·452	0·455	0·458	0·463	0·467	0·473	0·478	0·485	0·49	0·496	0·502	0·507	0·513	0·518	0·523	0·528	0·534	0·538	0·543	0·548	0·553	0·558
Taiwan (province of China)	0·667	0·678	0·685	0·694	0·702	0·711	0·719	0·731	0·743	0·747	0·754	0·763	0·772	0·779	0·787	0·795	0·802	0·81	0·817	0·824	0·83	0·833	0·838	0·843	0·848	0·852	0·856	0·86	0·865	0·868
Oceania	0·383	0·385	0·388	0·391	0·394	0·397	0·4	0·403	0·405	0·408	0·41	0·412	0·413	0·414	0·416	0·417	0·419	0·421	0·422	0·424	0·426	0·428	0·431	0·433	0·436	0·44	0·443	0·446	0·449	0·452
American Samoa	0·606	0·609	0·613	0·616	0·619	0·623	0·627	0·63	0·634	0·637	0·641	0·645	0·649	0·652	0·656	0·66	0·663	0·667	0·671	0·674	0·678	0·682	0·686	0·69	0·694	0·698	0·702	0·706	0·709	0·712
Cook Islands	0·625	0·631	0·637	0·643	0·649	0·654	0·659	0·663	0·667	0·671	0·676	0·682	0·687	0·692	0·697	0·701	0·706	0·711	0·715	0·719	0·722	0·726	0·73	0·734	0·739	0·744	0·75	0·755	0·76	0·764
Fiji	0·527	0·532	0·538	0·544	0·55	0·555	0·561	0·567	0·572	0·577	0·582	0·587	0·592	0·596	0·601	0·605	0·609	0·612	0·616	0·619	0·622	0·626	0·63	0·635	0·639	0·644	0·649	0·654	0·659	0·664
Guam	0·693	0·688	0·684	0·684	0·688	0·695	0·704	0·715	0·728	0·741	0·753	0·762	0·767	0·768	0·77	0·771	0·773	0·775	0·778	0·782	0·785	0·789	0·792	0·795	0·797	0·799	0·803	0·807	0·81	0·813
Kiribati	0·425	0·427	0·429	0·432	0·435	0·438	0·441	0·444	0·447	0·451	0·455	0·459	0·463	0·468	0·472	0·476	0·48	0·484	0·487	0·49	0·492	0·495	0·497	0·501	0·504	0·509	0·514	0·518	0·523	0·527
Marshall Islands	0·398	0·404	0·41	0·416	0·423	0·43	0·435	0·439	0·442	0·446	0·45	0·454	0·458	0·463	0·467	0·472	0·477	0·482	0·487	0·493	0·498	0·504	0·509	0·515	0·52	0·525	0·531	0·536	0·541	0·544
Federated States of Micronesia	0·447	0·453	0·459	0·465	0·471	0·478	0·483	0·488	0·492	0·497	0·502	0·507	0·511	0·516	0·521	0·525	0·53	0·534	0·538	0·542	0·546	0·55	0·554	0·558	0·561	0·565	0·569	0·573	0·577	0·58
Nauru	0·499	0·501	0·503	0·504	0·504	0·505	0·505	0·504	0·504	0·503	0·503	0·503	0·503	0·503	0·504	0·506	0·509	0·51	0·515	0·521	0·529	0·538	0·547	0·559	0·573	0·585	0·595	0·605	0·613	0·618
Niue	0·566	0·571	0·576	0·581	0·586	0·591	0·595	0·6	0·605	0·609	0·614	0·618	0·622	0·626	0·631	0·638	0·645	0·651	0·657	0·663	0·669	0·675	0·681	0·685	0·69	0·695	0·699	0·703	0·707	0·711
Northern Mariana Islands	0·692	0·698	0·704	0·709	0·714	0·718	0·722	0·725	0·727	0·731	0·738	0·744	0·748	0·75	0·751	0·752	0·752	0·753	0·753	0·752	0·751	0·75	0·75	0·75	0·75	0·751	0·756	0·761	0·767	0·771
Palau	0·621	0·629	0·636	0·642	0·647	0·652	0·658	0·663	0·668	0·672	0·676	0·68	0·683	0·686	0·69	0·693	0·697	0·7	0·703	0·705	0·707	0·71	0·714	0·717	0·72	0·725	0·729	0·732	0·735	0·738
Papua New Guinea	0·292	0·295	0·299	0·304	0·309	0·313	0·317	0·32	0·323	0·326	0·329	0·331	0·333	0·335	0·337	0·339	0·342	0·345	0·348	0·352	0·356	0·36	0·363	0·367	0·372	0·377	0·382	0·386	0·391	0·394
Samoa	0·531	0·535	0·539	0·543	0·546	0·55	0·555	0·559	0·563	0·567	0·571	0·576	0·581	0·586	0·59	0·595	0·599	0·603	0·607	0·61	0·612	0·615	0·618	0·62	0·623	0·626	0·629	0·633	0·637	0·641
Solomon Islands	0·279	0·283	0·288	0·294	0·3	0·306	0·312	0·317	0·321	0·326	0·328	0·33	0·331	0·332	0·333	0·336	0·339	0·343	0·348	0·353	0·358	0·364	0·371	0·377	0·382	0·387	0·393	0·398	0·403	0·407
Tokelau	0·427	0·432	0·438	0·444	0·45	0·456	0·463	0·47	0·477	0·484	0·491	0·498	0·504	0·511	0·519	0·526	0·534	0·542	0·55	0·557	0·565	0·573	0·58	0·588	0·595	0·602	0·608	0·615	0·621	0·626
Tonga	0·51	0·517	0·522	0·527	0·533	0·538	0·543	0·547	0·551	0·555	0·559	0·563	0·568	0·572	0·576	0·58	0·583	0·587	0·59	0·594	0·598	0·602	0·606	0·61	0·614	0·618	0·622	0·627	0·632	0·636
Tuvalu	0·426	0·434	0·442	0·45	0·458	0·465	0·47	0·476	0·483	0·49	0·496	0·502	0·509	0·514	0·519	0·523	0·527	0·531	0·537	0·541	0·545	0·549	0·553	0·558	0·562	0·567	0·573	0·579	0·584	0·589
Vanuatu	0·361	0·365	0·369	0·373	0·377	0·381	0·386	0·39	0·395	0·399	0·403	0·407	0·41	0·413	0·417	0·42	0·425	0·43	0·435	0·44	0·446	0·451	0·455	0·46	0·464	0·468	0·473	0·477	0·481	0·485
Southeast Asia	0·455	0·463	0·471	0·479	0·488	0·496	0·504	0·512	0·517	0·523	0·528	0·533	0·538	0·543	0·548	0·554	0·559	0·566	0·572	0·578	0·585	0·592	0·599	0·606	0·612	0·619	0·626	0·632	0·639	0·644
Cambodia	0·266	0·272	0·277	0·282	0·286	0·291	0·296	0·301	0·307	0·313	0·321	0·328	0·337	0·345	0·354	0·363	0·373	0·382	0·391	0·399	0·406	0·413	0·421	0·428	0·435	0·442	0·449	0·456	0·463	0·469
Indonesia	0·452	0·462	0·472	0·481	0·49	0·499	0·508	0·516	0·522	0·527	0·533	0·537	0·542	0·547	0·552	0·558	0·564	0·571	0·578	0·585	0·593	0·601	0·609	0·617	0·625	0·633	0·64	0·647	0·654	0·66
Aceh	0·482	0·493	0·502	0·512	0·521	0·529	0·537	0·544	0·55	0·554	0·559	0·563	0·567	0·572	0·577	0·582	0·587	0·592	0·598	0·603	0·608	0·614	0·62	0·626	0·633	0·64	0·646	0·653	0·66	0·666
Bali	0·443	0·454	0·465	0·475	0·485	0·495	0·505	0·513	0·519	0·524	0·528	0·532	0·537	0·541	0·545	0·55	0·555	0·561	0·567	0·574	0·582	0·59	0·598	0·606	0·613	0·621	0·628	0·635	0·642	0·648
Bangka-Belitung Islands	0·442	0·452	0·461	0·471	0·48	0·489	0·498	0·506	0·512	0·517	0·522	0·527	0·532	0·537	0·542	0·547	0·553	0·56	0·567	0·574	0·581	0·589	0·597	0·604	0·612	0·619	0·627	0·634	0·641	0·647
Banten	0·452	0·462	0·471	0·481	0·49	0·499	0·508	0·516	0·521	0·526	0·529	0·533	0·536	0·539	0·542	0·546	0·551	0·556	0·562	0·568	0·575	0·583	0·591	0·599	0·606	0·614	0·621	0·629	0·636	0·642
Bengkulu	0·405	0·416	0·427	0·437	0·448	0·458	0·468	0·477	0·483	0·488	0·492	0·496	0·5	0·505	0·509	0·515	0·52	0·527	0·534	0·542	0·55	0·558	0·567	0·575	0·583	0·591	0·598	0·606	0·613	0·619
Gorontalo	0·367	0·376	0·385	0·393	0·402	0·411	0·419	0·427	0·432	0·436	0·439	0·442	0·446	0·449	0·453	0·458	0·464	0·47	0·478	0·487	0·496	0·505	0·514	0·523	0·532	0·54	0·548	0·555	0·563	0·569
Jakarta	0·581	0·591	0·602	0·612	0·623	0·633	0·643	0·652	0·659	0·665	0·671	0·677	0·683	0·69	0·696	0·703	0·709	0·716	0·724	0·732	0·74	0·748	0·756	0·764	0·771	0·778	0·785	0·791	0·797	0·802
Jambi	0·422	0·432	0·442	0·452	0·462	0·471	0·481	0·489	0·495	0·5	0·505	0·51	0·515	0·52	0·525	0·531	0·538	0·545	0·554	0·562	0·571	0·581	0·59	0·598	0·606	0·614	0·621	0·628	0·635	0·641
West Java	0·446	0·456	0·466	0·475	0·485	0·494	0·503	0·511	0·517	0·521	0·526	0·53	0·534	0·538	0·542	0·547	0·553	0·559	0·567	0·574	0·582	0·59	0·598	0·606	0·614	0·621	0·629	0·636	0·643	0·648
Central Java	0·415	0·424	0·433	0·442	0·45	0·459	0·467	0·474	0·48	0·484	0·488	0·492	0·496	0·501	0·506	0·511	0·518	0·524	0·532	0·539	0·547	0·555	0·563	0·571	0·579	0·586	0·593	0·6	0·607	0·613
East Java	0·436	0·445	0·454	0·463	0·472	0·481	0·49	0·498	0·504	0·51	0·515	0·521	0·526	0·532	0·538	0·544	0·55	0·557	0·565	0·572	0·58	0·589	0·597	0·604	0·612	0·619	0·626	0·633	0·64	0·646
West Kalimantan	0·389	0·4	0·41	0·421	0·431	0·441	0·452	0·461	0·468	0·473	0·479	0·483	0·488	0·492	0·497	0·502	0·507	0·513	0·519	0·525	0·532	0·54	0·548	0·555	0·563	0·57	0·578	0·585	0·592	0·598
South Kalimantan	0·438	0·447	0·457	0·467	0·477	0·486	0·495	0·504	0·51	0·515	0·519	0·524	0·528	0·532	0·537	0·541	0·546	0·552	0·558	0·564	0·571	0·579	0·586	0·594	0·601	0·609	0·616	0·623	0·63	0·636
Central Kalimantan	0·452	0·462	0·472	0·483	0·495	0·505	0·516	0·525	0·531	0·536	0·54	0·544	0·548	0·551	0·555	0·559	0·564	0·569	0·575	0·582	0·589	0·596	0·604	0·611	0·619	0·626	0·633	0·64	0·647	0·653
East Kalimantan	0·563	0·573	0·582	0·59	0·598	0·606	0·614	0·621	0·627	0·632	0·637	0·642	0·646	0·651	0·656	0·662	0·667	0·674	0·681	0·688	0·695	0·703	0·711	0·719	0·727	0·734	0·742	0·749	0·756	0·762
North Kalimantan	0·513	0·523	0·533	0·542	0·551	0·561	0·57	0·58	0·588	0·595	0·603	0·611	0·619	0·627	0·635	0·644	0·653	0·662	0·671	0·68	0·689	0·698	0·707	0·715	0·723	0·731	0·738	0·745	0·752	0·758
Riau Islands	0·561	0·57	0·58	0·589	0·598	0·607	0·615	0·623	0·629	0·634	0·638	0·641	0·644	0·646	0·649	0·652	0·656	0·661	0·667	0·673	0·68	0·687	0·695	0·702	0·709	0·717	0·724	0·731	0·737	0·742
Lampung	0·394	0·404	0·414	0·423	0·433	0·444	0·454	0·463	0·47	0·476	0·482	0·487	0·492	0·497	0·502	0·507	0·513	0·52	0·528	0·537	0·546	0·556	0·565	0·574	0·582	0·59	0·598	0·605	0·612	0·617
Maluku	0·479	0·487	0·495	0·503	0·51	0·516	0·522	0·526	0·527	0·526	0·523	0·519	0·514	0·509	0·503	0·498	0·494	0·493	0·494	0·498	0·503	0·511	0·519	0·527	0·535	0·544	0·552	0·56	0·568	0·575
North Maluku	0·376	0·385	0·394	0·403	0·412	0·422	0·432	0·441	0·446	0·45	0·454	0·457	0·459	0·461	0·462	0·463	0·465	0·468	0·473	0·479	0·487	0·495	0·504	0·513	0·522	0·531	0·539	0·547	0·555	0·562
West Nusa Tenggara	0·341	0·351	0·361	0·372	0·383	0·394	0·405	0·415	0·423	0·43	0·437	0·445	0·452	0·459	0·467	0·474	0·482	0·489	0·497	0·505	0·513	0·522	0·53	0·538	0·546	0·554	0·561	0·568	0·575	0·582
East Nusa Tenggara	0·367	0·374	0·382	0·39	0·398	0·406	0·414	0·421	0·424	0·427	0·429	0·432	0·435	0·437	0·441	0·444	0·448	0·453	0·459	0·465	0·472	0·48	0·488	0·497	0·505	0·513	0·521	0·529	0·537	0·543
Papua	0·453	0·461	0·47	0·478	0·486	0·495	0·503	0·51	0·516	0·52	0·524	0·527	0·529	0·531	0·533	0·536	0·539	0·542	0·547	0·553	0·559	0·567	0·575	0·583	0·591	0·599	0·607	0·615	0·623	0·629
West Papua	0·443	0·452	0·461	0·47	0·478	0·487	0·495	0·503	0·508	0·512	0·516	0·52	0·524	0·528	0·532	0·537	0·543	0·55	0·559	0·569	0·58	0·593	0·604	0·615	0·625	0·634	0·643	0·652	0·66	0·666
Riau	0·524	0·535	0·545	0·555	0·564	0·572	0·58	0·588	0·594	0·599	0·605	0·611	0·617	0·622	0·627	0·632	0·637	0·643	0·649	0·656	0·663	0·67	0·678	0·685	0·693	0·7	0·707	0·714	0·721	0·727
West Sulawesi	0·371	0·38	0·388	0·397	0·405	0·413	0·421	0·428	0·433	0·437	0·44	0·444	0·447	0·45	0·454	0·458	0·463	0·47	0·477	0·486	0·495	0·505	0·515	0·524	0·533	0·542	0·55	0·558	0·566	0·573
South Sulawesi	0·409	0·419	0·428	0·437	0·446	0·455	0·465	0·473	0·479	0·485	0·49	0·495	0·501	0·506	0·511	0·517	0·523	0·53	0·537	0·545	0·554	0·564	0·573	0·581	0·59	0·598	0·605	0·613	0·62	0·626
Central Sulawesi	0·406	0·416	0·425	0·435	0·445	0·455	0·465	0·475	0·482	0·488	0·494	0·499	0·505	0·51	0·516	0·521	0·527	0·534	0·541	0·549	0·557	0·566	0·575	0·583	0·592	0·6	0·608	0·615	0·622	0·628
Southeast Sulawesi	0·373	0·384	0·394	0·404	0·414	0·423	0·433	0·443	0·45	0·456	0·462	0·468	0·473	0·479	0·486	0·493	0·5	0·508	0·517	0·526	0·536	0·546	0·556	0·565	0·574	0·582	0·59	0·598	0·606	0·612
North Sulawesi	0·473	0·483	0·492	0·501	0·51	0·52	0·529	0·538	0·543	0·548	0·551	0·555	0·558	0·561	0·565	0·568	0·573	0·578	0·585	0·592	0·6	0·608	0·615	0·623	0·631	0·638	0·645	0·652	0·658	0·664
West Sumatra	0·462	0·47	0·479	0·488	0·497	0·506	0·515	0·523	0·529	0·534	0·539	0·543	0·548	0·553	0·558	0·563	0·57	0·576	0·584	0·592	0·6	0·609	0·617	0·625	0·633	0·641	0·648	0·655	0·662	0·668
South Sumatra	0·455	0·464	0·473	0·482	0·49	0·498	0·507	0·514	0·52	0·525	0·529	0·534	0·538	0·543	0·548	0·554	0·56	0·567	0·574	0·582	0·59	0·598	0·607	0·615	0·622	0·63	0·637	0·644	0·651	0·657
North Sumatra	0·468	0·477	0·486	0·495	0·504	0·512	0·521	0·529	0·534	0·539	0·544	0·548	0·553	0·558	0·563	0·569	0·575	0·582	0·589	0·597	0·605	0·614	0·623	0·631	0·639	0·647	0·654	0·662	0·669	0·675
Yogyakarta	0·467	0·477	0·487	0·498	0·509	0·52	0·53	0·539	0·545	0·55	0·555	0·559	0·563	0·567	0·571	0·576	0·582	0·587	0·594	0·601	0·608	0·616	0·624	0·631	0·639	0·646	0·652	0·659	0·665	0·671
Laos	0·268	0·274	0·279	0·285	0·29	0·296	0·302	0·309	0·315	0·322	0·329	0·336	0·344	0·351	0·359	0·367	0·376	0·385	0·394	0·403	0·413	0·422	0·431	0·441	0·45	0·458	0·467	0·475	0·483	0·49
Malaysia	0·542	0·548	0·554	0·562	0·572	0·581	0·59	0·6	0·611	0·622	0·63	0·638	0·646	0·652	0·659	0·665	0·671	0·677	0·681	0·687	0·693	0·698	0·704	0·71	0·716	0·722	0·726	0·728	0·732	0·737
Maldives	0·303	0·314	0·324	0·336	0·347	0·359	0·37	0·382	0·394	0·406	0·417	0·427	0·437	0·447	0·456	0·464	0·473	0·481	0·49	0·497	0·504	0·511	0·518	0·525	0·532	0·538	0·544	0·551	0·557	0·562
Mauritius	0·527	0·532	0·535	0·543	0·556	0·565	0·57	0·576	0·583	0·588	0·593	0·598	0·603	0·608	0·614	0·621	0·627	0·633	0·64	0·646	0·652	0·658	0·665	0·673	0·68	0·686	0·69	0·695	0·7	0·705
Myanmar	0·284	0·287	0·29	0·295	0·3	0·306	0·313	0·32	0·327	0·335	0·344	0·353	0·363	0·373	0·384	0·395	0·406	0·417	0·427	0·437	0·446	0·455	0·464	0·473	0·482	0·49	0·498	0·506	0·514	0·521
Philippines	0·497	0·501	0·505	0·509	0·513	0·518	0·522	0·526	0·529	0·532	0·534	0·537	0·54	0·542	0·545	0·547	0·55	0·554	0·558	0·562	0·567	0·572	0·577	0·583	0·589	0·596	0·603	0·61	0·617	0·623
Seychelles	0·567	0·576	0·584	0·592	0·6	0·607	0·614	0·621	0·629	0·636	0·642	0·647	0·652	0·656	0·659	0·662	0·666	0·67	0·673	0·676	0·679	0·683	0·687	0·691	0·696	0·702	0·707	0·713	0·719	0·724
Sri Lanka	0·504	0·511	0·518	0·525	0·532	0·539	0·547	0·554	0·561	0·567	0·573	0·578	0·582	0·587	0·592	0·597	0·602	0·609	0·615	0·621	0·628	0·636	0·644	0·651	0·658	0·666	0·672	0·678	0·684	0·69
Thailand	0·508	0·518	0·527	0·536	0·544	0·553	0·562	0·569	0·574	0·578	0·583	0·589	0·594	0·599	0·605	0·61	0·616	0·623	0·629	0·633	0·638	0·643	0·649	0·655	0·66	0·666	0·671	0·676	0·682	0·687
Timor-Leste	0·274	0·28	0·286	0·293	0·3	0·308	0·317	0·326	0·334	0·339	0·345	0·352	0·358	0·364	0·374	0·388	0·406	0·421	0·436	0·448	0·458	0·469	0·48	0·488	0·493	0·498	0·503	0·508	0·511	0·514
Vietnam	0·39	0·397	0·404	0·412	0·42	0·429	0·438	0·447	0·455	0·463	0·471	0·478	0·486	0·493	0·501	0·509	0·517	0·525	0·533	0·541	0·549	0·558	0·566	0·573	0·581	0·589	0·596	0·604	0·611	0·617
Sub-Saharan Africa	0·291	0·295	0·299	0·302	0·306	0·309	0·314	0·318	0·322	0·327	0·331	0·336	0·341	0·346	0·352	0·359	0·365	0·372	0·38	0·386	0·394	0·401	0·409	0·416	0·423	0·431	0·438	0·445	0·452	0·456
Central sub-Saharan Africa	0·269	0·273	0·276	0·279	0·281	0·283	0·286	0·289	0·292	0·295	0·298	0·302	0·308	0·313	0·32	0·328	0·336	0·346	0·357	0·367	0·378	0·389	0·4	0·412	0·423	0·434	0·445	0·454	0·463	0·47
Angola	0·238	0·243	0·247	0·25	0·253	0·257	0·262	0·267	0·273	0·278	0·283	0·289	0·296	0·303	0·311	0·319	0·33	0·341	0·353	0·364	0·376	0·387	0·398	0·41	0·421	0·432	0·443	0·454	0·463	0·47

Table S1 Socio-demographic index values for all estimated GBD 2019 locations, 1990-2019

Location	1990	1991	1992	1993	1994	1995	1996	1997	1998	1999	2000	2001	2002	2003	2004	2005	2006	2007	2008	2009	2010	2011	2012	2013	2014	2015	2016	2017	2018	2019
Central African Republic	0·186	0·19	0·193	0·196	0·199	0·202	0·205	0·208	0·211	0·215	0·218	0·221	0·225	0·227	0·23	0·233	0·236	0·24	0·244	0·248	0·253	0·258	0·263	0·263	0·263	0·264	0·266	0·268	0·271	0·274
Congo (Brazzaville)	0·364	0·373	0·381	0·389	0·396	0·403	0·41	0·416	0·421	0·426	0·431	0·437	0·442	0·447	0·452	0·458	0·464	0·469	0·475	0·482	0·491	0·5	0·509	0·519	0·528	0·538	0·547	0·556	0·563	0·568
DR Congo	0·26	0·262	0·263	0·263	0·262	0·261	0·259	0·256	0·253	0·25	0·246	0·242	0·239	0·238	0·239	0·242	0·244	0·247	0·251	0·257	0·266	0·277	0·289	0·305	0·321	0·336	0·35	0·362	0·374	0·382
Equatorial Guinea	0·208	0·214	0·222	0·23	0·24	0·252	0·268	0·295	0·318	0·341	0·364	0·391	0·415	0·438	0·46	0·482	0·502	0·522	0·543	0·561	0·578	0·594	0·611	0·626	0·64	0·652	0·663	0·673	0·681	0·685
Gabon	0·388	0·399	0·409	0·42	0·431	0·442	0·453	0·463	0·474	0·483	0·493	0·502	0·51	0·519	0·528	0·537	0·546	0·554	0·563	0·57	0·579	0·587	0·596	0·605	0·614	0·623	0·632	0·641	0·649	0·656
Eastern sub-Saharan Africa	0·235	0·239	0·242	0·245	0·249	0·252	0·257	0·261	0·265	0·27	0·275	0·28	0·285	0·29	0·295	0·301	0·307	0·314	0·321	0·328	0·336	0·343	0·351	0·359	0·367	0·375	0·383	0·391	0·399	0·405
Burundi	0·198	0·201	0·204	0·207	0·209	0·21	0·21	0·211	0·212	0·213	0·213	0·214	0·216	0·218	0·22	0·223	0·226	0·23	0·234	0·238	0·243	0·248	0·254	0·26	0·266	0·27	0·274	0·278	0·282	0·284
Comoros	0·274	0·281	0·288	0·296	0·302	0·309	0·315	0·322	0·328	0·334	0·34	0·346	0·353	0·359	0·365	0·371	0·378	0·384	0·39	0·396	0·401	0·407	0·413	0·419	0·426	0·432	0·438	0·444	0·45	0·455
Djibouti	0·275	0·277	0·28	0·285	0·289	0·293	0·297	0·302	0·306	0·311	0·317	0·322	0·327	0·333	0·339	0·345	0·353	0·36	0·368	0·376	0·384	0·392	0·4	0·408	0·416	0·425	0·434	0·443	0·452	0·459
Eritrea	0·198	0·203	0·21	0·218	0·228	0·238	0·248	0·258	0·267	0·275	0·283	0·289	0·296	0·302	0·307	0·312	0·317	0·322	0·326	0·33	0·334	0·34	0·346	0·353	0·36	0·367	0·375	0·382	0·39	0·396
Ethiopia	0·144	0·146	0·147	0·149	0·151	0·153	0·157	0·161	0·164	0·167	0·171	0·176	0·18	0·183	0·189	0·195	0·203	0·213	0·223	0·233	0·244	0·256	0·268	0·279	0·291	0·302	0·313	0·324	0·334	0·343
Kenya	0·333	0·341	0·348	0·354	0·36	0·366	0·372	0·378	0·383	0·388	0·392	0·397	0·401	0·404	0·409	0·413	0·418	0·424	0·429	0·435	0·441	0·448	0·455	0·463	0·47	0·478	0·486	0·494	0·502	0·508
Baringo	0·248	0·257	0·266	0·274	0·283	0·291	0·299	0·307	0·315	0·322	0·329	0·335	0·341	0·348	0·353	0·359	0·365	0·372	0·378	0·384	0·392	0·4	0·408	0·417	0·426	0·436	0·447	0·457	0·468	0·476
Bomet	0·27	0·28	0·289	0·298	0·307	0·315	0·324	0·332	0·34	0·347	0·355	0·362	0·369	0·376	0·383	0·39	0·398	0·407	0·415	0·424	0·433	0·443	0·454	0·465	0·476	0·487	0·499	0·511	0·522	0·531
Bungoma	0·281	0·289	0·296	0·302	0·308	0·314	0·32	0·325	0·329	0·334	0·338	0·342	0·346	0·35	0·355	0·36	0·366	0·373	0·38	0·387	0·395	0·404	0·413	0·423	0·433	0·444	0·454	0·465	0·475	0·483
Busia	0·256	0·263	0·269	0·275	0·282	0·288	0·294	0·299	0·305	0·31	0·315	0·32	0·325	0·329	0·334	0·339	0·345	0·351	0·356	0·363	0·37	0·378	0·387	0·396	0·405	0·415	0·425	0·435	0·445	0·453
Elgeyo Marakwet	0·264	0·273	0·28	0·288	0·295	0·302	0·308	0·315	0·321	0·327	0·333	0·339	0·344	0·35	0·356	0·363	0·371	0·379	0·387	0·396	0·406	0·416	0·426	0·437	0·449	0·46	0·471	0·483	0·494	0·503
Embu	0·377	0·385	0·392	0·398	0·404	0·409	0·415	0·419	0·424	0·429	0·433	0·437	0·44	0·444	0·447	0·451	0·456	0·461	0·466	0·471	0·478	0·485	0·492	0·5	0·508	0·516	0·525	0·534	0·542	0·55
Garissa	0·187	0·192	0·195	0·199	0·203	0·207	0·211	0·215	0·218	0·221	0·224	0·227	0·23	0·233	0·235	0·239	0·242	0·246	0·25	0·255	0·259	0·264	0·27	0·275	0·281	0·287	0·293	0·299	0·305	0·31
Homa Bay	0·209	0·215	0·222	0·229	0·236	0·243	0·25	0·257	0·264	0·271	0·277	0·283	0·289	0·295	0·301	0·307	0·314	0·322	0·33	0·338	0·348	0·358	0·369	0·381	0·393	0·405	0·418	0·431	0·443	0·452
Isiolo	0·241	0·246	0·252	0·257	0·263	0·268	0·273	0·278	0·283	0·289	0·293	0·298	0·303	0·307	0·311	0·315	0·32	0·324	0·329	0·333	0·339	0·346	0·353	0·361	0·369	0·378	0·387	0·396	0·405	0·413
Kajiado	0·359	0·367	0·374	0·382	0·388	0·395	0·401	0·407	0·413	0·419	0·423	0·428	0·433	0·437	0·441	0·446	0·452	0·459	0·465	0·472	0·48	0·489	0·498	0·507	0·516	0·526	0·536	0·545	0·555	0·563
Kakamega	0·281	0·288	0·295	0·301	0·307	0·313	0·319	0·325	0·33	0·336	0·341	0·347	0·351	0·356	0·361	0·367	0·373	0·379	0·386	0·393	0·401	0·41	0·419	0·429	0·439	0·449	0·46	0·47	0·481	0·489
Kericho	0·243	0·252	0·261	0·269	0·278	0·286	0·294	0·302	0·31	0·318	0·326	0·333	0·341	0·348	0·356	0·363	0·372	0·381	0·389	0·398	0·408	0·419	0·43	0·441	0·453	0·465	0·477	0·489	0·501	0·509
Kiambu	0·425	0·434	0·441	0·447	0·453	0·459	0·465	0·47	0·474	0·479	0·483	0·488	0·492	0·496	0·5	0·505	0·511	0·518	0·524	0·531	0·538	0·546	0·554	0·562	0·57	0·578	0·586	0·594	0·602	0·609
Kilifi	0·292	0·299	0·306	0·312	0·319	0·325	0·331	0·337	0·343	0·348	0·353	0·357	0·362	0·366	0·37	0·375	0·38	0·386	0·392	0·399	0·407	0·415	0·424	0·433	0·443	0·453	0·463	0·474	0·484	0·492
Kirinyaga	0·372	0·38	0·387	0·393	0·399	0·404	0·41	0·414	0·419	0·423	0·428	0·432	0·436	0·44	0·444	0·449	0·454	0·46	0·465	0·471	0·477	0·484	0·491	0·499	0·506	0·514	0·523	0·531	0·539	0·546
Kisii	0·343	0·353	0·362	0·369	0·377	0·384	0·391	0·397	0·402	0·407	0·412	0·416	0·42	0·424	0·428	0·433	0·438	0·445	0·452	0·459	0·468	0·477	0·487	0·497	0·507	0·518	0·528	0·539	0·549	0·557
Kisumu	0·333	0·341	0·349	0·356	0·363	0·37	0·376	0·382	0·388	0·394	0·4	0·405	0·41	0·415	0·42	0·426	0·432	0·44	0·448	0·456	0·465	0·475	0·485	0·496	0·507	0·518	0·529	0·54	0·551	0·559
Kitui	0·266	0·273	0·28	0·286	0·293	0·299	0·305	0·311	0·317	0·322	0·327	0·333	0·338	0·342	0·348	0·353	0·359	0·366	0·372	0·38	0·388	0·396	0·406	0·415	0·425	0·435	0·446	0·456	0·466	0·474
Kwale	0·284	0·292	0·298	0·305	0·311	0·318	0·324	0·329	0·334	0·339	0·343	0·347	0·351	0·354	0·358	0·362	0·367	0·372	0·377	0·383	0·39	0·397	0·405	0·414	0·423	0·432	0·442	0·452	0·461	0·469
Laikipia	0·344	0·352	0·358	0·364	0·37	0·376	0·382	0·387	0·393	0·399	0·404	0·41	0·416	0·423	0·43	0·437	0·445	0·453	0·462	0·47	0·479	0·489	0·498	0·508	0·518	0·528	0·538	0·549	0·559	0·567
Lamu	0·31	0·317	0·323	0·329	0·335	0·341	0·347	0·352	0·356	0·361	0·365	0·369	0·373	0·377	0·382	0·386	0·391	0·397	0·402	0·408	0·414	0·421	0·429	0·436	0·444	0·453	0·462	0·471	0·48	0·488
Machakos	0·351	0·359	0·367	0·373	0·38	0·386	0·393	0·398	0·403	0·409	0·413	0·418	0·423	0·427	0·432	0·437	0·443	0·45	0·457	0·464	0·472	0·48	0·489	0·498	0·508	0·517	0·527	0·536	0·546	0·553
Makueni	0·262	0·268	0·274	0·28	0·286	0·293	0·299	0·305	0·311	0·317	0·322	0·328	0·333	0·338	0·343	0·348	0·354	0·361	0·367	0·374	0·381	0·389	0·398	0·406	0·416	0·425	0·435	0·445	0·455	0·462
Mandera	0·172	0·176	0·179	0·183	0·186	0·19	0·193	0·197	0·201	0·204	0·207	0·211	0·214	0·217	0·22	0·223	0·227	0·231	0·235	0·24	0·245	0·251	0·257	0·264	0·27	0·277	0·283	0·29	0·296	0·302
Marsabit	0·202	0·207	0·212	0·217	0·222	0·227	0·232	0·238	0·243	0·249	0·254	0·26	0·265	0·27	0·275	0·281	0·287	0·293	0·299	0·305	0·313	0·32	0·328	0·337	0·346	0·354	0·363	0·372	0·381	0·388
Meru	0·33	0·338	0·345	0·352	0·358	0·365	0·371	0·377	0·383	0·388	0·393	0·398	0·403	0·407	0·412	0·417	0·422	0·428	0·434	0·44	0·448	0·456	0·464	0·472	0·481	0·49	0·5	0·509	0·518	0·525
Migori	0·217	0·223	0·23	0·236	0·243	0·25	0·256	0·262	0·268	0·274	0·28	0·285	0·291	0·296	0·301	0·307	0·314	0·321	0·328	0·336	0·345	0·354	0·364	0·375	0·386	0·397	0·409	0·421	0·433	0·442
Mombasa	0·422	0·428	0·433	0·438	0·443	0·447	0·452	0·456	0·459	0·463	0·465	0·468	0·471	0·473	0·476	0·479	0·483	0·489	0·494	0·5	0·507	0·514	0·522	0·53	0·539	0·548	0·557	0·566	0·575	0·582
Murang'a	0·357	0·365	0·373	0·379	0·386	0·392	0·398	0·404	0·409	0·415	0·42	0·426	0·431	0·436	0·441	0·447	0·453	0·459	0·465	0·471	0·478	0·486	0·493	0·501	0·509	0·518	0·526	0·535	0·543	0·55
Nairobi	0·536	0·54	0·543	0·545	0·547	0·549	0·551	0·553	0·556	0·558	0·561	0·564	0·567	0·57	0·573	0·577	0·582	0·587	0·593	0·598	0·604	0·61	0·617	0·624	0·63	0·637	0·645	0·652	0·66	0·665
Nakuru	0·349	0·357	0·364	0·37	0·377	0·383	0·389	0·394	0·398	0·403	0·407	0·41	0·414	0·417	0·421	0·426	0·431	0·438	0·445	0·452	0·461	0·47	0·479	0·489	0·499	0·509	0·52	0·53	0·54	0·548
Nandi	0·314	0·324	0·333	0·341	0·349	0·357	0·365	0·372	0·379	0·385	0·392	0·397	0·403	0·408	0·413	0·419	0·425	0·432	0·438	0·445	0·453	0·462	0·471	0·481	0·491	0·502	0·513	0·524	0·534	0·543
Narok	0·202	0·212	0·22	0·229	0·236	0·244	0·251	0·257	0·263	0·269	0·274	0·279	0·284	0·289	0·293	0·299	0·304	0·31	0·316	0·322	0·33	0·338	0·347	0·356	0·366	0·376	0·387	0·398	0·409	0·418
Nyamira	0·343	0·352	0·361	0·369	0·377	0·385	0·392	0·399	0·406	0·413	0·419	0·426	0·432	0·437	0·443	0·45	0·456	0·464	0·471	0·478	0·487	0·496	0·506	0·517	0·527	0·538	0·55	0·561	0·571	0·579
Nyandarua	0·334	0·341	0·348	0·354	0·361	0·367	0·373	0·379	0·385	0·391	0·398	0·404	0·41	0·417	0·423	0·43	0·437	0·445	0·452	0·459	0·467	0·475	0·483	0·492	0·501	0·51	0·519	0·529	0·538	0·546
Nyeri	0·388	0·396	0·404	0·41	0·417	0·423	0·428	0·433	0·438	0·442	0·446	0·45	0·454	0·457	0·461	0·466	0·471	0·477	0·483	0·489	0·496	0·504	0·512	0·52	0·528	0·536	0·545	0·553	0·561	0·568
Samburu	0·177	0·183	0·188	0·193	0·199	0·204	0·209	0·214	0·218	0·223	0·228	0·232	0·236	0·241	0·245	0·25	0·255	0·261	0·267	0·274	0·281	0·289	0·297	0·306	0·314	0·324	0·333	0·343	0·353	0·361
Siaya	0·225	0·232	0·238	0·244	0·25	0·257	0·263	0·269	0·274	0·28	0·285	0·289	0·294	0·299	0·303	0·309	0·315	0·323	0·33	0·338	0·347	0·358	0·368	0·38	0·392	0·404	0·416	0·428	0·44	0·449
Taita Taveta	0·351	0·359	0·366	0·373	0·379	0·386	0·392	0·397	0·402	0·407	0·411	0·416	0·419	0·423	0·427	0·431	0·437	0·443	0·449	0·455	0·462	0·47	0·478	0·486	0·495	0·504	0·513	0·522	0·531	0·539
Tana River	0·226	0·232	0·238	0·243	0·249	0·254	0·259	0·264	0·268	0·272	0·275	0·279	0·281	0·284	0·287	0·29	0·295	0·3	0·305	0·31	0·317	0·324	0·331	0·34	0·348	0·357	0·366	0·375	0·384	0·391
Tharaka Nithi	0·341	0·348	0·355	0·362	0·369	0·376	0·383	0·39	0·396	0·403	0·41	0·416	0·423	0·429	0·436	0·442	0·449	0·457	0·464	0·471	0·479	0·488	0·497	0·506	0·516	0·526	0·536	0·546	0·556	0·564
Trans Nzoia	0·321	0·329	0·337	0·344	0·351	0·357	0·364	0·371	0·377	0·383	0·388	0·394	0·4	0·405	0·411	0·416	0·423	0·429	0·436	0·443	0·451	0·459	0·468	0·478	0·488	0·498	0·509	0·52	0·53	0·539
Turkana	0·185	0·191	0·196	0·202	0·208	0·214	0·22	0·226	0·232	0·237	0·242	0·247	0·252	0·256	0·26	0·263	0·268	0·272	0·276	0·28	0·286	0·292	0·299	0·306	0·314	0·322	0·33	0·339	0·347	0·355
Uasin Gishu	0·371	0·38	0·388	0·395	0·402	0·408	0·414	0·42	0·425	0·43	0·435	0·441	0·445	0·45	0·455	0·461	0·467	0·474	0·48	0·488	0·496	0·505	0·514	0·524	0·535	0·545	0·556	0·566	0·577	0·585
Vihiga	0·276	0·284	0·292	0·3	0·307	0·315	0·322	0·329	0·335	0·341	0·346	0·351	0·355	0·359	0·363	0·367	0·371	0·376	0·38	0·385	0·391	0·398	0·405	0·413	0·421	0·429	0·438	0·447	0·457	0·464
Wajir	0·146	0·15	0·154	0·157	0·161	0·165	0·169	0·173	0·176	0·18	0·183	0·185	0·188	0·19	0·192	0·194	0·197	0·2	0·203	0·206	0·21	0·215	0·22	0·225	0·231	0·236	0·242	0·248	0·253	0·259
West Pokot	0·189	0·196	0·203	0·21	0·217	0·224	0·23	0·237	0·243	0·249	0·255	0·261	0·267	0·273	0·28	0·286	0·293	0·301	0·309	0·317	0·326	0·336	0·346	0·357	0·368	0·379	0·39	0·402	0·413	0·422
Madagascar	0·265	0·268	0·27	0·273	0·275	0·277	0·28	0·283	0·286	0·29	0·294	0·299	0·302	0·307	0·311	0·316	0·32	0·324	0·328	0·332	0·336	0·342	0·348	0·355	0·361	0·369	0·376	0·383	0·391	0·396
Malawi	0·213	0·215	0·215	0·217	0·218	0·22	0·225	0·23	0·235	0·24	0·245	0·249	0·254	0·26	0·267	0·274	0·281	0·29	0·299	0·308	0·317	0·326	0·335	0·342	0·35	0·358	0·365	0·372	0·379	0·384
Mozambique	0·12	0·122	0·123	0·126	0·129	0·131	0·137	0·144	0·152	0·159	0·165	0·172	0·179	0·186	0·194	0·201	0·208	0·215	0·223	0·23	0·237	0·244	0·252	0·26	0·268	0·277	0·285	0·294	0·301	0·307
Rwanda	0·257	0·259	0·262	0·265	0·261	0·261	0·262	0·265	0·269	0·272	0·277	0·283	0·291	0·298	0·307	0·315	0·325	0·333	0·342	0·351	0·359	0·368	0·376	0·384	0·391	0·399	0·407	0·415	0·422	0·429
Somalia	0·0508	0·0514	0·0522	0·053	0·0539	0·0547	0·0556	0·0565	0·0574	0·0584	0·0593	0·0603	0·0613	0·0622	0·0632	0·0641	0·0651	0·0661	0·0671	0·0682	0·0692	0·0703	0·0716	0·0728	0·0742	0·0756	0·077	0·0785	0·0799	0·081
South Sudan	0·248	0·251	0·253	0·256	0·259	0·261	0·264	0·267	0·271	0·274	0·277	0·281	0·284	0·288	0·292	0·296	0·301	0·305	0·31	0·315	0·32	0·325	0·33	0·334	0·339	0·342	0·345	0·351	0·358	0·363
Uganda	0·167	0·169	0·172	0·176	0·181	0·187	0·194	0·201	0·208	0·216	0·225	0·234	0·243	0·253	0·262	0·272	0·282	0·293	0·303	0·314	0·325	0·335	0·345	0·355	0·364	0·373	0·382	0·391	0·399	0·404
Tanzania	0·26	0·265	0·269	0·273	0·276	0·28	0·284	0·288	0·292	0·296	0·301	0·305	0·31	0·315	0·32	0·326	0·331	0·337	0·342	0·348	0·354	0·361	0·368	0·375	0·383	0·391	0·399	0·408	0·416	0·423
Zambia	0·299	0·304	0·307	0·311	0·314	0·316	0·319	0·323	0·326	0·33	0·334	0·34	0·345	0·352	0·359	0·367	0·376	0·386	0·396	0·407	0·418	0·429	0·44	0·451	0·462	0·472	0·481	0·491	0·499	0·505
Southern sub-Saharan Africa	0·513	0·517	0·522	0·526	0·53	0·535	0·539	0·544	0·548	0·553	0·557	0·562	0·567	0·571	0·576	0·581	0·586	0·591	0·596	0·601	0·605	0·61	0·614	0·619	0·623	0·628	0·632	0·636	0·639	0·642
Botswana	0·431	0·441	0·451	0·459	0·467	0·475	0·483	0·491	0·498	0·506	0·514	0·521	0·528	0·535	0·541	0·548	0·555	0·562	0·569	0·575	0·581	0·587	0·593	0·6	0·606	0·612	0·618	0·624	0·63	0·634
eSwatini	0·392	0·401	0·41	0·419	0·427	0·435	0·443	0·45	0·456	0·462	0·468	0·474	0·479	0·484	0·49	0·495	0·501	0·507	0·513	0·519	0·526	0·532	0·538	0·545	0·551	0·557	0·563	0·569	0·574	0·577
Lesotho	0·321	0·327	0·333	0·34	0·346	0·353	0·36	0·367	0·373	0·38	0·387	0·393	0·399	0·405	0·411	0·417	0·423	0·429	0·435	0·441	0·448	0·455	0·462	0·469	0·476	0·483	0·489	0·496	0·502	0·507
Namibia	0·454	0·459	0·465	0·47	0·475	0·48	0·486	0·491	0·495	0·5	0·505	0·509	0·514	0·518	0·523	0·529	0·534	0·54	0·546	0·552	0·558	0·564	0·571	0·577	0·584	0·591	0·597	0·603	0·608	0·612
South Africa	0·552	0·556	0·56	0·564	0·567	0·572	0·576	0·58	0·584	0·588	0·593	0·597	0·602	0·607	0·611	0·616	0·622	0·627	0·632	0·637	0·642	0·647	0·651	0·656	0·66	0·664	0·668	0·672	0·676	0·678
Zimbabwe	0·394	0·403	0·409	0·415	0·421	0·426	0·432	0·437	0·441	0·444	0·446	0·448	0·448	0·447	0·444	0·441	0·437	0·433	0·428	0·425	0·425	0·427	0·432	0·438	0·445	0·452	0·459	0·465	0·471	0·476
Western sub-Saharan Africa	0·268	0·272	0·276	0·28	0·284	0·288	0·292	0·297	0·301	0·306	0·311	0·316	0·322	0·328	0·335	0·343	0·351	0·359	0·367	0·375	0·383	0·391	0·399	0·407	0·415	0·422	0·43	0·437	0·443	0·448
Benin	0·209	0·213	0·218	0·222	0·227	0·231	0·236	0·241	0·245	0·25	0·254	0·259	0·263	0·267	0·271	0·275	0·279	0·283	0·288	0·292	0·297	0·301	0·306	0·312	0·318	0·324	0·331	0·338	0·346	0·352
Burkina Faso	0·125	0·128	0·131	0·134	0·137	0·14	0·144	0·147	0·152	0·156	0·161	0·166	0·17	0·175	0·18	0·186	0·191	0·196	0·2	0·205	0·21	0·215	0·22	0·226	0·231	0·236	0·241	0·247	0·252	0·257
Cape Verde	0·292	0·298	0·305	0·311	0·318	0·326	0·333	0·342	0·35	0·36	0·37	0·379	0·388	0·397	0·407	0·415	0·424	0·434	0·443	0·452	0·461	0·469	0·477	0·484	0·491	0·498	0·505	0·512	0·519	0·525
Cameroon	0·313	0·32	0·325	0·33	0·334	0·338	0·342	0·346	0·349	0·353	0·357	0·36	0·364	0·368	0·373	0·378	0·384	0·39	0·397	0·404	0·412	0·42	0·428	0·436	0·445	0·455	0·464	0·474	0·483	0·49
Chad	0·108	0·112	0·115	0·118	0·12	0·123	0·125	0·128	0·13	0·132	0·135	0·137	0·14	0·144	0·149	0·156	0·162	0·167	0·173	0·179	0·185	0·191	0·197	0·203	0·21	0·216	0·223	0·228	0·234	0·238
Côte d'Ivoire	0·256	0·26	0·265	0·27	0·275	0·279	0·285	0·29	0·295	0·301	0·305	0·31	0·314	0·318	0·322	0·325	0·329	0·333	0·337	0·341	0·346	0·35	0·355	0·362	0·369	0·376	0·384	0·393	0·401	0·408
The Gambia	0·218	0·223	0·227	0·232	0·237	0·241	0·246	0·251	0·256	0·262	0·268	0·274	0·28	0·286	0·293	0·3	0·306	0·313	0·319	0·327	0·334	0·341	0·348	0·356	0·363	0·37	0·378	0·385	0·393	0·399
Ghana	0·355	0·362	0·368	0·374	0·38	0·385	0·391	0·396	0·401	0·406	0·411	0·415	0·42	0·426	0·431	0·437	0·444	0·45	0·458	0·466	0·474	0·484	0·494	0·504	0·514	0·523	0·531	0·541	0·549	0·557
Guinea	0·175	0·178	0·182	0·186	0·19	0·194	0·199	0·204	0·209	0·214	0·219	0·224	0·229	0·235	0·239	0·244	0·249	0·253	0·258	0·263	0·267	0·272	0·278	0·284	0·29	0·296	0·303	0·31	0·318	0·325
Guinea-Bissau	0·2	0·205	0·209	0·214	0·219	0·224	0·23	0·236	0·239	0·244	0·249	0·253	0·257	0·261	0·266	0·271	0·275	0·28	0·286	0·291	0·297	0·304	0·31	0·316	0·322	0·328	0·335	0·342	0·349	0·355
Liberia	0·221	0·222	0·219	0·214	0·206	0·196	0·183	0·176	0·175	0·184	0·203	0·22	0·238	0·245	0·252	0·258	0·265	0·272	0·279	0·287	0·296	0·305	0·314	0·325	0·335	0·344	0·351	0·358	0·365	0·37
Mali	0·126	0·129	0·132	0·136	0·139	0·143	0·147	0·151	0·155	0·159	0·163	0·168	0·173	0·178	0·183	0·188	0·193	0·198	0·203	0·209	0·214	0·22	0·225	0·23	0·235	0·241	0·247	0·253	0·259	0·263
Mauritania	0·308	0·314	0·319	0·326	0·332	0·338	0·344	0·349	0·355	0·36	0·365	0·369	0·374	0·379	0·384	0·39	0·398	0·406	0·413	0·42	0·427	0·435	0·443	0·45	0·459	0·467	0·474	0·482	0·49	0·496
Niger	0·0728	0·0746	0·0761	0·0777	0·0793	0·0808	0·0822	0·0836	0·0853	0·0871	0·0887	0·0907	0·093	0·0956	0·0982	0·101	0·104	0·108	0·111	0·115	0·119	0·123	0·128	0·133	0·138	0·143	0·148	0·153	0·158	0·162
Nigeria	0·305	0·308	0·312	0·315	0·319	0·324	0·329	0·334	0·339	0·344	0·35	0·356	0·363	0·371	0·381	0·392	0·402	0·412	0·422	0·432	0·442	0·451	0·46	0·469	0·478	0·487	0·495	0·503	0·51	0·515
São Tomé and PrÍncipe	0·299	0·302	0·306	0·309	0·313	0·317	0·322	0·327	0·332	0·338	0·344	0·351	0·358	0·365	0·373	0·381	0·39	0·398	0·407	0·416	0·424	0·433	0·443	0·452	0·461	0·47	0·478	0·487	0·495	0·502
Senegal	0·227	0·233	0·239	0·245	0·251	0·257	0·262	0·267	0·272	0·277	0·282	0·286	0·29	0·295	0·299	0·304	0·308	0·313	0·318	0·324	0·33	0·336	0·342	0·348	0·354	0·361	0·368	0·375	0·382	0·389
Sierra Leone	0·207	0·209	0·21	0·212	0·215	0·218	0·218	0·218	0·219	0·218	0·219	0·221	0·224	0·229	0·234	0·239	0·245	0·252	0·26	0·267	0·275	0·283	0·292	0·304	0·314	0·321	0·328	0·335	0·342	0·347
Togo	0·266	0·272	0·278	0·281	0·286	0·291	0·296	0·302	0·306	0·31	0·313	0·317	0·32	0·323	0·327	0·33	0·334	0·338	0·342	0·347	0·352	0·358	0·364	0·371	0·379	0·386	0·394	0·402	0·411	0·417

Table S2 Five socio-demographic index quintiles in GBD 2019

**Sdi_quintile**	**Lower_bound**	**Upper_bound**
Low SDI	0	0.454743
Low-middle SDI	0.454743	0.607679
Middle SDI	0.607679	0.689504
High-middle SDI	0.689504	0.805129
High SDI	0.805129	1

Table S3 Regional incident cases and age-standardized incidence rate of genitourinary cancers in 2019

Region	Kidney cancer		Bladder cancer		Prostate cancer	
	Incident cases (×10^3)	ASIR per 100,000 population	Incident cases (×10^3)	ASIR per 100,000 population	Incident cases (×10^3)	ASIR per 100,000 population
**Andean Latin America**	2.02(1.60 - 2.50)	3.51(2.78 - 4.35)	1.38(1.13 - 1.68)	2.52(2.05 - 3.05)	12.09(9.19 - 15.59)	47.52(36.13 - 61.32)
**Australasia**	4.09(3.29 - 5.10)	8.98(7.19 - 11.22)	4.41(3.56 - 5.52)	8.66(6.98 - 10.85)	25.70(19.14 - 37.96)	108.35(80.76 - 159.34)
**Caribbean**	1.86(1.54 - 2.21)	3.68(3.05 - 4.37)	2.82(2.42 - 3.3)	5.45(4.68 - 6.39)	22.76(17.70 - 28.12)	95.83(74.43 - 118.13)
**Central Asia**	3.91(3.52 - 4.34)	4.75(4.31 - 5.24)	3.26(2.93 - 3.60)	4.57(4.09 - 5.04)	4.77(3.84 - 5.73)	18.62(14.69 - 22.13)
**Central Europe**	17.69(15.49 - 20.14)	8.90(7.79 - 10.12)	27.06(23.61 - 30.69)	12.61(10.98 - 14.29)	43.82(32.06 - 51.72)	46.76(34.28 - 55.2)
**Central Latin America**	10.41(8.89 - 12.10)	4.32(3.69 - 5.02)	6.23(5.33 - 7.28)	2.68(2.30 - 3.14)	65.12(51.32 - 86.14)	62.29(49.43 - 82.24)
**Central Sub-Saharan Africa**	0.77(0.58 - 1.00)	1.13(0.84 - 1.55)	1.75(1.11 - 2.58)	3.78(2.35 - 5.56)	4.53(3.18 - 5.80)	29.16(20.09 - 37.48)
**East Asia**	64.26(54.11 - 75.60)	3.32(2.84 - 3.88)	105.42(88.44 - 124.57)	5.25(4.43 - 6.17)	161.97(126.34 - 213.69)	17.72(14.09 - 23.07)
**Eastern Europe**	32.49(29.18 - 36.28)	10.03(9.02 - 11.18)	24.38(21.59 - 27.36)	7.07(6.26 - 7.93)	64.66(45.77 - 79.26)	48.80(34.95 - 59.51)
**Eastern Sub-Saharan Africa**	3.30(2.72 - 4.09)	1.61(1.33 - 1.98)	4.62(3.95 - 5.44)	3.17(2.71 - 3.7)	16.32(12.86 - 20.22)	26.56(20.98 - 32.42)
**High-income Asia Pacific**	17.69(15.15 - 20.21)	4.45(3.87 - 5.07)	35.42(29.62 - 41.17)	7.50(6.42 - 8.69)	67.98(52.43 - 91.27)	31.64(24.54 - 42.35)
**High-income North America**	68.19(58.77 - 78.45)	11.72(10.12 - 13.47)	57.00(49.42 - 65.65)	8.98(7.77 - 10.35)	331.89(262.39 - 494.58)	113.02(89.54 - 168.18)
**North Africa and Middle East**	15.74(13.60 - 18.04)	3.18(2.76 - 3.62)	41.29(34.67 - 49.95)	9.55(8.09 - 11.41)	47.47(36.99 - 55.85)	23.71(18.52 - 27.88)
**Oceania**	0.10(0.08 - 0.13)	1.08(0.87 - 1.41)	0.17(0.13 - 0.22)	2.48(1.96 - 3.11)	0.66(0.48 - 0.84)	26.48(19.89 - 33.17)
**South Asia**	16.77(14.67 - 19.37)	1.13(0.99 - 1.31)	31.55(27.90 - 35.69)	2.38(2.10 - 2.69)	53.95(44.20 - 69.73)	9.26(7.62 - 11.98)
**Southeast Asia**	16.45(13.38 - 20.84)	2.58(2.10 - 3.26)	16.34(14.19 - 19.14)	2.85(2.46 - 3.33)	44.47(34.33 - 52.6)	19.31(14.80 - 22.85)

**Southern Latin America**	7.30(5.58 - 9.29)	9.05(6.92 - 11.57)	5.97(4.74 - 7.48)	7.11(5.63 - 8.92)	19.52(14.60 - 27.16)	53.97(40.42 - 74.92)
**Southern Sub-Saharan Africa**	1.13(1.03 - 1.23)	1.87(1.70 - 2.02)	2.20(1.96 - 2.45)	4.04(3.61 - 4.48)	10.11(8.08 - 11.91)	53.16(41.87 - 60.83)
**Tropical Latin America**	8.42(7.92 - 8.88)	3.54(3.34 - 3.74)	9.71(8.97 - 10.36)	4.09(3.76 - 4.37)	56.94(49.55 - 84.1)	54.94(47.67 - 81.28)
**Western Europe**	75.60(66.61 - 85.73)	9.31(8.20 - 10.59)	138.24(119.20 - 159.26)	14.93(12.82 - 17.3)	325.49(267.13 - 469.92)	78.67(64.34 - 113.34)
**Western Sub-Saharan Africa**	3.58(2.67 - 4.67)	1.20(0.98 - 1.47)	5.07(3.50 - 6.11)	2.93(2.05 - 3.49)	30.23(16.78 - 40.61)	43.52(24.40 - 57.94)

All data reported as number or rate (95% UI). *ASIR* age-standardized incidence rate; *UI* uncertainty interval

Table S4 Regional deaths and age - standardized mortality rate of genitourinary cancers in 2019

Region	Kidney cancer		Bladder cancer		Prostate cancer	
	Deaths (×10^3)	ASMR per 100,000 population	Deaths (×10^3)	ASMR per 100,000 population	Deaths (×10^3)	ASMR per 100,000 population
**Andean Latin America**	1.18(0.94 - 1.46)	2.12(1.69 - 2.63)	0.87(0.72 - 1.05)	1.63(1.33 - 1.97)	6.26(4.85 - 7.86)	25.50(19.68 - 32.03)
**Australasia**	1.71(1.54 - 1.89)	3.41(3.08 - 3.75)	1.63(1.43 - 1.81)	2.96(2.61 - 3.29)	5.41(4.43 - 7.64)	22.32(18.28 - 31.42)
**Caribbean**	0.99(0.83 - 1.16)	1.93(1.62 - 2.27)	1.52(1.31 - 1.75)	2.93(2.54 - 3.38)	9.83(7.85 - 12.06)	42.75(34.14 - 52.35)
**Central Asia**	2.06(1.87 - 2.29)	2.72(2.48 - 3.00)	1.60(1.44 - 1.77)	2.56(2.30 - 2.82)	2.57(2.06 - 3.06)	12.43(9.75 - 14.81)
**Central Europe**	9.63(8.50 - 10.84)	4.52(3.99 - 5.11)	11.88(10.47 - 13.44)	5.29(4.66 - 5.99)	18.83(14.10 - 22.05)	21.56(16.05 - 25.11)
**Central Latin America**	5.60(4.80 - 6.51)	2.38(2.04 - 2.76)	3.40(2.93 - 3.95)	1.51(1.30 - 1.76)	21.67(16.87 - 28.46)	22.11(17.32 - 29.24)
**Central Sub - Saharan Africa**	0.51(0.38 - 0.69)	0.89(0.66 - 1.25)	1.38(0.86 - 2.03)	3.35(2.06 - 4.93)	4.04(2.81 - 5.17)	29.92(20.54 - 38.38)
**East Asia**	25.49(21.43 - 29.95)	1.30(1.10 - 1.52)	42.16(36.02 - 49.35)	2.27(1.95 - 2.64)	57.21(45.38 - 74.04)	7.88(6.36 - 9.91)
**Eastern Europe**	14.90(13.40 - 16.55)	4.36(3.92 - 4.84)	10.93(9.70 - 12.2)	3.11(2.75 - 3.47)	20.46(15.2 - 25.07)	17.53(12.95 - 21.71)
**Eastern Sub - Saharan Africa**	2.17(1.80 - 2.68)	1.27(1.05 - 1.56)	3.63(3.11 - 4.24)	2.74(2.35 - 3.19)	13.81(10.91 - 16.67)	24.80(19.56 - 29.64)
**High - income Asia Pacific**	9.71(8.42 - 10.48)	2.00(1.79 - 2.13)	12.88(10.68 - 14.23)	2.26(1.92 - 2.47)	19.38(14.97 - 23.66)	8.60(6.67 - 10.53)
**High - income North America**	22.00(20.41 - 23.15)	3.49(3.25 - 3.66)	25.76(23.43 - 27.45)	3.81(3.49 - 4.05)	54.85(46.83 - 79.75)	19.08(16.25 - 27.72)
**North Africa and Middle East**	6.00(5.16 - 6.89)	1.40(1.21 - 1.61)	15.46(13.17 - 18.42)	4.05(3.49 - 4.81)	19.09(15.24 - 22.50)	11.71(9.39 - 13.95)
**Oceania**	0.05(0.04 - 0.06)	0.64(0.50 - 0.85)	0.10(0.08 - 0.13)	1.82(1.44 - 2.27)	0.51(0.38 - 0.65)	24.18(18.15 - 30.53)
**South Asia**	10.86(9.5 - 12.53)	0.78(0.68 - 0.90)	21.99(19.47 - 24.71)	1.80(1.59 - 2.01)	42.22(35.17 - 54.87)	8.12(6.67 - 10.64)
**Southeast Asia**	7.53(6.15 - 9.48)	1.27(1.04 - 1.60)	9.03(7.88 - 10.50)	1.76(1.53 - 2.03)	27.21(20.47 - 32.07)	13.56(10.27 - 15.97)

**Southern Latin America**	4.19(3.81 - 4.58)	5.05(4.59 - 5.51)	3.07(2.81 - 3.30)	3.58(3.28 - 3.86)	10.10(8.38 - 13.38)	29.53(24.42 - 38.78)
**Southern Sub - Saharan Africa**	0.72(0.65 - 0.78)	1.30(1.18 - 1.40)	1.49(1.31 - 1.66)	2.99(2.64 - 3.32)	7.41(5.82 - 8.41)	45.08(34.35 - 50.53)
**Tropical Latin America**	4.72(4.41 - 4.99)	1.99(1.85 - 2.10)	5.64(5.12 - 6.03)	2.45(2.21 - 2.62)	23.90(20.37 - 34.73)	25.48(21.50 - 37.14)
**Western Europe**	34.36(31.51 - 36.30)	3.65(3.40 - 3.83)	50.51(45.16 - 54.46)	4.78(4.32 - 5.14)	95.77(79.23 - 133.05)	21.60(17.88 - 29.78)
**Western Sub - Saharan Africa**	2.05(1.58 - 2.57)	0.86(0.71 - 1.02)	3.81(2.66 - 4.52)	2.48(1.74 - 2.91)	26.3(14.73 - 35.42)	41.74(23.48 - 55.68)

All data reported as number or rate (95% UI). *ASMR* age - standardized mortality rate; *UI* uncertainty interval

Table S5 Regional DALYs and age - standardized DALYs rate of genitourinary cancers in 2019

Region	Kidney cancer		Bladder cancer		Prostate cancer	
	DALYs (×10³)	ASDR per 100,000 population	DALYs (×10³)	ASDR per 100,000 population	DALYs (×10³)	ASDR per 100,000 population
**Andean Latin America**	31.13 (24.76 - 38.61)	53.62 (42.59 - 66.39)	16.30 (13.24 - 19.80)	29.49 (24.01 - 35.80)	101.03 (78.16 - 129.24)	397.17 (307.58 - 507.19)
**Australasia**	35.15 (31.96 - 38.32)	77.88 (71.23 - 84.88)	25.85 (23.07 - 28.72)	50.81 (45.57 - 56.37)	1053.23 (840.97 - 1368.07)	390.42 (323.54 - 552.32)
**Caribbean**	28.27 (23.13 - 33.70)	56.56 (46.13 - 67.39)	28.60 (24.79 - 33.11)	55.28 (47.89 - 63.99)	429.03 (310.36 - 522.73)	704.84 (565.44 - 866.14)
**Central Asia**	63.86 (57.32 - 71.31)	75.28 (67.94 - 83.53)	39.20 (35.20 - 43.68)	53.60 (48.20 - 59.36)	1033.62 (897.37 - 1515.66)	209.81 (167.55 - 250.27)
**Central Europe**	215.43 (189.34 - 243.88)	109.98 (96.51 - 124.60)	230.52 (201.86 - 262.13)	107.36 (93.95 - 122.07)	281.94 (220.71 - 345.22)	357.74 (264.07 - 417.95)
**Central Latin America**	154.32 (132.28 - 179.83)	63.66 (54.61 - 74.10)	67.32 (57.82 - 78.82)	28.82 (24.72 - 33.71)	81.22 (57.40 - 104.03)	369.29 (289.00 - 482.77)
**Central Sub - Saharan Africa**	18.59 (13.99 - 24.23)	23.94 (17.95 - 32.55)	33.21 (21.20 - 48.53)	65.11 (40.87 - 95.76)	93.66 (77.67 - 133.44)	469.77 (325.09 - 602.73)
**East Asia**	679.56 (572.05 - 800.58)	34.98 (29.69 - 40.58)	856.97 (729.75 - 1009.07)	42.44 (36.28 - 49.64)	423.13 (367.76 - 615.83)	120.64 (96.98 - 153.79)
**Eastern Europe**	376.93 (337.00 - 421.46)	116.44 (104.17 - 129.80)	231.53 (203.47 - 260.69)	67.13 (59.15 - 75.53)	378.32 (297.27 - 494.09)	336.41 (244.51 - 407.66)
**Eastern Sub - Saharan Africa**	76.89 (62.81 - 95.71)	32.81 (27.10 - 40.54)	84.04 (71.43 - 99.34)	53.51 (45.83 - 62.70)	482.04 (271.56 - 654.18)	432.87 (342.49 - 523.92)

**High - income Asia Pacific**	168.35 (153.45 - 178.77)	43.16 (40.29 - 45.66)	185.46 (162.08 - 202.86)	38.74 (34.73 - 42.25)	143.41 (113.29 - 165.24)	138.04 (112.79 - 177.07)
**High - income North America**	481.34 (453.06 - 506.24)	82.97 (78.32 - 87.12)	433.84 (405.14 - 458.84)	68.46 (64.26 - 72.24)	1499.83 (1261.99 - 2106.39)	355.25 (308.31 - 519.22)
**North Africa and Middle East**	183.63 (157.59 - 211.82)	37.23 (32.00 - 42.58)	378.67 (314.9 - 461.35)	86.17 (72.35 - 103.57)	166.52 (133.66 - 205.11)	186.81 (147.72 - 219.52)
**Oceania**	1.86 (1.43 - 2.44)	18.40 (14.46 - 24.04)	2.82 (2.18 - 3.68)	38.55 (30.03 - 49.23)	510.04 (381.79 - 602.06)	375.63 (279.62 - 475.23)
**South Asia**	314.86 (276.23 - 363.81)	20.64 (18.14 - 23.83)	487.70 (432.73 - 550.47)	35.29 (31.13 - 39.71)	345.76 (270.78 - 404.98)	127.05 (105.59 - 164.67)
**Southeast Asia**	225.24 (181.85 - 284.4)	34.79 (28.13 - 43.89)	194.86 (169.28 - 229.27)	33.19 (28.9 - 38.79)	329.83 (243.66 - 384.77)	218.62 (164.66 - 257.14)
**Southern Latin America**	99.60 (90.78 - 108.56)	124.32 (113.32 - 135.49)	56.96 (52.39 - 61.51)	68.01 (62.64 - 73.45)	298.74 (243.71 - 380.29)	462.58 (387.30 - 614.38)
**Southern Sub - Saharan Africa**	21.00 (18.90 - 22.96)	33.41 (30.19 - 36.36)	34.67 (30.37 - 39.31)	61.06 (53.81 - 68.9)	165.74 (139.82 - 220.45)	726.31 (574.70 - 822.50)
**Tropical Latin America**	126.91 (119.97 - 133.49)	53.24 (50.28 - 56.02)	109.22 (101.21 - 116.27)	45.60 (42.20 - 48.57)	763.89 (633.04 - 986.04)	417.24 (360.53 - 605.72)
**Western Europe**	654.12 (615.23 - 685.90)	81.41 (77.10 - 85.24)	805.53 (736.60 - 871.05)	86.82 (80.17 - 93.93)	53.79 (43.94 - 64.79)	349.18 (293.71 - 489.54)
**Western Sub - Saharan Africa**	95.78 (65.68 - 131.11)	26.58 (20.61 - 33.10)	89.31 (61.20 - 107.24)	48.18 (33.36 - 57.22)	10.10 (7.47 - 12.93)	661.04 (370.00 - 891.73)

All data reported as number or rate (95% UI). *DALYs* disability - adjusted life - years; *ASDR* age - standardized DALYs rate; *UI* uncertainty interval

Table S6 Incidence, mortality and DALYs of genitourinary cancers among the top three and bottom three countries in 2019

**Measure**	**Type**	**Sex**	**Top three countries**			**Bottom three countries**		
**Incident cases**	Kidney cancer	Both	USA (61,541.43)	China (59,826.74)	Russian Federation (22,381.03)	Tuvalu (0.17)	Niue (0.06)	Tokelau (0.02)
		Male	China (42,553.79)	USA (40,685.52)	Russian Federation (12,868.30)	Tuvalu (0.11)	Niue (0.05)	Tokelau (0.01)
		Female	USA (20,855.90)	China (17,272.96)	Russian Federation (9512.73)	Nauru (0.16)	Niue (0.02)	Tokelau (0.01)
	Bladder cancer	Both	China (100,020.20)	USA (49,737.46)	Italy (28,608.02)	Nauru (0.22)	Niue (0.09)	Tokelau (0.04)
		Male	China (82,680.76)	USA (35,559.27)	Italy (23,003.22)	Nauru (0.13)	Niue (0.06)	Tokelau (0.02)
		Female	China (17,339.44)	USA (14,178.18)	Japan (7249.58)	Nauru (0.09)	Niue (0.03)	Tokelau (0.02)
	Prostate cancer	Male	USA (308,584.29)	China (153,447.62)	Germany (75,379.81)	Niue (0.50)	Nauru (0.48)	Tokelau (0.19)
**Deaths**	Kidney cancer	Both	China (23,954.24)	USA (19,753.06)	Russian Federation (10,193.69)	Nauru (0.07)	Niue (0.03)	Tokelau (0.01)
		Male	China (16,883.05)	USA (13,021.69)	Russian Federation (6152.43)	Nauru (0.05)	Niue (0.02)	Tokelau (0.01)
		Female	China (7071.19)	USA (6731.37)	Russian Federation (4041.27)	Nauru (0.05)	Niue (0.02)	Tokelau (0.01)
	Bladder cancer	Both	China (40,094.24)	USA (22,767.44)	India (14,506.57)	Nauru (0.09)	Niue (0.05)	Tokelau (0.02)
		Male	China (31,504.64)	USA (16,352.97)	India (10,060.67)	Nauru (0.05)	Niue (0.03)	Tokelau (0.01)
		Female	China	USA	India	Nauru	Niue	Tokelau

			(8589.60)	(6414.47)	(4445.90)	(0.04)	(0.02)	(0.01)
	Prostate cancer	Male	China (54,390.88)	USA (48,323.44)	India (32,107.15)	Niue (0.27)	Nauru (0.26)	Tokelau (0.13)
**DALYs**	Kidney cancer	Both	China (642,799.34)	USA (434,447.43)	Russian Federation (253,081.09)	Tuvalu (2.65)	Niue (0.74)	Tokelau (0.32)
		Male	China (462,868.56)	USA (297,788.77)	Russian Federation (163,234.37)	Tuvalu (1.75)	Niue (0.56)	Tokelau (0.18)
		Female	China (179,930.77)	USA (136,658.66)	Russian Federation (89,846.73)	Nauru (0.78)	Niue (0.18)	Tokelau (0.13)
	Bladder cancer	Both	China (816,119.14)	USA (384,236.57)	India (313,585.91)	Nauru (2.88)	Niue (0.98)	Tokelau (0.53)
		Male	China (651,102.48)	USA (281,706.04)	India (217,921.25)	Nauru (1.64)	Niue (0.60)	Tokelau (0.27)
		Female	China (165,016.66)	USA (102,530.53)	India (95,664.65)	Palau (1.11)	Niue (0.38)	Tokelau (0.26)
	Prostate cancer	Male	China (1,002,594.87)	USA (926,634.87)	India (580,128.20)	Nauru (6.09)	Niue (4.59)	Tokelau (2.15)
**ASIR (1/10^5)**	Kidney cancer	Both	Czechia (15.66)	Estonia (12.96)	Iceland (12.54)	Bangladesh (0.86)	Papua New Guinea (0.79)	Niger (0.74)
		Male	Czechia (22.25)	Estonia (19.85)	Lithuania (18.89)	Kenya (0.98)	Papua New Guinea (0.98)	Niger (0.79)
		Female	Czechia (10.12)	Monaco (8.50)	Finland (8.27)	Bangladesh (0.65)	Central African Republic (0.61)	Papua New Guinea (0.59)
	Bladder cancer	Both	Monaco (31.92)	Lebanon (30.18)	San Marino (25.29)	Bangladesh (1.59)	Guatemala (1.53)	Nigeria (1.39)
		Male	Monaco (54.30)	Lebanon (53.97)	San Marino (44.73)	Bangladesh (2.26)	Guatemala (2.00)	Nigeria (1.91)
		Female	Monaco	Lebanon	Zimbabwe	Nigeria	Philippines	Bangladesh

			(12.54)	(10.75)	(9.64)	(0.91)	(0.91)	(0.85)
	Prostate cancer	Male	Saint Kitts and Nevis (235.26)	United States Virgin Islands (202.78)	Dominica (195.69)	Nepal (9.80)	India (8.91)	Bangladesh (8.87)
**ASMR (1/10^{5})**	Kidney cancer	Both	Uruguay (6.56)	Czechia (6.42)	Greenland (6.31)	Bangladesh (0.59)	Niger (0.55)	Papua New Guinea (0.49)
		Male	Uruguay (10.37)	Czechia (9.67)	Lithuania (9.06)	Bangladesh (0.76)	Papua New Guinea (0.63)	Niger (0.59)
		Female	Greenland (4.55)	Czechia (3.92)	Uruguay (3.73)	Vanuatu (0.45)	Bangladesh (0.41)	Papua New Guinea (0.34)
	Bladder cancer	Both	Lebanon (10.38)	Mali (10.06)	Monaco (9.44)	El Salvador (1.11)	Albania (1.08)	Palau (1.03)
		Male	Lebanon (17.45)	Monaco (16.05)	Mali (14.73)	Guatemala (1.50)	El Salvador (1.49)	Albania (1.47)
		Female	Zimbabwe (7.92)	Malawi (6.16)	Qatar (5.15)	Turkmenistan (0.64)	Sri Lanka (0.62)	Palau (0.59)
	Prostate cancer	Male	Dominica (126.28)	Grenada (98.79)	Saint Kitts and Nevis (97.13)	India (7.75)	Sri Lanka (7.39)	Egypt (7.01)
**ASDR (1/10^{5})**	Kidney cancer	Both	Uruguay (166.63)	Greenland (148.46)	Czechia (144.85)	Niger (16.95)	Bangladesh (15.62)	Papua New Guinea (14.81)
		Male	Uruguay (254.19)	Lithuania (219.46)	Ukraine (215.93)	Bangladesh (19.56)	Niger (19.44)	Papua New Guinea (18.72)
		Female	Greenland (104.13)	Uruguay (95.31)	Czechia (84.77)	Vanuatu (12.57)	Bangladesh (11.41)	Papua New Guinea (10.59)
	Bladder cancer	Both	Egypt (201.75)	Mali (197.04)	Lebanon (192.75)	El Salvador (20.82)	Guatemala (20.77)	Albania (20.70)
		Male	Lebanon (322.16)	Egypt (310.81)	Monaco (288.99)	Albania (27.67)	El Salvador (27.59)	Guatemala (26.30)

	Female	Zimbabwe (164.14)	Malawi (121.52)	Mali (103.32)	Paraguay (11.94)	Palau (11.13)	Sri Lanka (10.91)
Prostate cancer	Male	Dominica (1923.95)	Saint Kitts and Nevis (1607.79)	Grenada (1596.00)	Egypt (119.06)	China (118.94)	Bangladesh (118.34)

DALYs disability-adjusted life-years; *ASIR* age-standardized incidence rate; *ASMR* age-standardized mortality rate; *ASDR* age-standardized DALYs rate

Table S7 EAPC of ASMR for genitourinary cancers in 204 countries and territories from 1990 to 2019

**Location**	**EAPC (95% CI)**		
	**Kidney cancer**	**Bladder cancer**	**Prostate cancer**
**Afghanistan**	0.89(0.67 - 1.10)	-0.44(-0.47 to -0.40)	0.26(0.21 - 0.32)
**Albania**	2.14(1.83 - 2.46)	-0.28(-0.44 to -0.12)	-0.10(-0.22 - 0.01)
**Algeria**	0.69(0.57 - 0.82)	-0.75(-1.06 to -0.43)	-0.67(-0.71 to -0.62)
**American Samoa**	0.01(-0.34 - 0.35)	1.58(1.29 - 1.87)	0.24(0.11 - 0.36)
**Andorra**	0.15(0.09 - 0.21)	-0.95(-1.01 to -0.89)	-0.58(-0.63 to -0.53)
**Angola**	1.26(1.14 - 1.37)	-0.22(-0.27 to -0.18)	0.75(0.71 - 0.79)
**Antigua and Barbuda**	-0.54(-0.98 to -0.10)	0.67(0.44 - 0.89)	0.25(-0.10 - 0.59)
**Argentina**	-0.31(-0.54 to -0.08)	-1.10(-1.20 to -1.00)	-0.22(-0.46 - 0.03)
**Armenia**	5.74(4.84 - 6.65)	-0.05(-0.27 - 0.16)	1.75(1.63 - 1.86)
**Australia**	-0.53(-0.62 to -0.44)	-1.05(-1.17 to -0.93)	-1.48(-1.63 to -1.33)
**Austria**	-1.37(-1.47 to -1.26)	-1.02(-1.11 to -0.94)	-1.41(-1.57 to -1.25)
**Azerbaijan**	0.32(0.04 - 0.60)	0.83(0.72 - 0.94)	0.92(0.81 - 1.04)
**Bahamas**	-0.69(-0.94 to -0.43)	0.21(0.08 - 0.33)	-0.01(-0.20 - 0.18)
**Bahrain**	-0.90(-1.34 to -0.46)	-1.87(-2.09 to -1.65)	-0.46(-0.72 to -0.19)
**Bangladesh**	0.56(0.44 - 0.69)	-0.30(-0.48 to -0.12)	-0.44(-0.61 to -0.26)
**Barbados**	-0.87(-1.20 to -0.54)	0.37(0.26 - 0.49)	0.01(-0.32 - 0.34)
**Belarus**	3.88(2.90 - 4.86)	-1.41(-1.86 to -0.95)	0.62(0.44 - 0.80)
**Belgium**	-0.11(-0.33 - 0.12)	-1.15(-1.43 to -0.88)	-2.16(-2.27 to -2.04)

**Belize**	-0.14(-0.27 - 0.01)	0.79(0.43 - 1.15)	0.81(0.24 - 1.39)
**Benin**	1.34(1.21 - 1.48)	-1.90(-2.03 to -1.78)	1.86(1.68 - 2.03)
**Bermuda**	-2.00(-2.42 to -1.58)	-0.91(-1.09 to -0.72)	-0.26(-0.36 to -0.17)
**Bhutan**	2.22(2.15 - 2.30)	0.85(0.80 - 0.89)	1.04(0.98 - 1.10)
**Bolivia (Plurinational State of)**	1.58(1.53 - 1.64)	0.15(0.06 - 0.25)	0.80(0.74 - 0.85)
**Bosnia and Herzegovina**	2.28(2.01 - 2.55)	1.59(1.36 - 1.81)	1.30(1.04 - 1.56)
**Botswana**	1.43(1.02 - 1.83)	-0.31(-0.59 to -0.02)	0.22(-0.20 - 0.64)
**Brazil**	1.15(1.05 - 1.24)	-0.41(-0.45 to -0.37)	-0.28(-0.51 to -0.05)
**Brunei Darussalam**	1.70(1.47 - 1.93)	-0.08(-0.25 - 0.09)	1.93(1.48 - 2.38)
**Bulgaria**	5.83(4.92 - 6.75)	2.15(1.74 - 2.57)	2.07(1.78 - 2.36)
**Burkina Faso**	1.26(1.14 - 1.38)	-2.19(-2.42 to -1.95)	1.93(1.82 - 2.05)
**Burundi**	0.27(0.21 - 0.33)	-1.59(-1.72 to -1.47)	-0.19(-0.25 to -0.14)
**Cabo Verde**	4.32(3.65 - 4.99)	2.58(1.87 - 3.30)	2.53(1.74 - 3.33)
**Cambodia**	1.50(1.44 - 1.57)	-0.09(-0.27 - 0.08)	1.18(1.11 - 1.25)
**Cameroon**	0.82(0.77 - 0.86)	-1.12(-1.23 to -1.01)	1.90(1.74 - 2.06)
**Canada**	1.04(0.78 - 1.30)	-0.98(-1.07 to -0.88)	-2.30(-2.52 to -2.08)
**Central African Republic**	0.26(0.17 - 0.35)	-0.90(-0.95 to -0.86)	0.01(-0.03 - 0.05)
**Chad**	1.36(1.29 - 1.43)	-0.59(-0.72 to -0.47)	2.27(2.07 - 2.48)
**Chile**	0.62(0.51 - 0.73)	-0.03(-0.11 - 0.06)	0.31(0.05 - 0.57)
**China**	2.79(2.35 - 3.24)	-0.53(-0.58 to -0.49)	-0.22(-0.26 to -0.17)
**Colombia**	0.62(0.53 - 0.71)	-1.66(-1.80 to -1.52)	-1.20(-1.44 to -0.96)
**Comoros**	1.10(1.00 - 1.19)	-0.44(-0.56 to -0.32)	0.10(0.02 - 0.17)

**Congo**	0.46(0.28 - 0.65)	-0.56(-0.77 to -0.35)	0.01(-0.14 - 0.15)
**Cook Islands**	0.08(-0.07 - 0.23)	-0.04(-0.21 - 0.13)	-0.84(-0.90 to -0.77)
**Costa Rica**	1.79(1.59 - 1.99)	-0.46(-0.63 to -0.30)	0.63(0.30 - 0.96)
**Croatia**	3.85(3.22 - 4.48)	0.44(0.30 - 0.57)	0.30(0.12 - 0.47)
**Cuba**	-0.77(-1.20 to -0.32)	0.46(0.32 - 0.60)	0.62(0.55 - 0.70)
**Cyprus**	2.66(2.33 - 3.00)	-0.45(-0.66 to -0.24)	0.25(-0.13 - 0.64)
**Czechia**	0.60(0.14 - 1.06)	-0.48(-0.59 to -0.38)	-0.30(-0.58 to -0.03)
**Cote d'Ivoire**	0.62(0.52 - 0.72)	-1.80(-1.94 to -1.66)	1.67(1.51 - 1.83)
**Democratic People's Republic of Korea**	0.58(0.51 - 0.66)	-0.44(-0.49 to -0.40)	0.20(0.10 - 0.30)
**Democratic Republic of the Congo**	-0.13(-0.39 - 0.14)	-1.37(-1.56 to -1.18)	0.14(0.10 - 0.18)
**Denmark**	0.53(-0.02 - 1.08)	-0.76(-1.20 to -0.31)	-0.46(-0.65 to -0.27)
**Djibouti**	2.30(2.19 - 2.41)	0.20(0.16 - 0.24)	0.46(0.42 - 0.50)
**Dominica**	-0.10(-0.46 - 0.26)	0.78(0.66 - 0.91)	0.70(0.52 - 0.88)
**Dominican Republic**	2.11(1.57 - 2.66)	1.50(1.29 - 1.71)	1.42(0.86 - 1.99)
**Ecuador**	1.93(1.54 - 2.33)	0.56(0.25 - 0.87)	0.81(0.59 - 1.03)
**Egypt**	1.80(1.63 - 1.96)	0.34(0.23 - 0.45)	0.23(0.07 - 0.39)
**El Salvador**	1.43(1.28 - 1.58)	0.24(0.11 - 0.37)	0.92(0.49 - 1.34)
**Equatorial Guinea**	3.93(3.70 - 4.17)	1.11(0.94 - 1.27)	1.29(1.21 - 1.36)
**Eritrea**	1.81(1.52 - 2.11)	0.11(-0.10 - 0.33)	0.12(-0.12 - 0.36)
**Estonia**	3.67(2.91 - 4.43)	-0.10(-0.28 - 0.07)	1.47(1.22 - 1.71)
**Eswatini**	1.50(0.79 - 2.21)	0.37(0.12 - 0.62)	0.87(0.62 - 1.12)
**Ethiopia**	0.44(0.18 - 0.71)	-0.51(-0.60 to -0.42)	0.73(0.63 - 0.83)

**Fiji**	0.44(0.31 - 0.57)	1.03(0.79 - 1.27)	0.70(0.52 - 0.87)
**Finland**	-0.29(-0.37 to -0.21)	-1.29(-1.37 to -1.20)	-1.32(-1.49 to -1.16)
**France**	-0.15(-0.24 to -0.05)	-0.89(-0.92 to -0.86)	-2.17(-2.35 to -2.00)
**Gabon**	1.53(1.44 - 1.62)	-0.35(-0.43 to -0.27)	0.57(0.53 - 0.62)
**Gambia**	1.41(1.26 - 1.57)	0.49(0.36 - 0.61)	0.73(0.64 - 0.82)
**Georgia**	2.09(1.62 - 2.56)	2.13(1.75 - 2.51)	2.53(1.95 - 3.11)
**Germany**	-0.09(-0.21 - 0.04)	-1.30(-1.58 to -1.01)	-1.12(-1.28 to -0.96)
**Ghana**	-0.05(-0.33 - 0.22)	-1.22(-1.39 to -1.04)	-0.36(-0.54 to -0.18)
**Greece**	-0.05(-0.25 - 0.14)	-1.10(-1.24 to -0.96)	-1.09(-1.38 to -0.79)
**Greenland**	1.30(0.94 - 1.67)	-1.20(-1.32 to -1.08)	0.03(-0.06 - 0.12)
**Grenada**	0.06(-0.40 - 0.52)	0.66(0.47 - 0.84)	0.89(0.06 - 1.73)
**Guam**	-1.20(-1.51 to -0.90)	0.10(-0.18 - 0.39)	-0.78(-1.14 to -0.42)
**Guatemala**	1.52(0.99 - 2.06)	-0.71(-0.83 to -0.59)	1.66(0.96 - 2.36)
**Guinea**	1.07(1.01 - 1.14)	0.40(0.31 - 0.49)	0.97(0.82 - 1.12)
**Guinea-Bissau**	0.54(0.46 - 0.62)	-1.44(-1.55 to -1.33)	1.91(1.76 - 2.05)
**Guyana**	-0.42(-0.70 to -0.15)	0.19(0.11 - 0.26)	0.21(0.05 - 0.36)
**Haiti**	-0.57(-0.85 to -0.29)	-0.14(-0.26 to -0.02)	0.20(0.18 - 0.22)
**Honduras**	2.59(2.43 - 2.76)	1.73(1.54 - 1.93)	1.65(1.32 - 1.98)
**Hungary**	-0.13(-0.43 - 0.18)	-0.15(-0.32 - 0.02)	-0.90(-1.08 to -0.71)
**Iceland**	-0.21(-0.45 - 0.04)	-1.37(-1.50 to -1.25)	-1.07(-1.30 to -0.85)
**India**	1.30(1.20 - 1.40)	-0.26(-0.37 to -0.14)	-0.29(-0.39 to -0.18)
**Indonesia**	2.50(2.44 - 2.57)	0.56(0.50 - 0.62)	1.92(1.83 - 2.01)

**Iran (Islamic Republic of)**	1.23(1.00 - 1.46)	0.18(0.12 - 0.24)	0.51(0.31 - 0.72)
**Iraq**	1.54(1.27 - 1.80)	0.97(0.72 - 1.23)	0.63(0.49 - 0.77)
**Ireland**	0.74(0.48 - 1.01)	-0.59(-0.78 to -0.39)	-1.27(-1.50 to -1.05)
**Israel**	-0.22(-0.48 - 0.05)	-0.57(-0.75 to -0.38)	-1.74(-1.99 to -1.49)
**Italy**	-0.12(-0.17 to -0.07)	-1.29(-1.36 to -1.22)	-1.02(-1.21 to -0.82)
**Jamaica**	-0.85(-1.32 to -0.37)	-0.40(-0.69 to -0.11)	1.90(1.34 - 2.46)
**Japan**	0.38(0.24 - 0.51)	-0.60(-0.68 to -0.53)	-0.50(-0.59 to -0.42)
**Jordan**	2.33(2.09 - 2.58)	-0.39(-0.53 to -0.26)	0.31(0.06 - 0.57)
**Kazakhstan**	0.06(-0.37 - 0.50)	-0.50(-0.92 to -0.08)	0.67(0.53 - 0.81)
**Kenya**	2.88(2.68 - 3.09)	1.40(1.21 - 1.58)	1.98(1.77 - 2.19)
**Kiribati**	0.19(-0.05 - 0.42)	-0.07(-0.10 to -0.03)	-0.40(-0.58 to -0.23)
**Kuwait**	0.26(-0.30 - 0.82)	-0.10(-0.46 - 0.27)	0.94(0.59 - 1.28)
**Kyrgyzstan**	1.31(0.83 - 1.79)	-0.38(-0.73 to -0.03)	-0.83(-1.04 to -0.62)
**Lao People's Democratic Republic**	0.95(0.91 - 0.98)	-0.82(-0.96 to -0.67)	0.41(0.36 - 0.45)
**Latvia**	3.65(2.92 - 4.38)	0.74(0.51 - 0.97)	1.82(1.45 - 2.19)
**Lebanon**	2.73(2.48 - 2.99)	-0.44(-0.49 to -0.39)	0.70(0.57 - 0.83)
**Lesotho**	2.96(2.76 - 3.16)	1.42(1.25 - 1.58)	1.31(1.08 - 1.55)
**Liberia**	1.10(0.62 - 1.59)	-1.96(-2.06 to -1.86)	1.80(1.62 - 1.99)
**Libya**	1.37(1.24 - 1.50)	0.14(0.02 - 0.26)	0.29(0.21 - 0.37)
**Lithuania**	3.42(2.69 - 4.16)	-0.48(-0.71 to -0.25)	1.79(1.28 - 2.31)
**Luxembourg**	-0.91(-1.01 to -0.82)	-1.10(-1.15 to -1.04)	-2.11(-2.21 to -2.01)
**Madagascar**	0.87(0.80 - 0.94)	-1.21(-1.38 to -1.04)	-0.38(-0.52 to -0.25)

**Malawi**	1.06(0.97 - 1.16)	-0.22(-0.31 to -0.14)	0.86(0.75 - 0.98)
**Malaysia**	1.54(1.42 - 1.67)	-0.5(-0.77 to -0.23)	0.02(-0.22 - 0.25)
**Maldives**	0.75(0.53 - 0.97)	-1.47(-1.59 to -1.34)	0.06(-0.03 - 0.16)
**Mali**	1.14(1.07 - 1.20)	-0.08(-0.15 to -0.01)	0.70(0.66 - 0.75)
**Malta**	0.20(0.05 - 0.35)	-1.50(-1.58 to -1.42)	-1.41(-1.55 to -1.27)
**Marshall Islands**	0.57(0.45 - 0.68)	0.54(0.42 - 0.66)	0.01(-0.20 - 0.22)
**Mauritania**	0.43(0.35 - 0.51)	-1.92(-2.03 to -1.81)	1.55(1.39 - 1.70)
**Mauritius**	1.67(1.41 - 1.93)	-1.65(-1.92 to -1.38)	0.27(-0.05 - 0.60)
**Mexico**	1.07(1.00 - 1.15)	-0.32(-0.40 to -0.23)	-0.17(-0.28 to -0.07)
**Micronesia (Federated States of)**	0.26(0.13 - 0.39)	0.57(0.53 - 0.62)	0.55(0.47 - 0.64)
**Monaco**	0.89(0.70 - 1.09)	0.71(0.67 - 0.75)	-0.29(-0.36 to -0.22)
**Mongolia**	3.14(2.85 - 3.42)	-2.80(-3.24 to -2.36)	0.01(-0.15 - 0.16)
**Montenegro**	1.38(1.30 - 1.46)	0.51(0.43 - 0.58)	0.81(0.72 - 0.89)
**Morocco**	1.77(1.56 - 1.99)	0.58(0.30 - 0.86)	0.60(0.16 - 1.05)
**Mozambique**	2.43(2.29 - 2.56)	-0.15(-0.27 to -0.03)	1.17(1.11 - 1.23)
**Myanmar**	1.51(1.44 - 1.58)	-0.73(-0.79 to -0.67)	0.76(0.73 - 0.78)
**Namibia**	1.71(1.49 - 1.92)	0.27(0.06 - 0.47)	2.08(1.95 - 2.21)
**Nauru**	-0.33(-0.49 to -0.16)	0.30(0.18 - 0.41)	0.58(0.44 - 0.72)
**Nepal**	2.41(2.17 - 2.65)	0.56(0.43 - 0.68)	0.97(0.83 - 1.12)
**Netherlands**	0.32(0.15 - 0.48)	-0.39(-0.56 to -0.23)	-0.99(-1.14 to -0.84)
**New Zealand**	0.09(-0.05 - 0.23)	-1.47(-1.65 to -1.30)	-1.61(-1.78 to -1.44)
**Nicaragua**	2.12(1.81 - 2.44)	0.14(-0.23 - 0.52)	0.58(0.36 - 0.79)

**Niger**	0.03(-0.06 - 0.11)	-1.61(-1.75 to -1.48)	2.13(1.96 - 2.30)
**Nigeria**	1.29(1.21 - 1.38)	0.75(0.63 - 0.87)	1.17(1.05 - 1.30)
**Niue**	0.70(0.50 - 0.89)	0.32(0.25 - 0.39)	0.62(0.57 - 0.67)
**North Macedonia**	4.18(3.66 - 4.71)	0.66(0.46 - 0.86)	1.53(1.32 - 1.74)
**Northern Mariana Islands**	-0.32(-0.77 - 0.14)	3.22(2.62 - 3.84)	1.20(0.98 - 1.42)
**Norway**	0.29(0.10 - 0.47)	-1.10(-1.16 to -1.04)	-0.90(-1.08 to -0.73)
**Oman**	2.94(2.62 - 3.25)	0.70(0.51 - 0.90)	0.48(0.36 - 0.60)
**Pakistan**	2.01(1.76 - 2.26)	0.48(0.35 - 0.60)	0.55(0.41 - 0.69)
**Palau**	0.69(0.50 - 0.89)	0.05(-0.01 - 0.10)	-0.08(-0.15 to -0.01)
**Palestine**	1.11(0.92 - 1.30)	-0.56(-0.63 to -0.49)	-0.15(-0.28 to -0.01)
**Panama**	2.57(2.37 - 2.77)	-1.19(-1.38 to -1.01)	0.47(0.02 - 0.93)
**Papua New Guinea**	0.56(0.47 - 0.65)	0.92(0.87 - 0.96)	1.08(1.02 - 1.14)
**Paraguay**	0.43(0.30 - 0.55)	0.61(0.49 - 0.73)	1.18(0.78 - 1.59)
**Peru**	0.01(-0.23 - 0.25)	-0.70(-0.86 to -0.53)	0.21(0.07 - 0.36)
**Philippines**	0.59(0.39 - 0.79)	-0.78(-1.01 to -0.55)	-0.20(-0.40 - 0.01)
**Poland**	3.76(2.78 - 4.74)	0.44(0.29 - 0.60)	0.67(0.38 - 0.96)
**Portugal**	0.05(-0.16 - 0.25)	-0.46(-0.53 to -0.39)	-1.56(-1.76 to -1.36)
**Puerto Rico**	-0.23(-0.44 to -0.01)	-0.42(-0.56 to -0.29)	-1.41(-1.54 to -1.28)
**Qatar**	1.12(0.80 - 1.44)	1.99(1.70 - 2.28)	1.02(0.57 - 1.47)
**Republic of Korea**	1.77(1.16 - 2.39)	-1.30(-1.53 to -1.08)	0.14(-0.05 - 0.32)
**Republic of Moldova**	2.01(1.63 - 2.39)	-0.31(-0.59 to -0.02)	1.39(0.98 - 1.81)
**Romania**	2.04(1.95 - 2.13)	0.68(0.56 - 0.80)	0.88(0.76 - 1.00)

**Russian Federation**	0.25(0.02 - 0.48)	-0.62(-0.97 to -0.26)	1.48(1.35 - 1.60)
**Rwanda**	0.51(0.27 - 0.75)	-1.61(-1.84 to -1.37)	-0.13(-0.33 - 0.08)
**Saint Kitts and Nevis**	-1.63(-2.15 to -1.10)	-0.09(-0.27 - 0.08)	0.64(0.52 - 0.77)
**Saint Lucia**	-1.32(-1.77 to -0.87)	-0.63(-0.88 to -0.37)	-0.64(-0.88 to -0.40)
**Saint Vincent and the Grenadines**	-0.85(-1.37 to -0.34)	0.56(0.31 - 0.81)	0.41(0.19 - 0.62)
**Samoa**	-0.19(-0.30 to -0.09)	0.03(-0.02 - 0.08)	-0.57(-0.76 to -0.38)
**San Marino**	0.67(0.58 - 0.75)	-0.05(-0.13 - 0.04)	-0.51(-0.55 to -0.46)
**Sao Tome and Principe**	1.30(1.06 - 1.53)	1.18(1.11 - 1.25)	1.39(1.23 - 1.55)
**Saudi Arabia**	2.57(2.32 - 2.83)	-1.11(-1.46 to -0.77)	-1.04(-1.28 to -0.80)
**Senegal**	0.89(0.72 - 1.06)	-1.34(-1.44 to -1.25)	2.09(1.90 - 2.27)
**Serbia**	1.39(1.24 - 1.54)	0.67(0.57 - 0.78)	1.39(1.13 - 1.65)
**Seychelles**	0.75(0.30 - 1.21)	0.14(-0.06 - 0.34)	1.05(0.72 - 1.38)
**Sierra Leone**	0.81(0.65 - 0.97)	-2.25(-2.42 to -2.08)	1.94(1.77 - 2.12)
**Singapore**	0.14(-0.01 - 0.30)	-2.31(-2.55 to -2.07)	-0.63(-0.81 to -0.45)
**Slovakia**	1.79(1.44 - 2.13)	0.05(-0.2 - 0.31)	0.73(0.57 - 0.88)
**Slovenia**	1.89(1.56 - 2.22)	-0.21(-0.36 to -0.05)	0.67(0.38 - 0.95)
**Solomon Islands**	0.52(0.29 - 0.75)	0.71(0.64 - 0.77)	0.67(0.63 - 0.71)
**Somalia**	0.80(0.71 - 0.89)	-0.38(-0.41 to -0.34)	-0.07(-0.13 to -0.02)
**South Africa**	0.73(0.53 - 0.93)	-0.20(-0.48 - 0.08)	0.64(0.42 - 0.86)
**South Sudan**	0.82(0.74 - 0.90)	-0.33(-0.39 to -0.27)	-0.04(-0.07 - 0.01)
**Spain**	0.78(0.72 - 0.83)	-0.73(-0.79 to -0.67)	-1.6(-1.74 to -1.46)
**Sri Lanka**	-2.14(-3.12 to -1.14)	1.28(1.00 - 1.55)	0.17(0.03 - 0.31)

**Sudan**	2.72(2.40 - 3.04)	-0.55(-0.70 to -0.41)	0.68(0.61 - 0.74)
**Suriname**	-0.27(-0.69 - 0.16)	0.58(0.35 - 0.81)	0.83(0.61 - 1.05)
**Sweden**	-1.14(-1.26 to -1.02)	0.05(-0.02 - 0.11)	-1.15(-1.39 to -0.92)
**Switzerland**	0.65(0.09 - 1.21)	0.23(-0.09 - 0.56)	-2.05(-2.18 to -1.92)
**Syrian Arab Republic**	1.24(1.05 - 1.43)	0.26(0.15 - 0.37)	0.12(-0.01 - 0.26)
**Taiwan (Province of China)**	3.80(3.14 - 4.46)	-0.06(-0.23 - 0.11)	1.23(1.15 - 1.32)
**Tajikistan**	0.87(0.57 - 1.16)	1.10(0.98 - 1.23)	1.27(1.18 - 1.37)
**Thailand**	-0.08(-0.41 - 0.24)	-2.73(-3.06 to -2.40)	-0.73(-0.86 to -0.61)
**Timor-Leste**	2.15(1.92 - 2.39)	0.42(0.19 - 0.65)	1.83(1.69 - 1.96)
**Togo**	0.63(0.55 - 0.71)	-2.19(-2.36 to -2.02)	1.96(1.81 - 2.11)
**Tokelau**	0.80(0.71 - 0.89)	0.18(0.16 - 0.20)	0.29(0.24 - 0.34)
**Tonga**	0.64(0.18 - 1.11)	0.65(0.50 - 0.80)	0.15(-0.02 - 0.32)
**Trinidad and Tobago**	-2.35(-2.88 to -1.82)	-0.43(-0.79 to -0.07)	-0.56(-0.76 to -0.36)
**Tunisia**	1.44(1.40 - 1.48)	-0.43(-0.54 to -0.33)	0.25(0.19 - 0.30)
**Turkey**	0.58(0.50 - 0.66)	-0.18(-0.35 to -0.02)	-0.15(-0.66 - 0.37)
**Turkmenistan**	3.61(3.18 - 4.05)	1.34(1.12 - 1.56)	0.09(0.01 - 0.18)
**Tuvalu**	0.21(0.07 - 0.35)	0.20(0.17 - 0.24)	0.03(-0.05 - 0.11)
**Uganda**	3.16(2.90 - 3.42)	0.11(0.03 - 0.20)	1.05(0.88 - 1.21)
**Ukraine**	0.92(0.70 - 1.13)	0.14(-0.08 - 0.36)	-0.18(-0.39 - 0.04)
**United Arab Emirates**	1.63(1.31 - 1.95)	-1.18(-1.63 to -0.73)	-0.39(-0.69 to -0.08)
**United Kingdom**	0.39(0.32 - 0.45)	-1.06(-1.22 to -0.90)	-0.80(-0.89 to -0.70)
**United Republic of Tanzania**	1.89(1.78 - 2.01)	-0.47(-0.54 to -0.39)	0.10(0.07 - 0.13)

**United States of America**	-0.32(-0.39 to -0.25)	0.17(0.14 - 0.19)	-1.78(-1.94 to -1.62)
**United States Virgin Islands**	1.22(0.92 - 1.51)	1.54(1.19 - 1.89)	1.15(0.95 - 1.36)
**Uruguay**	-0.02(-0.16 - 0.12)	-0.81(-0.91 to -0.72)	-0.11(-0.41 - 0.20)
**Uzbekistan**	3.01(2.81 - 3.20)	1.82(1.50 - 2.14)	1.83(1.47 - 2.19)
**Vanuatu**	0.53(0.42 - 0.64)	1.04(0.92 - 1.15)	0.63(0.58 - 0.69)
**Venezuela (Bolivarian Republic of)**	0.16(-0.20 - 0.52)	-0.02(-0.18 - 0.15)	0.60(0.16 - 1.05)
**Viet Nam**	2.05(1.96 - 2.15)	0.68(0.63 - 0.74)	0.87(0.83 - 0.91)
**Yemen**	1.64(1.52 - 1.76)	0.16(0.08 - 0.24)	0.86(0.77 - 0.95)
**Zambia**	1.33(1.27 - 1.38)	-0.47(-0.52 to -0.41)	0.17(0.07 - 0.27)
**Zimbabwe**	1.12(0.70 - 1.54)	0.54(0.32 - 0.76)	0.73(0.38 - 1.08)

ASMR age-standardized mortality rate; *EAPC* estimated annual percentage change; *CI* confidence interval

Table S8 EAPC of ASDR for genitourinary cancers in 204 countries and territories from 1990 to 2019

Location	EAPC (95% CI)		
	Kidney cancer	Bladder cancer	Prostate cancer
**Afghanistan**	0.62(0.41 - 0.84)	-0.52(-0.54 to -0.49)	0.23(0.18 - 0.29)
**Albania**	2.13(1.80 - 2.46)	-0.30(-0.47 to -0.14)	-0.10(-0.23 - 0.03)
**Algeria**	0.66(0.57 - 0.76)	-0.75(-1.03 to -0.48)	-0.67(-0.72 to -0.62)
**American Samoa**	0.03(-0.32 - 0.39)	1.63(1.33 - 1.93)	0.23(0.10 - 0.35)
**Andorra**	0.07(-0.01 - 0.15)	-0.93(-0.98 to -0.88)	-0.45(-0.48 to -0.42)
**Angola**	0.86(0.78 - 0.95)	-0.42(-0.46 to -0.37)	0.61(0.58 - 0.65)
**Antigua and Barbuda**	-0.66(-1.07 to -0.24)	0.39(0.17 - 0.61)	0.01(-0.33 - 0.35)
**Argentina**	-0.75(-1.05 to -0.45)	-1.14(-1.24 to -1.04)	-0.28(-0.53 to -0.02)
**Armenia**	5.22(4.37 - 6.09)	-0.22(-0.46 - 0.02)	1.58(1.46 - 1.70)
**Australia**	-0.65(-0.73 to -0.56)	-1.43(-1.56 to -1.29)	-1.27(-1.44 to -1.10)
**Austria**	-1.73(-1.85 to -1.61)	-1.11(-1.20 to -1.03)	-1.28(-1.43 to -1.14)
**Azerbaijan**	-0.11(-0.38 - 0.17)	0.22(0.13 - 0.31)	0.61(0.51 - 0.71)
**Bahamas**	-0.74(-1.00 to -0.47)	0.10(-0.04 - 0.23)	-0.02(-0.13 - 0.09)
**Bahrain**	-1.14(-1.56 to -0.72)	-2.31(-2.53 to -2.09)	-0.67(-0.92 to -0.42)
**Bangladesh**	0.46(0.38 - 0.54)	-0.48(-0.61 to -0.35)	-0.60(-0.74 to -0.47)
**Barbados**	-1.01(-1.34 to -0.67)	0.27(0.18 - 0.36)	-0.01(-0.31 - 0.29)
**Belarus**	3.55(2.58 - 4.52)	-1.59(-2.05 to -1.13)	0.86(0.68 - 1.04)
**Belgium**	-0.38(-0.61 to -0.15)	-1.34(-1.58 to -1.10)	-1.86(-2.03 to -1.69)

**Belize**	-0.32(-0.44 to -0.19)	0.91(0.55 - 1.26)	0.82(0.27 - 1.38)
**Benin**	1.49(1.33 - 1.65)	-1.89(-2.02 to -1.75)	1.69(1.53 - 1.86)
**Bermuda**	-2.03(-2.46 to -1.60)	-1.06(-1.27 to -0.85)	-0.26(-0.34 to -0.19)
**Bhutan**	1.83(1.75 - 1.91)	0.40(0.36 - 0.45)	0.91(0.85 - 0.97)
**Bolivia (Plurinational State of)**	1.07(1.01 - 1.12)	-0.13(-0.23 to -0.03)	0.69(0.64 - 0.75)
**Bosnia and Herzegovina**	2.04(1.81 - 2.27)	1.32(1.13 - 1.51)	1.26(1.05 - 1.48)
**Botswana**	1.51(1.08 - 1.93)	-0.32(-0.64 to -0.01)	0.11(-0.35 - 0.58)
**Brazil**	0.78(0.69 - 0.87)	-0.52(-0.57 to -0.48)	-0.29(-0.54 to -0.04)
**Brunei Darussalam**	1.45(1.21 - 1.68)	-0.45(-0.63 to -0.27)	1.52(1.13 - 1.91)
**Bulgaria**	5.71(4.80 - 6.62)	2.06(1.67 - 2.45)	2.03(1.74 - 2.33)
**Burkina Faso**	1.45(1.32 - 1.58)	-2.15(-2.42 to -1.87)	1.84(1.75 - 1.93)
**Burundi**	0.04(-0.04 - 0.12)	-1.76(-1.90 to -1.62)	-0.32(-0.38 to -0.27)
**Cabo Verde**	4.53(3.76 - 5.31)	3.01(2.22 - 3.80)	2.45(1.65 - 3.26)
**Cambodia**	1.18(1.13 - 1.24)	-0.31(-0.49 to -0.13)	1.05(0.98 - 1.12)
**Cameroon**	0.87(0.82 - 0.92)	-1.03(-1.14 to -0.91)	1.85(1.69 - 2.00)
**Canada**	0.69(0.46 - 0.91)	-1.36(-1.47 to -1.26)	-2.35(-2.61 to -2.08)
**Central African Republic**	0.22(0.12 - 0.32)	-0.95(-1.01 to -0.90)	-0.05(-0.09 - 0.01)
**Chad**	1.30(1.23 - 1.37)	-0.58(-0.71 to -0.44)	2.20(1.99 - 2.40)
**Chile**	0.29(0.15 - 0.44)	-0.10(-0.18 to -0.03)	0.13(-0.07 - 0.34)
**China**	2.28(1.85 - 2.71)	-0.58(-0.64 to -0.53)	-0.24(-0.27 to -0.20)
**Colombia**	0.66(0.56 - 0.76)	-1.74(-1.87 to -1.60)	-1.12(-1.36 to -0.89)
**Comoros**	0.94(0.79 - 1.08)	-0.53(-0.69 to -0.37)	0.06(-0.04 - 0.15)

**Congo**	0.18(0.01 - 0.36)	-0.80(-1.01 to -0.59)	-0.21(-0.37 to -0.05)
**Cook Islands**	0.18(0.07 - 0.29)	-0.15(-0.33 - 0.03)	-0.85(-0.90 to -0.80)
**Costa Rica**	1.86(1.63 - 2.09)	-0.59(-0.74 to -0.44)	0.82(0.55 - 1.08)
**Croatia**	3.37(2.74 - 3.99)	0.22(0.11 - 0.34)	0.24(0.08 - 0.39)
**Cuba**	-1.16(-1.63 to -0.69)	0.55(0.41 - 0.69)	0.79(0.72 - 0.86)
**Cyprus**	2.58(2.27 - 2.89)	-0.15(-0.31 - 0.01)	0.19(-0.10 - 0.48)
**Czechia**	0.09(-0.34 - 0.52)	-0.78(-0.87 to -0.69)	-0.19(-0.46 - 0.09)
**Cote d'Ivoire**	0.80(0.71 - 0.89)	-1.83(-1.99 to -1.66)	1.52(1.38 - 1.66)
**Democratic People's Republic of Korea**	0.39(0.31 - 0.48)	-0.38(-0.43 to -0.34)	0.16(0.07 - 0.24)
**Democratic Republic of the Congo**	-0.38(-0.65 to -0.10)	-1.21(-1.40 to -1.03)	0.10(0.06 - 0.15)
**Denmark**	0.38(-0.17 - 0.93)	-1.19(-1.63 to -0.75)	-0.47(-0.69 to -0.24)
**Djibouti**	2.09(1.96 - 2.21)	0.12(0.08 - 0.17)	0.42(0.37 - 0.47)
**Dominica**	-0.09(-0.42 - 0.25)	0.60(0.48 - 0.72)	0.52(0.38 - 0.65)
**Dominican Republic**	1.66(1.05 - 2.27)	1.47(1.28 - 1.67)	1.50(1.01 - 2.00)
**Ecuador**	1.53(1.14 - 1.92)	0.28(0.01 - 0.56)	0.60(0.40 - 0.80)
**Egypt**	1.46(1.35 - 1.56)	0.14(0.07 - 0.21)	0.35(0.23 - 0.47)
**El Salvador**	0.97(0.85 - 1.10)	0.16(0.04 - 0.28)	0.88(0.47 - 1.28)
**Equatorial Guinea**	3.29(3.08 - 3.51)	0.55(0.35 - 0.75)	0.94(0.81 - 1.06)
**Eritrea**	1.54(1.26 - 1.83)	-0.07(-0.27 - 0.14)	-0.05(-0.29 - 0.19)
**Estonia**	3.02(2.25 - 3.79)	-0.51(-0.69 to -0.34)	1.46(1.22 - 1.71)
**Eswatini**	1.56(0.85 - 2.26)	0.38(0.09 - 0.66)	0.86(0.58 - 1.15)
**Ethiopia**	0.02(-0.24 - 0.29)	-0.98(-1.10 to -0.87)	0.52(0.40 - 0.63)

**Fiji**	0.50(0.38 - 0.62)	1.16(0.87 - 1.46)	0.60(0.43 - 0.77)
**Finland**	-0.65(-0.72 to -0.57)	-1.67(-1.76 to -1.58)	-1.06(-1.25 to -0.87)
**France**	-0.41(-0.51 to -0.32)	-0.99(-1.03 to -0.96)	-1.98(-2.19 to -1.77)
**Gabon**	1.33(1.23 - 1.43)	-0.49(-0.57 to -0.42)	0.46(0.41 - 0.50)
**Gambia**	1.07(0.86 - 1.27)	0.43(0.28 - 0.59)	0.71(0.59 - 0.82)
**Georgia**	1.83(1.39 - 2.27)	2.00(1.66 - 2.35)	2.67(2.07 - 3.27)
**Germany**	-0.69(-0.80 to -0.57)	-1.44(-1.70 to -1.18)	-0.89(-1.03 to -0.75)
**Ghana**	-0.70(-1.09 to -0.30)	-1.14(-1.31 to -0.96)	-0.33(-0.52 to -0.15)
**Greece**	-0.03(-0.15 - 0.10)	-0.97(-1.10 to -0.84)	-0.96(-1.20 to -0.73)
**Greenland**	1.08(0.74 - 1.43)	-1.27(-1.37 to -1.17)	-0.05(-0.13 - 0.02)
**Grenada**	-0.05(-0.55 - 0.45)	0.65(0.50 - 0.81)	0.87(0.15 - 1.58)
**Guam**	-0.69(-1.01 to -0.37)	0.64(0.39 - 0.90)	-0.64(-0.93 to -0.34)
**Guatemala**	1.38(0.82 - 1.94)	-0.49(-0.61 to -0.38)	1.52(0.91 - 2.15)
**Guinea**	0.92(0.83 - 1.01)	0.51(0.42 - 0.60)	1.00(0.85 - 1.15)
**Guinea-Bissau**	0.30(0.15 - 0.45)	-1.42(-1.51 to -1.32)	1.80(1.65 - 1.94)
**Guyana**	-0.31(-0.57 to -0.05)	0.28(0.20 - 0.36)	0.20(0.06 - 0.35)
**Haiti**	-0.60(-0.92 to -0.27)	-0.29(-0.43 to -0.14)	0.14(0.10 - 0.18)
**Honduras**	1.90(1.78 - 2.02)	1.51(1.33 - 1.68)	1.73(1.40 - 2.05)
**Hungary**	-0.36(-0.65 to -0.07)	-0.06(-0.24 - 0.13)	-0.69(-0.88 to -0.51)
**Iceland**	-0.25(-0.45 to -0.05)	-1.52(-1.64 to -1.40)	-1.07(-1.30 to -0.85)
**India**	1.26(1.18 - 1.34)	-0.22(-0.31 to -0.13)	-0.30(-0.38 to -0.22)
**Indonesia**	1.98(1.92 - 2.03)	0.31(0.25 - 0.36)	1.75(1.67 - 1.82)

**Iran (Islamic Republic of)**	0.92(0.76 - 1.07)	0.11(0.05 - 0.18)	0.53(0.34 - 0.73)
**Iraq**	1.39(1.17 - 1.62)	0.77(0.56 - 0.98)	0.59(0.45 - 0.74)
**Ireland**	0.48(0.23 - 0.74)	-0.81(-0.98 to -0.63)	-1.21(-1.45 to -0.97)
**Israel**	-0.40(-0.68 to -0.11)	-0.53(-0.73 to -0.33)	-1.54(-1.82 to -1.26)
**Italy**	-0.56(-0.60 to -0.52)	-1.57(-1.65 to -1.49)	-1.07(-1.25 to -0.89)
**Jamaica**	-0.96(-1.43 to -0.49)	-0.19(-0.49 - 0.11)	1.98(1.41 - 2.56)
**Japan**	-0.02(-0.15 - 0.11)	-0.64(-0.70 to -0.59)	-0.33(-0.44 to -0.23)
**Jordan**	2.06(1.88 - 2.24)	-0.51(-0.60 to -0.41)	0.23(0.04 - 0.42)
**Kazakhstan**	-0.20(-0.67 - 0.28)	-0.78(-1.26 to -0.29)	0.62(0.47 - 0.77)
**Kenya**	2.68(2.46 - 2.90)	1.39(1.19 - 1.59)	1.94(1.73 - 2.16)
**Kiribati**	0.12(-0.13 - 0.37)	-0.28(-0.32 to -0.24)	-0.62(-0.82 to -0.42)
**Kuwait**	-0.28(-0.81 - 0.25)	-0.41(-0.76 to -0.06)	0.79(0.50 - 1.09)
**Kyrgyzstan**	0.97(0.49 - 1.46)	-0.76(-1.10 to -0.42)	-1.36(-1.59 to -1.12)
**Lao People's Democratic Republic**	0.59(0.57 - 0.62)	-1.11(-1.26 to -0.95)	0.23(0.18 - 0.28)
**Latvia**	3.04(2.34 - 3.74)	0.33(0.08 - 0.59)	1.73(1.36 - 2.09)
**Lebanon**	2.53(2.27 - 2.78)	-0.43(-0.48 to -0.37)	0.93(0.79 - 1.06)
**Lesotho**	3.05(2.85 - 3.25)	1.51(1.32 - 1.71)	1.36(1.11 - 1.62)
**Liberia**	0.51(-0.16 - 1.17)	-1.99(-2.10 to -1.88)	1.67(1.50 - 1.84)
**Libya**	1.29(1.16 - 1.43)	0.14(0.03 - 0.26)	0.33(0.25 - 0.41)
**Lithuania**	3.01(2.28 - 3.75)	-0.81(-1.06 to -0.57)	1.92(1.38 - 2.47)
**Luxembourg**	-1.16(-1.25 to -1.08)	-1.27(-1.33 to -1.21)	-2.04(-2.14 to -1.94)
**Madagascar**	0.56(0.48 - 0.64)	-1.22(-1.38 to -1.05)	-0.36(-0.50 to -0.21)

**Malawi**	0.77(0.67 - 0.87)	-0.29(-0.40 to -0.18)	0.95(0.82 - 1.08)
**Malaysia**	1.51(1.41 - 1.60)	-0.55(-0.81 to -0.30)	0.08(-0.07 - 0.24)
**Maldives**	0.40(0.14 - 0.67)	-1.74(-1.88 to -1.59)	-0.06(-0.14 - 0.01)
**Mali**	1.33(1.20 - 1.46)	-0.24(-0.31 to -0.17)	0.66(0.62 - 0.71)
**Malta**	0.27(0.13 - 0.42)	-1.46(-1.54 to -1.39)	-1.24(-1.40 to -1.09)
**Marshall Islands**	0.63(0.49 - 0.78)	0.58(0.46 - 0.70)	-0.07(-0.31 - 0.16)
**Mauritania**	0.17(0.10 - 0.24)	-2.17(-2.27 to -2.07)	1.32(1.16 - 1.48)
**Mauritius**	1.70(1.45 - 1.95)	-1.78(-2.05 to -1.52)	0.18(-0.13 - 0.50)
**Mexico**	0.93(0.87 - 1.00)	-0.22(-0.3 to -0.14)	-0.11(-0.22 - 0.01)
**Micronesia (Federated States of)**	0.23(0.08 - 0.38)	0.56(0.52 - 0.61)	0.48(0.39 - 0.57)
**Monaco**	0.66(0.51 - 0.82)	0.51(0.46 - 0.56)	-0.24(-0.30 to -0.17)
**Mongolia**	3.01(2.71 - 3.30)	-3.22(-3.70 to -2.75)	-0.06(-0.20 - 0.08)
**Montenegro**	1.03(0.96 - 1.10)	0.34(0.25 - 0.43)	0.78(0.71 - 0.84)
**Morocco**	1.52(1.36 - 1.69)	0.49(0.28 - 0.71)	0.67(0.34 - 0.99)
**Mozambique**	2.32(2.16 - 2.49)	-0.02(-0.15 - 0.10)	1.26(1.19 - 1.32)
**Myanmar**	1.26(1.15 - 1.37)	-0.99(-1.04 to -0.93)	0.62(0.60 - 0.65)
**Namibia**	1.65(1.43 - 1.88)	0.12(-0.10 - 0.34)	2.00(1.86 - 2.13)
**Nauru**	-0.38(-0.57 to -0.19)	0.28(0.12 - 0.44)	0.53(0.38 - 0.69)
**Nepal**	2.08(1.81 - 2.34)	0.21(0.07 - 0.36)	0.84(0.69 - 1.00)
**Netherlands**	-0.07(-0.22 - 0.08)	-0.63(-0.78 to -0.49)	-0.85(-1.01 to -0.68)
**New Zealand**	-0.09(-0.23 - 0.06)	-1.72(-1.90 to -1.53)	-1.60(-1.76 to -1.43)
**Nicaragua**	1.50(1.30 - 1.70)	-0.14(-0.41 - 0.13)	0.65(0.47 - 0.83)

**Niger**	-0.52(-0.66 to -0.38)	-1.81(-1.96 to -1.65)	1.97(1.82 - 2.12)
**Nigeria**	0.62(0.50 - 0.75)	0.52(0.41 - 0.63)	1.09(0.97 - 1.21)
**Niue**	0.67(0.43 - 0.91)	0.26(0.20 - 0.32)	0.61(0.56 - 0.66)
**North Macedonia**	3.97(3.46 - 4.48)	0.48(0.29 - 0.68)	1.41(1.24 - 1.57)
**Northern Mariana Islands**	-0.31(-0.78 - 0.17)	3.37(2.73 - 4.02)	1.22(1.00 - 1.43)
**Norway**	0.01(-0.18 - 0.21)	-1.37(-1.44 to -1.30)	-0.89(-1.08 to -0.70)
**Oman**	2.44(2.06 - 2.82)	0.35(0.11 - 0.59)	0.40(0.28 - 0.52)
**Pakistan**	2.03(1.80 - 2.27)	0.50(0.36 - 0.64)	0.58(0.43 - 0.72)
**Palau**	0.67(0.46 - 0.88)	0.07(0.01 - 0.13)	-0.09(-0.16 to -0.02)
**Palestine**	1.00(0.81 - 1.20)	-0.38(-0.44 to -0.32)	-0.18(-0.29 to -0.07)
**Panama**	2.58(2.36 - 2.81)	-1.23(-1.40 to -1.07)	0.38(0.01 - 0.78)
**Papua New Guinea**	0.56(0.46 - 0.67)	0.90(0.84 - 0.96)	0.99(0.93 - 1.04)
**Paraguay**	0.13(-0.02 - 0.29)	0.61(0.49 - 0.73)	1.21(0.84 - 1.59)
**Peru**	-0.52(-0.81 to -0.23)	-0.83(-0.99 to -0.68)	0.19(0.06 - 0.33)
**Philippines**	0.57(0.39 - 0.75)	-0.75(-0.97 to -0.53)	-0.20(-0.39 to -0.01)
**Poland**	3.25(2.29 - 4.22)	0.25(0.10 - 0.40)	0.53(0.27 - 0.80)
**Portugal**	-0.44(-0.65 to -0.23)	-0.31(-0.37 to -0.25)	-1.38(-1.57 to -1.19)
**Puerto Rico**	-0.31(-0.52 to -0.10)	-0.31(-0.46 to -0.16)	-1.20(-1.32 to -1.08)
**Qatar**	0.22(-0.12 - 0.57)	1.32(1.06 - 1.58)	0.78(0.47 - 1.09)
**Republic of Korea**	1.22(0.64 - 1.82)	-1.62(-1.82 to -1.41)	0.12(-0.05 - 0.30)
**Republic of Moldova**	1.70(1.34 - 2.05)	-0.27(-0.55 - 0.01)	1.82(1.35 - 2.30)
**Romania**	1.61(1.52 - 1.69)	0.45(0.32 - 0.58)	1.00(0.89 - 1.11)

**Russian Federation**	-0.25(-0.52 - 0.02)	-0.89(-1.28 to -0.50)	1.52(1.38 - 1.66)
**Rwanda**	0.18(-0.05 - 0.40)	-1.96(-2.23 to -1.68)	-0.40(-0.65 to -0.15)
**Saint Kitts and Nevis**	-1.90(-2.45 to -1.34)	-0.40(-0.59 to -0.22)	0.38(0.23 - 0.54)
**Saint Lucia**	-1.24(-1.66 to -0.81)	-0.55(-0.80 to -0.30)	-0.49(-0.69 to -0.29)
**Saint Vincent and the Grenadines**	-1.02(-1.47 to -0.56)	0.45(0.23 - 0.67)	0.26(0.04 - 0.49)
**Samoa**	-0.37(-0.50 to -0.25)	0.01(-0.04 - 0.06)	-0.66(-0.85 to -0.47)
**San Marino**	0.64(0.56 - 0.72)	-0.09(-0.16 to -0.02)	-0.36(-0.40 to -0.32)
**Sao Tome and Principe**	0.13(-0.32 - 0.58)	1.06(0.98 - 1.13)	1.45(1.35 - 1.56)
**Saudi Arabia**	2.48(2.26 - 2.70)	-0.92(-1.21 to -0.62)	-0.86(-1.08 to -0.63)
**Senegal**	0.44(0.20 - 0.67)	-1.36(-1.48 to -1.24)	1.98(1.79 - 2.18)
**Serbia**	1.06(0.92 - 1.20)	0.47(0.38 - 0.55)	1.21(0.99 - 1.44)
**Seychelles**	0.64(0.18 - 1.10)	-0.07(-0.26 - 0.13)	0.97(0.65 - 1.29)
**Sierra Leone**	0.68(0.47 - 0.90)	-2.17(-2.34 to -2.00)	1.86(1.69 - 2.03)
**Singapore**	-0.13(-0.27 - 0.01)	-2.50(-2.72 to -2.29)	-0.56(-0.70 to -0.42)
**Slovakia**	1.46(1.11 - 1.82)	-0.20(-0.45 - 0.04)	0.63(0.49 - 0.77)
**Slovenia**	1.52(1.18 - 1.86)	-0.52(-0.72 to -0.32)	0.64(0.33 - 0.95)
**Solomon Islands**	0.56(0.31 - 0.80)	0.75(0.68 - 0.82)	0.56(0.50 - 0.61)
**Somalia**	0.67(0.58 - 0.77)	-0.42(-0.45 to -0.39)	-0.10(-0.15 to -0.04)
**South Africa**	0.30(0.13 - 0.46)	-0.38(-0.64 to -0.11)	0.63(0.39 - 0.87)
**South Sudan**	0.49(0.38 - 0.61)	-0.48(-0.56 to -0.41)	-0.12(-0.15 to -0.09)
**Spain**	0.46(0.40 - 0.51)	-0.94(-1.04 to -0.84)	-1.52(-1.67 to -1.37)
**Sri Lanka**	-2.13(-3.12 to -1.12)	1.24(0.99 - 1.48)	0.20(0.07 - 0.32)

**Sudan**	2.34(2.03 - 2.65)	-0.62(-0.76 to -0.47)	0.62(0.56 - 0.69)
**Suriname**	-0.43(-0.86 - 0.01)	0.58(0.36 - 0.79)	0.92(0.70 - 1.15)
**Sweden**	-1.47(-1.60 to -1.35)	-0.15(-0.24 to -0.06)	-1.10(-1.33 to -0.87)
**Switzerland**	0.25(-0.31 - 0.82)	-0.07(-0.39 - 0.25)	-1.98(-2.06 to -1.90)
**Syrian Arab Republic**	1.19(0.99 - 1.39)	0.11(0.02 - 0.20)	0.01(-0.16 - 0.16)
**Taiwan (Province of China)**	3.40(2.76 - 4.05)	-0.32(-0.49 to -0.16)	1.26(1.17 - 1.36)
**Tajikistan**	0.36(0.02 - 0.71)	0.66(0.49 - 0.83)	0.73(0.61 - 0.86)
**Thailand**	-0.30(-0.70 - 0.10)	-3.02(-3.40 to -2.64)	-0.77(-0.92 to -0.63)
**Timor-Leste**	1.70(1.42 - 1.98)	0.15(-0.11 - 0.42)	1.70(1.56 - 1.84)
**Togo**	0.48(0.39 - 0.58)	-2.07(-2.25 to -1.90)	1.89(1.75 - 2.04)
**Tokelau**	0.72(0.59 - 0.84)	0.20(0.17 - 0.23)	0.23(0.19 - 0.28)
**Tonga**	0.67(0.18 - 1.15)	0.71(0.56 - 0.85)	0.20(0.03 - 0.37)
**Trinidad and Tobago**	-2.32(-2.84 to -1.79)	-0.25(-0.59 - 0.09)	-0.61(-0.81 to -0.42)
**Tunisia**	1.29(1.26 - 1.32)	-0.34(-0.44 to -0.24)	0.34(0.30 - 0.38)
**Turkey**	0.20(0.14 - 0.25)	-0.34(-0.54 to -0.14)	-0.24(-0.64 - 0.16)
**Turkmenistan**	3.60(3.15 - 4.05)	1.89(1.64 - 2.13)	0.17(0.06 - 0.29)
**Tuvalu**	0.06(-0.07 - 0.18)	0.10(0.07 - 0.13)	-0.12(-0.21 to -0.03)
**Uganda**	2.97(2.71 - 3.23)	0.01(-0.12 - 0.13)	0.96(0.79 - 1.13)
**Ukraine**	0.92(0.68 - 1.15)	0.27(0.02 - 0.52)	0.07(-0.14 - 0.28)
**United Arab Emirates**	1.52(1.28 - 1.77)	-1.06(-1.45 to -0.67)	-0.33(-0.63 to -0.02)
**United Kingdom**	-0.04(-0.12 - 0.04)	-1.51(-1.68 to -1.35)	-0.82(-0.90 to -0.73)
**United Republic of Tanzania**	1.81(1.67 - 1.95)	-0.48(-0.55 to -0.41)	0.08(0.04 - 0.11)

**United States of America**	-0.65(-0.73 to -0.57)	-0.14(-0.18 to -0.09)	-1.70(-1.86 to -1.55)
**United States Virgin Islands**	0.93(0.71 - 1.15)	1.60(1.22 - 1.98)	1.42(1.15 - 1.69)
**Uruguay**	-0.31(-0.49 to -0.13)	-0.99(-1.11 to -0.88)	-0.12(-0.45 - 0.20)
**Uzbekistan**	2.60(2.43 - 2.77)	1.40(1.08 - 1.71)	1.40(1.12 - 1.67)
**Vanuatu**	0.68(0.56 - 0.80)	1.11(0.98 - 1.24)	0.59(0.54 - 0.64)
**Venezuela (Bolivarian Republic of)**	0.04(-0.34 - 0.41)	-0.02(-0.18 - 0.13)	0.79(0.37 - 1.20)
**Viet Nam**	2.11(1.99 - 2.24)	0.72(0.66 - 0.78)	0.78(0.75 - 0.81)
**Yemen**	1.47(1.34 - 1.60)	-0.03(-0.11 - 0.04)	0.85(0.75 - 0.94)
**Zambia**	1.15(1.11 - 1.19)	-0.50(-0.57 to -0.43)	0.18(0.06 - 0.31)
**Zimbabwe**	1.19(0.82 - 1.57)	0.63(0.41 - 0.86)	0.88(0.50 - 1.25)

ASDR age-standardized DALYs rate; *EAPC* estimated annual percentage change; *CI* confidence interval

Table S9 Percentage of genitourinary cancers deaths and DALYs attributable to risk factors in 1990 and 2019

Cancer	1990			2019		
	**Leading risk**	**Percentage of death (95% UI)**	**Percentage of DALYs (95% UI)**	**Leading risk**	**Percentage of death (95% UI)**	**Percentage of DALYs (95% UI)**
**Kidney cancer**	Smoking	20.78(15.00 - 26.43)	18.19(13.10 - 23.03)	High BMI	19.05(11.09 - 28.30)	18.55(10.92 - 27.37)
	High BMI	15.53(8.52 - 23.90)	13.98(7.72 - 21.74)	Smoking	18.09(12.81 - 23.44)	16.95(11.78 - 21.79)
	Occupational exposure to trichloroethylene	0.03(0.01 - 0.06)	0.04(0.01 - 0.07)	Occupational exposure to trichloroethylene	0.05(0.01 - 0.09)	0.06(0.01 - 0.11)
**Bladder cancer**	Smoking	41.05(32.07 - 49.14)	42.86(34.04 - 50.23)	Smoking	33.89(25.88 - 41.80)	36.76(44.00 - 28.46)
	High FPG	7.03(1.42 - 15.37)	6.24(1.23 - 13.77)	High FPG	9.98(2.09 - 21.30)	9.11(19.59 - 1.87)
**Prostate cancer**	Smoking	8.29(3.73 - 12.59)	8.82(3.99 - 13.38)	Smoking	6.01(2.71 - 9.29)	6.60(3.02 - 10.10)

BMI body - mass index; *DALYs* disability - adjusted life - years; *FPG* fasting plasma glucose *UI* uncertainty interval
