# Supplementary material for: Global, regional, and national burden of kidney, bladder, and prostate cancers and their attributable risk factors, 1990–2019
Source: Mil Med Res. 2021 Nov 24;8:60. doi: 10.1186/s40779-021-00354-z (PMC8611255; doi:10.1186/s40779-021-00354-z)
Supplement: Supplementary file 2 — Additional file 2. Fig. S1. Percentage of incident cases, deaths, and DALYs for genitourinary cancers in 1990 and 2019. a Incident cases. b Deaths. c DALYs. DALYs disability-adjusted life-years. Fig. S2. Global ASIR, ASMR, and ASDR of genitourinary cancers from 1990 to 2019. a ASIR. b ASMR. c ASDR. ASIR age-standardized incidence rate, ASMR age-standardized mortality rate, ASDR age-standardized DALYs rate, DALYs disability-adjusted life-years. Fig. S3. EAPC of ASMR for genitourinary cancers in global and 21 regions. a Kidney cancer. b Bladder cancer. c Prostate cancer. ASMR age-standardized mortality rate, EAPC estimated annual percentage change. Fig. S4. EAPC of ASDR for genitourinary cancers in global and 21 regions. a Kidney cancer. b Bladder cancer. c Prostate cancer. ASDR age-standardized DALYs rate, EAPC estimated annual percentage change. Fig. S5. Burden of kidney cancer by SDI quintile from 1990 to 2019. DALYs disability-adjusted life-years, SDI sociodemographic index. Fig. S6. Burden of bladder cancer by SDI quintile from 1990 to 2019. DALYs disability-adjusted life-years, SDI sociodemographic index. Fig. S7. Burden of prostate cancer by SDI quintile from 1990 to 2019. DALYs disability-adjusted life-years, SDI sociodemographic index. Fig. S8. ASMR of kidney cancer for 21 regions and 204 countries and territories by SDI. a 21 regions by SDI from 1990 to 2019. b 204 countries and territories by SDI in 2019. ASMR age-standardized mortality rate, SDI sociodemographic index. Fig. S9. ASDR of kidney cancer for 21 regions and 204 countries and territories by SDI. a 21 regions by SDI from 1990 to 2019. b 204 countries and territories by SDI in 2019. ASDR age-standardized DALYs rate, SDI sociodemographic index. Fig. S10. ASIR of bladder cancer for 21 regions and 204 countries and territories by SDI. a 21 regions by SDI from 1990 to 2019. b 204 countries and territories by SDI in 2019. ASIR age-standardized incidence rate, SDI sociodemographic index. Fig. S11. ASIR o [file 40779_2021_354_MOESM2_ESM.pdf]

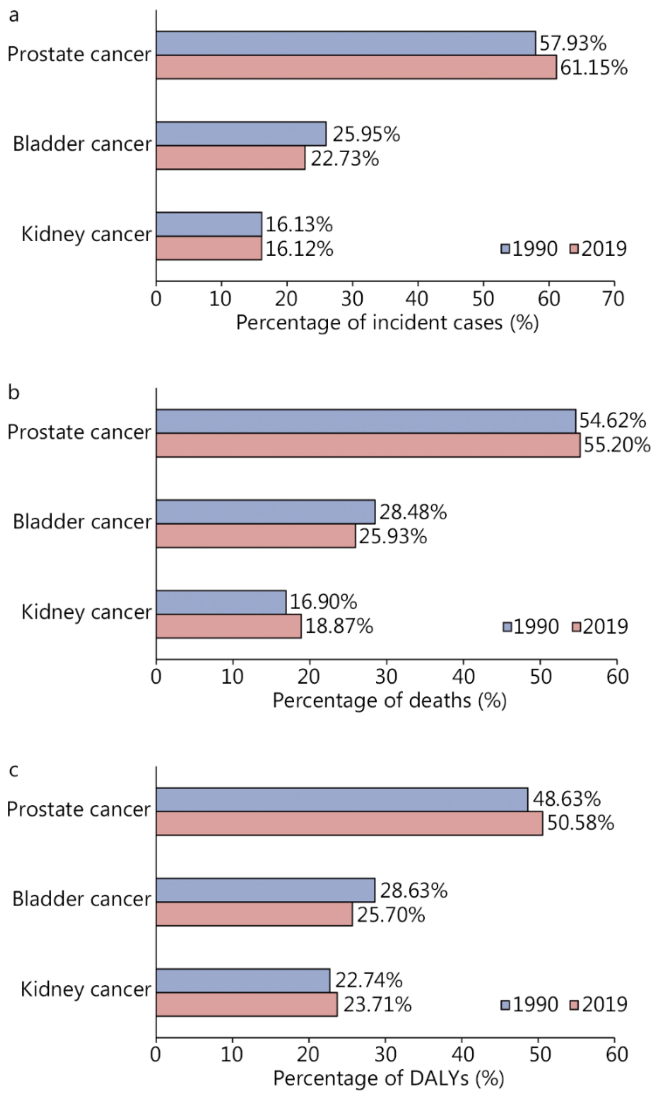

Fig. S1 Percentage of incident cases, deaths, and DALYs for genitourinary cancers in 1990 and 2019. **a** Incident cases. **b** Deaths. **c** DALYs. DALYs disability-adjusted life-years

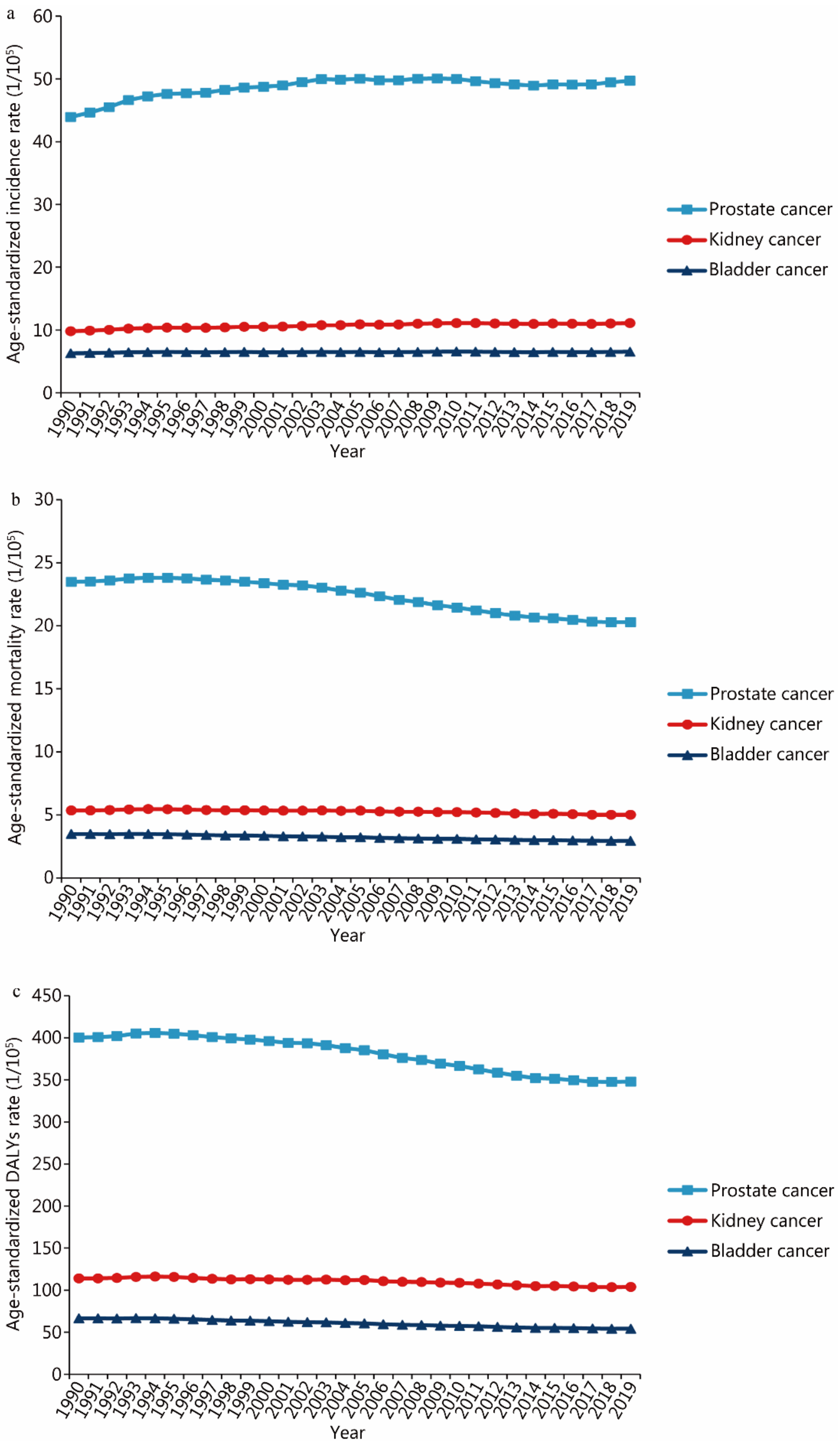

Fig. S2 Global ASIR, ASMR, and ASDR of genitourinary cancers from 1990 to 2019. **a** ASIR. **b** ASMR. **c** ASDR. ASIR age-standardized incidence rate, ASMR age-standardized mortality rate, ASDR age-standardized DALYs rate, DALYs disability-adjusted life-years

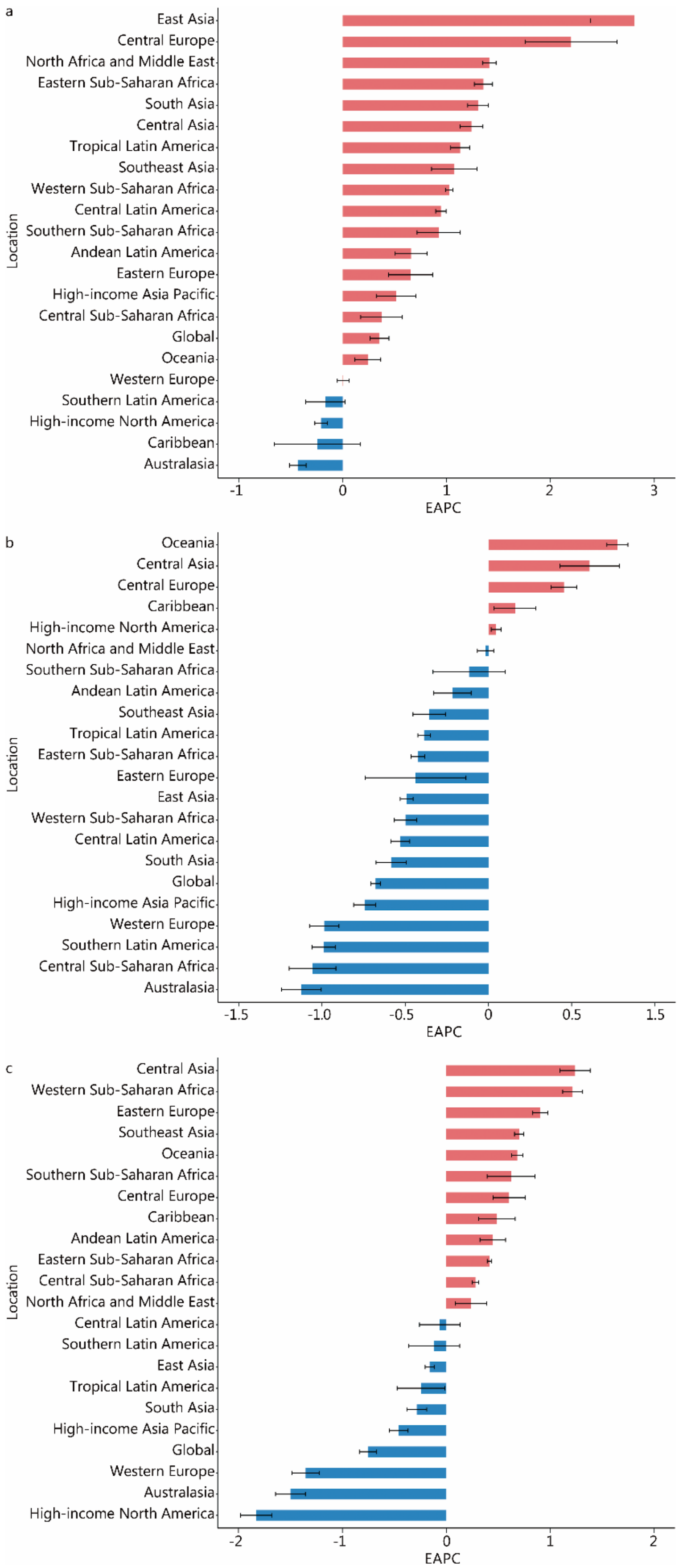

Fig. S3 EAPC of ASMR for genitourinary cancers in global and 21 regions. a Kidney cancer. b Bladder cancer. c Prostate cancer. ASMR age-standardized mortality rate, EAPC estimated annual percentage change

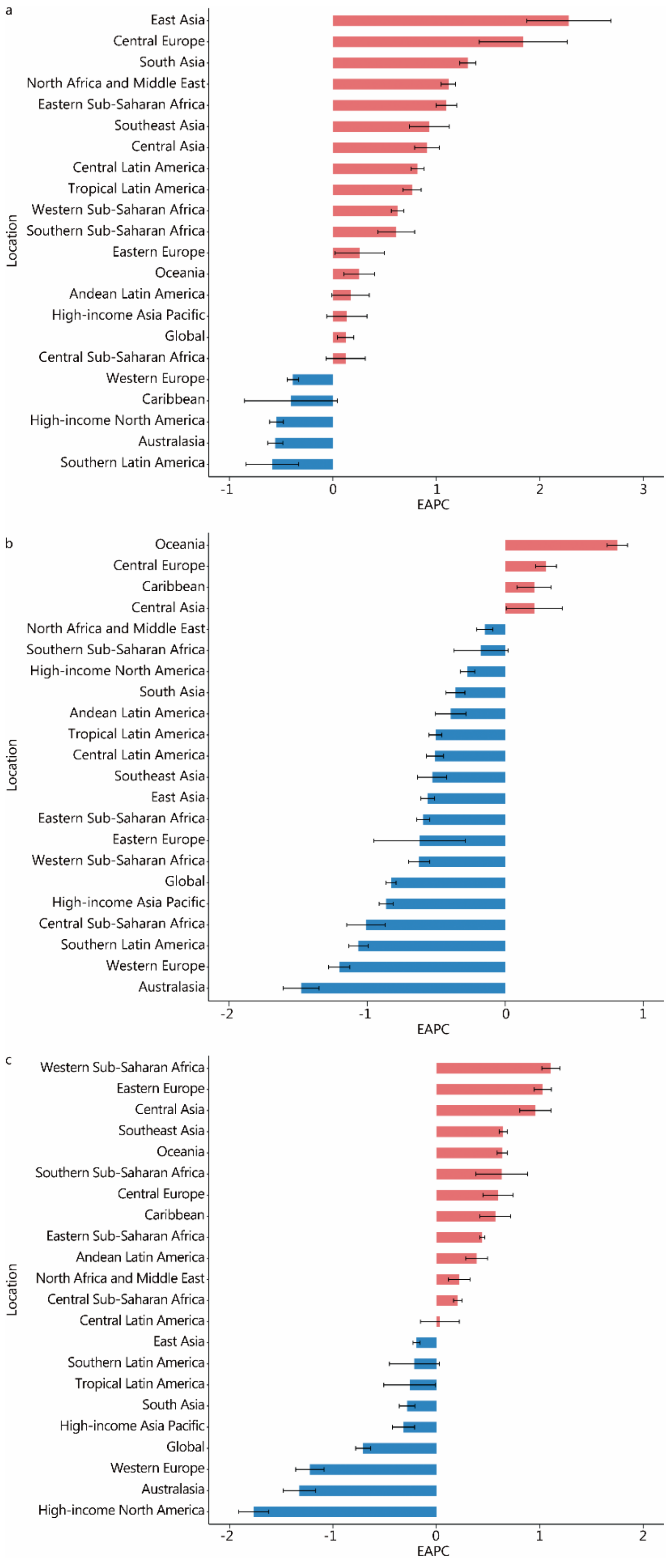

Fig. S4 EAPC of ASDR for genitourinary cancers in global and 21 regions. a Kidney cancer. b Bladder cancer. c Prostate cancer. ASDR age-standardized DALYs rate, EAPC estimated annual percentage change

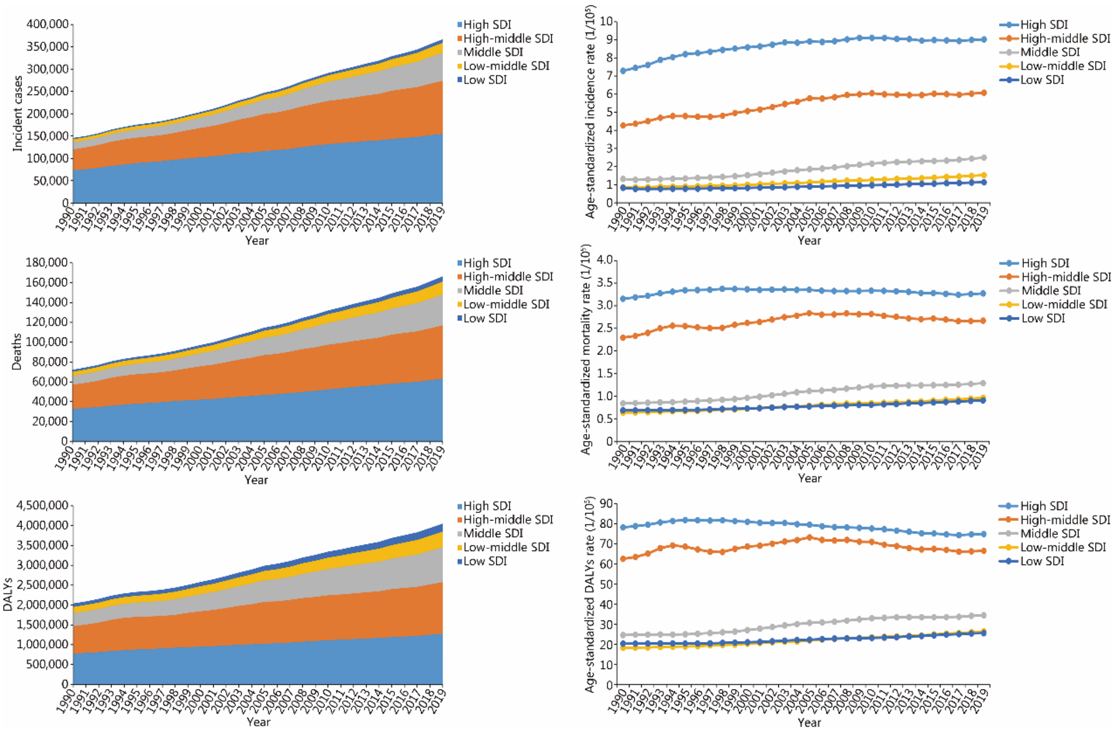

Fig. S5 Burden of kidney cancer by SDI quintile from 1990 to 2019. DALYs disability-adjusted life-years, SDI sociodemographic index

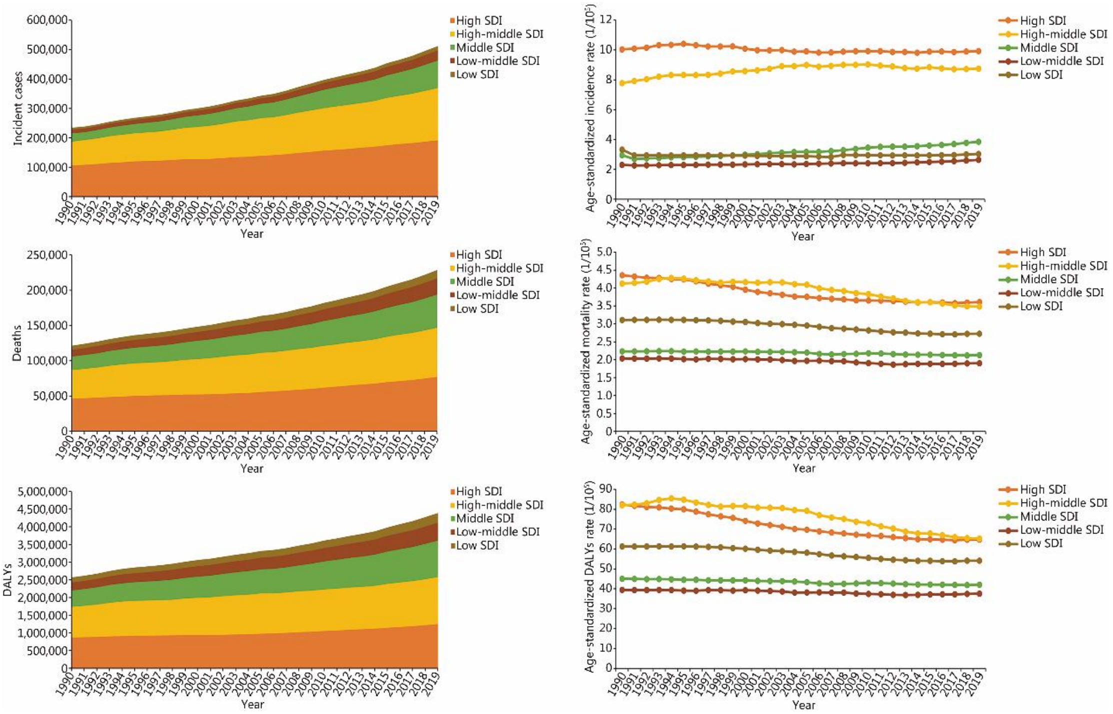

Fig. S6 Burden of bladder cancer by SDI quintile from 1990 to 2019. DALYs disability-adjusted life-years, SDI sociodemographic index

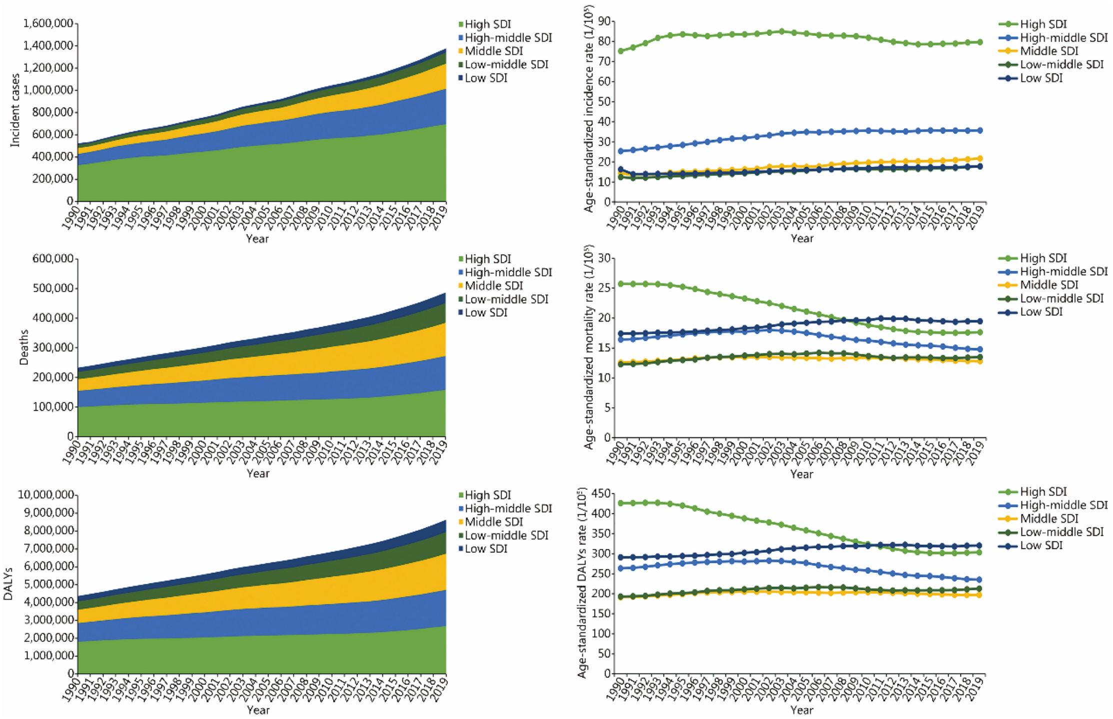

Fig. S7 Burden of prostate cancer by SDI quintile from 1990 to 2019. DALYs disability-adjusted life-years, SDI sociodemographic index

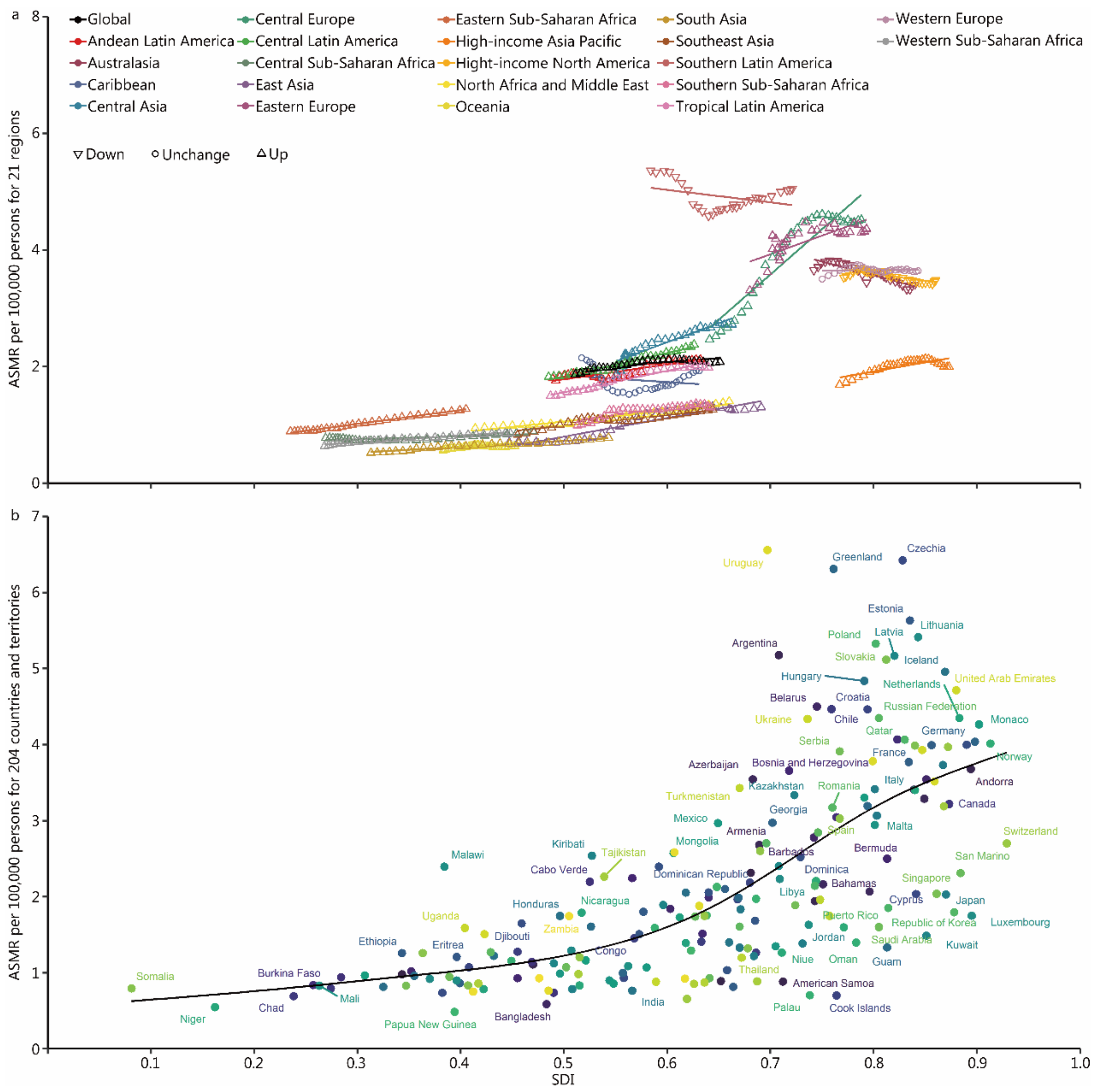

Fig. S8 ASMR of kidney cancer for 21 regions and 204 countries and territories by SDI. **a** 21 regions by SDI from 1990 to 2019. **b** 204 countries and territories by SDI in 2019. ASMR age-standardized mortality rate, SDI sociodemographic index

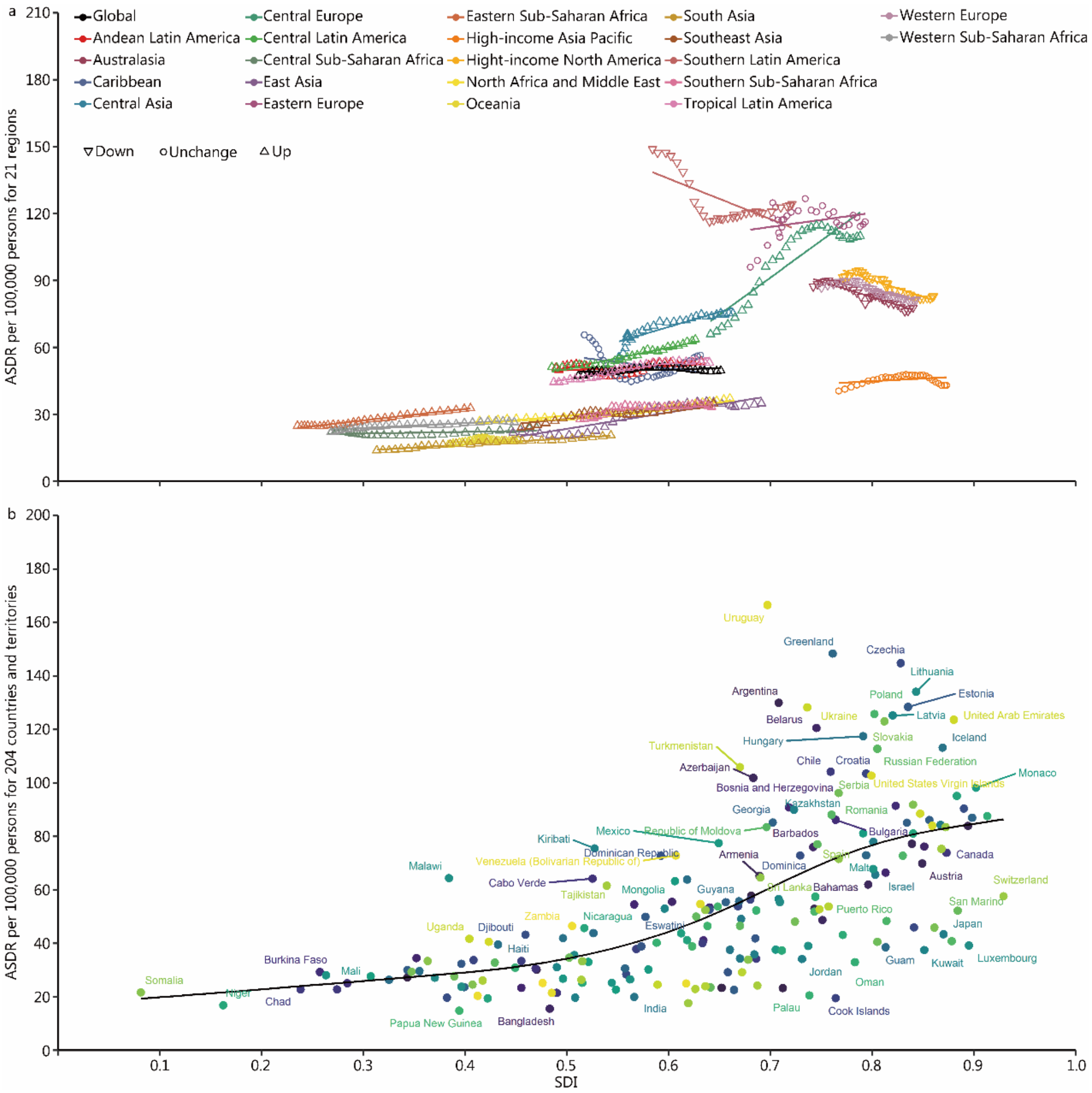

Fig. S9 ASDR of kidney cancer for 21 regions and 204 countries and territories by SDI. **a** 21 regions by SDI from 1990 to 2019. **b** 204 countries and territories by SDI in 2019. ASDR age-standardized DALYs rate, SDI sociodemographic index

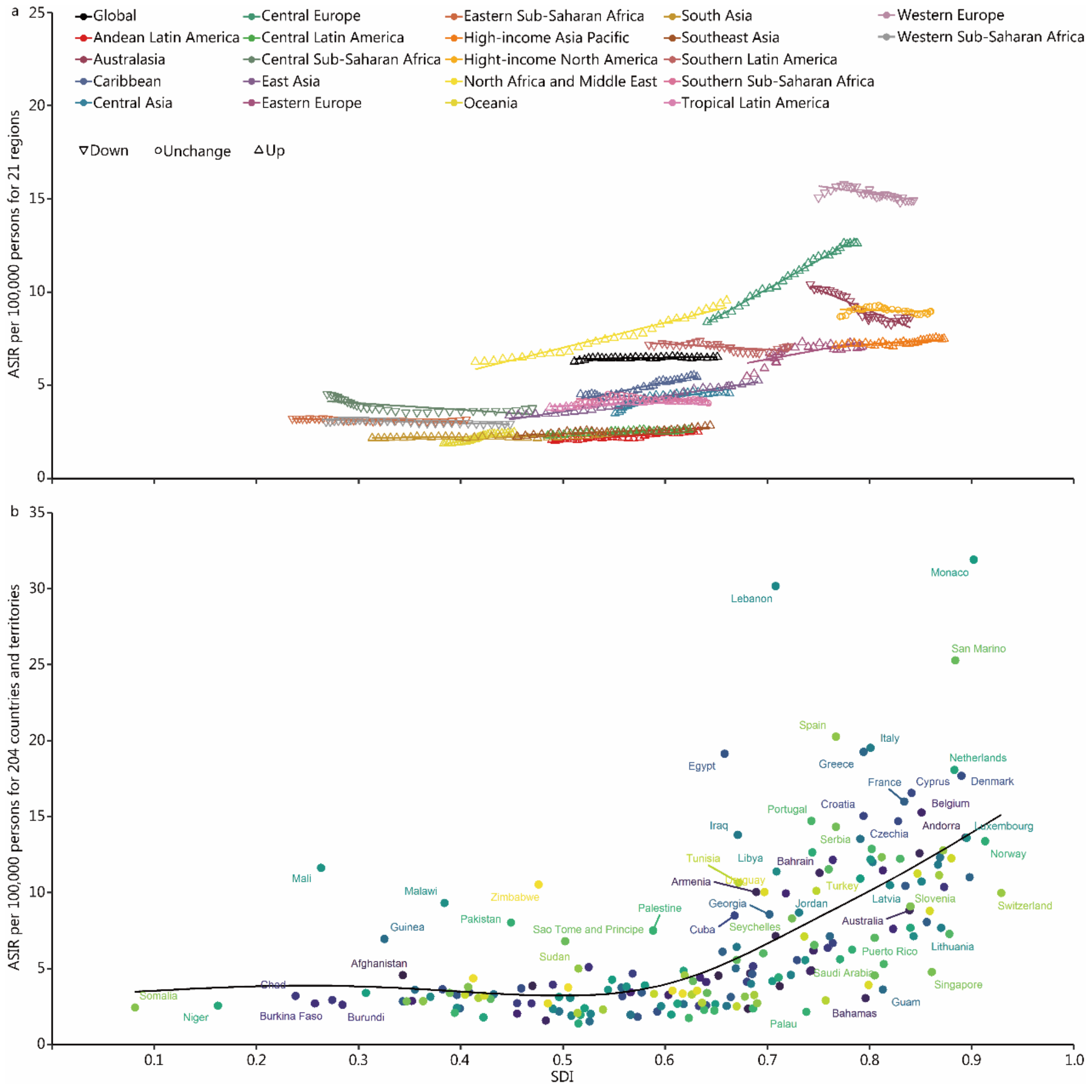

Fig. S10 ASIR of bladder cancer for 21 regions and 204 countries and territories by SDI. **a** 21 regions by SDI from 1990 to 2019. **b** 204 countries and territories by SDI in 2019. ASIR age-standardized incidence rate, SDI sociodemographic index

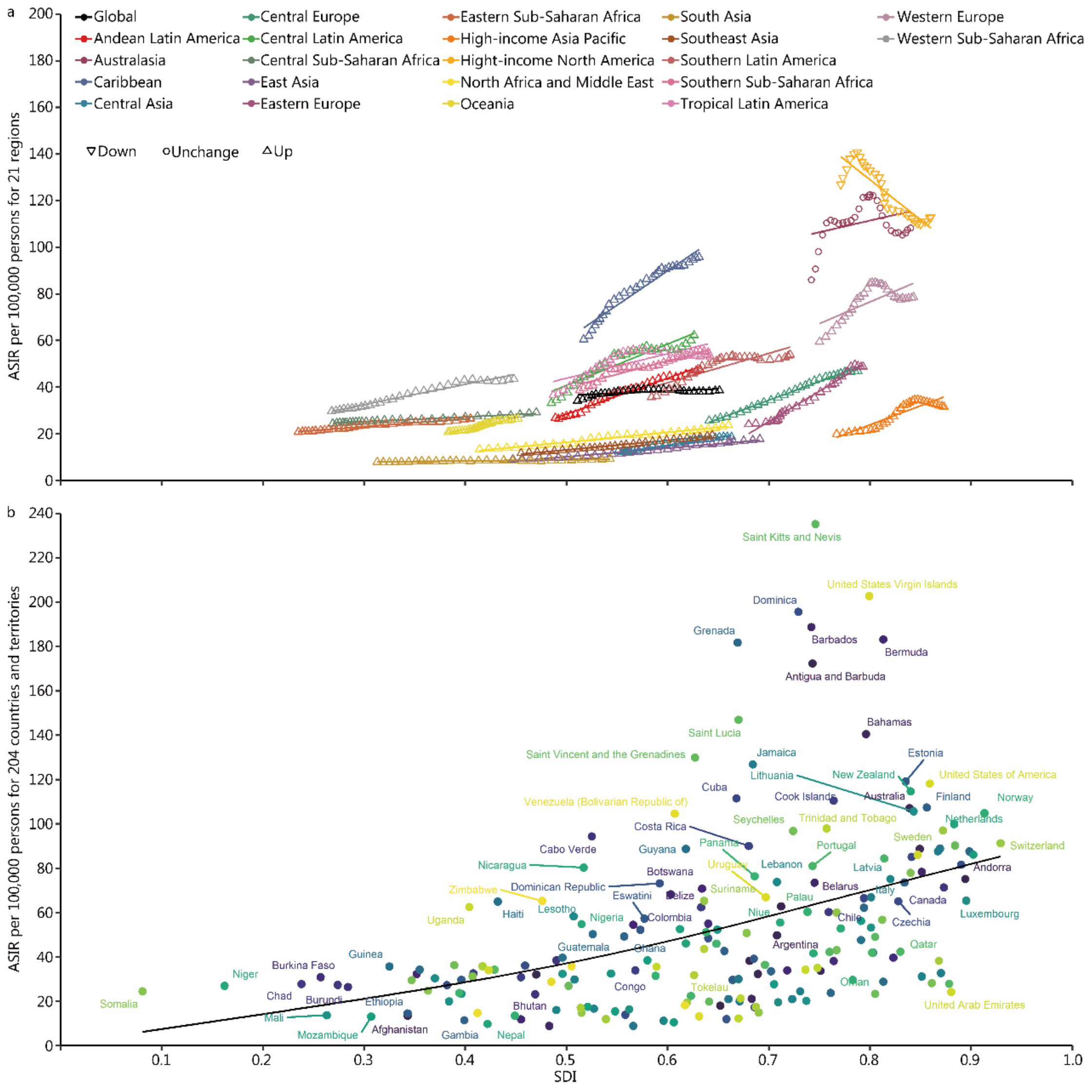

Fig. S11 ASIR of prostate cancer for 21 regions and 204 countries and territories by SDI. **a** 21 regions by SDI from 1990 to 2019. **b** 204 countries and territories by SDI in 2019. ASIR age-standardized incidence rate, SDI sociodemographic index

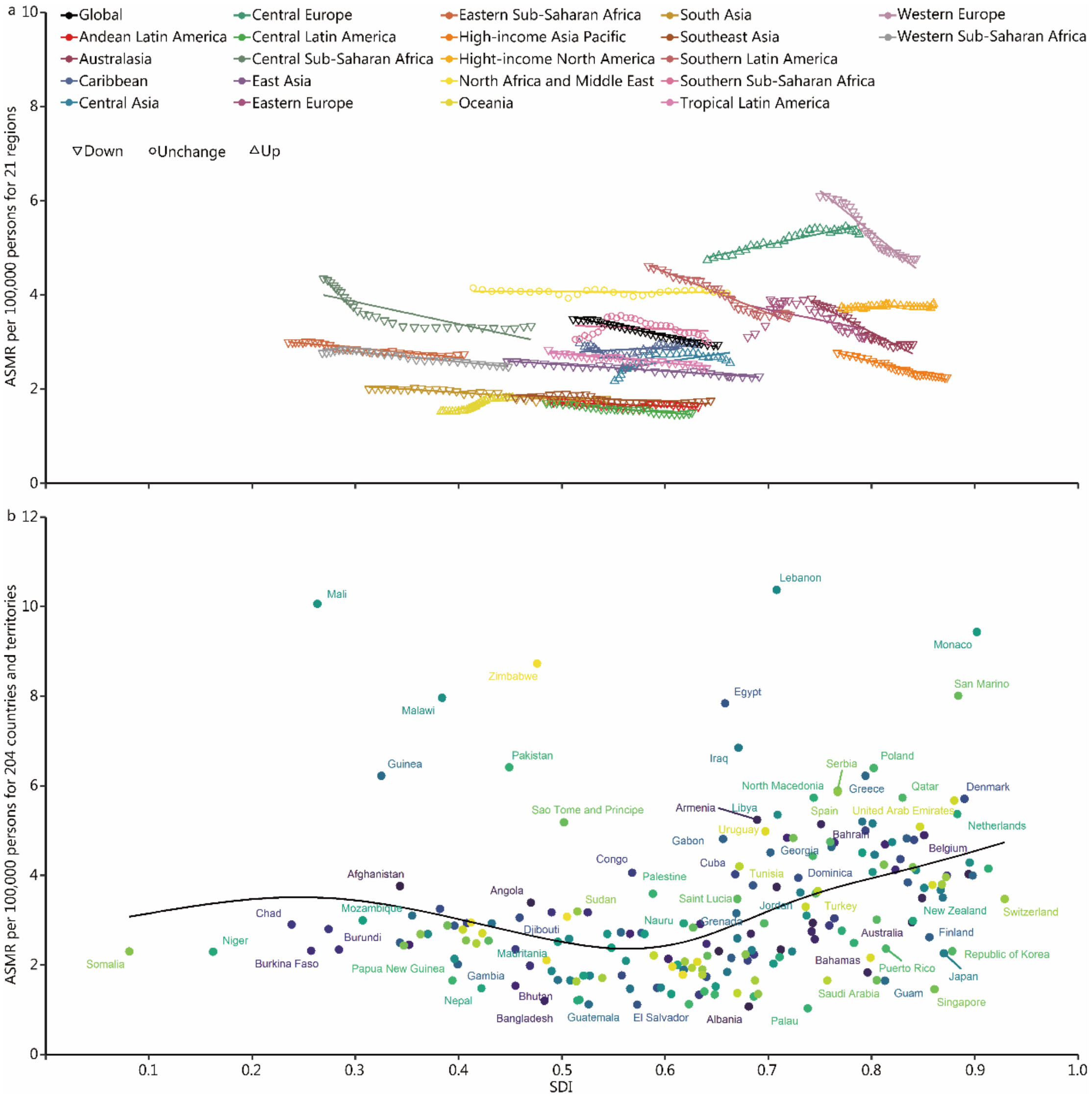

Fig. S12 ASMR of bladder cancer for 21 regions and 204 countries and territories by SDI. **a** 21 regions by SDI from 1990 to 2019. **b** 204 countries and territories by SDI in 2019. ASMR age-standardized mortality rate, SDI sociodemographic index

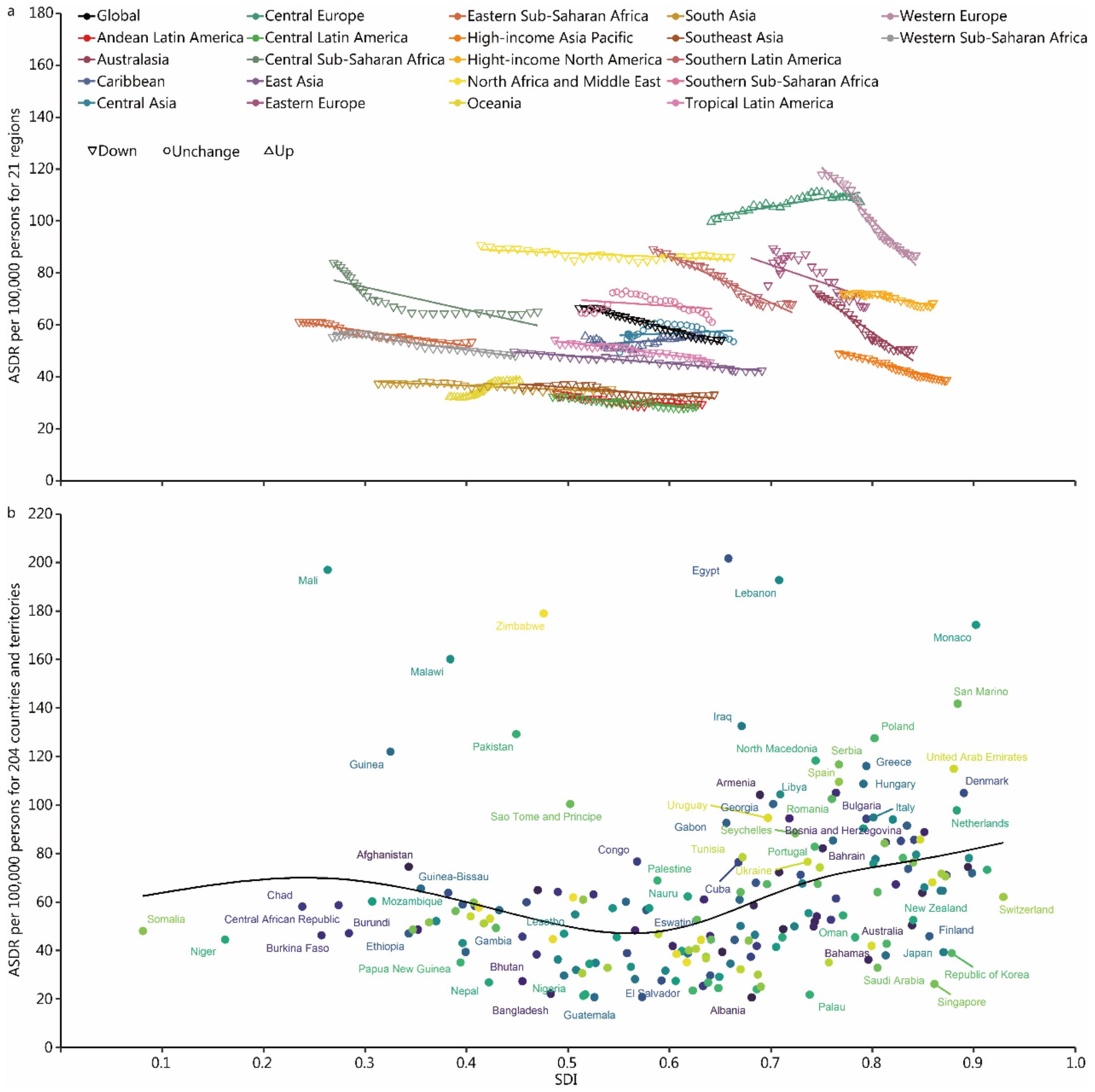

Fig. S13 ASDR of bladder cancer for 21 regions and 204 countries and territories by SDI. **a** 21 regions by SDI from 1990 to 2019. **b** 204 countries and territories by SDI in 2019. ASDR age-standardized DALYs rate, SDI sociodemographic index

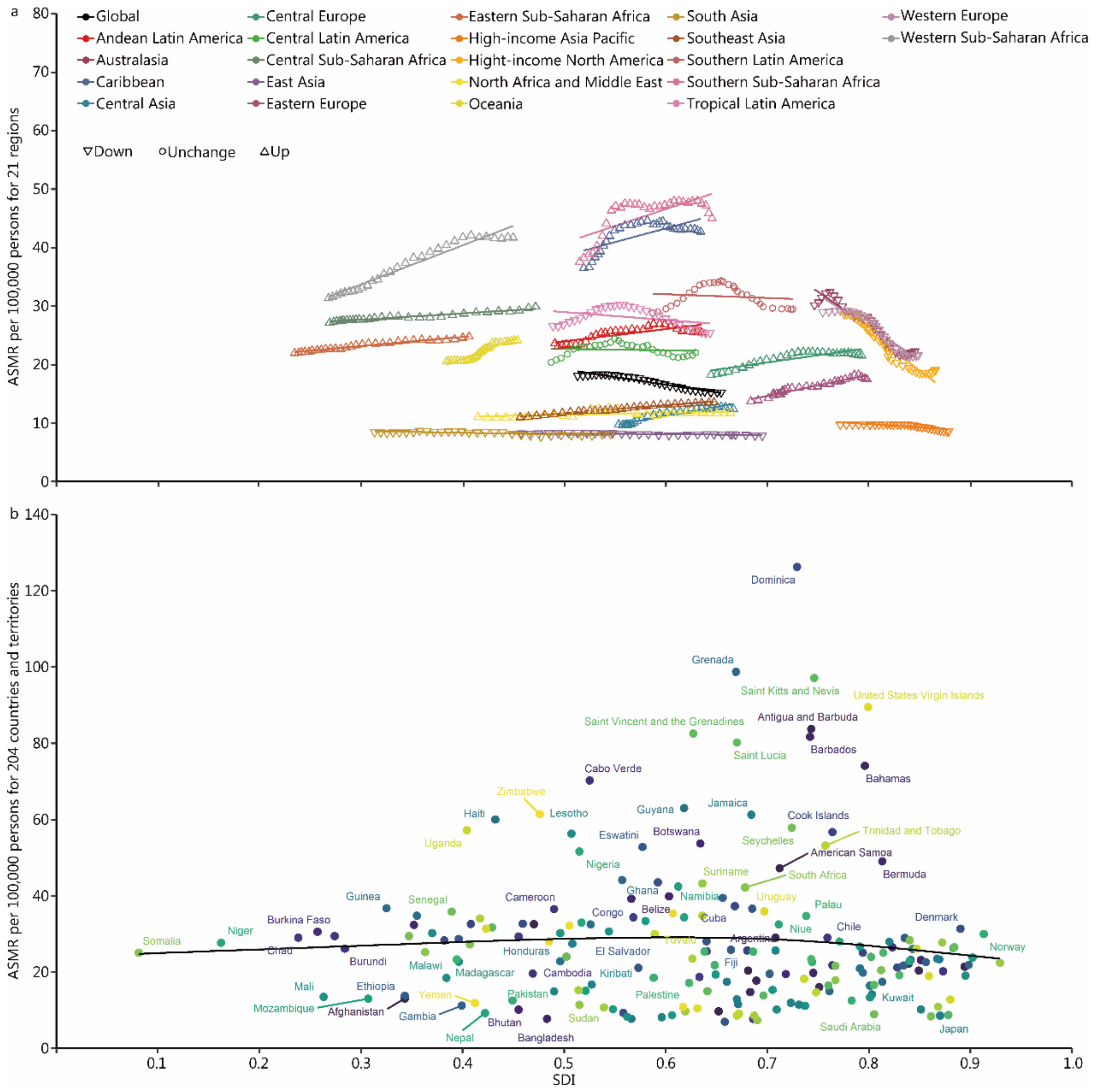

Fig. S14 ASMR of prostate cancer for 21 regions and 204 countries and territories by SDI. **a** 21 regions by SDI from 1990 to 2019. **b** 204 countries and territories by SDI in 2019. ASMR age-standardized mortality rate, SDI sociodemographic index

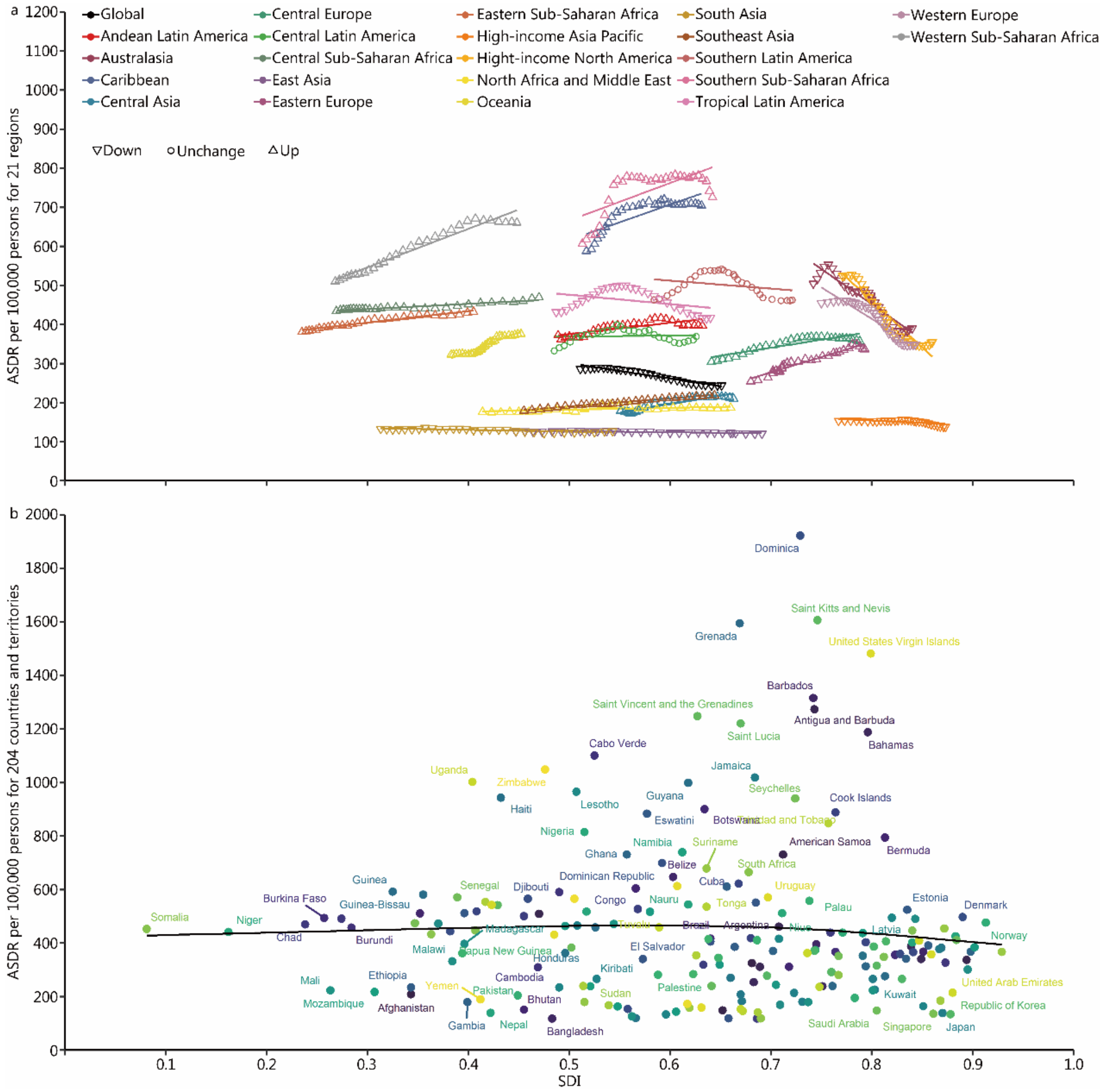

Fig. S15 ASDR of prostate cancer for 21 regions and 204 countries and territories by SDI. **a** 21 regions by SDI from 1990 to 2019. **b** 204 countries and territories by SDI in 2019. ASDR age-standardized DALYs rate, SDI sociodemographic index

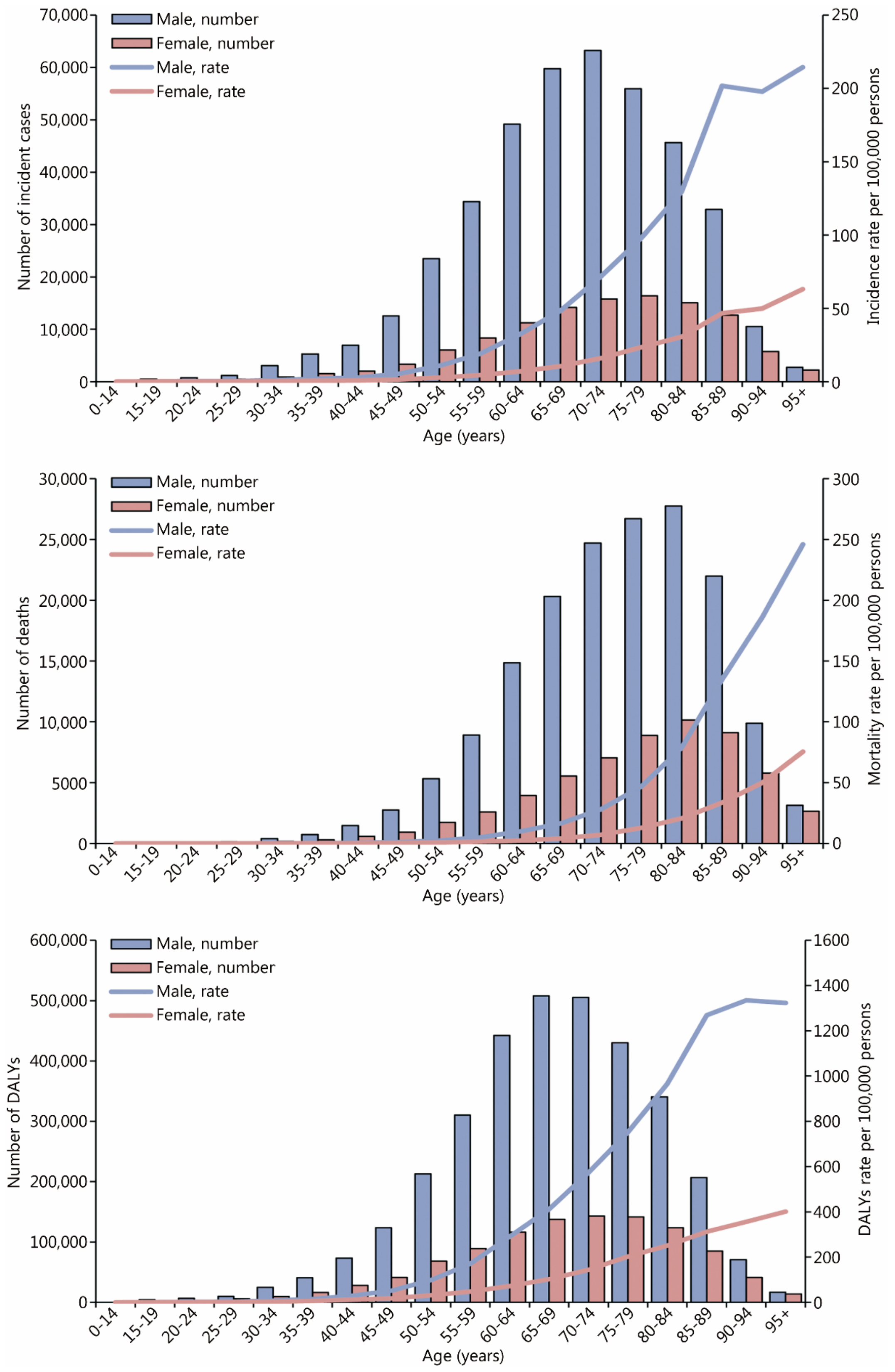

Fig. S16 Global incidence, mortality, and DALYs of bladder cancer by age and sex in 2019. DALYs disability-adjusted life-years

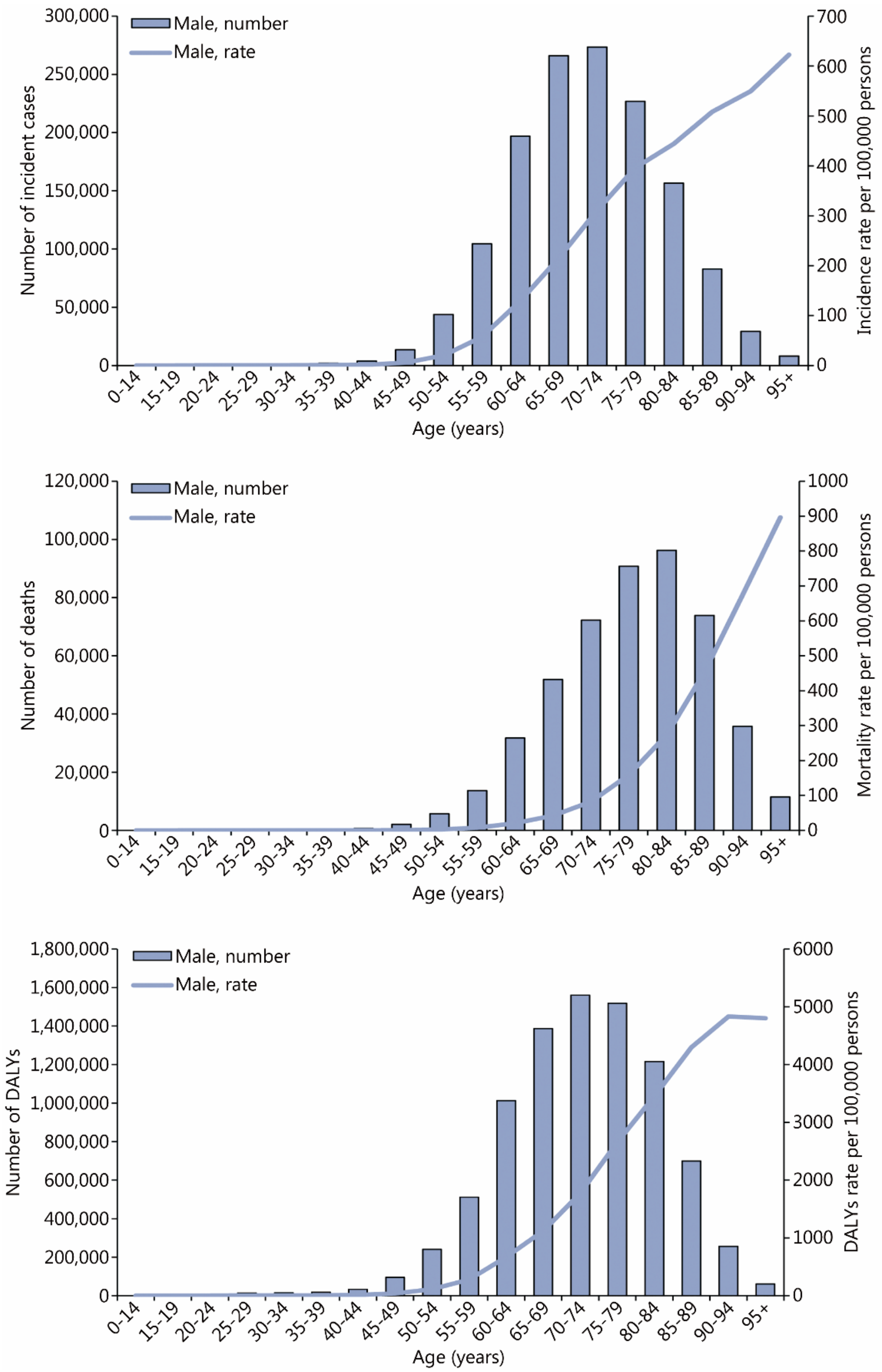

Fig. S17 Global incidence, mortality, and DALYs of prostate cancer by age and sex in 2019. DALYs disability-adjusted life-years
